# Association of circulating fatty acids with cardiovascular disease risk: analysis of individual-level data in three large prospective cohorts and updated meta-analysis

**DOI:** 10.1093/eurjpc/zwae315

**Published:** 2024-10-04

**Authors:** Fanchao Shi, Rajiv Chowdhury, Eleni Sofianopoulou, Albert Koulman, Luanluan Sun, Marinka Steur, Krasimira Aleksandrova, Christina C Dahm, Matthias B Schulze, Yvonne T van der Schouw, Claudia Agnoli, Pilar Amiano, Jolanda M A Boer, Christian S Bork, Natalia Cabrera-Castro, Fabian Eichelmann, Alexis Elbaz, Marta Farràs, Alicia K Heath, Rudolf Kaaks, Verena Katzke, Pekka Keski-Rahkonen, Giovanna Masala, Conchi Moreno-Iribas, Salvatore Panico, Keren Papier, Dafina Petrova, J Ramón Quirós, Fulvio Ricceri, Gianluca Severi, Anne Tjønneland, Tammy Y N Tong, Rosario Tumino, Nicholas J Wareham, Elisabete Weiderpass, Emanuele Di Angelantonio, Nita G Forouhi, John Danesh, Adam S Butterworth, Stephen Kaptoge

**Affiliations:** BHF Cardiovascular Epidemiology Unit, Department of Public Health and Primary Care, University of Cambridge, Cambridge CB2 0BB, UK; Victor Phillip Dahdaleh Heart and Lung Research Institute, University of Cambridge, Papworth Road, Cambridge Biomedical Campus, Cambridge CB2 0BB, UK; Stempel College of Public Health and Social Work, Florida International University, Miami, FL, USA; BHF Cardiovascular Epidemiology Unit, Department of Public Health and Primary Care, University of Cambridge, Cambridge CB2 0BB, UK; Victor Phillip Dahdaleh Heart and Lung Research Institute, University of Cambridge, Papworth Road, Cambridge Biomedical Campus, Cambridge CB2 0BB, UK; Medical Research Council Epidemiology Unit, Institute of Metabolic Science, University of Cambridge, Cambridge, UK; BHF Cardiovascular Epidemiology Unit, Department of Public Health and Primary Care, University of Cambridge, Cambridge CB2 0BB, UK; Victor Phillip Dahdaleh Heart and Lung Research Institute, University of Cambridge, Papworth Road, Cambridge Biomedical Campus, Cambridge CB2 0BB, UK; Division of Human Nutrition and Health, Wageningen University, Wageningen, The Netherlands; Biomarkers and Metabolism Research Group, Department of Epidemiological Methods and Etiological Research, Leibniz Institute for Prevention Research and Epidemiology, Bremen, Germany; Faculty of Human and Health Sciences, University of Bremen, Bremen, Germany; Department of Public Health, Aarhus University, Aarhus, Denmark; Department of Molecular Epidemiology, German Institute of Human Nutrition Potsdam-Rehbruecke, Nuthetal, Germany; Institute of Nutritional Science, University of Potsdam, Nuthetal, Germany; Julius Center for Health Sciences and Primary Care, University Medical Center Utrecht, Utrecht University, Utrecht, The Netherlands; Epidemiology and Prevention Unit, Fondazione IRCCS Istituto Nazionale dei Tumori, Milan, Italy; Centro de Investigación Biomédica en Red de Epidemiología y Salud Pública (CIBERESP), Madrid, Spain; Ministry of Health of the Basque Government Sub Directorate for Public Health and Addictions of Gipuzkoa, San Sebastian, Spain; Biodonostia Health Research Institute, Epidemiology of Chronic and Communicable Diseases Group, San Sebastian, Spain; National Institute of Public Health and the Environment (RIVM), Bilthoven, The Netherlands; Department of Cardiology, Aalborg University Hospital, Aalborg, Denmark; Centro de Investigación Biomédica en Red de Epidemiología y Salud Pública (CIBERESP), Madrid, Spain; Department of Epidemiology, Regional Health Council, IMIB-Arrixaca, Murcia, Spain; Department of Molecular Epidemiology, German Institute of Human Nutrition Potsdam-Rehbruecke, Nuthetal, Germany; German Center for Diabetes Research (DZD), Neuherberg, Germany; Paris-Saclay University, UVSQ, Inserm, Gustave Roussy, CESP, Villejuif, France; Unit of Nutrition and Cancer, Epidemiology Research Program, Catalan Institute of Oncology (ICO), Bellvitge Biomedical Research Institute (IDIBELL), 08908 L’Hospitalet de Llobregat, Spain; Department of Epidemiology and Biostatistics, School of Public Health, Imperial College London, London, UK; Department of Cancer Epidemiology, German Cancer Research Center (DKFZ), Heidelberg, Germany; Department of Cancer Epidemiology, German Cancer Research Center (DKFZ), Heidelberg, Germany; Nutrition and Metabolism Branch, International Agency for Research on Cancer (IARC/WHO), Lyon, France; Clinical Epidemiology Unit, Institute for cancer research, prevention and clinical network (ISPRO), Florence, Italy; Instituto de Salud Pública y Laboral de Navarra, 31003 Pamplona, Spain; Centro de Investigación Biomédica en Red de Epidemiología y Salud Pública (CIBERESP), 28029 Madrid, Spain; Navarra Institute for Health Research (IdiSNA), 31008 Pamplona, Spain; School of Medicine, Federico II University, Naples, Italy; Cancer Epidemiology Unit, Nuffield Department of Population Health, University of Oxford, Oxford, UK; Centro de Investigación Biomédica en Red de Epidemiología y Salud Pública (CIBERESP), 28029 Madrid, Spain; Escuela Andaluza de Salud Pública (EASP), 18011 Granada, Spain; Instituto de Investigación Biosanitaria ibsGRANADA, 18012 Granada, Spain; Public Health Directorate, Asturias, Spain; Centre for Biostatistics, Epidemiology, and Public Health (C-BEPH), Department of Clinical and Biological Sciences, University of Turin, Turin, Italy; Paris-Saclay University, UVSQ, Inserm, Gustave Roussy, CESP, Villejuif, France; Department of Statistics, Computer Science, Applications ‘G Parenti’, University of Florence, Florence, Italy; The Danish Cancer Institute, Strandboulevarden 49, 2100 Copenhagen O, Denmark; Department of Public Health, University of Copenghagen, Copenhagen, Denmark; Cancer Epidemiology Unit, Nuffield Department of Population Health, University of Oxford, Oxford, UK; Hyblean Association for Epidemiological Research (AIRE-ONLUS), Ragusa, Italy; Medical Research Council Epidemiology Unit, Institute of Metabolic Science, University of Cambridge, Cambridge, UK; International Agency for Research on Cancer (IARC/WHO), Lyon, France; BHF Cardiovascular Epidemiology Unit, Department of Public Health and Primary Care, University of Cambridge, Cambridge CB2 0BB, UK; Victor Phillip Dahdaleh Heart and Lung Research Institute, University of Cambridge, Papworth Road, Cambridge Biomedical Campus, Cambridge CB2 0BB, UK; BHF Centre of Research Excellence, School of Clinical Medicine, Addenbrooke's Hospital, University of Cambridge, Cambridge, UK; Health Data Research UK Cambridge, Wellcome Genome Campus and University of Cambridge, Hinxton, UK; NIHR Blood and Transplant Research Unit in Donor Health and Behaviour, University of Cambridge, Cambridge CB2 0BB, UK; Health Data Science Centre, Human Technopole, Milan, Italy; Medical Research Council Epidemiology Unit, Institute of Metabolic Science, University of Cambridge, Cambridge, UK; BHF Cardiovascular Epidemiology Unit, Department of Public Health and Primary Care, University of Cambridge, Cambridge CB2 0BB, UK; Victor Phillip Dahdaleh Heart and Lung Research Institute, University of Cambridge, Papworth Road, Cambridge Biomedical Campus, Cambridge CB2 0BB, UK; BHF Centre of Research Excellence, School of Clinical Medicine, Addenbrooke's Hospital, University of Cambridge, Cambridge, UK; Health Data Research UK Cambridge, Wellcome Genome Campus and University of Cambridge, Hinxton, UK; NIHR Blood and Transplant Research Unit in Donor Health and Behaviour, University of Cambridge, Cambridge CB2 0BB, UK; Department of Human Genetics, Wellcome Sanger Institute, Hinxton, UK; BHF Cardiovascular Epidemiology Unit, Department of Public Health and Primary Care, University of Cambridge, Cambridge CB2 0BB, UK; Victor Phillip Dahdaleh Heart and Lung Research Institute, University of Cambridge, Papworth Road, Cambridge Biomedical Campus, Cambridge CB2 0BB, UK; BHF Centre of Research Excellence, School of Clinical Medicine, Addenbrooke's Hospital, University of Cambridge, Cambridge, UK; Health Data Research UK Cambridge, Wellcome Genome Campus and University of Cambridge, Hinxton, UK; NIHR Blood and Transplant Research Unit in Donor Health and Behaviour, University of Cambridge, Cambridge CB2 0BB, UK; BHF Cardiovascular Epidemiology Unit, Department of Public Health and Primary Care, University of Cambridge, Cambridge CB2 0BB, UK; Victor Phillip Dahdaleh Heart and Lung Research Institute, University of Cambridge, Papworth Road, Cambridge Biomedical Campus, Cambridge CB2 0BB, UK; NIHR Blood and Transplant Research Unit in Donor Health and Behaviour, University of Cambridge, Cambridge CB2 0BB, UK

**Keywords:** Fatty acids, Cardiovascular disease, Coronary heart disease, Stroke, Cohort study, Meta-analysis

## Abstract

**Aims:**

Associations of saturated and unsaturated fatty acids (FAs) with cardiovascular disease (CVD) remain controversial. We therefore aimed to investigate the prospective associations of objectively measured FAs with CVD, including incident coronary heart disease (CHD) and stroke, as well as CVD mortality.

**Methods and results:**

Circulating FA concentrations expressed as the percentage of total FAs were assayed in 172 891 participants without prior vascular disease at baseline from the European Prospective Investigation into Cancer and Nutrition-CVD (EPIC-CVD) (7343 CHD; 6499 stroke), UK Biobank (1825; 1474), and INTERVAL (285; 209) cohort studies. Hazard ratio (HR) per 1-standard deviation (SD) higher FA concentrations was estimated using Cox regression models and pooled by random-effects meta-analysis. Systematic reviews with meta-analysis published by 6 May 2023 on associations between FAs and CVDs were systematically searched and updated meta-analyses using random-effects model were conducted. Evidence from randomized controlled trials (RCTs) was also summarized. Higher concentrations of total saturated FAs (SFAs) were associated with higher cardiovascular risks in the combined analysis, with differential findings noted for SFA sub-types in further analysis restricted to EPIC-CVD: positive associations for even-chain SFA [HR for CHD 1.24 (95% CI: 1.18–1.32); stroke 1.23 (1.10–1.38)] and negative associations for odd-chain [0.82 (0.76–0.87); 0.73 (0.67–0.78)] and longer-chain [0.95 (0.80–1.12); 0.84 (0.72–0.99)] SFA. In the combined analysis, total *n*-3 polyunsaturated FA (PUFA) [0.91 (0.85–0.97)], including docosahexaenoic acid (DHA) [0.91 (0.84–0.98)], was negatively associated with incident CHD risk. Similarly, total *n*-6 PUFA [0.94 (0.91–0.98)], including linoleic acid (LA) [0.89 (0.83–0.95)], was negatively associated with incident stroke risk. In contrast, more detailed analyses in EPIC-CVD revealed that several downstream *n*-6 PUFAs of LA were positively associated with CHD risk. Updated meta-analyses of 37 FAs including 49 non-overlapping studies, involving between 7787 and 22 802 CHD cases and between 6499 and 14 221 stroke cases, showed broadly similar results as our combined empirical analysis and further suggested significant inverse associations of individual long-chain *n*-3 PUFAs and LA on both CHD and stroke. The findings of long-chain *n*-3 PUFAs were consistent with those from published RCTs on CHD despite insufficient evidence in monotherapy, while RCT evidence remained unclear for the rest of the explored FAs.

**Conclusion:**

Our study provides an overview of the most recent evidence on the associations between objectively measured FAs and CVD outcomes. Collectively, the data reveal notable differences in associations by SFA sub-types and call for further studies, especially RCTs, to explore these links.

## Graphical Abstract

**Graphical Abstract:**
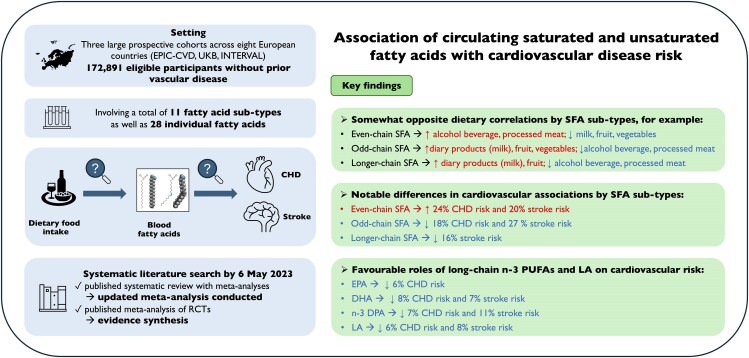

**See the editorial comment for this article Saturated vs. unsaturated fatty acids: should we reconsider their cardiovascular effects?', by A. Pirillo and A.L. Catapano, https://doi.org/10.1093/eurjpc/zwae340.**

## Introduction

Cardiovascular disease (CVD), including coronary heart disease (CHD) and stroke, remains the leading cause of morbidity and mortality globally.^1^ As a potentially modifiable factor, dietary fatty acid (FA) intakes and their associations with cardiometabolic health have been investigated for decades.^2,3^ It has been well recognized that FAs are not only energy-bearing nutrients, but can also modulate major circulating lipids including low-density lipoprotein cholesterol (LDL-C), high-density lipoprotein cholesterol (HDL-C), and triglycerides (TG)^4^ that may have an important role in the development and progression of CVD.

However, guidelines on dietary FA intake for CVD prevention remain controversial and inconsistent,^5^ at least in part reflecting the inconclusive evidence from observational investigations and randomized controlled trials (RCTs).^6^ Despite long-lasting beliefs concerning the beneficial properties of *n*-3 polyunsaturated FA (PUFA) intake on CVD, findings from different studies are still mixed.^2^ Meanwhile, most RCTs were conducted among patients with pre-existing CVD or at high CVD risk and evidence for general population has been limited.^2^ Associations of *n*-6 PUFA intake with CVD have been also uncertain given conflicting evidence.^3^ Saturated fatty acids (SFAs) are thought to be detrimental to cardiovascular health and recommendations to limit SFA intake have persisted.^7^ However, mounting evidence suggests no strong benefits of reducing SFA intake on CVD risk,^8^ but debate continues.^5^ Additionally, studies on dietary FAs may be limited by the subjectiveness of dietary assessment and therefore overlook possible differences in endogenous metabolism and biological activities of individual FAs.^6,9,10^ Although relationships between objectively measured FAs and cardiovascular risks have been evaluated in some studies, current evidence remains limited by their sample size (relatively small numbers of cases), scale (inadequate precision), depth (inclusion of a small sub-set of FA sub-types), and quality (e.g. inability to account for regression dilution bias or insufficient confounder adjustments). There is also inconsistency in assay compartments (e.g. plasma, erythrocytes, or adipose tissue),^11^ and quantification methods of FAs (i.e. relative or absolute concentration).^4,12^ Moreover, most studies have focused primarily on CHD, with the relevance of most FAs on other vascular outcomes (e.g. stroke) being less frequently studied and remaining unclear.^2,3^

We therefore aimed to assess the associations of circulating FA sub-types, either individually or in combination, with incident cardiovascular events using individual data from three prospective cohorts with a combined sample size of 172 891 participants without prior vascular disease. We also conducted a systematic literature search and updated meta-analyses to contextualize our findings and provide a quantitative synthesis of all available evidence thus far.

## Methods

### Study design and population

We analysed individual participant data from three large prospective cohort studies that assayed plasma or serum FAs in blood samples collected at baseline, namely: the European Prospective Investigation into Cancer and Nutrition-CVD (EPIC-CVD) study, a case-cohort study of ∼30 000 participants nested within the pan-European EPIC study that recruited ∼520 000 participants between 1992 and 2000 from 10 European countries^13^; the UK Biobank (UKB) study, a large prospective cohort study of over 500 000 participants recruited between 2006 and 2010 from 22 centres across the UK^14^; and the INTERVAL study, a multi-centre prospective study of ∼45 000 participants initially recruited into a randomized trial of blood donation frequency between 2012 and 2014 at NHS blood and transplant blood donation centres across England.^15^ Details of each study have been described previously.^13–15^ To be eligible for inclusion in the current analyses, participants had to have information recorded on baseline FA measurements, history of vascular disease, and incident CVD outcomes. This resulted in 172 891 eligible participants without prior vascular disease (defined as any history of heart disease, stroke, transient ischemic attack, peripheral vascular disease, or cardiovascular surgery at baseline) (31 606 from EPIC-CVD, 99 762 from UKB, and 41 523 from INTERVAL) included in the analysis (see Supplementary material online, Supplementary Figure*S1*). All participating studies obtained informed consent from participants and received ethical approval from their local ethics committee.

### Assessment of fatty acids

In EPIC-CVD, 38 individual plasma phospholipid FAs were assayed using an established gas chromatography (GC) method^16^ and expressed as percentages (%) of total phospholipid FAs. Twenty-eight individual FAs with relative concentrations higher than 0.05% were retained in the current analysis and used to calculate the main FA sub-types (see Supplementary material online, Supplementary Table*S1* and Supplementary material online, Figure*S2*). In both UKB and INTERVAL, five FA sub-types and two individual FAs in total serum or plasma (i.e. all the FAs in TG, phospholipids, cholesterol esters, or as free FAs) were assayed using a high-throughput nuclear magnetic resonance (NMR) metabolomics platform (Nightingale Health Ltd., Helsinki, Finland)^17^ and expressed either as absolute concentrations (mmol/L) or relative concentrations of total FAs (%). We conducted combined analysis of the three data sources as prior experimental evidence has suggested highly comparable FAs concentrations in matched plasma and serum samples^18^ and also shown high correspondence of FA quantification between NMR and GC (Pearson correlation *r* > 0.92).^17^ Details of the FA measures in each study are provided in Supplementary Methods. To maximize statistical power, the current analysis first focused on seven FAs (%), individually or in groups, measured in all three studies: total SFA, total monounsaturated fatty acid (MUFA), total PUFA, total *n*-3 PUFA, total *n*-6 PUFA, docosahexaenoic acid (DHA), and linoleic acid (LA). Additionally, in EPIC-CVD it was possible to define three SFA sub-types by summation of components, namely: even-chain (sum of 14:0, 16:0, and 18:0), odd-chain (sum of 15:0 and 17:0) and longer-chain (sum of 20:0, 22:0, 23:0, and 24:0) SFAs.

### Ascertainment of outcome

Outcomes were ascertained through linkages to routinely available national datasets, questionnaires, or active follow-up and classified according to the International Classification of Diseases, Tenth Revision (ICD-10) codes (see Supplementary Methods). Primary outcomes included CHD defined as fatal ischaemic heart disease (ICD-10 code: I20-I25) or non-fatal myocardial infarction (I21-I23) and stroke defined as any cerebrovascular disease (I60-I69). Secondary outcomes included total CVD defined as any non-fatal or fatal cardiovascular events (I20-I69), CVD mortality (I21-I69), and stroke sub-types (i.e. ischaemic stroke (IS) (I63) and haemorrhagic stroke (HS) (I60-I61)).

### Statistical analysis

The analysis plan employed in the current study was based broadly on our earlier published analyses.^9,10^ To minimize heterogeneity across studies, FA concentrations were statistically standardized within each study before analysis (i.e. [value—mean]/SD where SD refers to standard deviation, with summary statistics in EPIC-CVD calculated in the sub-cohort). To investigate the relationships of dietary intakes with circulating FAs, semi-partial correlation coefficients were calculated in the sub-cohort of EPIC-CVD (*n* = 15 838 participants) by regressing FAs (%) on self-reported dietary food intakes (g/day), SFA, MUFA, and PUFA (%), respectively, adjusted for batch, sex, age, and total energy intake.

Associations of circulating FA sub-types, either individually or in combination, with CHD and stroke were assessed by random-effects meta-analysis of study and country-specific hazard ratios (HRs), calculated using Cox proportional hazards regression models with age as timescale and stratified by sex and, as appropriate, centre.^19^ Participants contributed follow up time from age at baseline survey to age at first occurrence of CHD, stroke, death or end of follow up. To account for the case-cohort design of EPIC-CVD, Cox models were adapted using the Prentice-weighted method.^9,10^ The shape of dose-response association between FAs and CHD and stroke was assessed by meta-analysis of fractional polynomials as described previously,^20^ adjusted for conventional risk factors. Assay batch group was additionally adjusted for in EPIC-CVD to account for potential batch effects. To maximize the available data and limit potential overadjustment for variables that could mediate associations between FAs and CVDs, the basic models were adjusted for conventional risk factors harmonized across three studies to the extent that was possible, namely: age (years), smoking status (current, non-current), history of diabetes (yes, no), history of hypertension (yes, no), and physical activity (active, inactive). Though history of hypertension and diabetes were not assessed in INTERVAL, participants were considered generally healthy as they were blood donors, with low prevalence expected. Missing data of categorical covariates were coded as a separate category in adjustments using dummy variables.

To further assess independence of the associations, HRs were additionally progressively adjusted for potential confounders or mediators on a complete-case basis, particularly: other non-lipid conventional CVD risk factors that may be related to diet, i.e. alcohol consumption (current, non-current) and body mass index (BMI; kg/m^2^); and lipid markers, i.e. total cholesterol (TC; mmol/L), HDL-C (mmol/L) and TG (mmol/L). In analyses restricted to EPIC-CVD, we also assessed the associations adjusted for dietary variables (intakes of fruit and vegetables, dairy products, meat, fish, olive oil, margarine, and alcoholic beverages [g/day]) before the lipid adjustment. Further analyses included the mutual adjustments of SFA sub-types and PUFA sub-types, respectively.

Effect modification was assessed using tests for interaction between FAs and several baseline characteristics, with the *P*-values for interaction calculated using continuous variables where applicable.^19^ A significance threshold of 0.0002 was used to interpret sub-group analysis results, corresponding to Bonferroni correction for 22 comparisons (11 sub-groups and 2 primary outcomes) at 0.05 nominal level in secondary analyses of each of 10 primary FAs exposures.

Sensitivity analyses: (i) calculated HRs excluding the initial 2 years of follow-up to assess potential reverse causality; (ii) assessed the associations independent of inflammation (as measured by C-reactive protein [CRP]) and either haemoglobin A1c (HbA1c) or glucose levels; (iii) compared associations of FAs expressed in absolute vs. relative concentrations in UKB and INTERVAL; (iv) calculated regression dilution ratios (RDRs) among 1187 UKB participants with repeat FA data and inferred the extent of possible aetiological associations corrected for long-term within-person variability of FA measurements^21^; and (v) adjusted for competing risks using the Fine and Gray regression model with censoring due to CHD, stroke, or death from causes other than the event of interest considered as competing events when relevant.

### Systematic review and updated meta-analysis

To contextualize findings of the current study and update the existing evidence, a systematic literature search was conducted in PubMed, Web of Science, EMBASE, and the Cochrane Library to identify previous systematic reviews with meta-analyses published by 6 May 2023 that reported on the associations of FA biomarkers and CVD outcomes (see Supplementary material online, Supplementary Table*S2*). Study selection and data extraction were conducted by two reviewers (FS and LS) independently, and discussed with the third reviewer (SK). Articles were excluded if the study population were non-adult, pregnant, critically ill, or restricted to those with any pre-existing disease (e.g. patients with established CVD at baseline). Each primary study included in the eligible meta-analyses was reviewed and details of estimates for any FA-outcome association reported by the primary study were extracted. The extracted estimates were standardized to correspond to relative risks (RR) per 1 SD higher FA using established methods (see Supplementary Methods). Inclusion criteria required that the corresponding primary study was prospective in study design (i.e. nested case-control, case-cohort, or prospective cohort and RCTs), selected participants from general populations, assessed FAs in relative concentrations, and investigated the associations of FAs with CHD or stroke. When one primary study reported several estimates with different degrees of adjustments for the same FA-outcome association, the one obtained from the publication with the largest number of cases or the most adjusted one that did not include the adjustment for lipids or circulating FA biomarkers was selected, as circulating lipids may act as potential mediators between FA and CVD risks.^22^ For primary studies reporting estimates of FA biomarkers in more than one biological tissue, one association estimate was selected using the following hierarchy to preference lipid compartments, which was more consistent with our current study: plasma/serum phospholipids > total plasma or serum > erythrocyte > plasma/serum cholesterol esters > adipose tissue.

An updated meta-analysis was conducted in a pragmatic approach, by adding the estimates of the current study using the random-effects (DerSimonian-Laird) method due to expected heterogeneity between studies (e.g. differences in participant characteristics, adjustments, and assay compartments). Overall heterogeneity was expressed as *I*^2^ statistic,^23^ with values of 25%, 50%, and 75% indicating low, medium, and high heterogeneity, respectively. Sub-group analyses were conducted based on random-effects meta-regression to explore the potential heterogeneity from geographic regions and lipid compartments. Secondarily, systematic reviews with meta-analysis of RCTs of FA intake on CHD and stroke were also searched, and the one including most RCTs was selected for evidence summary when multiple articles were identified on the same topic. We used Stata, version 16 for all analyses and R version 4.2.2 for displaying some results as Circos plots.

## Results

### Baseline characteristics of the study participants

The combined analysis included 172 891 participants without prior vascular disease from EPIC-CVD, UKB, and INTERVAL (*Table 1*; Supplementary material online, Supplementary Table*S3*). There were 9453 CHD and 8182 stroke cases recorded during median follow-up durations of 9.4 to 12.6 years across studies. In EPIC-CVD, where FAs were measured in the plasma phospholipid fraction, SFA comprised 45.9% of total FAs with even-chain SFA being the greatest contributor; PUFA comprised 42.6% of total FAs, with *n*-6 PUFA (35.9%) being more abundant than *n*-3 PUFA (6.7%), of which LA (22.6%), arachidonic acid (AA, 9.3%), DHA (4.3%), dihomo-gamma-linolenic acid (DGLA, 3.1%) and eicosapentaenoic acid (EPA, 1.2%) had relatively high concentrations (i.e. all > 1%); whereas MUFA (11.0%) comprised a smaller proportion (see Supplementary material online, Table*S4*). In UKB and INTERVAL, measuring total plasma or serum FAs, PUFA (42.6% and 37.4%, respectively) comprised the greatest proportion followed by SFA (34.0% and 37.1%) and MUFA (23.4% and 25.6%).

**Table 1:** Characteristics of the study participants

Baseline characteristics	Overall^a^ (*n* = 172 891)	EPIC-CVD^a^ (*n* = 31 606)	UKB (*n* = 99 762)	INTERVAL (*n* = 41 523)
Location	Europe	Eight European countries	UK	UK
Women, *n* (%)	85 844 (54.6)	9922 (62.4)	55 044 (55.2)	20 878 (50.3)
Age (years), mean (SD)	52.5 (11.6)	52.2 (9.1)	56.4 (8.1)	43.2 (14.2)
BMI (kg/m^2^), mean (SD)^b^	26.9 (4.8)	26.1 (4.3)	27.3 (4.7)	26.5 (5.0)
Current smoking, *n* (%)^b^	18 152 (11.6)	4078 (25.9)	10 494 (10.5)	3581 (8.7)
History of hypertension, *n* (%)^b^	29 904 (26.0)	5637 (35.8)	24 267 (24.4)	NR
History of diabetes, *n* (%)^b^	4757 (4.2)	450 (3.1)	4307 (4.3)	NR
Lipid-lowering medication, *n* (%)^b^	13 162 (12.8)	373 (9.9)	12 789 (12.9)	NR
Main fatty acids (%)^c^				
Assay Compartment	—	Plasma phospholipid	Total serum/plasma	Total serum/plasma
Plasma sample, *n* (%)	131 413 (76.0)	31 606 (100.0)	99 762 (100.0)	45 (0.1)
Serum sample, *n* (%)	41 478 (24.0)	0 (0.0)	0 (0.0)	41 478 (99.9)
Total SFA	39.0 (1.9)	45.9 (1.2)	34.0 (1.9)	37.1 (1.9)
Even-chain SFA	44.6 (1.2)	44.6 (1.2)	NR	NR
Odd-chain SFA	0.6 (0.1)	0.6 (0.1)	NR	NR
Long and very long-chain SFA	0.7 (0.2)	0.7 (0.2)	NR	NR
Total MUFA	20.0 (2.8)	11.0 (1.9)	23.4 (2.6)	25.6 (3.4)
Total PUFA	40.9 (3.5)	42.6 (2.1)	42.6 (3.7)	37.4 (3.5)
*n*-3 PUFA	5.0 (1.5)	6.7 (1.9)	4.4 (1.5)	3.8 (0.9)
DHA	2.5 (0.7)	4.3 (1.2)	2.0 (0.7)	1.2 (0.4)
*n*-6 PUFA	35.9 (3.5)	35.9 (2.9)	38.2 (3.6)	33.6 (3.3)
LA	26.3 (3.3)	22.6 (3.2)	29.2 (3.4)	27.0 (3.2)
Incident cardiovascular outcomes				
Follow-up, years (5th–95th percentile)^d^	11.0 (5.1 to 13.3)	10.1 (1.2 to 15.1)	11.7 (8.0 to 13.1)	9.4 (8.4 to 10.0)
Total CVD	20 130	16 055	3534	541
CHD	9453	7343	1825	285
Stroke	8182	6499	1474	209
Ischaemic stroke	4935	3834	951	150
Haemorrhagic stroke	1603	1201	347	55
CVD mortality	3674	2573	977	124

BMI, body mass index; CHD, coronary heart disease; CVD, cardiovascular disease; DHA, docosahexaenoic acid; LA, linoleic acid; MUFA, monounsaturated fatty acid; NR, not reported; PUFA, polyunsaturated fatty acid; SD, standard deviation; SFA, saturated fatty acid.

^a^Baseline characteristics of EPIC-CVD were summarized based on sub-cohort participants (*n* = 15 889) given its case-cohort study design. The total number of EPIC-CVD participants (*n* = 31 606) was the sum of sub-cohort and cases of total CVD, CHD, stroke, and CVD mortality.

^b^There were missing data for BMI (0.4%), smoking (0.6%), history of diabetes (3.2%), and history of hypertension (0.5%). Information on lipid-lowering medication was available from 99.2% of UKB participants but only 29.7% of EPIC-CVD participants. Information on history of diabetes, history of hypertension, and lipid-lowering medication was not reported in INTERVAL and not counted in the overall, but they were considered as generally healthy given participants were all blood donors.

^c^The average concentrations of fatty acids (%) in the overall column were the pooled estimates of those from EPIC-CVD sub-cohort, UKB, and INTERVAL using random-effects meta-analysis.

^d^For EPIC-CVD, follow-up years were 10.1 (1.2 to 15.1) for total CVD, 11.9 (2.6 to 15.6) for CHD, 12.6 (2.8 to 16.2) for stroke and its sub-types, and 12.9 (4.1 to 16.2) for CVD mortality, respectively. For UKB, follow-up years were 11.8 (10.4 to 13.2) for CVD mortality. For INTERVAL, follow-up years were 9.7 (8.7 to 10.2) for CVD mortality.

### Fatty acids and dietary intake

**Figure 1:**
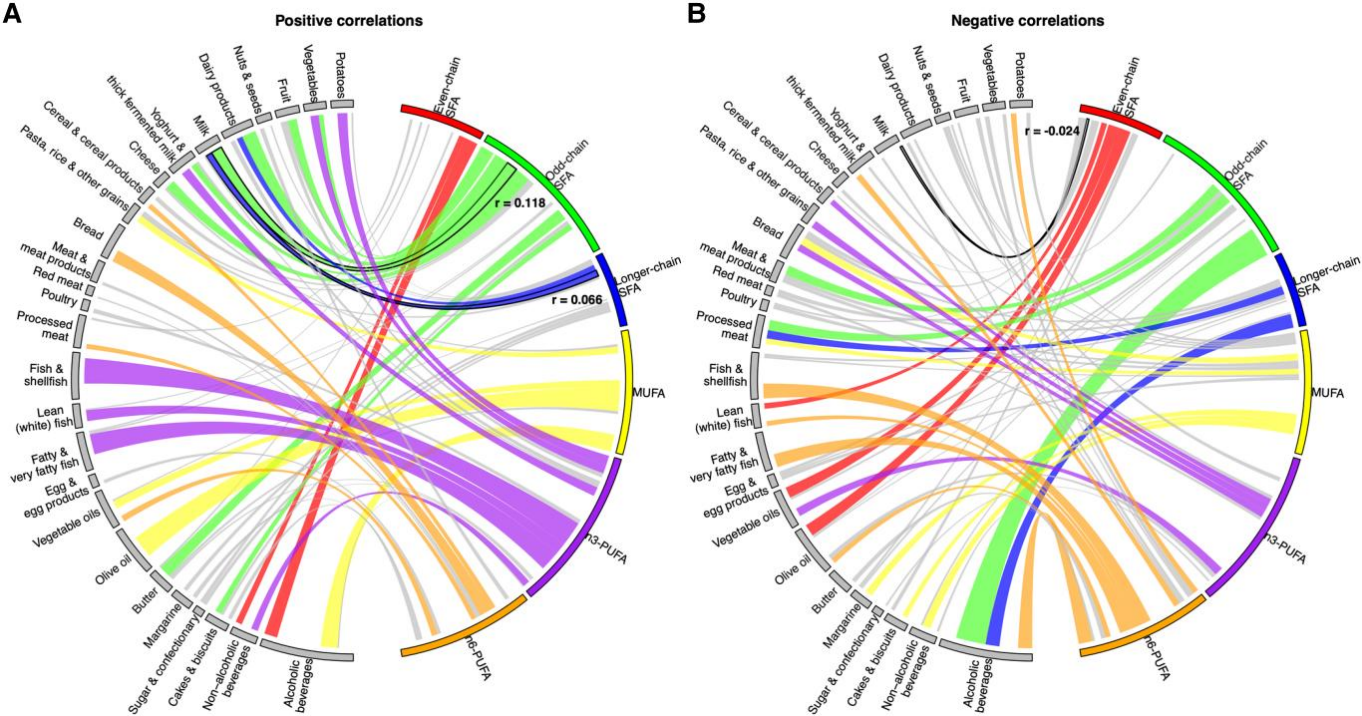
Circos plot of correlations between major fatty acid sub-types (%) and self-reported food intake (g/day) in EPIC-CVD study, separated into positive (left side) and negative (right side) associations. MUFA, monounsaturated fatty acid; PUFA, polyunsaturated fatty acid; SFA, saturated fatty acid. Analyses were conducted restricted to the sub-cohort of EPIC-CVD study (*n* = 15 838). Width of curves indicates the strength of statistically significant correlations with *P*-value <0.05 (i.e. semipartial correlation coefficients adjusted for batch, sex, age, and total energy intake), and correlations with the absolute value of coefficient <0.05 were coloured in grey. For example, correlations of milk intake with even-chain, odd-chain, and longer-chain SFAs were −0.024, 0.118, and 0.066, respectively (highlighted by black borders in the figure). Further detailed correlations between individual plasma phospholipid fatty acids and food intake were illustrated in Supplementary material online, Supplementary Figure*S3*.

There were broadly distinct patterns of associations of dietary intakes within plasma phospholipid FA groups, though with modest or weak correlations (*Figure 1*; Supplementary material online, Supplementary Figure*S3*). Odd-chain SFA was positively associated with dairy products (*r* = 0.15), fruit (*r* = 0.10), and vegetables (*r* = 0.05) intake and negatively associated with alcoholic beverage (*r* = −0.32) and processed meat (*r* = −0.11) intakes, which were relatively opposite to those observed for even-chain SFA. Patterns of dietary intake correlations with longer-chain SFA were similar to, but weaker than, those with odd-chain SFA. Positive correlations with fish intake were most evident for *n*-3 PUFA (*r* = 0.27), particularly for EPA (*r* = 0.18) and DHA (*r* = 0.32), whereas *n*-6 PUFA was associated with higher intakes of bread, processed meat, cereal, and cereal products (0.05 < *r* < 0.13). MUFA was particularly associated with higher olive oil and alcoholic beverage intake (*r* > 0.18) and trans-FA was especially associated with higher intakes of margarine, potatoes, and dairy products (*r* > 0.15). Correlations between measured plasma phospholipid FAs and estimated dietary FAs intake were moderate: SFA (*r* = 0.11), MUFA (*r* = 0.27), and PUFA (*r* = 0.28).

### Saturated fatty acids biomarkers and risk of cardiovascular outcomes

**Figure 2:**
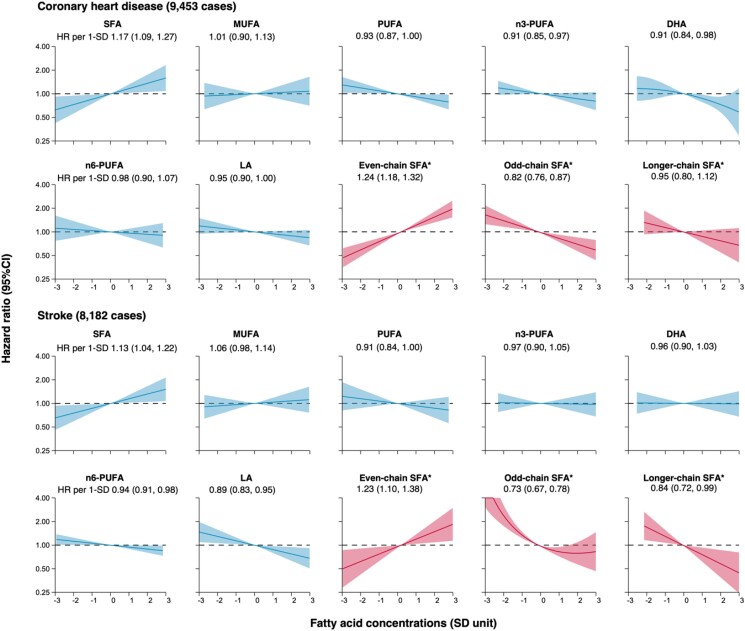
Dose-response curve and hazard ratios for associations of fatty acids with coronary heart disease and stroke, estimated in 172 891 participants from EPIC-CVD, UKB, and INTERVAL studies. CI, confidence interval; DHA, docosahexaenoic acid; HR, hazard ratio; LA, linoleic acid; MUFA, monounsaturated fatty acid; PUFA, polyunsaturated fatty acid; SD, standard deviation; SFA, saturated fatty acid. Results are expressed as HRs according to fatty acid concentrations in SD units using random effects meta-analysis, adjusted for batch (EPIC-CVD only), age, smoking status, history of diabetes, history of hypertension, and physical activity, and stratified by centre (EPIC-CVD only) and sex. The shaded area represents the 95% CI for the dose-response curve. The final estimates are pooled HRs (95% CI) per 1-SD higher fatty acid concentrations with the same adjustment using random-effects meta-analysis. *Restricted to EPIC-CVD (7343 CHD cases; 6499 stroke cases) as concentrations of SFA sub-types were only available from EPIC-CVD (as shown in red curves). Detailed information of estimates for different cardiovascular outcomes in Supplementary material online, *Tables S5* and *S6*.

Associations of relative concentrations of SFA and its sub-types with cardiovascular risks were mostly log-linear adjusted for non-dietary lifestyle factors (*Figure 2*; Supplementary material online, Supplementary Table*S5*). HRs per 1-SD higher SFA were 1.17 (1.09–1.27) for CHD and 1.13 (1.04–1.22) for stroke, with differential associations found for SFA sub-types: even-chain SFA was positively associated with CHD [HR 1.24 (1.18–1.32)] and stroke [1.23 (1.10–1.38)], whereas odd-chain SFA was negatively associated with CHD [0.82 (0.76–0.87)] and stroke [0.73 (0.67–0.78)]. There was no statistically significant association of longer-chain SFA with CHD [0.95 (0.80–1.12)], whereas an inverse association was found for stroke [0.84 (0.72–0.99)]. These associations remained broadly similar after further adjustment for alcohol consumption, BMI, and dietary intakes in smaller sub-sets of data with complete relevant information (*Tables 2* and *3*; Supplementary material online, Supplementary Tables*S6* and S7). Significant associations remained for even-chain and odd-chain SFA with additional adjustments for lipids or mutual adjustment for each other (*Table 3*).

**Table 2:** Hazard ratios per 1-SD higher fatty acids for CHD and stroke, with further adjustments for potential confounders or mediators in EPIC-CVD, UKB, and INTERVAL studies

Fatty acids (%) \ adjustments^a^	CHD (8424 cases)		Stroke (6380 cases)	
	HR (95% CI)	*P*	HR (95% CI)	*P*
Total SFA				
Adjusted for age, sex, and lifestyle factors	1.18 (1.09, 1.28)	<0.001	1.16 (1.07, 1.26)	<0.001
Plus alcohol consumption and BMI	1.14 (1.06, 1.24)	0.001	1.11 (1.04, 1.18)	0.001
Plus lipid markers	1.07 (1.02, 1.13)	0.012	1.11 (1.06, 1.16)	<0.001
Total MUFA				
Adjusted for age, sex, and lifestyle factors	1.03 (0.92, 1.14)	0.635	1.06 (0.98, 1.15)	0.164
Plus alcohol consumption and BMI	1.04 (0.94, 1.14)	0.458	1.06 (0.98, 1.15)	0.145
Plus lipid markers	1.03 (0.92, 1.16)	0.578	1.08 (0.97, 1.20)	0.161
Total PUFA				
Adjusted for age, sex, and lifestyle factors	0.91 (0.86, 0.97)	0.002	0.90 (0.82, 0.98)	0.013
Plus alcohol consumption and BMI	0.91 (0.87, 0.96)	<0.001	0.91 (0.83, 0.99)	0.028
Plus lipid markers	0.94 (0.87, 1.02)	0.156	0.89 (0.80, 1.00)	0.051
*n*-3 PUFA				
Adjusted for age, sex, and lifestyle factors	0.90 (0.84, 0.97)	0.004	0.98 (0.89, 1.09)	0.727
Plus alcohol consumption and BMI	0.89 (0.84, 0.94)	<0.001	0.99 (0.90, 1.08)	0.758
Plus lipid markers	0.89 (0.85, 0.94)	<0.001	1.00 (0.91, 1.10)	0.976
DHA				
Adjusted for age, sex, and lifestyle factors	0.90 (0.83, 0.98)	0.011	0.98 (0.89, 1.08)	0.719
Plus alcohol consumption and BMI	0.89 (0.83, 0.95)	0.001	0.99 (0.91, 1.08)	0.812
Plus lipid markers	0.88 (0.84, 0.93)	<0.001	1.00 (0.90, 1.10)	0.956
*n*-6 PUFA				
Adjusted for age, sex, and lifestyle factors	0.97 (0.89, 1.05)	0.482	0.93 (0.89, 0.97)	<0.001
Plus alcohol consumption and BMI	0.98 (0.90, 1.06)	0.637	0.95 (0.91, 0.99)	0.008
Plus lipid markers	1.02 (0.94, 1.11)	0.567	0.88 (0.83, 0.93)	<0.001
LA				
Adjusted for age, sex, and lifestyle factors	0.95 (0.90, 1.00)	0.033	0.86 (0.79, 0.93)	<0.001
Plus alcohol consumption and BMI	0.98 (0.93, 1.03)	0.426	0.89 (0.83, 0.95)	0.001
Plus lipid markers	0.99 (0.94, 1.04)	0.625	0.89 (0.85, 0.93)	<0.001

BMI, body mass index; CHD, coronary heart disease; CI, confidence interval; DHA, docosahexaenoic acid; HR, hazard ratio; LA, linoleic acid; MUFA, monounsaturated fatty acid; PUFA, polyunsaturated fatty acid; SD, standard deviation; SFA, saturated fatty acid.

Results are pooled HRs (95% CI) per 1-SD higher fatty acid concentrations using random-effects meta-analysis, estimated on a complete-case basis (*n* = 167 620) due to missing data of potential confounders or mediators.

^a^Adjustments: (i) the basic adjustment for age, sex and lifestyle factors consists of adjustment for batch (EPIC-CVD only), age, smoking, history of diabetes, history of hypertension, and physical activity, stratified by centre (EPIC-CVD only) and sex; (ii) plus alcohol consumption and BMI; and (iii) plus lipid markers (i.e. total cholesterol, high-density-lipoprotein cholesterol, loge triglycerides).

**Table 3:** Hazard ratios per 1-SD higher saturated fatty acid sub-types for CHD and stroke, with progressive adjustments for potential confounders or mediators in EPIC-CVD study

	Even-chain SFA		Odd-chain SFA		Longer-chain SFA	
	HR (95% CI) per 1-SD higher		HR (95% CI) per 1-SD higher		HR (95% CI) per 1-SD higher	
	CHD (*n* = 6341)	Stroke (*n* = 4719)	CHD (*n* = 6341)	Stroke (*n* = 4719)	CHD (*n* = 6341)	Stroke (*n* = 4719)
Basic adjustment for age, sex, and lifestyle factors^a^						
Adjusted for batch and age	1.31 (1.22, 1.41)	1.31 (1.15, 1.51)	0.75 (0.70, 0.82)	0.69 (0.63, 0.76)	0.87 (0.76, 0.99)	0.80 (0.61, 1.04)
Plus smoking status	1.30 (1.21, 1.39)	1.31 (1.14, 1.50)	0.78 (0.73, 0.84)	0.72 (0.65, 0.78)	0.88 (0.76, 1.02)	0.80 (0.63, 1.02)
Plus history of diabetes	1.31 (1.21, 1.42)	1.33 (1.19, 1.48)	0.78 (0.72, 0.84)	0.68 (0.63, 0.74)	0.88 (0.75, 1.04)	0.79 (0.65, 0.95)
Plus history of hypertension	1.27 (1.19, 1.36)	1.28 (1.15, 1.42)	0.81 (0.75, 0.88)	0.73 (0.67, 0.79)	0.95 (0.80, 1.12)	0.83 (0.71, 0.96)
Plus physical activity	1.26 (1.19, 1.32)	1.28 (1.16, 1.42)	0.81 (0.75, 0.88)	0.73 (0.67, 0.79)	0.96 (0.81, 1.14)	0.83 (0.71, 0.97)
Plus alcohol consumption and BMI						
Plus alcohol consumption	1.27 (1.20, 1.35)	1.29 (1.16, 1.43)	0.79 (0.74, 0.84)	0.73 (0.67, 0.79)	0.94 (0.80, 1.12)	0.82 (0.70, 0.97)
Plus BMI	1.23 (1.16, 1.29)	1.22 (1.11, 1.34)	0.81 (0.77, 0.86)	0.75 (0.69, 0.81)	0.95 (0.81, 1.11)	0.84 (0.72, 0.98)
Plus dietary intakes (g/day)						
Plus fruit and vegetables	1.23 (1.16, 1.30)	1.22 (1.11, 1.34)	0.82 (0.77, 0.87)	0.75 (0.69, 0.82)	0.95 (0.81, 1.11)	0.84 (0.72, 0.98)
Plus dairy products	1.23 (1.16, 1.30)	1.23 (1.12, 1.35)	0.82 (0.76, 0.87)	0.76 (0.70, 0.82)	0.94 (0.81, 1.10)	0.84 (0.73, 0.98)
Plus meat and meat products	1.23 (1.16, 1.30)	1.23 (1.12, 1.35)	0.82 (0.77, 0.87)	0.76 (0.70, 0.82)	0.94 (0.81, 1.09)	0.85 (0.73, 0.98)
Plus fish and shellfish	1.24 (1.17, 1.31)	1.23 (1.12, 1.35)	0.82 (0.77, 0.88)	0.76 (0.70, 0.82)	0.94 (0.81, 1.09)	0.85 (0.73, 0.98)
Plus olive oil	1.25 (1.17, 1.34)	1.24 (1.13, 1.36)	0.82 (0.77, 0.88)	0.76 (0.70, 0.82)	0.94 (0.81, 1.09)	0.84 (0.72, 0.98)
Plus margarine	1.24 (1.17, 1.32)	1.24 (1.15, 1.34)	0.82 (0.77, 0.88)	0.76 (0.70, 0.83)	0.95 (0.82, 1.11)	0.84 (0.72, 0.98)
Plus alcoholic beverages	1.27 (1.18, 1.36)	1.23 (1.14, 1.33)	0.79 (0.72, 0.87)	0.76 (0.70, 0.83)	0.95 (0.79, 1.13)	0.85 (0.73, 0.98)
Plus lipid markers						
Plus TC	1.24 (1.16, 1.34)	1.22 (1.14, 1.31)	0.82 (0.75, 0.91)	0.76 (0.70, 0.83)	0.93 (0.77, 1.12)	0.84 (0.72, 0.98)
Plus HDL-C	1.17 (1.10, 1.25)	1.19 (1.11, 1.28)	0.82 (0.74, 0.90)	0.76 (0.69, 0.83)	0.92 (0.79, 1.08)	0.85 (0.73, 0.98)
Plus log triglycerides	1.17 (1.10, 1.24)	1.18 (1.10, 1.27)	0.83 (0.75, 0.92)	0.77 (0.71, 0.84)	0.94 (0.83, 1.06)	0.89 (0.78, 1.02)
Plus mutual adjustment of each other ^b^						
Plus other SFA sub-types	1.21 (1.12, 1.30)	1.18 (1.09, 1.27)	0.82 (0.76, 0.89)	0.81 (0.74, 0.88)	1.02 (0.88, 1.18)	0.93 (0.84, 1.03)

BMI, body mass index; CHD, coronary heart disease; CI, confidence interval; HDL-C, high-density-lipoprotein cholesterol; HR, hazard ratio; M, model; SD, standard deviation; SFA, saturated fatty acid; TC, total cholesterol.

SFA sub-types included even-chain (sum of 14:0, 16:0, and 18:0), odd-chain (sum of 15:0 and 17:0) and longer-chain (sum of 20:0, 22:0, 23:0, and 24:0) SFA. Results are pooled HRs (95% CI) per 1-SD higher fatty acid concentrations using random-effects meta-analysis, estimated on a complete-case basis within EPIC-CVD (sub-cohort *n* = 15 125) due to missing data of potential confounders or mediators.

^a^The basic adjustment for age, sex, and lifestyle factors consists of adjustment for batch, age, smoking status, history of diabetes, history of hypertension, and physical activity, stratified by centre and sex.

^b^Mutual adjustment for even-chain, odd-chain, and longer-chain SFAs to assess the independence of each other, based on the model adjusted for batch, age, sex (stratified), smoking, history of diabetes, history of hypertension, physical activity, BMI, and dietary intakes.

### Unsaturated fatty acids biomarkers and risk of cardiovascular outcomes

Higher relative concentrations of total *n*-3 PUFA were negatively associated with CHD [0.91 (0.85–0.97)] but were not associated with stroke [0.97 (0.90–1.05)] or other cardiovascular outcomes, which remained similar with further adjustment for alcohol consumption, BMI, and lipids or total *n*-6 PUFA (*Figure 2*; Supplementary material online, Supplementary Tables*S5* and S6). Similar results were observed for DHA [CHD 0.91 (0.84–0.98); stroke 0.96 (0.90–1.03)], the greatest contributor of *n*-3 PUFA. No consistent significant associations were found for total long-chain *n*-3 PUFA, omega-3 index, or other contributors of *n*-3 PUFA individually (i.e. alpha-linolenic acid [ALA], EPA, and docosapentaenoic acid [n3-DPA]) (see Supplementary material online, Supplementary Table*S9*).

Higher relative concentrations of total *n*-6 PUFA were log-linearly associated with lower stroke risk, with a HR per 1-SD of 0.94 (0.91–0.98), which remained significant after additional adjustment for dietary factors, lipids, or total *n*-3 PUFA (*Figure 2*; Supplementary material online, Supplementary Tables*S5* and S6). Total *n*-6 PUFA was not associated with CHD [0.98 (0.90–1.07)] with basic adjustment, but showed a negative association when further adjusted for total *n*-3 PUFA [0.91 (0.88–0.95)]. Similar associations were observed for LA [CHD 0.95 (0.90–1.00); stroke 0.89 (0.83–0.95)], the dominant component of *n*-6 PUFA. Among other individual *n*-6 PUFAs, there were strong positive associations of DGLA with CHD [1.41 (1.29–1.53)] and stroke [1.22 (1.12–1.34)], which remained after maximal adjustment. Docosatetraenoic acid (DTA), docosapentenoic acid (*n*-6 DPA), and gamma linolenic acid (GLA) were positively associated with CHD whereas AA was positively associated with stroke, but associations were no longer statistically significant after adjustment for dietary intakes or lipids (see Supplementary material online, Supplementary Table*S9*). Among ratio variables, DGLA to LA ratio was associated with higher CHD and stroke risks while AA to DGLA ratio was associated with lower CHD risk (see Supplementary material online, Supplementary Table*S11*).

No significant associations of MUFA were observed for the main cardiovascular outcomes (*Figure 2*). MUFA was only positively associated with CVD mortality risk (see Supplementary material online, Supplementary Tables*S5*–S6), but analysis of individual components found higher concentrations of palmitoleic acid (16:1) to be associated with higher risks of stroke (see Supplementary material online, Supplementary Table*S8*). Stearoyl-CoA-desaturase (SCD)-16 (i.e. ratio of 16:1 to 16:0) was also positively associated with stroke risk even after maximal adjustments (see Supplementary material online, Supplementary Table*S11*). Significant negative associations of total trans FAs (particularly for trans-18:1) were observed for CHD, which persisted after further adjustments (see Supplementary material online, Supplementary Table*S10*).

Findings were similar in sensitivity analyses, including after excluding the initial two years of follow-up (see Supplementary material online, Supplementary eTable*S5*), with further adjustment for CRP and HbA1C or glucose (see Supplementary material online, Supplementary eTable*S12*), and in sub-group analyses assessing possible modification by sex, age, smoking, physical activity, alcohol consumption, BMI, TC, or HDL-C (see Supplementary material online, Supplementary eFigures*S4 and*S5). The positive association of total SFA with CHD was attenuated among individuals with history of diabetes whereas the inverse association of PUFA with CHD was attenuated among those with prior hypertension; meanwhile, the inverse association of LA with CHD was attenuated with higher TG concentrations (all *P* for interaction < 2 × 10^−4^). No substantial differences were found in the magnitude of associations obtained for relative vs. absolute concentrations, except that associations of total PUFA and *n*-6 PUFA with CHD were discordant when adjusted for non-lipid risk factors and attenuated towards null when further adjusted for lipids (see Supplementary material online, Supplementary Table*S13*). The RDRs for FAs calculated using UKB data ranged from 0.44 to 0.67 and HRs corrected for regression dilution bias were about 50% to two-fold stronger (see Supplementary material online, Supplementary Table*S14*). Results from competing risks-adjusted analyses were broadly similar to those of primary analyses (see Supplementary material online, Supplementary Table*S15*) including results for ischaemic stroke outcome being broadly similar to those presented for overall stroke.

### Updated meta-analysis with existing evidence

**Figure 3:**
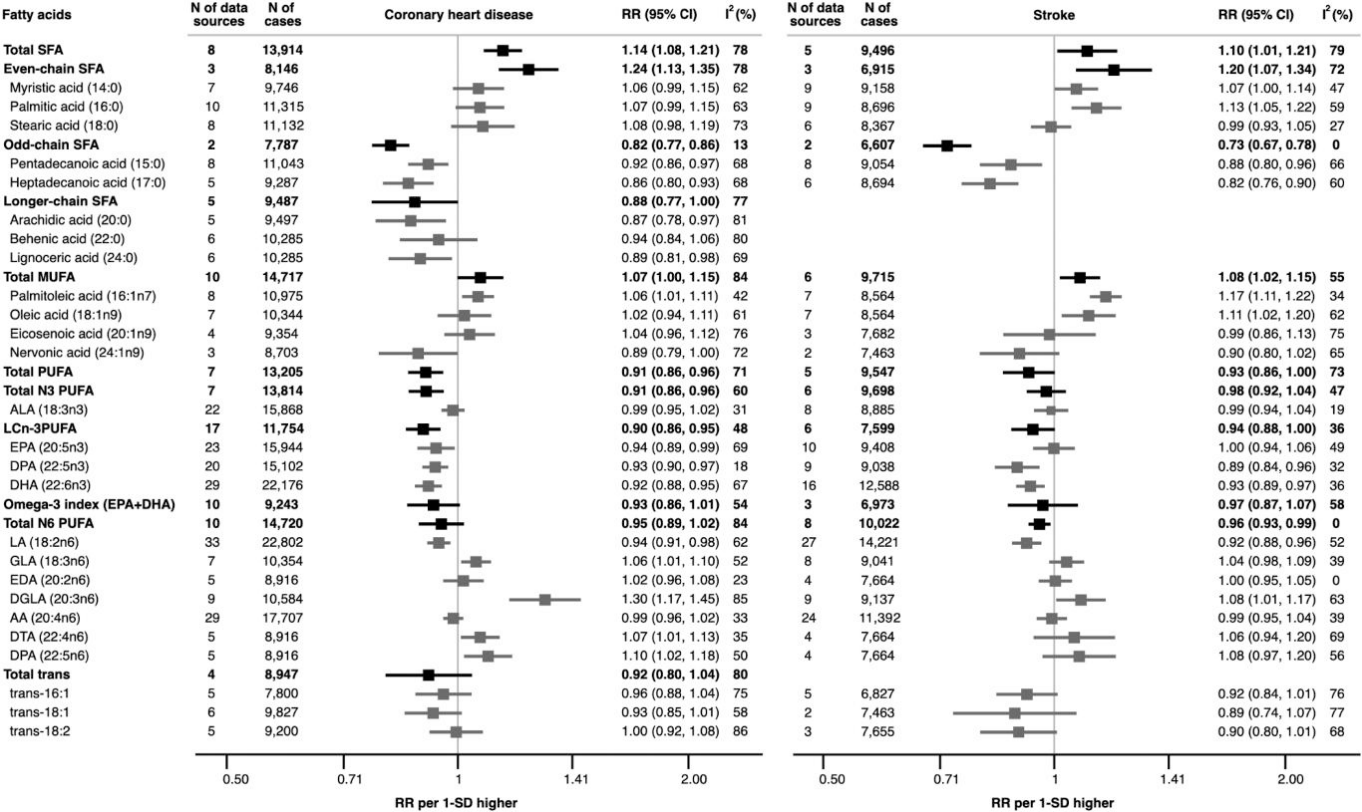
Updated meta-analysis combining results from EPIC-CVD, UKB, and INTERVAL studies with published evidence for associations of fatty acid biomarkers with coronary heart disease and stroke, separately. RR, relative risk; SD, standard deviation. For the abbreviations of fatty acids, please refer to the Supplementary material online, *Table S4*. The results of updated meta-analyses using random-effects method were summarized for the fatty acids, of which associations with coronary heart disease or stroke were identified from existing published evidence. Results for ischaemic stroke in Supplementary material online, *Figure S7*.

Supplementary material online, Supplementary Figure *S6* shows the flow diagram for the systematic literature search process and Supplementary material online, Tables*S16*–S20 summarize detailed study characteristics of the 49 identified primary studies identified as reporting non-duplicated associations of FA biomarkers with CHD and stroke. Depending on the FA investigation, the totality of previous evidence has involved sample sizes of between 444 and 13 349 CHD cases and between 108 and 6039 stroke cases from non-duplicate studies. The updated meta-analyses involved between 7787 and 22 802 CHD and between 6499 and 14 221 stroke cases and demonstrated broadly similar pooled results as observed in our primary study analyses (*Figure 3*; Supplementary material online, Supplementary Figure*S7*), with a few exceptions, including: significant evidence for negative association of DHA, *n*-3 DPA, and LA with both CHD and stroke; null evidence for association of trans-FAs with CHD and stroke; and significant negative association of EPA and total long-chain *n*-3 PUFA with CHD in the meta-analysis (*Figure 3*). Alpha-linolenic acid and AA were not associated with CHD and stroke risk, and findings were consistent across geographical regions and lipid compartments (see Supplementary material online, Supplementary Figures*S8*–S11). However, associations of total MUFA with CHD risk somewhat differed across various lipid compartments (*P* < 0.001) and associations of individual odd-chain SFAs with both CHD and stroke suggestively varied by geographic region (0.001 < *P* < 0.021).

Results of the supplementary literature review conducted to summarize evidence from meta-analysis of RCTs of FA and risk of CHD and stroke are presented in Supplementary material online, Supplementary Table*S21*. Consistent with our observational findings, combined RCT evidence including 32 trials suggested that long-chain omega-3 fat intake may slightly reduce the CHD risk, but there were no apparent effects on stroke risk. The synthesized evidence from RCTs of saturated and omega-6 fat intake was rated of poor quality and unclear. Furthermore, there was little RCT evidence for monotherapy targeted at one FA supplementation (e.g. DHA, n3-DPA, LA, or odd-chain SFA).

## Discussion

There is an unmet need of conclusive evidence on the relevance of fatty acids for primary prevention of CVD. Current CVD dietary guidelines remain controversial in light of mixed findings from existing RCTs of targeted FA supplementation (mainly *n*-3 and *n*-6 PUFAs and SFAs) and observational studies of circulating FAs. Our combined analysis of 172 891 participants with 9453 CHD and 8182 stroke cases, as well as an updated meta-analysis with evidence from 49 non-overlapping prior studies, provides the largest and most extensive investigation to date on associations between circulating FAs and incident major cardiovascular outcomes in generally healthy populations. Differential associations were observed for SFA sub-types, in that even-chain SFA had positive associations with cardiovascular risk whereas odd-chain and longer-chain SFA had negative associations. Higher total *n*-3 PUFA was associated with lower CHD risk while higher total *n*-6 PUFA was associated with lower stroke risk. Among individual PUFAs, LA (the predominant *n*-6 PUFA), DHA, and n3-DPA were each negatively associated with risks of CHD and stroke, while DGLA was positively associated with CHD and stroke. ALA, an *n*-3 PUFA mainly derived from plant sources, was not associated with lower CHD or stroke risk; and AA, a key metabolite of LA, was not associated with higher CHD or stroke risk.

The positive correlations between dairy product intake and odd-chain SFA concentrations are consistent with previous evidence that odd-chain SFAs (especially 15:0) are considered biomarkers of dairy intake,^24^ a type of food source possibly associated with lower cardiovascular risk.^25^ Our results of negative associations of odd-chain SFAs (15:0 and 17:0), either individually or in combination, with risks of CHD and stroke also suggested the potential cardiovascular benefits of dairy consumption. The distinction between fat-rich food and FAs (e.g. dairy intake vs. odd-chain SFA) is however important, as the former also includes other nutrients that may be either beneficial or harmful for CVD.^8^ Meanwhile, odd-chain SFAs are only a minor group of FAs in dairy, which mainly consists of even-chain SFA and MUFA. The previous assumption that odd-chain SFAs are totally derived from dairy intakes originating from rumen microbial fermentation has been eroding slowly, and emerging evidence suggests that odd-chain SFA can be synthesized endogenously from elongation of short-chain FAs associated with dietary fiber^26^ or α-oxidation of even-chain SFA.^27^ Nevertheless, the strong negative evidence of odd-chain SFAs remained after adjusting for related dietary food intake, lipids, and other SFA sub-types, further supporting its potential beneficial role. However, it is important to highlight that the beneficial observations of odd-chain SFA might depend on the lipid compartment, as suggested by EPIC-Potsdam study on diabetes.^28^ We also observed null associations of odd-chain SFAs in the sub-groups other than plasma phospholipids (e.g. total plasma/serum or red blood cells), despite the non-significant differences with small numbers of studies included for comparison. More clinical and experimental research is required to investigate mechanisms whereby odd-chain SFAs may influence CVD. Longer-chain SFAs were weakly inversely associated with cardiovascular risks, and may be partly related to links to a potentially favourable profile of blood lipids, insulin resistance, and inflammatory markers^4^ though underlying mechanisms are poorly understood. Our study findings involving up to 11 043 CHD and 9054 stroke cases provide the strongest evidence so far for differential associations of SFA sub-types and challenge broad-based dietary recommendations of lowering total saturated fat intake.^5^

Recent meta-analyses of RCTs suggested around 9% lower risk of CHD with long-chain *n*-3 supplementation but little benefits on stroke.^2,29^ While majority of the RCTs had investigated EPA and DHA combined, a few RCTs of EPA monotherapy seemed to show more prominent effects than EPA + DHA.^29^ This may be related to the distinct biological properties not shared by EPA and DHA.^30^ Several studies indicated comparable or even greater efficacy of DHA in reducing TGs and individual pro-inflammatory cytokines than EPA^31,32^; however, DHA was suggested to be associated with increases in LDL-C levels whereas EPA had a minimal or neutral effect on LDL-C.^33^ EPA and DHA also have their own roles in maintaining membrane structure, but when EPA and DHA are combined, the resulting effects would be attenuated compared with their separate actions,^34^ which may partly explain the differences between EPA and EPA + DHA in clinical trials. Of interest, in our study, DHA was similarly associated with lower risks of both CHD and stroke whereas EPA only exhibited significant inverse associations with CHD. This is somewhat consistent with the overall null RCT evidence on stroke, given no similar clinical trials of DHA monotherapy have been conducted. Future studies are also warranted to investigate any mechanistic differences for EPA vs. DHA vs. EPA + DHA across different vascular domains. Moreover, background fish intake in RCTs should be highlighted as EPA and DHA concentrations are strongly influenced by dietary seafood intake.^35^ In contrast, n3-DPA is present in fish at lower concentrations, and circulating n3-DPA appears to be mainly derived from endogenous elongation of EPA.^35^ N3-DPA may have equivalent or greater efficacy than EPA or DHA for lowering TG and cholesterol and inhibiting platelet aggregation,^36^ but research remains scarce. Our observations suggest the potential beneficial role of n3-DPA on both CHD and stroke, and more research is needed to clarify its effects on cardiovascular health. The effects of ALA, the most common essential *n*-3 PUFA derived mainly from plant sources, on cardiovascular risk are also of particular interest, but evidence remained inconsistent and limited.^2^ Our study incorporating 15 868 CHD and 8885 stroke cases (nearly twice and four-fold as prior work, separately) recorded null evidence of objectively measured ALA in general populations, with consistent findings across lipid compartments.

Our findings provide convincing evidence for differential associations of individual *n*-6 PUFAs (i.e. LA, GLA, DGLA, DTA, and n6-DPA) with CHD or stroke risks, which may partly explain the conflicting results from RCTs of *n*-6 PUFA supplementation.^3^ Some evidence has suggested potential cardiometabolic benefits of dietary *n*-6 PUFA, including favourable associations with lipids, inflammation, insulin resistance, blood pressure, and body composition^37–39^. LA, the most abundant PUFA, is an essential FA that cannot be synthesized by humans. Our inverse finding of circulating LA and stroke risk is consistent with the above beneficial biological effects,^37–39^ as well as recent evidence where LA concentrations were expressed as either relative^11^ or absolute ones.^12^ Despite previously reported inverse association of self-reported LA intake with CHD,^40^ we did not find a robust significant association of circulating LA concentration with CHD in our primary study analysis, a finding that was consistent with previous largest study based on 30 prospective cohorts (11 857 CHD cases).^11^ Our findings with updated meta-analysis evaluating objectively measured LA in 22 802 CHD and 14 221 stroke cases considerably extend these previous results and add strong support for the cardiovascular benefits of LA. However, the intermediate metabolic products (i.e. GLA and DGLA), the product to substrate ratio of DGLA to LA, and the further downstream products (i.e. DTA and n6-DPA) were positively associated in particular with CHD risk. This highlights the important roles of *in vivo* metabolism which has been established for diabetes,^10^ though such endogenous conversion from LA only occurs at a rather low rate.^41^ We further supported that circulating levels of AA are not harmful to cardiometabolic health, in line with recent seminal findings on diabetes.^42^

Substantial differences in MUFA proportions between lipid compartments were noted in our results, together with their inconsistent associations with CHD risk. Although comparisons of FA fractions were not conducted within the same study population, similar proportions within corresponding lipid compartments were observed in other studies.^43,44^ Despite the potential beneficial effects of MUFA intake from plant sources on CHD,^45^ we observed positive associations of circulating MUFAs with cardiovascular risk, especially palmitoleic acid and SCD-16. Circulating MUFA seems unlikely to well reflect the dietary MUFA intake but may be affected by both SFA intake and desaturase activity, since much of MUFA is derived from SFA by SCD.^46^ In contrast, circulating trans FAs are more likely to be affected by dietary intake, with trans-18:1 being derived largely from industrially produced foods associated with adverse cardiovascular health.^47^ The seemingly opposite findings for trans-FA in our analysis of EPIC-CVD may not be surprising given the effective ban of industrially produced trans-FAs in the early 2000s.^47^

Our investigation has important strengths. The prospective study design, large sample sizes, long-term follow-up, adjustment for several potential confounders, a series of sensitivity analyses, and updated meta-analyses, allowed us to report robust findings. The use of objectively measured FAs prevented recall biases and allowed detailed evaluation of FA sub-types, thus leading to reliable findings that may not be disentangled by dietary intake studies. Furthermore, we provided reliable evidence for stroke, which has been less frequently studied than CHD. Different associations with CHD and stroke were found for several FAs, suggesting future aetiological studies should be cautious about using composite events (e.g. total CVD) as means to increase study power.

Potential limitations of this study deserve careful consideration. First, the true aetiological associations of FAs may be far from observed estimates, given the within-person fluctuation of FAs.^21^ Though the variability of FAs assessed in the EPIC-Norfolk study^48^ was much larger than those examined in our analysis within UKB participants, batch effects may make a difference given intraclass correlation coefficients could be affected by measurement scale but RDRs not. Our exploratory analysis corrected for RDRs suggests that the potential aetiological estimates may be much stronger than observed when using one time point measurement. However, measurement error in covariates should also be considered. Second, our investigation mainly focused on FAs in relative concentrations, but this is a valid method more commonly used in epidemiological studies.^49^ In our supplementary analyses, the directions and magnitudes of associations of FAs in absolute vs. relative concentrations were comparable, with no substantial differences found after full adjustment. Although NMR allowed the quantification of FAs in absolute concentration, its low resolution hampered the identification of multiple individual FAs within SFA, MUFA, *n*-3 PUFA, and *n*-6 PUFA groups,^12^ thus making the GC method more appropriate for detailed investigation. Although FA analytical methods were not standardized across studies, statistical standardisation of FA concentrations within each study could minimize this concern. Despite extensive efforts to harmonize across studies, methodological heterogeneity may still exist. For some FAs, sub-group analysis by lipid compartments was also hampered given few studies. None of the included studies investigated the free FA compartment whereas recent evidence highlights the importance of free FAs.^50^ Finally, there may be measurement errors due to self-reported factors, as well as residual confounding attributable to unmeasured or imprecisely measured covariates.

In conclusion, the current study, involving ∼20 000 incident CHD and incident stroke cases and supplemented by an updated meta-analysis of all prior evidence, provides the strongest evidence yet on the associations between FA sub-types and cause-specific vascular outcomes. In particular, our findings reinforce that risk associations vary importantly across SFA sub-types, and therefore challenge the current broad dietary recommendations focused solely on lowering overall SFA intake—in essence dietary recommendations need consider up to date evidence on circulating FAs and findings of RCTs. Finally, we report a favourable role of long-chain *n*-3 PUFAs and LA on cardiovascular health—a finding generally consistent with the available trial evidence on *n*-3 PUFAs and CHD and remains to be further proven, at least supporting current recommendations on PUFAs. Our findings could inspire the design of future RCTs to help refine dietary guidelines.

## Supplementary Material

zwae315_Supplementary_Data

## Data Availability

This paper analysed data from three separate studies. EPIC-CVD data were provided by EPIC centres and details on how to access EPIC data and biospecimens are available at: https://epic.iarc.fr/access. Enquiries for access to INTERVAL study data should be addressed to the steering committee at helpdesk@intervalstudy.org.uk and information, including the data access policy, is available to review at http://www.donorhealth-btru.nihr.ac.uk/project/bioresource. Data from UK Biobank study is available to any bona fide scientific researcher on application at: https://www.ukbiobank.ac.uk/enable-your-research/apply-for-access.

## References

[1] Mozaffarian D , Benjamin EJ, Go AS, Arnett DK, Blaha MJ, Cushman M, et al Heart disease and stroke statistics—2015 update. Circulation 2015;131:e29–e322. 25520374 10.1161/CIR.0000000000000152

[2] Abdelhamid AS , Brown TJ, Brainard JS, Biswas P, Thorpe GC, Moore HJ, et al Omega-3 fatty acids for the primary and secondary prevention of cardiovascular disease. Cochrane Database Syst Rev 2020;3:CD003177. 32114706 10.1002/14651858.CD003177.pub5 PMC7049091

[3] Hooper L , Al-Khudairy L, Abdelhamid AS, Rees K, Brainard JS, Brown TJ, et al Omega-6 fats for the primary and secondary prevention of cardiovascular disease. Cochrane database Syst Rev 2018;7:CD011094. 30019765 10.1002/14651858.CD011094.pub3 PMC6513455

[4] Malik VS , Chiuve SE, Campos H, Rimm EB, Mozaffarian D, Hu FB, et al Circulating very-long-chain saturated fatty acids and incident coronary heart disease in US men and women. Circulation 2015;132:260–268. 26048094 10.1161/CIRCULATIONAHA.114.014911 PMC4519378

[5] Krauss RM , Kris-Etherton PM. Public health guidelines should recommend reducing saturated fat consumption as much as possible: debate consensus. Am J Clin Nutr 2020;112:25–26. 32491172 10.1093/ajcn/nqaa134

[6] Chowdhury R , Warnakula S, Kunutsor S, Crowe F, Ward HA, Johnson L, et al Association of dietary, circulating, and supplement fatty acids with coronary risk. Ann Intern Med 2014;160:398. 24723079 10.7326/M13-1788

[7] Sacks FM , Lichtenstein AH, Wu JHY, Appel LJ, Creager MA, Kris-Etherton PM, et al Dietary fats and cardiovascular disease: a presidential advisory from the American Heart Association. Circulation 2017;136:e1–e23. 28620111 10.1161/CIR.0000000000000510

[8] Steur M , Johnson L, Sharp SJ, Imamura F, Sluijs I, Key TJ, et al Dietary fatty acids, macronutrient substitutions, food sources and incidence of coronary heart disease: findings from the EPIC-CVD case-cohort study across nine European countries. J Am Heart Assoc 2021;10:e019814. 34796724 10.1161/JAHA.120.019814 PMC9075396

[9] Forouhi NG , Koulman A, Sharp SJ, Imamura F, Kröger J, Schulze MB, et al Differences in the prospective association between individual plasma phospholipid saturated fatty acids and incident type 2 diabetes: the EPIC-InterAct case-cohort study. Lancet Diabetes Endocrinol 2014;2:810–818. 25107467 10.1016/S2213-8587(14)70146-9 PMC4196248

[10] Forouhi NG , Imamura F, Sharp SJ, Koulman A, Schulze MB, Zheng J, et al Association of plasma phospholipid n-3 and n-6 polyunsaturated fatty acids with type 2 diabetes: the EPIC-InterAct case-cohort study. PLOS Med 2016;13:e1002094. 27434045 10.1371/journal.pmed.1002094 PMC4951144

[11] Marklund M , Wu JHY, Imamura F, Del Gobbo LC, Fretts A, de Goede J, et al Biomarkers of dietary Omega-6 fatty acids and incident cardiovascular disease and mortality. Circulation 2019;139:2422–2436. 30971107 10.1161/CIRCULATIONAHA.118.038908 PMC6582360

[12] Borges MC , Schmidt AF, Jefferis B, Wannamethee SG, Lawlor DA, Kivimaki M, et al Circulating fatty acids and risk of coronary heart disease and stroke: individual participant data meta-analysis in up to 16 126 participants. J Am Heart Assoc 2020;9:e013131. 32114887 10.1161/JAHA.119.013131 PMC7335585

[13] Danesh J , Saracci R, Berglund G, Feskens E, Overvad K, Panico S, et al EPIC-Heart: the cardiovascular component of a prospective study of nutritional, lifestyle and biological factors in 520,000 middle-aged participants from 10 European countries. Eur J Epidemiol 2007;22:129. 17295097 10.1007/s10654-006-9096-8

[14] Sudlow C , Gallacher J, Allen N, Beral V, Burton P, Danesh J, et al UK biobank: an open access resource for identifying the causes of a wide range of Complex diseases of middle and old age. PLOS Med 2015;12:e1001779. 25826379 10.1371/journal.pmed.1001779 PMC4380465

[15] Moore C , Sambrook J, Walker M, Tolkien Z, Kaptoge S, Allen D, et al The INTERVAL trial to determine whether intervals between blood donations can be safely and acceptably decreased to optimise blood supply: study protocol for a randomised controlled trial. Trials 2014;15:363. 25230735 10.1186/1745-6215-15-363 PMC4177700

[16] Wang L , Summerhill K, Rodriguez-Canas C, Mather I, Patel P, Eiden M, et al Development and validation of a robust automated analysis of plasma phospholipid fatty acids for metabolic phenotyping of large epidemiological studies. Genome Med 2013;5:39. 23618465 10.1186/gm443 PMC3706814

[17] Würtz P , Havulinna AS, Soininen P, Tynkkynen T, Prieto-Merino D, Tillin T, et al Metabolite profiling and cardiovascular event risk. Circulation 2015;131:774–785. 25573147 10.1161/CIRCULATIONAHA.114.013116 PMC4351161

[18] Buchanan CDC , Lust CAC, Burns JL, Hillyer LM, Martin SA, Wittert GA, et al Analysis of major fatty acids from matched plasma and serum samples reveals highly comparable absolute and relative levels. Prostaglandins Leukot Essent Fat Acids 2021;168:102268. 10.1016/j.plefa.2021.102268 33831721

[19] Thompson S , Kaptoge S, White I, Wood A, Perry P, Danesh J. Statistical methods for the time-to-event analysis of individual participant data from multiple epidemiological studies. Int J Epidemiol 2010;39:1345–1359. 20439481 10.1093/ije/dyq063 PMC2972437

[20] White IR , Kaptoge S, Royston P, Sauerbrei W. Meta-analysis of non-linear exposure-outcome relationships using individual participant data: a comparison of two methods. Stat Med 2019;38:326–338. 30284314 10.1002/sim.7974 PMC6492097

[21] Wood A . Regression dilution methods for meta-analysis: assessing long-term variability in plasma fibrinogen among 27 247 adults in 15 prospective studies. Int J Epidemiol 2006;35:1570–1578. 17148467 10.1093/ije/dyl233

[22] Clarke R , Frost C, Collins R, Appleby P, Peto R. Dietary lipids and blood cholesterol: quantitative meta-analysis of metabolic ward studies. BMJ 1997;314:112–117. 9006469 10.1136/bmj.314.7074.112 PMC2125600

[23] Higgins JPT , Thompson SG, Deeks JJ, Altman DG. Measuring inconsistency in meta-analyses. BMJ 2003;327:557–560. 12958120 10.1136/bmj.327.7414.557 PMC192859

[24] Risérus U , Marklund M. Milk fat biomarkers and cardiometabolic disease. Curr Opin Lipidol 2017;28:46–51. 27906713 10.1097/MOL.0000000000000381 PMC5214382

[25] Praagman J , Beulens JWJ, Alssema M, Zock PL, Wanders AJ, Sluijs I, et al The association between dietary saturated fatty acids and ischemic heart disease depends on the type and source of fatty acid in the European prospective investigation into cancer and nutrition–Netherlands cohort. Am J Clin Nutr 2016;103:356–365. 26791181 10.3945/ajcn.115.122671

[26] Weitkunat K , Bishop CA, Wittmüss M, Machate T, Schifelbein T, Schulze MB, et al Effect of microbial Status on hepatic odd-chain fatty acids is diet-dependent. Nutrients 2021;13:1546. 34064336 10.3390/nu13051546 PMC8147859

[27] Jenkins B , West J, Koulman A. A review of odd-chain fatty acid metabolism and the role of pentadecanoic acid (C15:0) and heptadecanoic acid (C17:0) in health and disease. Molecules 2015;20:2425–2444. 25647578 10.3390/molecules20022425 PMC6272531

[28] Prada M , Wittenbecher C, Eichelmann F, Wernitz A, Drouin-Chartier J-P, Schulze MB. Association of the odd-chain fatty acid content in lipid groups with type 2 diabetes risk: a targeted analysis of lipidomics data in the EPIC-Potsdam cohort. Clin Nutr 2021;40:4988–4999. 34364238 10.1016/j.clnu.2021.06.006

[29] Khan SU , Lone AN, Khan MS, Virani SS, Blumenthal RS, Nasir K, et al Effect of omega-3 fatty acids on cardiovascular outcomes: a systematic review and meta-analysis. eClinicalMedicine 2021;38:100997. 34505026 10.1016/j.eclinm.2021.100997 PMC8413259

[30] Kaur G , Mason RP, Steg PG, Bhatt DL. Omega-3 fatty acids for cardiovascular event lowering. Eur J Prev Cardiol 2024;31:1005–1014. 38169319 10.1093/eurjpc/zwae003

[31] Klingel SL , Metherel AH, Irfan M, Rajna A, Chabowski A, Bazinet RP, et al EPA and DHA have divergent effects on serum triglycerides and lipogenesis, but similar effects on lipoprotein lipase activity: a randomized controlled trial. Am J Clin Nutr 2019;110:1502–1509. 31535138 10.1093/ajcn/nqz234

[32] So J , Wu D, Lichtenstein AH, Tai AK, Matthan NR, Maddipati KR, et al EPA and DHA differentially modulate monocyte inflammatory response in subjects with chronic inflammation in part via plasma specialized pro-resolving lipid mediators: a randomized, double-blind, crossover study. Atherosclerosis 2021;316:90–98. 33303222 10.1016/j.atherosclerosis.2020.11.018

[33] Mori TA , Burke V, Puddey IB, Watts GF, O’Neal DN, Best JD, et al Purified eicosapentaenoic and docosahexaenoic acids have differential effects on serum lipids and lipoproteins, LDL particle size, glucose, and insulin in mildly hyperlipidemic men. Am J Clin Nutr 2000;71:1085–1094. 10799369 10.1093/ajcn/71.5.1085

[34] Sherratt SCR , Juliano RA, Copland C, Bhatt DL, Libby P, Mason RP. EPA and DHA containing phospholipids have contrasting effects on membrane structure. J Lipid Res 2021;62:100106. 34400132 10.1016/j.jlr.2021.100106 PMC8430377

[35] Mozaffarian D , Wu JHY. (n-3) fatty acids and cardiovascular health: are effects of EPA and DHA shared or complementary? J Nutr 2012;142:614S–625S. 22279134 10.3945/jn.111.149633 PMC3278271

[36] Maki KC , Bobotas G, Dicklin MR, Huebner M, Keane WF. Effects of MAT9001 containing eicosapentaenoic acid and docosapentaenoic acid, compared to eicosapentaenoic acid ethyl esters, on triglycerides, lipoprotein cholesterol, and related variables. J Clin Lipidol 2017;11:102–109. 28391875 10.1016/j.jacl.2016.10.010

[37] Bjermo H , Iggman D, Kullberg J, Dahlman I, Johansson L, Persson L, et al Effects of n-6 PUFAs compared with SFAs on liver fat, lipoproteins, and inflammation in abdominal obesity: a randomized controlled trial. Am J Clin Nutr 2012;95:1003–1012. 22492369 10.3945/ajcn.111.030114

[38] Imamura F , Micha R, Wu JHY, de Oliveira Otto MC, Otite FO, Abioye AI, et al Effects of saturated fat, polyunsaturated fat, monounsaturated fat, and carbohydrate on glucose-insulin homeostasis: a systematic review and meta-analysis of randomised controlled feeding trials. PLOS Med 2016;13:e1002087. 27434027 10.1371/journal.pmed.1002087 PMC4951141

[39] Miura K , Stamler J, Nakagawa H, Elliott P, Ueshima H, Chan Q, et al Relationship of dietary linoleic acid to blood pressure. Hypertension 2008;52:408–414. 18606902 10.1161/HYPERTENSIONAHA.108.112383 PMC6668335

[40] Farvid MS , Ding M, Pan A, Sun Q, Chiuve SE, Steffen LM, et al Dietary linoleic acid and risk of coronary heart disease: a systematic review and meta-analysis of prospective cohort studies. Circulation 2014;130:1568–1578. 25161045 10.1161/CIRCULATIONAHA.114.010236 PMC4334131

[41] Murff HJ , Edwards TL. Endogenous production of long-chain polyunsaturated fatty acids and metabolic disease risk. Curr Cardiovasc Risk Rep 2014;8:418. 26392837 10.1007/s12170-014-0418-1 PMC4574498

[42] Wu JHY , Marklund M, Imamura F, Tintle N, Ardisson Korat AV, de Goede J, et al Omega-6 fatty acid biomarkers and incident type 2 diabetes: pooled analysis of individual-level data for 39 740 adults from 20 prospective cohort studies. Lancet Diabetes Endocrinol 2017;5:965–974. 29032079 10.1016/S2213-8587(17)30307-8 PMC6029721

[43] Matthan NR , Ooi EM, Van Horn L, Neuhouser ML, Woodman R, Lichtenstein AH. Plasma phospholipid fatty acid biomarkers of dietary fat quality and endogenous metabolism predict coronary heart disease risk: a nested case-control study within the women’s health initiative observational study. J Am Heart Assoc 2014;3:e000764. 25122663 10.1161/JAHA.113.000764 PMC4310362

[44] Holmes MV , Millwood IY, Kartsonaki C, Hill MR, Bennett DA, Boxall R, et al Lipids, lipoproteins, and metabolites and risk of myocardial infarction and stroke. J Am Coll Cardiol 2018;71:620–632. 29420958 10.1016/j.jacc.2017.12.006 PMC5811927

[45] Zong G , Li Y, Sampson L, Dougherty LW, Willett WC, Wanders AJ, et al Monounsaturated fats from plant and animal sources in relation to risk of coronary heart disease among US men and women. Am J Clin Nutr 2018;107:445 453. 29566185 10.1093/ajcn/nqx004 PMC5875103

[46] Ntambi JM , Miyazaki M. Regulation of stearoyl-CoA desaturases and role in metabolism. Prog Lipid Res 2004;43:91–104. 14654089 10.1016/s0163-7827(03)00039-0

[47] Harris WS , Pottala JV, Vasan RS, Larson MG, Robins SJ. Changes in erythrocyte membrane trans and marine fatty acids between 1999 and 2006 in older Americans. J Nutr 2012;142:1297–1303. 22623386 10.3945/jn.112.158295 PMC3374668

[48] Zheng J-S , Imamura F, Sharp SJ, Koulman A, Griffin JL, Mulligan AA, et al Changes in plasma phospholipid fatty acid profiles over 13 years and correlates of change: European prospective investigation into cancer and nutrition-norfolk study. Am J Clin Nutr 2019;109:1527–1534. 30997506 10.1093/ajcn/nqz030 PMC6537938

[49] Hodson L , Skeaff CM, Fielding BA. Fatty acid composition of adipose tissue and blood in humans and its use as a biomarker of dietary intake. Prog Lipid Res 2008;47:348–380. 18435934 10.1016/j.plipres.2008.03.003

[50] Chitsazan M , Chitsazan M. The role of free fatty acids as a prognostic biomarker in coronary artery disease patients with type 2 diabetes. Eur J Prev Cardiol 2023;30:728–729. 37069738 10.1093/eurjpc/zwad100

