# Supplementary material for: Association of circulating fatty acids with cardiovascular disease risk: analysis of individual-level data in three large prospective cohorts and updated meta-analysis
Source: Eur J Prev Cardiol. 2024 Oct 4;32(3):233–46. doi: 10.1093/eurjpc/zwae315 (PMC11832215; doi:10.1093/eurjpc/zwae315)
Supplement: zwae315_Supplementary_Data [file zwae315_supplementary_data.pdf]

Association of circulating fatty acids with cardiovascular disease risk: Analysis of individual-level data in three large prospective cohorts and updated meta-analysis

Supplementary Data

eMethods ... **3**

Study Design and Population ... **3**

Supplementary eFigure 1. Flowchart of the inclusion and exclusion of participants from UKB, INTERVAL, and EPIC-CVD ... 3

Assessment of Fatty Acids ... **4**

Supplementary eTable 1. Calculation of main fatty acid subtypes in EPIC-CVD. ... 4

Supplementary eFigure 2. Schematic showing the structure and names of FA subtypes and individual FAs on investigation, as well as their biosynthesis pathways[14–17] ... 6

Covariates ... **7**

Outcomes ascertainment ... **7**

Systematic review and updated meta-analysis ... **8**

Supplementary eTable 2. Literature search strategy for all the databases ... 10

eResults: Tables ... ***12***

Supplementary eTable 3. Summary of baseline characteristics, as well as correlations with SFA, MUFA, and PUFA ... 12

Supplementary eTable 4. Detailed baseline FA concentrations of EPIC-CVD participants by subcohort and CVD status. ... 13

Supplementary eTable 5. Hazard ratios per 1-SD higher FAs for cardiovascular events in primary analysis (estimated in 172,891 participants from EPIC-CVD, UKB and INTERVAL) and sensitivity analysis 1 (excluding follow-up within 2 years from baseline) ... 14

Supplementary eTable 6. Hazard ratios per 1-SD higher FAs for cardiovascular events, with further adjustments estimated in 167,620 participants from EPIC-CVD, UKB and INTERVAL. ... 15

Supplementary eTable 7. Associations of plasma phospholipids total SFA, SFA subtypes and individual SFAs with CHD and stroke, with further adjustments estimated from EPIC-CVD participants (subcohort n=15,125). ... 16

Supplementary eTable 8. Associations of plasma phospholipids total and individual MUFAs with CHD and stroke, with further adjustments estimated from EPIC-CVD participants (subcohort n=15,125). ... 17

Supplementary eTable 9. Associations of plasma phospholipids total PUFA, PUFA subtypes and individual PUFAs with CHD and stroke, with further adjustments estimated from EPIC-CVD participants (subcohort n=15,125). ... 18

Supplementary eTable 10. Associations of plasma phospholipids total and individual TFAs with CHD and stroke, with further adjustments estimated from EPIC-CVD participants (subcohort n=15,125). ... 19

Supplementary eTable 11. Associations of plasma phospholipids FA ratio variables with CHD and stroke, with further adjustments estimated from EPIC-CVD participants (subcohort n=15,125). ... 20

Supplementary eTable 12. Associations of FA subtypes with CHD and stroke in sensitivity analysis 2, adjusted for conventional CVD risk factors and plus further adjustments for CRP and HbA1C or glucose ... 20

Supplementary eTable 13. Comparisons between associations of fatty acids in absolute and relative concentrations with CHD and stroke in sensitivity analysis 3, estimated from UKB and INTERVAL. ... 21

Supplementary eTable 14. Estimated regression dilution ratios for fatty acids and corrected hazard ratios in sensitivity analysis 4. ... 22

Supplementary eTable 15. Hazard ratios and subdistribution hazard ratios per 1-SD higher FAs for cardiovascular events adjusted for lifestyle factors without/with adjustment for competing risks in sensitivity analysis 5, estimated in 172,891 participants from EPIC-CVD, UKB and INTERVAL ... 23

Supplementary eTable 16. The study profiles of identified systematic reviews with meta-analysis on FA and cardiovascular outcomes, as well as the inclusion of eligible primary studies ... 24

Supplementary eTable 17. Characteristics of 49 non-duplicated primary studies included in the updated meta-analysis of associations of FA biomarkers with CHD and stroke ... 25

Supplementary eTable 18. Further characteristics of non-duplicated associations of fatty acid biomarkers with CHD included in the updated meta-analyses ... 29

Supplementary eTable 19. Further characteristics of non-duplicated associations of fatty acid biomarkers with stroke included in the updated meta-analyses ... 36
Supplementary eTable 20. Further characteristics of non-duplicated associations of fatty acid biomarkers with ischaemic stroke included in the updated meta-analyses ... 41
Supplementary eTable 21. The evidence for CHD and stroke, summarized from the most comprehensive meta-analyses of randomized controlled trials on fatty acids supplementation ... 45

eResults: Figures ... 46

Supplementary eFigure 3. Heatmap of correlations between plasma phospholipid FAs (%) and self-reported food intake (g/day) in EPIC-CVD study ... 46
Supplementary eFigure 4A-G. Hazard ratios per 1-SD higher FAs for CHD and stroke by explored baseline characteristics, estimated from EPIC-CVD, UKB and INTERVAL studies. ... 47
Supplementary eFigure 5A-C. Hazard ratios per 1-SD higher SFA subtypes for CHD and stroke by explored baseline characteristics, estimated from EPIC-CVD participants. ... 54
Supplementary eFigure 6. Literature review flow diagram. ... 57
Supplementary eFigure 7. Updated meta-analysis combining results from EPIC-CVD, UKB and INTERVAL studies with published evidence for associations of fatty acid biomarkers with ischaemic stroke. ... 58
Supplementary eFigure 8. Associations of FAs with CHD risk by geographical region. ... 59
Supplementary eFigure 9. Associations of FAs with stroke risk by geographical region. ... 61
Supplementary eFigure 10. Associations of FAs with CHD risk by lipid compartments. ... 63
Supplementary eFigure 11. Associations of FAs with stroke risk by lipid compartments. ... 65

Reference ... 67

eMethods

Study Design and Population

Supplementary eFigure 1. Flowchart of the inclusion and exclusion of participants from UKB, INTERVAL, and EPIC-CVD

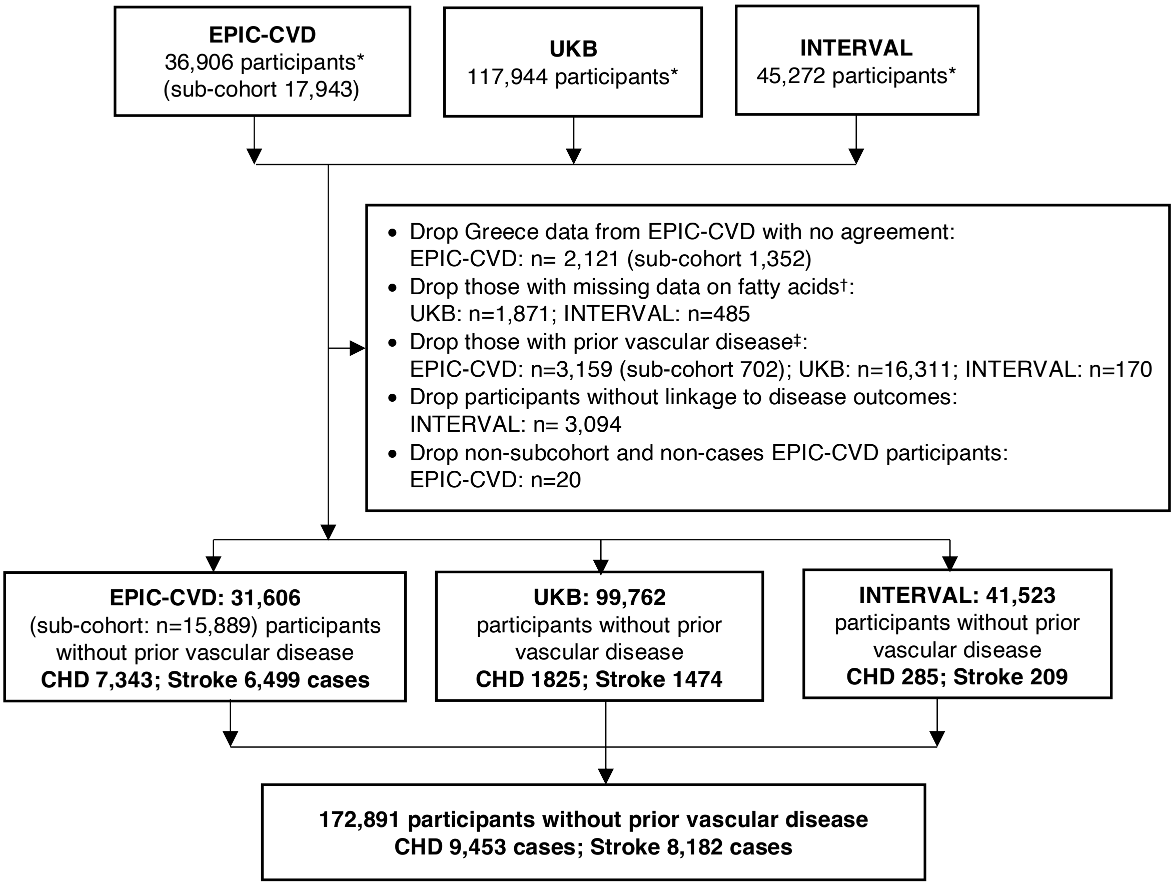

* Participants involved in the measurement of fatty acids.

† To maximize the sample size, we focused on the primary exposures of interest as described in Methods, i.e., saturated fatty acid and its three subtypes, monounsaturated fatty acid, n-3 and n-6 polyunsaturated fatty acid, docosahexaenoic acid, and linoleic acid. In EPIC-CVD, all participants had complete measurements on 38 individual fatty acids except C16:1t and missing data on C16:1t was not considered in the inclusion criteria here. In INTERVAL, the measurement on one trans fatty acid (i.e., conjugated linoleic acid) was also provided though missing data existed, and it was also not considered in the inclusion criteria here. Missingness of FAs was 1.6% and 1.1% in UKB and INTERVAL, respectively.

‡ Prior vascular disease was defined as any history of heart disease, stroke, transient ischemic attack, peripheral vascular disease, or cardiovascular surgery at baseline. Information on prior vascular disease was based on questionnaires (EPIC-CVD and UKB) or data linkage to medical records (INTERVAL and UKB).

Assessment of Fatty Acids

In EPIC-CVD, phospholipid fatty acids (FAs) were profiled at the Medical Research Council Human Nutrition Research laboratory (Cambridge, UK) by analyzing plasma samples stored at baseline at -196°C (-150°C in Denmark), a temperature at which FAs remain stable[1]. The assay methods have previously been described[2], including (i) hydrolysis and methylation to convert phospholipid FAs into more volatile fatty acid methyl esters (FAMEs); (ii) separation of different FAMEs by gas chromatography (GC) (J&W HP-88, 30 m length) equipped with flame ionisation detection (7890N GC [Agilent Technologies]); (iii) identification of FAMEs by comparison of their retention times with those of individual FAME standards; and finally, (iv) measurements of the relative quantities of individual FAs, expressed as percentages (%) of the total FA signal[2]. In the chromatogram, all compounds in the standard mixture were well resolved with the exception of C20:3n3 and C20:4n6, which co-eluted; however, the level of C20:3n3 in human samples is negligible, and therefore this peak was annotated as C20:4n6[2,3]. Human and equine plasma (Sera Laboratories International, West Sussex, UK) were used as quality control samples and included in each batch[2]. A total of 38 individual plasma phospholipid FAs were identified and 28 of them with average relative concentrations higher than 0.05% were finally included in the current analysis and used to calculate the concentrations of main FA subtypes **(Supplementary eTable 1 & eFigure 2)**. Several estimates for plasma desaturase enzyme activities were also calculated, including stearoyl-CoA-desaturase 16:1n7/ 16:0 (SCD-16) and 18:1n9/ 18:0 (SCD-18)[4,5], D6-desaturase (D6D; 18:3n6/ 18:2n6)[6–8], D5-desaturase (D5D; 20:4n6/ 20:3n6)[7,8], and 20:3n6 to18:2n6 ratio[8].

Supplementary eTable 1. Calculation of main fatty acid subtypes in EPIC-CVD.

Fatty acid subtypes		Individual fatty acids included in the calculation
**SFA**	**Even-chain SFA (n=3)**	C14:0, C16:0, C18:0
	**Odd-chain SFA (n=2)**	C15:0, C17:0
	**Longer-chain SFA (n=4)**	C20:0, C22:0, C23:0, C24:0
	*SFAs with relative concentrations < 0.05 % (n=6)	C8:0, C10:0, C11:0, C12:0, C13:0, C21:0
**MUFA**	(n=5)	C16:1, C17:1, C18:1, C20:1, C24:1
	*MUFAs with relative concentrations < 0.05 % (n=3)	C14:1, C15:1, C22:1
**PUFA**	**n-3 PUFA (n=4)**	C18:3n3, C20:5n3, C22:5n3, C22:6n3
	**n-6 PUFA (n=7)**	C18:2n6, C18:3n6, C20:2n6, C20:3n6, C22:4n6, C22:5n6, C20:4n6
	*PUFA with relative concentrations < 0.05 % (n=1)	C22:2
trans-FA†	(n=3)	C18:1n9t, C18:2n6t, C16:1t

FA: Fatty acid; MUFA: Monounsaturated fatty acid; PUFA: Polyunsaturated fatty acid; SFA: Saturated fatty acid.
Individual fatty acids with average relative concentrations higher than 0.05% were included in the further analysis.
* The 10 individual FAs with average relative concentrations < 0.05 % were excluded in the calculation and analysis.
† Among trans-FAs, C16:1t was measured in 22,890 out of 31,606 EPIC-CVD participants.

In UKB, FAs in total plasma (i.e., all the FAs in triglycerides, phospholipids, cholesterol esters, or as free FAs)[9] were assayed using a high-throughput nuclear magnetic resonance (NMR) metabolomics platform developed by Nightingale Health Ltd (Helsinki, Finland), which has been described previously[10]. The metabolomics data in UKB were quantified between 2019 and 2020 from non-fasting plasma samples collected at baseline of a random sample of the full UKB cohort (~120,000 participants) for Phase 1 data release. Similarly, total serum or plasma FAs in INTERVAL were also quantified by the Nightingale platform but at a much earlier date (around 2015). Among the final included INTERVAL sample (n=41,523), FA profiles of most participants (n=41,478) were assayed in the total serum, with those of the remaining 45 participants being assayed in the total plasma. Nevertheless, experimental evidence suggests that FAs from matched plasma and serum samples reveal

highly comparable absolute and relative levels[11]. Overall, five FA subtypes (i.e., SFA, MUFA, PUFA, n-3 PUFA, and n-6 PUFA) and two individual FAs (i.e., linoleic acid [LA; 18:2n6] and docosahexaenoic acid [DHA; 22:6n3]) were all identified in both studies, with conjugated linoleic acid being additionally assayed in INTERVAL only.

In NMR, the concentration of n-3 PUFA (as an example) is quantified based on a spectral signal that arises from all the FAs containing the n-3 double bond, and therefore n-3 PUFA is not calculated as a sum of known concentrations of individual n-3 PUFAs[12]. Thus, the components of FA subtypes in UKB and INTERVAL might be slightly different from those in EPIC-CVD given the different assay methods. However, there should not be much difference as all major components have been included in the calculation of FA subtypes. The resolution is another main difference between NMR and GC methods for FA profiling. For example, the DHA signal is quantified separately from a specific NMR signal unique to this molecule, whereas the resolution of high-throughput NMR does currently not allow sufficient resolution for the robust and independent quantification of eicosapentaenoic acid (EPA; 20:5n3) due to spectral overlap[12]. Despite these two analytical techniques used between studies, the high correspondence of the quantification of FAs, either individually (e.g., DHA) or in combination (e.g., total PUFA), between NMR and GC (Pearson correlation $r > 0.92$) further supported our combined analysis[13].

Supplementary eFigure 2. Schematic showing the structure and names of FA subtypes and individual FAs on investigation, as well as their biosynthesis pathways[14–17]

(A) Types of fatty acids[14]

Saturated	Monounsaturated	Polyunsaturated	Trans
• no double bond<br>• straight structure	• one double bond in cis configuration<br>• bent structure	• multiple double bonds in cis configuration<br>• even more "bent" in structure	• one or more double bonds in trans configuration<br>• straight structure

(B) Detailed structure and names of fatty acid subtypes[15–17]

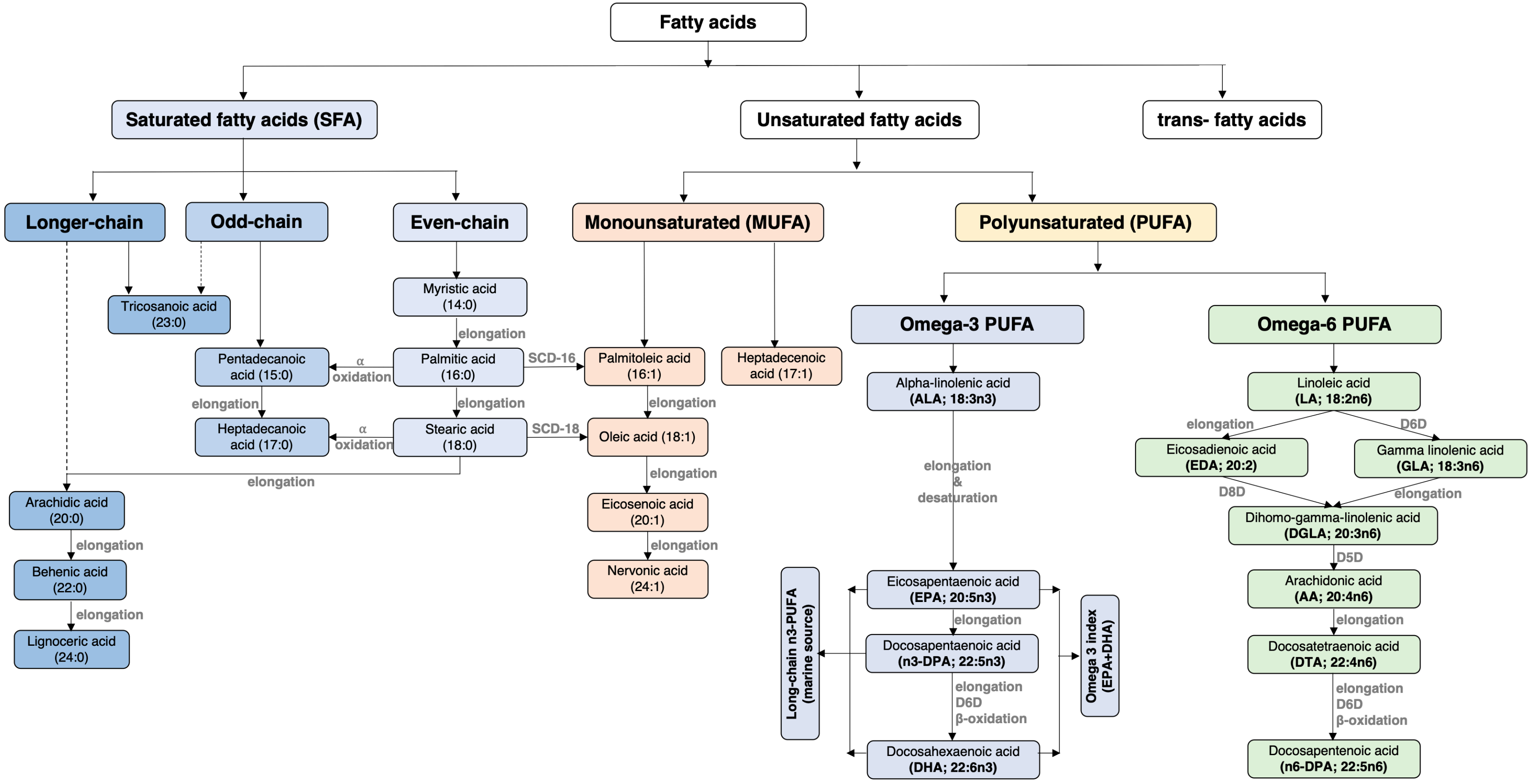

SCD: stearoyl-CoA-desaturase; D5D: D5-desaturase; D6D: D6-desaturase; D8D: D8-desaturase.

Covariates

Baseline information on food intakes (g/day) were not included in further adjustments in UKB and INTERVAL because only 14.2% of the current included UKB participants completed the 24-hour recall questionnaire while the 24-hour recall questionnaire was not included in the baseline survey of INTERVAL. Data on history of hypertension and history of diabetes was also not available from INTERVAL, however, INTERVAL participants were considered as generally healthy given they were all blood donors. In EPIC-CVD, physical activity was assessed by Cambridge index of physical activity with four categories (i.e., inactive, moderately inactive, moderately active, and active), which was further categorized into "inactive" and "active". In INTERVAL, physical activity was first estimated by the combination of occupational and leisure-time physical activity: i) the self-evaluated four levels of leisure-time physical activity was used to approximate the four levels of time spent in sports and cycling in the Cambridge index of physical activity; ii) then, a simple four-level variable of physical activity was obtained based on similar rules of the Cambridge index of physical activity[18], which was further categorized into "inactive" and "active". In UKB, physical activity was assessed by the metabolic equivalent task (MET) scores based on International Physical Activity Questionnaire guidelines[19] and individuals are considered physically active when they achieved MET minutes of 600 or more per week, and otherwise inactive[20]. To make use of most the available data in each study, adjustments for baseline lipids involved those measured by conventional biochemistry assays in EPIC-CVD and those measured by the NMR platform in UKB and INTERVAL.

The following summarizes the missing data on the covariates across three studies, separately and collectively:

Cohort	Categorical Covariates, n (%)				
	Smoking	*History of diabetes	*History of Hypertension	Physical activity	Alcohol intake
**EPIC-CVD** (n=31,606)	305 (1.0)	3,846 (12.2)	406 (1.3)	580 (1.8)	78 (0.2)
**UKB** (n=99,762)	141 (0.1)	372 (0.4)	293 (0.3)	18,753 (18.8)	204 (0.2)
**INTERVAL** (n= 41,523)	579 (1.4)	---	---	809 (2.0)	512 (1.2)
**Overall** (n=172,891)	1,025 (0.6)	4,218 (3.2)	699 (0.5)	20,142 (11.7)	794 (0.5)

Cohort	Continuous Covariates, n (%)					
	BMI	TC	HDL-C	TG	*Dietary intakes	Overall
**EPIC-CVD** (n=31,606)	185 (0.6)	3,140 (9.9)	2,407 (7.6)	3,243 (10.3)	117 (0.4)	3,524 (11.1)
**UKB** (n=99,762)	331 (0.3)	760 (0.8)	362 (0.4)	752(0.8)	---	1,533 (1.5)
**INTERVAL** (n= 41,523)	214 (0.5)	0 (0.0)	0 (0.00)	0 (0.00)	---	214 (0.5)
**Overall** (n=172,891)	730 (0.4)	3,900 (2.3)	2,769 (1.6)	3,995 (2.3)	117 (0.4)	5,176 (3.0)

* History of diabetes and history of hypertension were not assessed in INTERVAL, but participants were considered generally healthy as they were blood donors with low prevalence expected. Adjustments for dietary intakes were only included in the analysis of EPIC-CVD.

The primary analysis was stratified by sex and adjusted for conventional risk factors, including age, smoking, history of diabetes, history of hypertension, and physical activity. Missing values of categorical covariates were coded as a separate category in adjustments using dummy variables, which has been shown to induce minimal bias[21]. In further analyses adjusted for lifestyle factors that may be related to diet (i.e., alcohol intake and BMI), dietary intake, and lipids, complete-case analyses excluding those with missing data on continuous covariates were conducted.

Outcomes ascertainment

In UKB and INTERVAL, follow-up for incident outcomes was conducted mainly through linkages to routinely available national datasets, including Hospital Episode Statistics (HES), as well as national death and cancer

registries. In EPIC-CVD, cardiovascular events, including coronary heart disease and stroke, were ascertained by different methods depending on the follow-up procedures used by each centre, including active follow-up through questionnaires, linkage with registries, medical records, or a combination of these. Nonfatal events were further validated by additional review of medical records and/or linkage with registries. Fatal events were generally ascertained through mortality registries. Within each contributing centre, information has been collected and centrally harmonised at the EPIC-CVD Coordinating Centre.

Besides, history of disease at baseline was defined by i) self-reported questionnaires in EPIC-CVD; ii) medical records in INTERVAL; and iii) a combination of self-report and medical records in UKB (i.e., using self-reported information recorded at baseline visit and updated using information on hospitalization before baseline extracted from HES).

Systematic review and updated meta-analysis

Data extraction:

From each retrieved systematic review with meta-analysis on associations between FA biomarkers and CVD risks, the following characteristics were extracted and summarised: name of first author, year of publication, data sources, searched time, number of included articles, FAs of interest, outcomes of interest, eligible primary studies on CHD or stroke, and reasons for exclusion of primary studies.

From each retrieved primary studies, the following characteristics were extracted and summarised: name of first author, year of publication, study design, geographical location, data source, population type, sample size, follow-up years, proportion of female participants, age, baseline year, specimen, FAs of interest, outcome of interest, number of cases, reported association estimates, scale of reported estimates, and degree of statistical adjustment for covariates. The extracted estimates were standardized to correspond to relative risks (RR) per 1 SD higher FA using established methods as described below.

From each retrieved systematic review with meta-analysis of randomized trials, the following characteristics were extracted and summarised: name of first author, year of publication, data sources, searched time, FAs of interest, outcomes of interest, number of trials, number of participants, relative effect, certainty of evidence, and comments.

For duplicated RRs: The one estimated from the largest sample size or from the cohort with longer follow-up duration was selected if separate primary studies reported duplicate RRs using the same study population. When one primary study reported several estimates with different degrees of adjustments for the same FA-outcome association, the one obtained from the largest sample size was selected in priority, or the most adjusted one that did not include adjustment for lipids or circulating FA biomarkers were selected in the case of the same sample size.

Conversion of RRs: RRs extracted from each primary study may be reported in different scales and an established method[22] was used to harmonise the RRs to a common scale (per 1-SD) as described below. Given the standard normal distribution, the means of some quantile groups are summarised below.

-0.80	0.80					
-1.09	0	1.09				
-1.27	-0.32	0.32	1.27			
-1.40	-0.53	0	0.53	1.40		
-1.50	-0.68	-0.21	0.21	0.68	1.50	
-1.58	-0.80	-0.37	0	0.37	0.80	1.58

The means of some quantile groups, N(0,1).

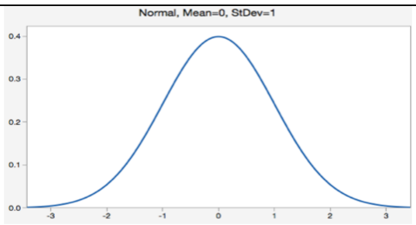

Assuming the normal distribution of exposure and a linear association with disease risk[22], the log RR for the highest versus lowest third of exposure, for instance, is expected to correspond to 2.18 SD higher exposure, while the log RR for quintile 5 versus quintile 3 of exposure, for instance, is expected to correspond to 1.40 SD higher exposure. Thus, using an RR comparing the highest versus lowest third of exposure of 0.83 (95%CI [confidence interval]: 0.54-1.28) as an example, the corresponding RR per 1-SD can be obtained using the following formulas:

lnRR= ln(0.83)

SElnRR= (log upper relative risk- log lower relative risk)/(2*1.96)

Converted Relative Risk= exp(lnRR/2.18)

95%CI: (exp((lnRR-1.96*SElnRR)/2.18), exp((lnRR+1.96*SElnRR)/2.18))

Conversion factor= lncRR / lnRR

SElnRR: Standard error of log relative risk; lnRR: log relative risk; lncRR: log converted relative risk

Finally, the converted RR per 1-SD higher exposure is 0.92 (95%CI: 0.75-1.12), with a conversion factor of 0.46.

Supplementary eTable 2. Literature search strategy for all the databases

PubMed, search through 6 May 2023	Results
("Fatty Acids"[Mesh] OR "Fatty Acids, Unsaturated"[Mesh] OR "Fatty Acids, Omega-6"[Mesh] OR "Fatty Acids, Omega-3"[Mesh] OR "Fatty Acids, Monounsaturated"[Mesh] OR "Fatty Acids, Nonesterified"[Mesh] OR "Fatty Acids, Essential"[Mesh] OR "Trans Fatty Acids"[Mesh] OR "Fatty Acids" OR "Saturated Fatty Acids" OR "Unsaturated Fatty Acids" OR "Omega-6 Fatty Acids" OR "Omega-3 Fatty Acids" OR "Polyunsaturated Fatty Acids" OR "Monounsaturated Fatty Acids" OR "Essential Fatty Acids" OR "Trans Fatty Acids" OR "Serum Fatty Acids" OR "Plasma Fatty Acids" OR "Blood Fatty Acids" OR "Circulating Fatty Acids" OR "Erythrocyte Fatty Acids" OR "Phospholipid Fatty Acids" OR "Cholesteryl Ester Fatty Acids" OR "Adipose Tissue Fatty Acids" OR "Dietary Fatty Acids" OR "Fatty Acids Intake" OR "Myristic Acid" OR "Palmitic Acid" OR "Stearic Acid" OR "Pentadecanoic Acid" OR "Margaric Acid" OR "Heptadecanoic Acid" OR "Arachidic Acid" OR "Behenic Acid" OR "Tricosanoic Acid" OR "Lignoceric Acid" OR "Caprylic Acid" OR "Capric Acid" OR "Lauric Acids" OR "Palmitoleic Acid" OR "Oleic Acid" OR "Gondoic Acid" OR "Nervonic Acid" OR "Myristoleic Acid" OR "Erucic Acid" OR "alpha-Linolenic Acid" OR "Eicosapentaenoic Acid" OR "Docosahexaenoic Acid" OR "Docosapentaenoic Acid" OR "Linoleic Acid" OR "Gamma-linolenic Acid" OR "Eicosadienoic Acid" OR "Dihomo-gamma-linolenic Acid" OR "Arachidonic Acid" OR "Adrenic Acid" OR "Osbond Acid" OR "Docosadienoic Acid" OR "Linolenic Acid" OR "Conjugated Linoleic Acid" OR "Elaidic Acid")	1,270
**AND** ("Cardiovascular Diseases"[Mesh] OR "Cardiovascular Diseases" OR "Cardiovascular Disease" OR "Cardiovascular Event" OR "Coronary disease" OR "Coronary heart disease" OR "Coronary artery disease" OR "Ischemic Heart Disease" OR "Ischaemic Heart Disease" OR "Myocardial ischaemia" OR "Myocardial ischemia" OR "Myocardial infarction" OR "Heart attack" OR "Heart failure" OR "Cardiocerebrovascular disease" OR "Cerebrovascular disease" OR "Stroke" OR "Cerebrovascular accident" OR "Cardiovascular mortality" OR "All-cause Mortality" OR "Mortality" OR "Survival")	
**AND** ("Review Literature as Topic"[Mesh] OR "Systematic Review" OR "Meta-Analysis")	
Filter: English language	1,222
**Scientific Citation Index Expanded, search through 6 May 2023**	
TS= ("Fatty Acids" OR "Saturated Fatty Acids" OR "Unsaturated Fatty Acids" OR "Omega-6 Fatty Acids" OR "Omega-3 Fatty Acids" OR "Polyunsaturated Fatty Acids" OR "Monounsaturated Fatty Acids" OR "Essential Fatty Acids" OR "Trans Fatty Acids" OR "Serum Fatty Acids" OR "Plasma Fatty Acids" OR "Blood Fatty Acids" OR "Circulating Fatty Acids" OR "Erythrocyte Fatty Acids" OR "Phospholipid Fatty Acids" OR "Cholesteryl Ester Fatty Acids" OR "Adipose Tissue Fatty Acids" OR "Dietary Fatty Acids" OR "Fatty Acids Intake" OR "Myristic Acid" OR "Palmitic Acid" OR "Stearic Acid" OR "Pentadecanoic Acid" OR "Margaric Acid" OR "Heptadecanoic Acid" OR "Arachidic Acid" OR "Behenic Acid" OR "Tricosanoic Acid" OR "Lignoceric Acid" OR "Caprylic Acid" OR "Capric Acid" OR "Lauric Acids" OR "Palmitoleic Acid" OR "Oleic Acid" OR "Gondoic Acid" OR "Nervonic Acid" OR "Myristoleic Acid" OR "Erucic Acid" OR "alpha-Linolenic Acid" OR "Eicosapentaenoic Acid" OR "Docosahexaenoic Acid" OR "Docosapentaenoic Acid" OR "Linoleic Acid" OR "Gamma-linolenic Acid" OR "Eicosadienoic Acid" OR "Dihomo-gamma-linolenic Acid" OR "Arachidonic Acid" OR "Adrenic Acid" OR "Osbond Acid" OR "Docosadienoic Acid" OR "Linolenic Acid" OR "Conjugated Linoleic Acid" OR "Elaidic Acid")	872
**AND**	
TS= ("Cardiovascular Diseases" OR "Cardiovascular Disease" OR "Cardiovascular Event" OR "Coronary disease" OR "Coronary heart disease" OR "Coronary artery disease" OR "Ischemic Heart Disease" OR "Ischaemic Heart Disease" OR "Myocardial ischaemia" OR "Myocardial ischemia" OR "Myocardial infarction" OR "Heart attack" OR "Heart failure" OR "Cardiocerebrovascular disease" OR "Cerebrovascular disease" OR "Stroke" OR "Cerebrovascular accident" OR "Cardiovascular mortality" OR "All-cause Mortality" OR "Mortality" OR "Survival")	
**AND**	
TS = ("Systematic Review" OR "Meta-Analysis")	
Filter: English language	864
**EMBASE, search through 6 May 2023**	
("Fatty Acids" OR "Saturated Fatty Acids" OR "Unsaturated Fatty Acids" OR "Omega-6 Fatty Acids" OR "Omega-3 Fatty Acids" OR "Polyunsaturated Fatty Acids" OR "Monounsaturated Fatty Acids" OR "Essential Fatty Acids" OR "Trans Fatty Acids" OR "Serum Fatty Acids" OR "Plasma Fatty Acids" OR "Blood Fatty Acids" OR "Circulating Fatty Acids" OR "Erythrocyte Fatty Acids" OR "Phospholipid Fatty Acids" OR "Cholesteryl Ester Fatty Acids" OR "Adipose Tissue Fatty Acids" OR "Dietary Fatty Acids" OR "Fatty Acids Intake" OR "Myristic Acid" OR "Palmitic Acid" OR "Stearic Acid" OR	1,307

"Pentadecanoic Acid" OR "Margaric Acid" OR "Heptadecanoic Acid" OR "Arachidic Acid" OR "Behenic Acid" OR "Tricosanoic Acid" OR "Lignoceric Acid" OR "Caprylic Acid" OR "Capric Acid" OR "Lauric Acids" OR "Palmitoleic Acid" OR "Oleic Acid" OR "Gondoic Acid" OR "Nervonic Acid" OR "Myristoleic Acid" OR "Erucic Acid" OR "alpha-Linolenic Acid" OR "Eicosapentaenoic Acid" OR "Docosahexaenoic Acid" OR "Docosapentaenoic Acid" OR "Linoleic Acid" OR "Gamma-linolenic Acid" OR "Eicosadienoic Acid" OR "Dihomo-gamma-linolenic Acid" OR "Arachidonic Acid" OR "Adrenic Acid" OR "Osbond Acid" OR "Docosadienoic Acid" OR "Linolenic Acid" OR "Conjugated Linoleic Acid" OR "Elaidic Acid").af.<br>**AND**<br>("Cardiovascular Diseases" OR "Cardiovascular Disease" OR "Cardiovascular Event" OR "Coronary disease" OR "Coronary heart disease" OR "Coronary artery disease" OR "Ischemic Heart Disease" OR "Ischaemic Heart Disease" OR "Myocardial ischaemia" OR "Myocardial ischemia" OR "Myocardial infarction" OR "Heart attack" OR "Heart failure" OR "Cardiocerebrovascular disease" OR "Cerebrovascular disease" OR "Stroke" OR "Cerebrovascular accident" OR "Cardiovascular mortality" OR "All-cause Mortality" OR "Mortality" OR "Survival").af.<br>**AND**<br>("Systematic Review" OR "Meta-Analysis").af.<br>(Limited to Embase Status)<br>Filter: English language	1,271
**Cochrane Library, search through 6 May 2023**	
All Text = ("Fatty Acids" OR "Saturated Fatty Acids" OR "Unsaturated Fatty Acids" OR "Omega-6 Fatty Acids" OR "Omega-3 Fatty Acids" OR "Polyunsaturated Fatty Acids" OR "Monounsaturated Fatty Acids" OR "Essential Fatty Acids" OR "Trans Fatty Acids" OR "Serum Fatty Acids" OR "Plasma Fatty Acids" OR "Blood Fatty Acids" OR "Circulating Fatty Acids" OR "Erythrocyte Fatty Acids" OR "Phospholipid Fatty Acids" OR "Cholesteryl Ester Fatty Acids" OR "Adipose Tissue Fatty Acids" OR "Dietary Fatty Acids" OR "Fatty Acids Intake" OR "Myristic Acid" OR "Palmitic Acid" OR "Stearic Acid" OR "Pentadecanoic Acid" OR "Margaric Acid" OR "Heptadecanoic Acid" OR "Arachidic Acid" OR "Behenic Acid" OR "Tricosanoic Acid" OR "Lignoceric Acid" OR "Caprylic Acid" OR "Capric Acid" OR "Lauric Acids" OR "Palmitoleic Acid" OR "Oleic Acid" OR "Gondoic Acid" OR "Nervonic Acid" OR "Myristoleic Acid" OR "Erucic Acid" OR "alpha-Linolenic Acid" OR "Eicosapentaenoic Acid" OR "Docosahexaenoic Acid" OR "Docosapentaenoic Acid" OR "Linoleic Acid" OR "Gamma-linolenic Acid" OR "Eicosadienoic Acid" OR "Dihomo-gamma-linolenic Acid" OR "Arachidonic Acid" OR "Adrenic Acid" OR "Osbond Acid" OR "Docosadienoic Acid" OR "Linolenic Acid" OR "Conjugated Linoleic Acid" OR "Elaidic Acid")<br>**AND**<br>All Text = ("Cardiovascular Diseases" OR "Cardiovascular Disease" OR "Cardiovascular Event" OR "Coronary disease" OR "Coronary heart disease" OR "Coronary artery disease" OR "Ischemic Heart Disease" OR "Ischaemic Heart Disease" OR "Myocardial ischaemia" OR "Myocardial ischemia" OR "Myocardial infarction" OR "Heart attack" OR "Heart failure" OR "Cardiocerebrovascular disease" OR "Cerebrovascular disease" OR "Stroke" OR "Cerebrovascular accident" OR "Cardiovascular mortality" OR "All-cause Mortality" OR "Mortality" OR "Survival")<br>**AND**<br>Cochrane Reviews	337

eResults: Tables

Supplementary eTable 3. Summary of baseline characteristics, as well as correlations with SFA, MUFA, and PUFA

Baseline characteristics	N of cohorts	Sample size	Overall statistics*	Correlation with FAs¶		
				SFA	MUFA	PUFA
**Conventional CVD risk factors included in the basic model**						
Women, n (%)	3	172,891	85,844 (54.6)	-0.021	-0.115	0.154
Age (years), mean (SD)	3	172,891	52.5 (11.6)	0.016	0.039	-0.060
Current smoking, n (%)	3	171,866	18,152 (11.6)	0.019	0.062	-0.090
History of hypertension, n (%)†	2	130,669	29,904 (26.0)	0.021	0.058	-0.109
History of diabetes, n (%)†	2	127,150	4,757 (4.2)	<0.001^	0.052	-0.072
Active physical activity, n (%)	3	152,749	109,512 (79.7)	-0.011	-0.045	0.064
**Other non-lipid conventional CVD risk factors that may be related to diet**						
Current alcohol consumption, n (%)	3	172,097	140,920 (90.1)	0.029	-0.011	-0.014
BMI (kg/m^2), mean (SD)	3	172,161	26.9 (4.8)	0.048	0.173	-0.247
**Dietary intakes**						
Intakes of fruit and vegetables (g/day), median (IQR)	1	31,489	372.6 (237.3, 550.9)	-0.041	0.014^	0.014^
Intakes of dairy products (g/day), median (IQR)	1	31,489	286.9 (167.6, 456.2)	0.023	-0.025	-0.007^
Intakes of meat (g/day), median (IQR)	1	31,489	102.8 (69.2, 143.0)	-0.007^	-0.043	0.054
Intakes of fish (g/day), median (IQR)	1	31,489	29.0 (14.9, 52.1)	0.002^	-0.023	0.031
Intakes of olive oil (g/day), median (IQR)	1	31,489	0.0 (0.0, 14.7)	-0.133	0.238	-0.121
Intakes of margarine (g/day), median (IQR)	1	31,489	4.9 (0.1, 21.7)	-0.022	-0.078	0.055
Intakes of alcoholic beverages (g/day), median (IQR)	1	31,489	85.0 (9.6, 255.0)	0.082	0.180	-0.192
**Lipids**						
Total cholesterol (mmol/l), mean (SD)	3	168,991	4.6 (1.0)	0.018	-0.008	-0.007
High-density lipoprotein cholesterol (mmol/l), mean (SD)	3	170,122	1.4 (0.3)	0.021	-0.203	0.214
Log_e(triglyceride) (mmol/l), mean (SD)	3	168,896	0.1 (0.4)	0.127	0.393	-0.573
**Inflammation and glucose**						
Log_e(C-reactive protein) (mg/l), mean (SD)	3	165,786	0.2 (1.1)	0.051	0.143	-0.214
HbA1C (%), mean (SD)	2	124,917	4.9 (0.9)	0.022	0.084	-0.147
Glucose (mmol/l), mean (SD)	2	140,885	3.3 (1.2)	0.050	0.129	-0.117

BMI: Body mass index; FAs: Fatty acids; IQR: Interquartile range; MUFA: Monounsaturated fatty acid; PUFA: Polyunsaturated fatty acid; SD: Standard deviation; SFA: Saturated fatty acid.

* Baseline characteristics of EPIC-CVD were summarized based on sub-cohort participants (n=15,889) given its case-cohort study design. The total number of EPIC-CVD participants (n=31,606) was the sum of subcohort and cases of total CVD, CHD, Stroke, and CVD mortality.

¶ Semipartial correlation coefficients, adjusted for sex, age, study, and batch (total energy intake was included in the adjustment for dietary variables). Analyses were conducted based on complete cases (for EPIC-CVD study, restricted to the sub-cohort).

^ P for correlation >0.05.

† Information on history of diabetes and history of hypertension was not reported in INTERVAL, but they were considered as generally healthy given participants were all blood donors.

Supplementary eTable 4. Detailed baseline FA concentrations of EPIC-CVD participants by subcohort and CVD status.

**Baseline characteristics**	**Total**	
	Subcohort	CVD
N	15,889	16,055
**FAs (mol%), Mean (SD)**		
**Total SFA**	**45.90 (1.21)**	**46.35 (1.14)**
**Even-chain SFA**	**44.58 (1.23)**	**45.07 (1.16)**
Myristic acid (14:0)	0.37 (0.11)	0.39 (0.10)
Palmitic acid (16:0)	30.09 (1.68)	30.48 (1.61)
Stearic acid (18:0)	14.11 (1.34)	14.20 (1.23)
**Odd-chain SFA**	**0.63 (0.13)**	**0.59 (0.13)**
Pentadecanoic acid (15:0)	0.21 (0.07)	0.20 (0.06)
Heptadecanoic acid (17:0)	0.41 (0.09)	0.39 (0.09)
**Longer-chain SFA**	**0.70 (0.21)**	**0.69 (0.17)**
Arachidic acid (20:0)	0.13 (0.04)	0.13 (0.04)
Behenic acid (22:0)	0.23 (0.08)	0.23 (0.07)
Tricosanoic acid (23:0)	0.11 (0.05)	0.10 (0.03)
Lignoceric acid (24:0)	0.23 (0.07)	0.22 (0.06)
**Total MUFA**	**11.02 (1.92)**	**11.12 (1.94)**
Palmitoleic acid (16:1)	0.51 (0.26)	0.58 (0.29)
Heptadecenoic acid (17:1)	0.07 (0.06)	0.06 (0.04)
Oleic acid (18:1)	9.85 (1.76)	9.89 (1.75)
Eicosenoic acid (20:1)	0.24 (0.07)	0.24 (0.08)
Nervonic acid (24:1)	0.35 (0.11)	0.35 (0.11)
**Total PUFA**	**42.64 (2.14)**	**42.06 (2.23)**
**n-3 PUFA**	**6.71 (1.95)**	**6.96 (2.09)**
Alpha-linolenic acid (ALA; 18:3n3)	0.31 (0.17)	0.30 (0.14)
**Long-chain n-3 PUFA (EPA+ n3-DPA+ DHA)**	**6.40 (1.94)**	**6.66 (2.08)**
Eicosapentaenoic acid (EPA; 20:5n3)	1.23 (0.80)	1.40 (0.88)
Docosapentaenoic acid (n3-DPA; 22:5n3)	0.89 (0.24)	0.96 (0.22)
Docosahexaenoic acid (DHA; 22:6n3)	4.27 (1.24)	4.30 (1.29)
**Omega-3 index (EPA+DHA)**	5.50 (1.83)	5.70 (1.97)
**n-6 PUFA**	**35.93 (2.94)**	**35.11 (3.10)**
Linoleic acid (LA; 18:2n6)	22.61 (3.17)	22.17 (3.15)
Gamma linolenic acid (GLA; 18:3n6)	0.08 (0.05)	0.09 (0.05)
Eicosadienoic acid (EDA; 20:2)	0.38 (0.07)	0.38 (0.09)
Dihomo-gamma-linolenic acid (DGLA; 20:3n6)	3.14 (0.78)	3.17 (0.76)
Docosatetraenoic (DTA; 22:4n6)	0.29 (0.08)	0.28 (0.08)
Docosapentenoic acid (n6-DPA; 22:5n6)	0.19 (0.09)	0.17 (0.08)
Arachidonic acid (AA; 20:4n6)	9.25 (1.81)	8.84 (1.75)
**trans-FA**	0.35 (0.19)	0.40 (0.21)
Elaidic acid (18:1n9t)	0.25 (0.18)	0.27 (0.18)
Conjugated linoleic acid (CLA; 18:2n6t)	0.07 (0.03)	0.07 (0.03)
trans-palmitoleic acid (TPA; 16:1t) *	0.06 (0.03)	0.07 (0.03)
**FA ratios**		
Ratio of 16:1 to 16:0	0.02 (0.01)	0.02 (0.01)
Ratio of 18:1n9c to 18:0	0.71 (0.16)	0.71 (0.16)
Ratio of 18:3n6 to 18:2n6	0.00 (0.00)	0.00 (0.01)
Ratio of 20:3n6 to 18:2n6	0.14 (0.05)	0.15 (0.59)
Ratio of 20:4n6 to 20:3n6	3.18 (6.23)	2.93 (0.85)
Ratio of n-6 to n-3 PUFA	5.87 (1.97)	5.58 (2.01)
Ratio of PUFA to MUFA	4.02 (0.88)	3.92 (0.85)

CVD: Cardiovascular disease; FA: Fatty acid; MUFA: Monounsaturated fatty acid; PUFA: Polyunsaturated fatty acid; SD: Standard deviation; SFA: Saturated fatty acid.
* Among 9,597 sub-cohort participants and 13,261 CVD cases.

Supplementary eTable 5. Hazard ratios per 1-SD higher FAs for cardiovascular events in primary analysis (estimated in 172,891 participants from EPIC-CVD, UKB and INTERVAL) and sensitivity analysis 1 (excluding follow-up within 2 years from baseline)

FAs (%)	CHD		Stroke		Ischaemic stroke		Haemorrhagic stroke		Total CVD		CVD mortality	
	HR (95%CI)	*P*	HR (95%CI)	*P*	HR (95%CI)	*P*	HR (95%CI)	*P*	HR (95%CI)	*P*	HR (95%CI)	*P*
**Primary analysis: estimated among 172,891 participants from EPIC-CVD, UKB and INTERVAL**												
	**9,453 cases**		**8,182 cases**		**4,935 cases**		**1,603 cases**		**20,130 cases**		**3,674 cases**	
Total SFA	1.17 (1.09, 1.27)	<0.001	1.13 (1.04, 1.22)	0.002	1.16 (1.05, 1.28)	0.004	0.99 (0.81, 1.20)	0.887	1.16 (1.08, 1.25)	<0.001	1.14 (1.00, 1.28)	0.042
Total MUFA	1.01 (0.90, 1.13)	0.913	1.06 (0.98, 1.14)	0.170	1.01 (0.91, 1.12)	0.856	1.11 (0.93, 1.33)	0.243	1.02 (0.94, 1.11)	0.580	1.14 (1.05, 1.22)	0.001
Total PUFA	0.93 (0.87, 1.00)	0.038	0.91 (0.84, 1.00)	0.048	0.94 (0.85, 1.04)	0.216	0.93 (0.78, 1.11)	0.401	0.92 (0.86, 0.98)	0.010	0.87 (0.82, 0.91)	<0.001
n-3 PUFA	0.91 (0.85, 0.97)	0.007	0.97 (0.90, 1.05)	0.448	0.98 (0.87, 1.10)	0.680	1.03 (0.85, 1.25)	0.737	0.95 (0.89, 1.01)	0.113	1.02 (0.86, 1.22)	0.812
DHA	0.91 (0.84, 0.98)	0.016	0.96 (0.90, 1.03)	0.296	0.97 (0.86, 1.09)	0.606	1.07 (0.87, 1.32)	0.505	0.95 (0.89, 1.02)	0.168	0.99 (0.84, 1.16)	0.914
n-6 PUFA	0.98 (0.90, 1.07)	0.659	0.94 (0.91, 0.98)	0.004	0.95 (0.91, 1.00)	0.052	0.94 (0.86, 1.02)	0.130	0.94 (0.90, 0.99)	0.023	0.91 (0.87, 0.97)	0.001
LA	0.95 (0.90, 1.00)	0.039	0.89 (0.83, 0.95)	0.001	0.90 (0.84, 0.96)	0.003	0.98 (0.86, 1.11)	0.728	0.89 (0.83, 0.95)	<0.001	0.89 (0.82, 0.97)	0.007
Ratio of PUFA to MUFA	0.98 (0.87, 1.10)	0.753	0.95 (0.86, 1.06)	0.362	1.00 (0.87, 1.13)	0.947	0.94 (0.81, 1.10)	0.454	0.97 (0.88, 1.07)	0.530	0.86 (0.81, 0.92)	<0.001
Ratio of n-6 to n-3 PUFA	1.05 (0.97, 1.14)	0.225	1.01 (0.94, 1.08)	0.829	1.02 (0.90, 1.15)	0.776	0.99 (0.85, 1.15)	0.881	1.03 (0.98, 1.09)	0.267	0.99 (0.86, 1.13)	0.837
**Sensitivity analysis: excluding follow-up within 2 years from baseline**												
	**8,478 cases**		**7,509 cases**		**4,564 cases**		**1,444 cases**		**18,210 cases**		**3,360 cases**	
Total SFA	1.17 (1.09, 1.27)	<0.001	1.12 (1.04, 1.21)	0.003	1.15 (1.04, 1.26)	0.005	1.02 (0.83, 1.24)	0.866	1.16 (1.08, 1.25)	<0.001	1.14 (1.00, 1.30)	0.054
Total MUFA	1.03 (0.93, 1.14)	0.582	1.05 (0.96, 1.15)	0.290	1.01 (0.90, 1.12)	0.900	1.09 (0.91, 1.30)	0.364	1.03 (0.95, 1.12)	0.514	1.16 (1.10, 1.22)	<0.001
Total PUFA	0.92 (0.86, 0.97)	0.002	0.92 (0.83, 1.02)	0.106	0.94 (0.85, 1.06)	0.318	0.96 (0.80, 1.14)	0.632	0.92 (0.86, 0.98)	0.013	0.87 (0.83, 0.92)	<0.001
n-3 PUFA	0.92 (0.85, 0.98)	0.014	0.96 (0.88, 1.04)	0.301	0.96 (0.86, 1.08)	0.512	0.99 (0.81, 1.21)	0.928	0.95 (0.89, 1.01)	0.107	1.02 (0.85, 1.22)	0.838
DHA	0.91 (0.84, 0.99)	0.026	0.97 (0.89, 1.06)	0.532	0.96 (0.86, 1.08)	0.539	1.01 (0.83, 1.23)	0.913	0.95 (0.89, 1.02)	0.140	1.00 (0.84, 1.18)	0.991
n-6 PUFA	0.96 (0.89, 1.04)	0.369	0.96 (0.92, 1.00)	0.035	0.96 (0.91, 1.01)	0.131	0.97 (0.88, 1.07)	0.529	0.94 (0.89, 0.99)	0.022	0.92 (0.86, 0.98)	0.013
LA	0.94 (0.90, 0.99)	0.013	0.89 (0.83, 0.96)	0.003	0.90 (0.83, 0.98)	0.013	1.03 (0.87, 1.22)	0.720	0.88 (0.83, 0.94)	<0.001	0.90 (0.83, 0.97)	0.006
Ratio of PUFA to MUFA	0.96 (0.86, 1.07)	0.446	0.96 (0.85, 1.08)	0.466	1.00 (0.87, 1.15)	0.996	0.98 (0.86, 1.13)	0.801	0.96 (0.88, 1.06)	0.467	0.85 (0.81, 0.91)	<0.001
Ratio of n-6 to n-3 PUFA	1.03 (0.95, 1.12)	0.473	1.02 (0.95, 1.10)	0.518	1.02 (0.92, 1.13)	0.734	1.04 (0.88, 1.24)	0.647	1.04 (0.98, 1.10)	0.209	0.98 (0.84, 1.15)	0.806

CHD: Coronary heart disease; CI: Confidence interval; CVD: Cardiovascular disease; DHA: Docosahexaenoic acid; FA: Fatty acid; HR: Hazard ratio; LA: Linoleic acid; MUFA: Monounsaturated fatty acid; PUFA: Polyunsaturated fatty acid; SD: Standard deviation; SFA: Saturated fatty acid.

Results are pooled HRs (95% CI) per 1-SD higher FAs using random-effects meta-analysis, adjusted for batch (EPIC-CVD only), age, smoking, history of diabetes, history of hypertension, and physical activity, stratified by center (EPIC-CVD only) and sex.

Supplementary eTable 6. Hazard ratios per 1-SD higher FAs for cardiovascular events, with further adjustments estimated in 167,620 participants from EPIC-CVD, UKB and INTERVAL.

FAs (%) \ Adjustments*	CHD (8,424 cases)		Stroke (6,380 cases)		Ischaemic stroke (3,826 cases)		Haemorrhagic stroke (1,277 cases)		Total CVD (17,368 cases)		CVD mortality (3,237 cases)	
	HR (95%CI)	*P*	HR (95%CI)	*P*	HR (95%CI)	*P*	HR (95%CI)	*P*	HR (95%CI)	*P*	HR (95%CI)	*P*
**Total SFA**												
Adjusted for age, sex, and lifestyle factors	1.18 (1.09, 1.28)	<0.001	1.16 (1.07, 1.26)	<0.001	1.19 (1.07, 1.31)	0.001	1.05 (0.85, 1.31)	0.647	1.18 (1.10, 1.28)	<0.001	1.12 (0.99, 1.28)	0.075
Plus alcohol consumption and BMI	1.14 (1.06, 1.24)	0.001	1.11 (1.04, 1.18)	0.001	1.13 (1.04, 1.23)	0.005	1.08 (0.88, 1.32)	0.455	1.14 (1.07, 1.22)	<0.001	1.10 (0.96, 1.26)	0.160
Plus lipid markers	1.07 (1.02, 1.13)	0.012	1.11 (1.06, 1.16)	<0.001	1.11 (1.05, 1.17)	<0.001	1.10 (0.89, 1.36)	0.391	1.09 (1.05, 1.14)	<0.001	1.10 (0.97, 1.24)	0.144
**Total MUFA**												
Adjusted for age, sex, and lifestyle factors	1.03 (0.92, 1.14)	0.635	1.06 (0.98, 1.15)	0.164	1.02 (0.92, 1.13)	0.718	1.11 (0.93, 1.33)	0.25	1.03 (0.95, 1.12)	0.445	1.13 (1.05, 1.22)	0.001
Plus alcohol consumption and BMI	1.04 (0.94, 1.14)	0.458	1.06 (0.98, 1.15)	0.145	1.02 (0.94, 1.11)	0.596	1.09 (0.90, 1.32)	0.377	1.05 (0.98, 1.11)	0.149	1.10 (1.04, 1.16)	0.001
Plus lipid markers	1.03 (0.92, 1.16)	0.578	1.08 (0.97, 1.20)	0.161	1.01 (0.89, 1.15)	0.898	1.12 (0.92, 1.35)	0.265	1.05 (0.95, 1.16)	0.341	1.15 (0.97, 1.36)	0.109
**Total PUFA**												
Adjusted for age, sex, and lifestyle factors	0.91 (0.86, 0.97)	0.002	0.90 (0.82, 0.98)	0.013	0.92 (0.85, 1.00)	0.055	0.90 (0.75, 1.08)	0.272	0.90 (0.85, 0.95)	<0.001	0.87 (0.83, 0.91)	<0.001
Plus alcohol consumption and BMI	0.91 (0.87, 0.96)	<0.001	0.91 (0.83, 0.99)	0.028	0.94 (0.87, 1.02)	0.149	0.92 (0.76, 1.12)	0.403	0.91 (0.87, 0.96)	<0.001	0.91 (0.86, 0.96)	0.001
Plus lipid markers	0.94 (0.87, 1.02)	0.156	0.89 (0.80, 1.00)	0.051	0.94 (0.83, 1.07)	0.335	0.90 (0.72, 1.11)	0.318	0.93 (0.85, 1.01)	0.093	0.86 (0.74, 1.01)	0.062
**n-3 PUFA**												
Adjusted for age, sex, and lifestyle factors	0.90 (0.84, 0.97)	0.004	0.98 (0.89, 1.09)	0.727	1.00 (0.87, 1.14)	1.000	1.06 (0.88, 1.29)	0.535	0.96 (0.89, 1.03)	0.230	1.00 (0.85, 1.19)	0.984
Plus alcohol consumption and BMI	0.89 (0.84, 0.94)	<0.001	0.99 (0.90, 1.08)	0.758	1.01 (0.89, 1.14)	0.889	1.05 (0.88, 1.27)	0.577	0.96 (0.90, 1.03)	0.267	1.03 (0.87, 1.22)	0.752
Plus lipid markers	0.89 (0.85, 0.94)	<0.001	1.00 (0.91, 1.10)	0.976	1.02 (0.90, 1.17)	0.708	1.08 (0.89, 1.32)	0.425	0.98 (0.91, 1.05)	0.576	1.06 (0.89, 1.28)	0.502
or plus n-6 PUFA	0.90 (0.83, 0.97)	0.006	0.92 (0.80, 1.06)	0.264	0.97 (0.83, 1.14)	0.715	0.96 (0.75, 1.24)	0.768	0.92 (0.85, 1.00)	0.058	0.91 (0.78, 1.05)	0.199
**DHA**												
Adjusted for age, sex, and lifestyle factors	0.90 (0.83, 0.98)	0.011	0.98 (0.89, 1.08)	0.719	0.99 (0.86, 1.15)	0.898	1.11 (0.90, 1.37)	0.319	0.96 (0.89, 1.04)	0.321	0.97 (0.83, 1.14)	0.731
Plus alcohol consumption and BMI	0.89 (0.83, 0.95)	0.001	0.99 (0.91, 1.08)	0.812	1.00 (0.88, 1.14)	0.983	1.12 (0.91, 1.36)	0.289	0.97 (0.90, 1.04)	0.393	1.00 (0.86, 1.16)	0.986
Plus lipid markers	0.88 (0.84, 0.93)	<0.001	1.00 (0.90, 1.10)	0.956	1.01 (0.87, 1.17)	0.905	1.16 (0.92, 1.46)	0.210	0.98 (0.91, 1.05)	0.537	1.03 (0.87, 1.22)	0.731
or plus n-6 PUFA	0.89 (0.83, 0.95)	0.001	0.95 (0.84, 1.07)	0.372	0.98 (0.84, 1.14)	0.773	1.06 (0.83, 1.36)	0.639	0.94 (0.88, 1.02)	0.143	0.91 (0.81, 1.03)	0.126
**n-6 PUFA**												
Adjusted for age, sex, and lifestyle factors	0.97 (0.89, 1.05)	0.482	0.93 (0.89, 0.97)	<0.001	0.93 (0.89, 0.98)	0.005	0.87 (0.76, 0.99)	0.040	0.93 (0.88, 0.97)	0.002	0.91 (0.84, 0.99)	0.020
Plus alcohol consumption and BMI	0.98 (0.90, 1.06)	0.637	0.95 (0.91, 0.99)	0.008	0.96 (0.91, 1.01)	0.110	0.90 (0.81, 0.99)	0.038	0.94 (0.92, 0.97)	<0.001	0.95 (0.88, 1.02)	0.140
Plus lipid markers	1.02 (0.94, 1.11)	0.567	0.88 (0.83, 0.93)	<0.001	0.92 (0.85, 0.98)	0.016	0.80 (0.70, 0.91)	0.001	0.94 (0.88, 1.01)	0.078	0.88 (0.78, 0.99)	0.032
or plus n-3 PUFA	0.91 (0.88, 0.95)	<0.001	0.87 (0.78, 0.98)	0.017	0.92 (0.83, 1.02)	0.110	0.87 (0.70, 1.08)	0.215	0.90 (0.85, 0.96)	<0.001	0.94 (0.88, 1.00)	0.064
**LA**												
Adjusted for age, sex, and lifestyle factors	0.95 (0.90, 1.00)	0.033	0.86 (0.79, 0.93)	<0.001	0.85 (0.77, 0.94)	0.001	0.94 (0.80, 1.10)	0.432	0.88 (0.82, 0.94)	<0.001	0.89 (0.83, 0.96)	0.003
Plus alcohol consumption and BMI	0.98 (0.93, 1.03)	0.426	0.89 (0.83, 0.95)	0.001	0.89 (0.82, 0.97)	0.007	0.92 (0.82, 1.04)	0.187	0.91 (0.86, 0.96)	0.001	0.93 (0.85, 1.01)	0.068
Plus lipid markers	0.99 (0.94, 1.04)	0.625	0.89 (0.85, 0.93)	<0.001	0.90 (0.85, 0.96)	0.001	0.90 (0.81, 0.99)	0.026	0.92 (0.87, 0.96)	0.001	0.90 (0.82, 0.98)	0.020
or plus n-3 PUFA	0.95 (0.91, 0.99)	0.013	0.85 (0.77, 0.93)	0.001	0.86 (0.77, 0.96)	0.007	0.99 (0.76, 1.30)	0.963	0.88 (0.82, 0.94)	<0.001	0.91 (0.86, 0.96)	0.001
**Ratio of PUFA to MUFA**												
Adjusted for age, sex, and lifestyle factors	0.96 (0.86, 1.07)	0.439	0.94 (0.85, 1.05)	0.287	0.98 (0.87, 1.12)	0.800	0.94 (0.80, 1.09)	0.393	0.96 (0.87, 1.05)	0.348	0.86 (0.81, 0.91)	<0.001
Plus alcohol consumption and BMI	0.96 (0.87, 1.07)	0.492	0.94 (0.85, 1.04)	0.243	0.98 (0.88, 1.10)	0.753	0.94 (0.79, 1.11)	0.455	0.95 (0.88, 1.03)	0.193	0.90 (0.85, 0.96)	0.001
Plus lipid markers	0.97 (0.86, 1.10)	0.640	0.92 (0.79, 1.07)	0.293	0.98 (0.82, 1.16)	0.778	0.89 (0.74, 1.08)	0.235	0.94 (0.83, 1.07)	0.372	0.85 (0.69, 1.06)	0.152
**Ratio of n-6 to n-3 PUFA**												
Adjusted for age, sex, and lifestyle factors	1.05 (0.97, 1.14)	0.218	0.98 (0.89, 1.08)	0.717	0.98 (0.85, 1.13)	0.800	0.93 (0.77, 1.12)	0.426	1.02 (0.96, 1.09)	0.458	0.99 (0.86, 1.14)	0.908
Plus alcohol consumption and BMI	1.07 (1.00, 1.15)	0.062	0.98 (0.90, 1.07)	0.659	0.98 (0.86, 1.12)	0.778	0.95 (0.81, 1.13)	0.583	1.02 (0.97, 1.08)	0.447	0.98 (0.85, 1.13)	0.806
Plus lipid markers	1.09 (1.02, 1.16)	0.006	0.97 (0.89, 1.07)	0.549	0.98 (0.85, 1.12)	0.737	0.94 (0.79, 1.13)	0.517	1.02 (0.96, 1.09)	0.480	0.95 (0.82, 1.11)	0.539

BMI: Body mass index; CHD: Coronary heart disease; CI: Confidence interval; CVD: Cardiovascular disease; DHA: Docosahexaenoic acid; FA: Fatty acid; HR: Hazard ratio; LA: Linoleic acid; MUFA: Monounsaturated fatty acid; PUFA: Polyunsaturated fatty acid; SD: Standard deviation; SFA: Saturated fatty acid.

Results are pooled HRs (95% CI) per 1-SD higher FAs using random-effects meta-analysis, estimated on a complete-case basis (n=167,620) due to missing data of potential confounders or mediators.

* Adjustments: i) basic adjustment for age, sex and lifestyle factors consists of adjustment for batch (EPIC-CVD only), age, smoking, history of diabetes, history of hypertension, and physical activity, stratified by center (EPIC-CVD only) and sex; ii) plus alcohol consumption and BMI; and iii) plus lipid markers (i.e., total cholesterol, high-density-lipoprotein cholesterol, loge triglycerides) or plus other FAs (for PUFA subtypes only), respectively.

Supplementary eTable 7. Associations of plasma phospholipids total SFA, SFA subtypes and individual SFAs with CHD and stroke, with further adjustments estimated from EPIC-CVD participants (subcohort n=15,125).

FAs (%)	Adjustments*	CHD (6,341 cases)		Stroke (4,719 cases)	
		HR (95%CI)	*P*	HR (95%CI)	*P*
**Total SFA**	**Adjusted for age, sex, and lifestyle factors**	**1.22 (1.16, 1.29)**	**<0.001**	**1.22 (1.11, 1.34)**	**<0.001**
	**Plus alcohol consumption and BMI**	**1.20 (1.12, 1.27)**	**<0.001**	**1.16 (1.06, 1.26)**	**0.001**
	**Plus dietary intakes**	**1.22 (1.13, 1.32)**	**<0.001**	**1.17 (1.09, 1.25)**	**<0.001**
	**Plus lipid markers**	**1.11 (1.05, 1.19)**	**0.001**	**1.14 (1.06, 1.23)**	**<0.001**
**Even-chain SFA**	**Adjusted for age, sex, and lifestyle factors**	**1.26 (1.19, 1.32)**	**<0.001**	**1.28 (1.16, 1.42)**	**<0.001**
	**Plus alcohol consumption and BMI**	**1.23 (1.16, 1.29)**	**<0.001**	**1.22 (1.11, 1.34)**	**<0.001**
	**Plus dietary intakes**	**1.27 (1.18, 1.36)**	**<0.001**	**1.23 (1.14, 1.33)**	**<0.001**
	**Plus lipid markers**	**1.17 (1.10, 1.24)**	**<0.001**	**1.18 (1.10, 1.27)**	**<0.001**
Myristic acid (14:0)	Adjusted for age, sex, and lifestyle factors	1.11 (1.02, 1.20)	0.013	1.03 (0.91, 1.16)	0.639
	Plus alcohol consumption and BMI	1.10 (1.02, 1.19)	0.016	1.02 (0.91, 1.14)	0.737
	Plus dietary intakes	1.12 (1.04, 1.20)	0.002	1.04 (0.94, 1.17)	0.428
	Plus lipid markers	1.08 (0.99, 1.17)	0.092	1.01 (0.92, 1.12)	0.792
Palmitic acid (16:0)	Adjusted for age, sex, and lifestyle factors	1.07 (0.95, 1.20)	0.275	1.20 (1.11, 1.29)	<0.001
	Plus alcohol consumption and BMI	1.09 (0.99, 1.21)	0.080	1.19 (1.11, 1.28)	<0.001
	Plus dietary intakes	1.08 (0.95, 1.24)	0.242	1.21 (1.12, 1.30)	<0.001
	Plus lipid markers	1.06 (0.93, 1.22)	0.374	1.19 (1.10, 1.28)	<0.001
Stearic acid (18:0)	Adjusted for age, sex, and lifestyle factors	1.20 (1.05, 1.38)	0.007	1.00 (0.92, 1.08)	0.973
	Plus alcohol consumption and BMI	1.11 (0.98, 1.25)	0.115	0.95 (0.88, 1.03)	0.222
	Plus dietary intakes	1.12 (0.99, 1.27)	0.081	0.96 (0.89, 1.04)	0.283
	Plus lipid markers	1.03 (0.92, 1.15)	0.595	0.95 (0.88, 1.02)	0.150
**Odd-chain SFA (c150 c170)**	**Adjusted for age, sex, and lifestyle factors**	**0.81 (0.75, 0.88)**	**<0.001**	**0.73 (0.67, 0.79)**	**<0.001**
	**Plus alcohol consumption and BMI**	**0.81 (0.77, 0.86)**	**<0.001**	**0.75 (0.69, 0.81)**	**<0.001**
	**Plus dietary intakes**	**0.79 (0.72, 0.87)**	**<0.001**	**0.76 (0.70, 0.83)**	**<0.001**
	**Plus lipid markers**	**0.83 (0.75, 0.92)**	**<0.001**	**0.77 (0.71, 0.84)**	**<0.001**
**Odd-chain SFA (c150 c170 c230)**	**Adjusted for age, sex, and lifestyle factors**	**0.82 (0.75, 0.90)**	**<0.001**	**0.73 (0.68, 0.80)**	**<0.001**
	**Plus alcohol consumption and BMI**	**0.82 (0.77, 0.88)**	**<0.001**	**0.76 (0.70, 0.82)**	**<0.001**
	**Plus dietary intakes**	**0.80 (0.72, 0.89)**	**<0.001**	**0.77 (0.69, 0.85)**	**<0.001**
	**Plus lipid markers**	**0.83 (0.74, 0.94)**	**0.002**	**0.78 (0.71, 0.86)**	**<0.001**
Pentadecanoic acid (15:0)	Adjusted for age, sex, and lifestyle factors	0.87 (0.81, 0.93)	<0.001	0.77 (0.66, 0.89)	0.001
	Plus alcohol consumption and BMI	0.87 (0.81, 0.93)	<0.001	0.78 (0.67, 0.91)	0.001
	Plus dietary intakes	0.87 (0.79, 0.96)	0.007	0.82 (0.69, 0.98)	0.029
	Plus lipid markers	0.90 (0.81, 0.99)	0.026	0.83 (0.69, 1.00)	0.047
Heptadecanoic acid (17:0)	Adjusted for age, sex, and lifestyle factors	0.81 (0.73, 0.89)	<0.001	0.76 (0.69, 0.83)	<0.001
	Plus alcohol consumption and BMI	0.82 (0.76, 0.88)	<0.001	0.78 (0.71, 0.85)	<0.001
	Plus dietary intakes	0.79 (0.70, 0.88)	<0.001	0.77 (0.71, 0.84)	<0.001
	Plus lipid markers	0.82 (0.73, 0.93)	0.001	0.79 (0.72, 0.87)	<0.001
**Longer-chain SFA (c200 c220 c230 c240)**	**Adjusted for age, sex, and lifestyle factors**	**0.96 (0.81, 1.14)**	**0.629**	**0.83 (0.71, 0.97)**	**0.015**
	**Plus alcohol consumption and BMI**	**0.95 (0.81, 1.11)**	**0.511**	**0.84 (0.72, 0.98)**	**0.023**
	**Plus dietary intakes**	**0.95 (0.79, 1.13)**	**0.555**	**0.85 (0.73, 0.98)**	**0.028**
	**Plus lipid markers**	**0.94 (0.83, 1.06)**	**0.299**	**0.89 (0.78, 1.02)**	**0.083**
**Longer-chain SFA (c200 c220 c240)**	**Adjusted for age, sex, and lifestyle factors**	**0.95 (0.79, 1.13)**	**0.547**	**0.83 (0.71, 0.96)**	**0.013**
	**Plus alcohol consumption and BMI**	**0.94 (0.80, 1.10)**	**0.414**	**0.83 (0.72, 0.97)**	**0.020**
	**Plus dietary intakes**	**0.93 (0.78, 1.12)**	**0.455**	**0.85 (0.73, 0.98)**	**0.026**
	**Plus lipid markers**	**0.93 (0.82, 1.06)**	**0.284**	**0.90 (0.79, 1.02)**	**0.088**
Arachidic acid (20:0)	Adjusted for age, sex, and lifestyle factors	0.84 (0.74, 0.96)	0.008	0.85 (0.75, 0.96)	0.009
	Plus alcohol consumption and BMI	0.83 (0.74, 0.94)	0.002	0.86 (0.76, 0.98)	0.020
	Plus dietary intakes	0.82 (0.73, 0.91)	<0.001	0.86 (0.76, 0.98)	0.024
	Plus lipid markers	0.85 (0.76, 0.94)	0.002	0.89 (0.79, 1.00)	0.058
Behenic acid (22:0)	Adjusted for age, sex, and lifestyle factors	1.03 (0.87, 1.22)	0.730	0.86 (0.79, 0.95)	0.003
	Plus alcohol consumption and BMI	1.02 (0.86, 1.20)	0.833	0.87 (0.79, 0.96)	0.005
	Plus dietary intakes	1.01 (0.84, 1.21)	0.890	0.89 (0.81, 0.98)	0.015
	Plus lipid markers	0.99 (0.86, 1.12)	0.828	0.93 (0.86, 1.01)	0.080
Tricosanoic acid (23:0)	Adjusted for age, sex, and lifestyle factors	0.98 (0.86, 1.11)	0.706	0.91 (0.83, 1.00)	0.055
	Plus alcohol consumption and BMI	0.99 (0.86, 1.13)	0.837	0.91 (0.83, 1.01)	0.066
	Plus dietary intakes	0.98 (0.85, 1.12)	0.721	0.93 (0.86, 1.01)	0.100
	Plus lipid markers	0.98 (0.86, 1.11)	0.740	0.95 (0.88, 1.03)	0.215
Lignoceric acid (24:0)	Adjusted for age, sex, and lifestyle factors	0.94 (0.83, 1.06)	0.292	0.86 (0.74, 1.00)	0.052
	Plus alcohol consumption and BMI	0.96 (0.87, 1.06)	0.414	0.88 (0.76, 1.01)	0.063
	Plus dietary intakes	0.95 (0.85, 1.07)	0.404	0.89 (0.78, 1.01)	0.073
	Plus lipid markers	0.97 (0.91, 1.05)	0.481	0.92 (0.81, 1.04)	0.194

BMI: Body mass index; CHD: Coronary heart disease; CI: Confidence interval; FA: Fatty acid; HR: Hazard ratio; SD: Standard deviation; SFA: Saturated fatty acid.

Results are pooled HRs (95% CI) per 1-SD higher fatty acid concentrations using random-effects meta-analysis, estimated on a complete-case basis within EPIC-CVD (subcohort n=15,125) due to missing data of potential confounders or mediators.

* The basic adjustment included adjustment for batch, age, smoking, history of diabetes, history of hypertension, and physical activity, stratified by center and sex; then, plus alcohol consumption (current, non-current) and BMI; then, plus intakes of fruit and vegetables, dairy products, meat, fish, olive oil, margarine, and alcoholic beverages (g/day); and finally, plus total cholesterol, high-density-lipoprotein cholesterol, loge triglycerides.

Supplementary eTable 8. Associations of plasma phospholipids total and individual MUFAs with CHD and stroke, with further adjustments estimated from EPIC-CVD participants (subcohort n=15,125).

FAs (%)	Adjustments*	CHD (6,341 cases)		Stroke (4,719 cases)	
		HR (95%CI)	*P*	HR (95%CI)	*P*
**Total MUFA**	**Adjusted for age, sex, and lifestyle factors**	**0.96 (0.90, 1.03)**	**0.302**	**1.03 (0.90, 1.19)**	**0.634**
	**Plus alcohol consumption and BMI**	**0.98 (0.91, 1.05)**	**0.574**	**1.05 (0.93, 1.20)**	**0.416**
	**Plus dietary intakes**	**0.98 (0.90, 1.08)**	**0.724**	**1.08 (0.96, 1.21)**	**0.220**
	**Plus lipid markers**	**0.98 (0.88, 1.10)**	**0.790**	**1.07 (0.95, 1.19)**	**0.250**
Palmitoleic acid (16:1)	Adjusted for age, sex, and lifestyle factors	1.06 (0.98, 1.14)	0.137	1.18 (1.09, 1.28)	<0.001
	Plus alcohol consumption and BMI	1.05 (0.98, 1.12)	0.201	1.17 (1.07, 1.28)	0.001
	Plus dietary intakes	1.07 (0.97, 1.18)	0.176	1.19 (1.08, 1.32)	0.001
	Plus lipid markers	1.06 (0.98, 1.15)	0.163	1.17 (1.07, 1.28)	0.001
Heptadecenoic acid (17:1)	Adjusted for age, sex, and lifestyle factors	0.96 (0.80, 1.16)	0.664	1.05 (0.95, 1.17)	0.348
	Plus alcohol consumption and BMI	0.95 (0.79, 1.15)	0.611	1.06 (0.95, 1.18)	0.292
	Plus dietary intakes	0.94 (0.78, 1.15)	0.562	1.06 (0.96, 1.16)	0.234
	Plus lipid markers	0.95 (0.77, 1.18)	0.657	1.06 (0.97, 1.15)	0.213
Oleic acid (18:1)	Adjusted for age, sex, and lifestyle factors	0.95 (0.88, 1.03)	0.233	1.01 (0.87, 1.18)	0.867
	Plus alcohol consumption and BMI	0.97 (0.89, 1.06)	0.537	1.04 (0.90, 1.20)	0.581
	Plus dietary intakes	0.97 (0.87, 1.09)	0.614	1.06 (0.94, 1.20)	0.362
	Plus lipid markers	0.97 (0.86, 1.10)	0.666	1.05 (0.93, 1.18)	0.431
Eicosenoic acid (20:1)	Adjusted for age, sex, and lifestyle factors	1.02 (0.91, 1.14)	0.730	0.93 (0.78, 1.12)	0.469
	Plus alcohol consumption and BMI	1.06 (0.95, 1.17)	0.295	0.95 (0.80, 1.15)	0.618
	Plus dietary intakes	1.04 (0.93, 1.16)	0.470	0.99 (0.83, 1.18)	0.928
	Plus lipid markers	1.07 (0.95, 1.20)	0.246	1.01 (0.85, 1.19)	0.931
Nervonic acid (24:1)	Adjusted for age, sex, and lifestyle factors	0.90 (0.79, 1.04)	0.144	0.90 (0.79, 1.03)	0.120
	Plus alcohol consumption and BMI	0.90 (0.80, 1.01)	0.086	0.91 (0.81, 1.02)	0.119
	Plus dietary intakes	0.89 (0.79, 1.00)	0.058	0.92 (0.83, 1.03)	0.145
	Plus lipid markers	0.96 (0.89, 1.05)	0.382	1.00 (0.93, 1.07)	0.938

BMI: Body mass index; CHD: Coronary heart disease; CI: Confidence interval; FA: Fatty acid; HR: Hazard ratio; MUFA: Monounsaturated fatty acid; SD: Standard deviation.

Results are pooled HRs (95% CI) per 1-SD higher fatty acid concentrations using random-effects meta-analysis, estimated on a complete-case basis within EPIC-CVD (subcohort n=15,125) due to missing data of potential confounders or mediators.

* The basic adjustment included adjustment for batch, age, smoking, history of diabetes, history of hypertension, and physical activity, stratified by center and sex; then, plus alcohol consumption (current, non-current) and BMI; then, plus intakes of fruit and vegetables, dairy products, meat, fish, olive oil, margarine, and alcoholic beverages (g/day); and finally, plus total cholesterol, high-density-lipoprotein cholesterol, loge triglycerides.

Supplementary eTable 9. Associations of plasma phospholipids total PUFA, PUFA subtypes and individual PUFAs with CHD and stroke, with further adjustments estimated from EPIC-CVD participants (subcohort n=15,125).

FAs (%)	Adjustments*	CHD (6,341 cases)		Stroke (4,719 cases)	
		HR (95%CI)	*P*	HR (95%CI)	*P*
**Total PUFA**	**Adjusted for age, sex, and lifestyle factors**	**0.95 (0.90, 1.01)**	**0.097**	**0.90 (0.78, 1.03)**	**0.128**
	**Plus alcohol consumption and BMI**	**0.95 (0.90, 1.01)**	**0.112**	**0.90 (0.79, 1.03)**	**0.131**
	**Plus dietary intakes**	**0.94 (0.86, 1.03)**	**0.178**	**0.88 (0.78, 0.99)**	**0.029**
	**Plus lipid markers**	**0.97 (0.88, 1.08)**	**0.578**	**0.90 (0.80, 1.00)**	**0.056**
**n-3 PUFA**	**Adjusted for age, sex, and lifestyle factors**	**0.94 (0.84, 1.05)**	**0.271**	**0.99 (0.85, 1.15)**	**0.879**
	**Plus alcohol consumption and BMI**	**0.92 (0.84, 1.02)**	**0.112**	**0.98 (0.86, 1.12)**	**0.820**
	**Plus dietary intakes**	**0.96 (0.86, 1.07)**	**0.430**	**0.98 (0.85, 1.13)**	**0.793**
	**Plus lipid markers**	**0.94 (0.84, 1.04)**	**0.196**	**1.00 (0.86, 1.15)**	**0.952**
Alpha-linolenic acid (ALA; 18:3n3)	Adjusted for age, sex, and lifestyle factors	0.93 (0.87, 1.00)	0.057	1.00 (0.92, 1.10)	0.918
	Plus alcohol consumption and BMI	0.94 (0.87, 1.02)	0.144	1.02 (0.94, 1.11)	0.651
	Plus dietary intakes	0.94 (0.88, 1.01)	0.103	1.01 (0.93, 1.09)	0.865
	Plus lipid markers	0.97 (0.90, 1.04)	0.434	1.02 (0.94, 1.11)	0.610
**Long-chain n-3 PUFA**	**Adjusted for age, sex, and lifestyle factors**	**0.94 (0.85, 1.04)**	**0.242**	**0.99 (0.84, 1.15)**	**0.873**
	**Plus alcohol consumption and BMI**	**0.92 (0.84, 1.02)**	**0.102**	**0.98 (0.86, 1.12)**	**0.800**
	**Plus dietary intakes**	**0.95 (0.86, 1.05)**	**0.286**	**0.98 (0.85, 1.13)**	**0.784**
	**Plus lipid markers**	**0.93 (0.85, 1.02)**	**0.144**	**0.99 (0.86, 1.15)**	**0.922**
Eicosapentaenoic acid (EPA; 20:5n3)	Adjusted for age, sex, and lifestyle factors	0.90 (0.79, 1.02)	0.091	0.99 (0.86, 1.15)	0.943
	Plus alcohol consumption and BMI	0.92 (0.82, 1.03)	0.169	0.99 (0.87, 1.12)	0.838
	Plus dietary intakes	0.96 (0.87, 1.05)	0.347	0.99 (0.87, 1.13)	0.887
	Plus lipid markers	0.99 (0.89, 1.10)	0.859	1.01 (0.89, 1.15)	0.887
Docosapentaenoic acid (n3-DPA; 22:5n3)	Adjusted for age, sex, and lifestyle factors	0.91 (0.84, 0.98)	0.017	0.90 (0.79, 1.03)	0.135
	Plus alcohol consumption and BMI	0.95 (0.88, 1.03)	0.243	0.92 (0.81, 1.04)	0.199
	Plus dietary intakes	0.96 (0.87, 1.06)	0.454	0.93 (0.81, 1.06)	0.280
	Plus lipid markers	0.92 (0.85, 0.99)	0.036	0.93 (0.81, 1.06)	0.258
Docosahexaenoic acid (DHA; 22:6n3)	Adjusted for age, sex, and lifestyle factors	0.95 (0.84, 1.07)	0.364	0.99 (0.85, 1.16)	0.914
	Plus alcohol consumption and BMI	0.93 (0.84, 1.03)	0.142	0.99 (0.86, 1.13)	0.860
	Plus dietary intakes	0.95 (0.85, 1.06)	0.376	0.98 (0.85, 1.14)	0.826
	Plus lipid markers	0.91 (0.83, 1.00)	0.061	0.99 (0.85, 1.15)	0.910
**Omega-3 index (EPA+DHA)**	**Adjusted for age, sex, and lifestyle factors**	**0.93 (0.84, 1.02)**	**0.126**	**0.99 (0.84, 1.17)**	**0.915**
	**Plus alcohol consumption and BMI**	**0.92 (0.84, 1.00)**	**0.055**	**0.99 (0.86, 1.13)**	**0.847**
	**Plus dietary intakes**	**0.94 (0.85, 1.03)**	**0.164**	**0.98 (0.84, 1.14)**	**0.829**
	**Plus lipid markers**	**0.93 (0.85, 1.02)**	**0.126**	**1.00 (0.86, 1.16)**	**0.978**
**n-6 PUFA**	**Adjusted for age, sex, and lifestyle factors**	**1.02 (0.93, 1.11)**	**0.679**	**0.90 (0.84, 0.97)**	**0.006**
	**Plus alcohol consumption and BMI**	**1.02 (0.93, 1.12)**	**0.629**	**0.92 (0.85, 0.98)**	**0.018**
	**Plus dietary intakes**	**0.98 (0.87, 1.10)**	**0.699**	**0.90 (0.83, 0.98)**	**0.010**
	**Plus lipid markers**	**1.02 (0.90, 1.15)**	**0.793**	**0.91 (0.84, 0.99)**	**0.020**
Linoleic acid (LA; 18:2n6)	Adjusted for age, sex, and lifestyle factors	0.94 (0.88, 1.00)	0.065	0.83 (0.76, 0.90)	<0.001
	Plus alcohol consumption and BMI	0.97 (0.90, 1.05)	0.437	0.86 (0.80, 0.92)	<0.001
	Plus dietary intakes	0.95 (0.86, 1.04)	0.278	0.85 (0.78, 0.91)	<0.001
	Plus lipid markers	1.01 (0.91, 1.11)	0.894	0.86 (0.80, 0.93)	<0.001
Gamma linolenic acid (GLA; 18:3n6)	Adjusted for age, sex, and lifestyle factors	1.10 (1.03, 1.18)	0.007	1.05 (0.95, 1.16)	0.326
	Plus alcohol consumption and BMI	1.06 (1.00, 1.12)	0.040	1.02 (0.94, 1.10)	0.684
	Plus dietary intakes	1.06 (0.98, 1.14)	0.140	1.01 (0.94, 1.09)	0.739
	Plus lipid markers	0.98 (0.89, 1.08)	0.719	0.96 (0.90, 1.03)	0.300
Eicosadienoic acid (EDA; 20:2n6)	Adjusted for age, sex, and lifestyle factors	1.02 (0.92, 1.12)	0.732	0.98 (0.89, 1.08)	0.642
	Plus alcohol consumption and BMI	0.99 (0.90, 1.10)	0.879	0.96 (0.87, 1.05)	0.336
	Plus dietary intakes	0.97 (0.88, 1.07)	0.585	0.96 (0.88, 1.05)	0.354
	Plus lipid markers	0.94 (0.85, 1.04)	0.261	0.96 (0.87, 1.05)	0.344
Dihomo-gamma-linolenic acid (DGLA; 20:3n6)	Adjusted for age, sex, and lifestyle factors	1.41 (1.29, 1.53)	<0.001	1.22 (1.12, 1.34)	<0.001
	Plus alcohol consumption and BMI	1.30 (1.22, 1.39)	<0.001	1.15 (1.06, 1.24)	0.001
	Plus dietary intakes	1.30 (1.21, 1.39)	<0.001	1.19 (1.10, 1.29)	<0.001
	Plus lipid markers	1.13 (1.02, 1.25)	0.017	1.12 (1.03, 1.22)	0.009
Docosatetraenoic acid (DTA; 22:4n6)	Adjusted for age, sex, and lifestyle factors	1.11 (1.04, 1.18)	0.001	1.09 (0.94, 1.27)	0.230
	Plus alcohol consumption and BMI	1.10 (1.03, 1.17)	0.003	1.10 (0.97, 1.26)	0.152
	Plus dietary intakes	1.08 (0.99, 1.18)	0.092	1.10 (0.98, 1.24)	0.118
	Plus lipid markers	1.06 (0.96, 1.16)	0.246	1.09 (0.97, 1.22)	0.151
Docosapentenoic acid (n6-DPA; 22:5n6)	Adjusted for age, sex, and lifestyle factors	1.11 (1.04, 1.19)	0.002	1.09 (0.94, 1.27)	0.270
	Plus alcohol consumption and BMI	1.09 (1.02, 1.18)	0.015	1.09 (0.95, 1.24)	0.212
	Plus dietary intakes	1.08 (1.01, 1.16)	0.034	1.10 (0.97, 1.25)	0.147
	Plus lipid markers	1.05 (0.97, 1.13)	0.268	1.07 (0.95, 1.20)	0.252
Arachidonic acid (AA; 20:4n6)	Adjusted for age, sex, and lifestyle factors	1.01 (0.94, 1.09)	0.753	1.13 (1.05, 1.22)	0.002
	Plus alcohol consumption and BMI	0.99 (0.91, 1.07)	0.782	1.10 (1.02, 1.19)	0.014
	Plus dietary intakes	0.96 (0.87, 1.05)	0.362	1.08 (1.00, 1.17)	0.052
	Plus lipid markers	0.98 (0.88, 1.09)	0.706	1.09 (1.01, 1.18)	0.026

BMI: Body mass index; CHD: Coronary heart disease; CI: Confidence interval; FA: Fatty acid; HR: Hazard ratio; PUFA: Polyunsaturated fatty acid; SD: Standard deviation. Results are pooled HRs (95% CI) per 1-SD higher fatty acid concentrations using random-effects meta-analysis, estimated on a complete-case basis within EPIC-CVD (subcohort n=15,125) due to missing data of potential confounders or mediators.

* The basic adjustment included adjustment for batch, age, smoking, history of diabetes, history of hypertension, and physical activity, stratified by center and sex; then, plus alcohol consumption (current, non-current) and BMI; then, plus intakes of fruit and vegetables, dairy products, meat, fish, olive oil, margarine, and alcoholic beverages (g/day); and finally, plus total cholesterol, high-density-lipoprotein cholesterol, loge triglycerides.

Supplementary eTable 10. Associations of plasma phospholipids total and individual TFAs with CHD and stroke, with further adjustments estimated from EPIC-CVD participants (subcohort n=15,125).

FAs (%)	Adjustments*	CHD (6,341 cases)		Stroke (4,719 cases)	
		HR (95%CI)	*P*	HR (95%CI)	*P*
**Total TFA**	**Adjusted for age, sex, and lifestyle factors**	**0.82 (0.74, 0.92)**	**0.001**	**0.82 (0.66, 1.02)**	**0.073**
	**Plus alcohol consumption and BMI**	**0.81 (0.74, 0.90)**	**<0.001**	**0.83 (0.68, 1.03)**	**0.088**
	**Plus dietary intakes**	**0.82 (0.72, 0.93)**	**0.002**	**0.84 (0.69, 1.02)**	**0.080**
	**Plus lipid markers**	**0.85 (0.73, 1.00)**	**0.057**	**0.85 (0.70, 1.03)**	**0.103**
Elaidic acid (18:1n9t)	Adjusted for age, sex, and lifestyle factors	0.87 (0.81, 0.94)	0.001	0.84 (0.68, 1.04)	0.117
	Plus alcohol consumption and BMI	0.88 (0.81, 0.95)	0.001	0.85 (0.70, 1.05)	0.134
	Plus dietary intakes	0.86 (0.79, 0.93)	<0.001	0.85 (0.69, 1.03)	0.104
	Plus lipid markers	0.87 (0.78, 0.97)	0.009	0.85 (0.70, 1.04)	0.122
Conjugated linoleic acid (CLA; 18:2n6t)	Adjusted for age, sex, and lifestyle factors	1.00 (0.85, 1.17)	0.971	0.86 (0.76, 0.98)	0.022
	Plus alcohol consumption and BMI	1.05 (0.88, 1.25)	0.583	0.89 (0.79, 1.00)	0.042
	Plus dietary intakes	1.19 (0.90, 1.59)	0.217	0.91 (0.82, 1.01)	0.089
	Plus lipid markers	1.20 (0.91, 1.60)	0.195	0.92 (0.84, 1.02)	0.100
trans-palmitoleic acid (TPA; 16:1t) †	Adjusted for age, sex, and lifestyle factors	0.97 (0.86, 1.10)	0.675	0.91 (0.77, 1.07)	0.243
	Plus alcohol consumption and BMI	0.97 (0.86, 1.10)	0.614	0.92 (0.79, 1.08)	0.314
	Plus dietary intakes	0.94 (0.80, 1.10)	0.428	0.95 (0.80, 1.12)	0.550
	Plus lipid markers	0.98 (0.82, 1.17)	0.798	0.96 (0.81, 1.13)	0.603

BMI: Body mass index; CHD: Coronary heart disease; CI: Confidence interval; FA: Fatty acid; HR: Hazard ratio; SD: Standard deviation; TFA: Trans fatty acid.

Results are pooled HRs (95% CI) per 1-SD higher fatty acid concentrations using random-effects meta-analysis, estimated on a complete-case basis within EPIC-CVD (subcohort n=15,125) due to missing data of potential confounders or mediators.

* The basic adjustment included adjustment for batch, age, smoking, history of diabetes, history of hypertension, and physical activity, stratified by center and sex; then, plus alcohol consumption (current, non-current) and BMI; then, plus intakes of fruit and vegetables, dairy products, meat, fish, olive oil, margarine, and alcoholic beverages (g/day); and finally, plus total cholesterol, high-density-lipoprotein cholesterol, loge triglycerides.

† Due to missing data, analysis of TPA was conducted among 9,044 sub-cohort participants with 5,659 CHD and 3,663 stroke cases.

Supplementary eTable 11. Associations of plasma phospholipids FA ratio variables with CHD and stroke, with further adjustments estimated from EPIC-CVD participants (subcohort n=15,125).

Ratios of fatty acids	Adjustments*	CHD (6,341 cases)		Stroke (4,719 cases)	
		HR (95%CI)	*P*	HR (95%CI)	*P*
Ratio of 16:1 to 16:0	Adjusted for age, sex, and lifestyle factors	1.05 (0.97, 1.14)	0.233	1.18 (1.07, 1.29)	0.001
	Plus alcohol consumption and BMI	1.04 (0.96, 1.12)	0.303	1.16 (1.05, 1.28)	0.003
	Plus dietary intakes	1.07 (0.97, 1.18)	0.179	1.19 (1.06, 1.32)	0.002
	Plus lipid markers	1.06 (0.97, 1.16)	0.184	1.16 (1.05, 1.29)	0.004
Ratio of 18:1 to 18:0	Adjusted for age, sex, and lifestyle factors	0.92 (0.85, 0.99)	0.023	1.02 (0.90, 1.15)	0.810
	Plus alcohol consumption and BMI	0.96 (0.90, 1.03)	0.277	1.05 (0.94, 1.18)	0.384
	Plus dietary intakes	0.93 (0.83, 1.04)	0.204	1.07 (0.96, 1.19)	0.211
	Plus lipid markers	0.95 (0.85, 1.07)	0.419	1.07 (0.97, 1.19)	0.165
Ratio of PUFA to MUFA	Adjusted for age, sex, and lifestyle factors	1.02 (0.94, 1.11)	0.629	0.97 (0.81, 1.16)	0.722
	Plus alcohol consumption and BMI	1.02 (0.93, 1.12)	0.648	0.95 (0.80, 1.13)	0.570
	Plus dietary intakes	1.01 (0.91, 1.12)	0.854	0.93 (0.79, 1.08)	0.341
	Plus lipid markers	1.03 (0.91, 1.16)	0.647	0.94 (0.81, 1.09)	0.438
Ratio of n-6 to n-3 PUFA	Adjusted for age, sex, and lifestyle factors	1.02 (0.89, 1.17)	0.798	0.97 (0.84, 1.11)	0.647
	Plus alcohol consumption and BMI	1.04 (0.92, 1.18)	0.486	0.97 (0.86, 1.10)	0.647
	Plus dietary intakes	0.99 (0.85, 1.14)	0.845	0.97 (0.85, 1.11)	0.654
	Plus lipid markers	1.07 (0.96, 1.20)	0.195	0.96 (0.84, 1.10)	0.576
Ratio of 18:3n6 to 18:2n6 (D6D)	Adjusted for age, sex, and lifestyle factors	1.06 (1.00, 1.13)	0.050	1.06 (0.97, 1.16)	0.185
	Plus alcohol consumption and BMI	1.03 (0.99, 1.07)	0.213	1.04 (0.96, 1.11)	0.336
	Plus dietary intakes	1.04 (0.98, 1.11)	0.188	1.03 (0.97, 1.10)	0.337
	Plus lipid markers	0.98 (0.90, 1.07)	0.708	1.00 (0.92, 1.08)	0.983
Ratio of 20:4n6 to 20:3n6 (D5D)	Adjusted for age, sex, and lifestyle factors	0.14 (0.07, 0.27)	<0.001	0.58 (0.29, 1.17)	0.127
	Plus alcohol consumption and BMI	0.24 (0.14, 0.39)	<0.001	0.79 (0.44, 1.44)	0.449
	Plus dietary intakes	0.22 (0.14, 0.36)	<0.001	0.63 (0.36, 1.11)	0.111
	Plus lipid markers	0.58 (0.32, 1.07)	0.081	0.97 (0.59, 1.60)	0.905
Ratio of 20:3n6 to 18:2n6	Adjusted for age, sex, and lifestyle factors	1.28 (1.10, 1.50)	0.002	1.21 (1.04, 1.39)	0.011
	Plus alcohol consumption and BMI	1.21 (1.07, 1.37)	0.002	1.15 (1.03, 1.29)	0.015
	Plus dietary intakes	1.22 (1.07, 1.39)	0.004	1.18 (1.05, 1.34)	0.008
	Plus lipid markers	1.08 (0.99, 1.17)	0.088	1.12 (1.02, 1.23)	0.021

BMI: Body mass index; CHD: Coronary heart disease; CI: Confidence interval; FA: Fatty acid; HR: Hazard ratio; MUFA: Monounsaturated fatty acid; PUFA: Polyunsaturated fatty acid; SD: Standard deviation.

Results are pooled HRs (95% CI) per 1-SD higher fatty acid concentrations using random-effects meta-analysis, estimated on a complete-case basis within EPIC-CVD (subcohort n=15,125) due to missing data of potential confounders or mediators.

* The basic adjustment included adjustment for batch, age, smoking, history of diabetes, history of hypertension, and physical activity, stratified by center and sex; then, plus alcohol consumption (current, non-current) and BMI; then, plus intakes of fruit and vegetables, dairy products, meat, fish, olive oil, margarine, and alcoholic beverages (g/day); and finally, plus total cholesterol, high-density-lipoprotein cholesterol, loge triglycerides.

Supplementary eTable 12. Associations of FA subtypes with CHD and stroke in sensitivity analysis 2, adjusted for conventional CVD risk factors and plus further adjustments for CRP and HbA1C or glucose

FAs	CHD (7,872 cases)				Stroke (5,866 cases)			
	Adjusted for conventional CVD risk factors†		Plus CPR+ HbA1C or glucose		Adjusted for conventional CVD risk factors†		Plus CPR+ HbA1C or glucose	
	HR (95%CI)	*P*	HR (95%CI)	*P*	HR (95%CI)	*P*	HR (95%CI)	*P*
**Total SFA**	1.07 (1.01, 1.13)	0.023	1.07 (1.00, 1.15)	0.051	1.12 (1.07, 1.17)	<0.001	1.09 (1.05, 1.14)	<0.001
Even-chain SFA*	1.17 (1.10, 1.24)	<0.001	1.18 (1.12, 1.25)	<0.001	1.17 (1.10, 1.25)	<0.001	1.13 (1.05, 1.21)	0.001
Odd-chain SFA*	0.82 (0.74, 0.91)	<0.001	0.83 (0.74, 0.92)	<0.001	0.75 (0.69, 0.82)	<0.001	0.77 (0.71, 0.84)	<0.001
Longer-chain SFA*	0.94 (0.81, 1.08)	0.366	0.96 (0.82, 1.13)	0.617	0.88 (0.77, 1.01)	0.064	0.88 (0.76, 1.02)	0.087
**Total MUFA**	1.05 (0.94, 1.17)	0.413	1.07 (0.97, 1.17)	0.166	1.10 (0.99, 1.21)	0.064	1.10 (1.00, 1.21)	0.045
**Total PUFA**	0.93 (0.87, 1.00)	0.056	0.93 (0.87, 0.99)	0.019	0.87 (0.79, 0.96)	0.007	0.88 (0.80, 0.97)	0.008
**n-3 PUFA**	0.89 (0.86, 0.93)	<0.001	0.91 (0.87, 0.96)	<0.001	1.00 (0.90, 1.11)	0.958	1.01 (0.93, 1.11)	0.768
DHA	0.88 (0.84, 0.93)	<0.001	0.90 (0.86, 0.94)	<0.001	1.00 (0.90, 1.12)	0.987	1.02 (0.93, 1.13)	0.676
**n-6 PUFA**	1.02 (0.95, 1.09)	0.614	0.99 (0.93, 1.05)	0.701	0.86 (0.82, 0.92)	<0.001	0.87 (0.82, 0.92)	<0.001
LA	0.98 (0.94, 1.03)	0.438	0.98 (0.94, 1.03)	0.507	0.87 (0.82, 0.92)	<0.001	0.89 (0.85, 0.93)	<0.001

CHD: Coronary heart disease; CI: Confidence interval; CRP: C-reactive protein; DHA: Docosahexaenoic acid; FA: Fatty acid; HbA1C: Hemoglobin A1C; HR: Hazard ratio; LA: Linoleic acid; MUFA: Monounsaturated fatty acid; PUFA: Polyunsaturated fatty acid; SD: Standard deviation; SFA: Saturated fatty acid.

Results are pooled HRs (95% CI) per 1-SD higher FAs using random-effects meta-analysis, estimated on a complete-case basis (n= 157,479) due to missing data of potential confounders or mediators.

* For even-chain, odd-chain, and longer-chain SFAs, analyses were restricted to EPIC-CVD (n= 27,116), involving 5,937 CHD and 4,338 stroke cases.

† Models were adjusted for conventional CVD risk factors, including age, smoking, history of diabetes, history of hypertension, physical activity, alcohol consumption, BMI, total cholesterol, high-density-lipoprotein cholesterol, and loge triglycerides, and stratified by sex. Comparisons were between with or without further adjustment for CPR and HbA1C (for EPIC-CVD and UKB) or glucose (for INTERVAL only due to no baseline data on HbA1C).

Supplementary eTable 13. Comparisons between associations of fatty acids in absolute and relative concentrations with CHD and stroke in sensitivity analysis 3, estimated from UKB and INTERVAL.

FAs \ Adjustments*	CHD (2,083 cases)				Stroke (1,661 cases)			
	Relative (%)		Absolute (mmol/l)		Relative (%)		Absolute (mmol/l)	
	HR (95%CI)	*P*	HR (95%CI)	*P*	HR (95%CI)	*P*	HR (95%CI)	*P*
**Total SFA**								
Adjusted for age, sex, and lifestyle factors	0.99 (0.89, 1.10)	0.837	1.21 (1.12, 1.30)	<0.001	1.06 (1.01, 1.12)	0.011	1.03 (0.99, 1.08)	0.169
Plus alcohol consumption and BMI	0.98 (0.88, 1.09)	0.678	1.20 (1.11, 1.28)	<0.001	1.06 (1.01, 1.11)	0.019	1.02 (0.98, 1.07)	0.312
Plus lipid markers	1.00 (0.94, 1.08)	0.927	1.05 (0.71, 1.55)	0.808	1.09 (1.00, 1.20)	0.050	1.14 (0.84, 1.55)	0.398
**Total MUFA**								
Adjusted for age, sex, and lifestyle factors	1.23 (1.18, 1.28)	<0.001	1.24 (1.16, 1.33)	<0.001	1.07 (1.02, 1.12)	0.007	1.04 (0.99, 1.09)	0.103
Plus alcohol consumption and BMI	1.20 (1.15, 1.25)	<0.001	1.22 (1.15, 1.30)	<0.001	1.04 (0.99, 1.10)	0.138	1.02 (0.97, 1.07)	0.373
Plus lipid markers	1.24 (1.06, 1.45)	0.007	1.16 (0.83, 1.62)	0.393	1.19 (1.00, 1.41)	0.049	1.15 (0.91, 1.45)	0.242
**Total PUFA**								
Adjusted for age, sex, and lifestyle factors	0.86 (0.83, 0.89)	<0.001	1.20 (1.14, 1.27)	<0.001	0.92 (0.88, 0.97)	0.001	0.97 (0.92, 1.02)	0.231
Plus alcohol consumption and BMI	0.88 (0.84, 0.92)	<0.001	1.21 (1.13, 1.30)	<0.001	0.94 (0.90, 0.99)	0.016	0.97 (0.93, 1.02)	0.317
Plus lipid markers	0.87 (0.76, 1.00)	0.058	0.96 (0.79, 1.16)	0.652	0.81 (0.66, 0.98)	0.029	0.77 (0.67, 0.88)	<0.001
**n-3 PUFA**								
Adjusted for age, sex, and lifestyle factors	0.87 (0.82, 0.91)	<0.001	1.03 (0.93, 1.14)	0.636	0.97 (0.84, 1.12)	0.697	0.98 (0.85, 1.14)	0.830
Plus alcohol consumption and BMI	0.88 (0.84, 0.93)	<0.001	1.03 (0.93, 1.15)	0.529	0.99 (0.85, 1.15)	0.898	1.00 (0.86, 1.16)	0.957
Plus lipid markers	0.90 (0.85, 0.94)	<0.001	0.90 (0.85, 0.95)	<0.001	1.00 (0.84, 1.19)	0.977	0.98 (0.83, 1.17)	0.860
**DHA**								
Adjusted for age, sex, and lifestyle factors	0.83 (0.78, 0.87)	<0.001	0.98 (0.85, 1.14)	0.824	0.96 (0.86, 1.08)	0.525	0.97 (0.86, 1.10)	0.653
Plus alcohol consumption and BMI	0.85 (0.81, 0.89)	<0.001	1.00 (0.88, 1.15)	0.987	0.99 (0.88, 1.11)	0.838	0.99 (0.88, 1.12)	0.880
Plus lipid markers	0.89 (0.84, 0.94)	<0.001	0.90 (0.85, 0.95)	<0.001	0.99 (0.83, 1.17)	0.895	0.97 (0.84, 1.11)	0.666
**n-6 PUFA**								
Adjusted for age, sex, and lifestyle factors	0.89 (0.86, 0.93)	<0.001	1.23 (1.18, 1.29)	<0.001	0.92 (0.84, 1.01)	0.100	0.99 (0.94, 1.04)	0.606
Plus alcohol consumption and BMI	0.92 (0.88, 0.96)	<0.001	1.24 (1.18, 1.29)	<0.001	0.95 (0.89, 1.02)	0.174	0.99 (0.94, 1.04)	0.686
Plus lipid markers	0.95 (0.88, 1.03)	0.224	1.05 (0.80, 1.39)	0.703	0.82 (0.75, 0.90)	<0.001	0.82 (0.67, 1.00)	0.050
**LA**								
Adjusted for age, sex, and lifestyle factors	0.95 (0.85, 1.05)	0.325	1.24 (1.18, 1.29)	<0.001	0.91 (0.78, 1.05)	0.180	0.98 (0.94, 1.03)	0.531
Plus alcohol consumption and BMI	0.98 (0.88, 1.08)	0.664	1.24 (1.19, 1.30)	<0.001	0.93 (0.82, 1.06)	0.258	0.99 (0.94, 1.04)	0.621
Plus lipid markers	0.97 (0.92, 1.03)	0.271	1.02 (0.85, 1.22)	0.872	0.90 (0.84, 0.97)	0.004	0.82 (0.63, 1.06)	0.136
**CLA† (INTERVAL only)**								
Adjusted for age, sex, and lifestyle factors	0.95 (0.83, 1.07)	0.381	1.05 (0.95, 1.16)	0.370	0.92 (0.80, 1.07)	0.298	0.98 (0.84, 1.13)	0.756
Plus alcohol consumption and BMI	0.94 (0.83, 1.07)	0.372	1.05 (0.94, 1.16)	0.418	0.92 (0.80, 1.07)	0.305	0.97 (0.84, 1.13)	0.734
Plus lipid markers	0.85 (0.73, 0.98)	0.021	0.85 (0.73, 0.98)	0.030	0.89 (0.76, 1.04)	0.153	0.91 (0.76, 1.08)	0.270

BMI: Body mass index; CHD: Coronary heart disease; CI: Confidence interval; CLA: Conjugated linoleic acid; DHA: Docosahexaenoic acid; FA: Fatty acid; HR: Hazard ratio; LA: Linoleic acid; MUFA: Monounsaturated fatty acid; PUFA: Polyunsaturated fatty acid; SD: Standard deviation; SFA: Saturated fatty acid.

Results are pooled HRs (95% CI) per 1-SD higher FAs using random-effects meta-analysis, estimated on a complete-case basis (n=139,538) due to missing data of potential confounders or mediators.

* Adjustments: i) basic adjustment for age, sex and lifestyle factors consists of adjustment for age, smoking, history of diabetes, history of hypertension, and physical activity, stratified by sex; ii) plus alcohol consumption and BMI; and iii) plus lipid markers (i.e., total cholesterol, high-density-lipoprotein cholesterol, loge triglycerides).

† CLA was only available in INTERVAL; due to missing data, analysis of CLA here was conducted among 37,926 INTERVAL participants, including 251 CHD and 190 stroke cases.

Supplementary eTable 14. Estimated regression dilution ratios for fatty acids and corrected hazard ratios in sensitivity analysis 4.

FAs (%)	RDR (95%CI)	HR (95%CI) per SD for CHD	HR (95%CI) per SD for Stroke
Total SFA	0.44 (0.39, 0.49)	1.17 (1.09, 1.27)	1.13 (1.04, 1.22)
		1.44 (1.21, 1.71)	1.32 (1.11, 1.56)
Total MUFA	0.61 (0.57, 0.65)	1.01 (0.90, 1.13)	1.06 (0.98, 1.14)
		1.01 (0.84, 1.21)	1.09 (0.96, 1.25)
Total PUFA	0.55 (0.51, 0.60)	0.93 (0.87, 1.00)	0.91 (0.84, 1.00)
		0.88 (0.78, 0.99)	0.85 (0.72, 1.00)
n-3 PUFA	0.57 (0.53, 0.62)	0.91 (0.85, 0.97)	0.97 (0.90, 1.05)
		0.85 (0.75, 0.96)	0.95 (0.83, 1.09)
DHA	0.53 (0.48, 0.57)	0.91 (0.84, 0.98)	0.96 (0.90, 1.03)
		0.83 (0.71, 0.96)	0.93 (0.81, 1.07)
n-6 PUFA	0.55 (0.50, 0.59)	0.98 (0.90, 1.07)	0.94 (0.91, 0.98)
		0.96 (0.82, 1.13)	0.90 (0.84, 0.97)
LA	0.55 (0.51, 0.60)	0.95 (0.90, 1.00)	0.89 (0.83, 0.95)
		0.91 (0.83, 1.00)	0.81 (0.71, 0.91)
Ratio of PUFA to MUFA	0.58 (0.53, 0.62)	0.98 (0.87, 1.10)	0.95 (0.86, 1.06)
		0.97 (0.79, 1.19)	0.92 (0.76, 1.10)
Ratio of n-6 to n-3 PUFA	0.67 (0.62, 0.72)	1.05 (0.97, 1.14)	1.01 (0.94, 1.08)
		1.08 (0.96, 1.21)	1.01 (0.91, 1.13)

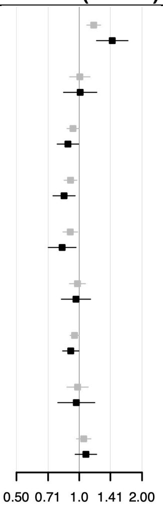

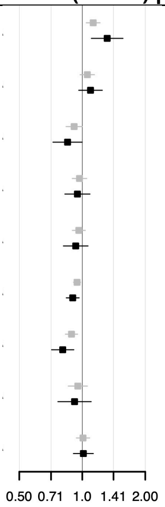

CHD: Coronary heart disease; DHA: Docosahexaenoic acid; FA: Fatty acid; HR: Hazard ratio; LA: Linoleic acid; MUFA: Monounsaturated fatty acid; PUFA: Polyunsaturated fatty acid; RDR: Regression dilution ratio; SD: Standard deviation; SFA: Saturated fatty acid.
RDRs were estimated from repeat data of 1187 UKB participants, adjusted for sex, age, smoking status, history of diabetes, history of hypertension, and physical activity. Estimates in grey were the original HRs estimated from 172,891 participants from EPIC-CVD, UKB and INTERVAL, while estimates in black were the final HRs corrected for regression dilution bias.

Supplementary eTable 15. Hazard ratios and subdistribution hazard ratios per 1-SD higher FAs for cardiovascular events adjusted for lifestyle factors without/with adjustment for competing risks in sensitivity analysis 5, estimated in 172,891 participants from EPIC-CVD, UKB and INTERVAL

**FAs (%)**	**CHD**		**Stroke**		**Ischaemic stroke**		**Haemorrhagic stroke**	
**Cox model results**	**9,453 cases**		**8,182 cases**		**4,935 cases**		**1,603 cases**	
**(primary analysis)**	**HR (95%CI)**	***P***	**HR (95%CI)**	***P***	**HR (95%CI)**	***P***	**HR (95%CI)**	***P***
**Total SFA**	1.17 (1.09, 1.27)	<0.001	1.13 (1.04, 1.22)	0.002	1.16 (1.05, 1.28)	0.004	0.99 (0.81, 1.20)	0.887
Even-chain SFA*	1.24 (1.18, 1.32)	<0.001	1.23 (1.10, 1.38)	<0.001	1.28 (1.12, 1.47)	<0.001	1.02 (0.76, 1.37)	0.908
Odd-chain SFA*	0.82 (0.76, 0.87)	<0.001	0.73 (0.67, 0.78)	<0.001	0.71 (0.64, 0.79)	<0.001	0.72 (0.62, 0.85)	<0.001
Longer-chain SFA*	0.95 (0.80, 1.12)	0.520	0.84 (0.72, 0.99)	0.035	0.90 (0.79, 1.03)	0.120	0.84 (0.63, 1.10)	0.204
**Total MUFA**	1.01 (0.90, 1.13)	0.913	1.06 (0.98, 1.14)	0.170	1.01 (0.91, 1.12)	0.856	1.11 (0.93, 1.33)	0.243
**Total PUFA**	0.93 (0.87, 1.00)	0.038	0.91 (0.84, 1.00)	0.048	0.94 (0.85, 1.04)	0.216	0.93 (0.78, 1.11)	0.401
**n-3 PUFA**	0.91 (0.85, 0.97)	0.007	0.97 (0.90, 1.05)	0.448	0.98 (0.87, 1.10)	0.680	1.03 (0.85, 1.25)	0.737
DHA	0.91 (0.84, 0.98)	0.016	0.96 (0.90, 1.03)	0.296	0.97 (0.86, 1.09)	0.606	1.07 (0.87, 1.32)	0.505
**n-6 PUFA**	0.98 (0.90, 1.07)	0.659	0.94 (0.91, 0.98)	0.004	0.95 (0.91, 1.00)	0.052	0.94 (0.86, 1.02)	0.130
LA	0.95 (0.90, 1.00)	0.039	0.89 (0.83, 0.95)	0.001	0.90 (0.84, 0.96)	0.003	0.98 (0.86, 1.11)	0.728
**Fine and Gray model results (competing risk adjusted)**	**9,208 cases; 13,176 competing events (non-CHD death and stroke)†**		**7,968 cases; 14,758 competing events (non-stroke death and CHD)**		**4,792 cases; 15,333 competing events (non-ischaemic stroke death and CHD)**		**1,574 cases; 15,158 competing events (non-haemorrhagic stroke death and CHD)**	
	**SHR (95% CI)**	**P**	**SHR (95% CI)**	**P**	**SHR (95% CI)**	**P**	**SHR (95% CI)**	**P**
**Total SFA**	1.14(1.06,1.23)	<0.001	1.11(1.02,1.21)	0.019	1.13(1.01,1.27)	0.037	0.95(0.72,1.26)	0.714
Even-chain SFA*	1.20(1.13,1.29)	<0.001	1.17(1.03,1.33)	0.016	1.20(1.02,1.43)	0.030	0.96(0.62,1.48)	0.859
Odd-chain SFA*	0.88(0.83,0.93)	<0.001	0.77(0.69,0.87)	<0.001	0.74(0.62,0.87)	<0.001	0.73(0.57,0.94)	0.013
Longer-chain SFA*	0.99(0.88,1.12)	0.887	0.91(0.79,1.05)	0.203	0.96(0.86,1.08)	0.531	0.82(0.61,1.10)	0.187
**Total MUFA**	0.98(0.86,1.11)	0.701	1.05(1.01,1.10)	0.010	1.03(0.96,1.10)	0.419	1.11(0.94,1.31)	0.212
**Total PUFA**	0.97(0.89,1.07)	0.575	0.95(0.87,1.04)	0.286	0.98(0.88,1.09)	0.648	0.97(0.80,1.17)	0.741
**n-3 PUFA**	0.92(0.85,1.00)	0.051	0.96(0.91,1.02)	0.176	0.97(0.88,1.07)	0.502	1.03(0.89,1.21)	0.664
DHA	0.92(0.83,1.01)	0.070	0.96(0.91,1.01)	0.088	0.96(0.87,1.05)	0.355	1.05(0.88,1.24)	0.591
**n-6 PUFA**	1.01(0.91,1.11)	0.922	0.95(0.91,0.99)	0.021	0.97(0.91,1.03)	0.345	0.91(0.80,1.04)	0.162
LA	0.96(0.90,1.04)	0.329	0.92(0.86,0.98)	0.014	0.92(0.85,1.00)	0.042	1.00(0.84,1.20)	0.958

CHD: Coronary heart disease; CI: Confidence interval; CVD: Cardiovascular disease; DHA: Docosahexaenoic acid; FA: Fatty acid; HR: Hazard ratio; LA: Linoleic acid; MUFA: Monounsaturated fatty acid; PUFA: Polyunsaturated fatty acid; SD: Standard deviation; SFA: Saturated fatty acid; SHR: Subdistribution Hazard Ratios.

Model included: batch (EPIC-CVD only), center (EPIC-CVD only), sex, age, smoking, history of diabetes, history of hypertension, and physical activity.

The slight differences in the number of cases of main event between Cox model results and the Fine & Gray model results came from overlap of CHD and Stroke cases in the EPIC-CVD case-cohort study (i.e., 245 participants coded as both CHD and stroke with stroke happening first; and 214 participants coded as both CHD and stroke with CHD happening first).

* For even-chain, odd-chain, and longer-chain SFAs, analyses were restricted to EPIC-CVD (n=31,606; 7,343 CHD cases and 6,499 stroke cases in Cox model results).

† Results of CHD remained similar when being contrasted with ischemic stroke instead of overall stroke (i.e., 9,275 cases and 10,871 competing events [non-CHD death and ischaemic stroke]).

Supplementary eTable 16. The study profiles of identified systematic reviews with meta-analysis on FA and cardiovascular outcomes, as well as the inclusion of eligible primary studies

Author, year	Databases	Up to	N articles	Fatty acids of interest	Outcome of interest	Eligible primary studies on CHD or stroke*	Reasons for exclusion*
Li, 2022[23]	PubMed, EMBASE, Web of Science, Cochrane Library	Mar 2022	25	***SFAs:*** SFA, C14:0, C16:0, C18:0, C15:0, C17:0, C15:0 +C17:0, C20:0, C22:0,C24:0	CHD, Stroke, CVD	***19 non-duplicated***[24–42] + 2 duplicated[43,44] with other primary studies or current studies	**Not exposure of interest:** 1 did not assess FAs in relative concentration (%)[45]; **Not outcomes of interest:** 1 on CVD[46]; 1 on CVD mortality[47]; **No estimates reported:** 1 did not report RR or 95%CI[48].
Jiang, 2022[49]	PubMed, EMBASE, Web of Science, Cochrane Library	1 Mar 2022	31	***N3-PUFAs:*** ALA, EPA, n3-DPA, DHA, EPA+DPA+DHA	CHD, Stroke, CVD	22 [25,30,32,33,36,39,44,50–64] (***11 non-duplicated***[50–52,54–56,58,59,62–64] + 11 duplicated[25,30,32,33,36,39,44,53,57,60,61])	**Not outcomes of interest:** 2 on CVD[46,65]; 6 on CVD mortality[47,66–70]; **No estimates reported:** 1 did not report RR or 95% CI[48]
Ren, 2022[71]	PubMed, Embase, Web of Science, Cochrane Library	May 2021	32	***N6-PUFAs:*** n6-PUFA, LA, AA	CHD	13 [25,27,39,44,51,63,72–78] (***7 non-duplicated***[72–78] + 6 duplicated[25,27,39,44,51,63])	**Not prospective study design:** 11 case-control studies[79–89]; **Not general population:** 3 in patients with CVD[90–92]; **Not exposure of interest:** 1 did not assess FAs in relative concentration (%)[93]; **Not outcomes of interest:** 2 on CVD[65,94]; 2 on CVD mortality[47,69].
Trieu, 2021[38]	Medline, Embase, Scopus, Web of Science, CENTRAL	27 Jun 2021	18	***FA biomarkers of dairy fat intake:*** C15:0, C17:0, trans16:1n7	CHD, stroke, HF, total CVD, and mortality	14[31–35,38,40,41,43,44,95–98] (***2 non-duplicated***[95,96] + 12 duplicated[31–35,38,40,41,43,44,76,98])	**Not outcomes of interest:** 3 on HF[99–101], 1 on CVD mortality[102]
Naghshi, 2021[103]	PubMed, Scopus, ISI Web of Science, Google Scholar	30 Apr 2021	13	***ALA***	CVD mortality, CHD mortality	5[50,64,75,104,105] (***1 non-duplicated***[105] + 4 duplicated[50,64,75,104])	**Not general population:** 2 in patients with CVD[90,106]; **Not outcomes of interest:** 6 on CVD mortality[47,67,69,102,107,108].
Liu, 2020[109]	PubMed, Embase, Web of Science	18 Jul 2019	10	***Longer-chain SFA:*** C20:0, C22:0, C24:0, longer-chain SFA	Any CVD as total CVD	4[26,37,74,76] duplicated with above identified primary studies	**Not prospective study design:** 2 case-control studies[89,110]; **Not outcomes of interest:** 1 on CVD mortality[111], 2 on HF[100,112], 1 on AF[113];
Zhang, 2020[114]	PubMed, EMBASE, Web of Science	29 Feb 2020	11	***LA***	Stroke	10[29,30,32,33,57,58,72,77,78,115] duplicated with above identified primary studies or current studies	**Not prospective study design:** 1 case-control study[116];
Li, 2020[117]	PUBMED, EMBASE	31 Jul 2019	14	***LA***	CVD mortality	5[73,75,77,78,118] (***1 non-duplicated***[118] + 4 duplicated[73,75,77,78])	**Not general population:** 2 in patients with CVD[90,92]; **Not outcomes of interest:** 1 on CVD[65]; 6 on CVD mortality[47,67,69,102,107,119].
Yang, 2017[120]	PubMed, EMBASE, Cochrane Library,Web of science,ProQuest	Jun 2017	10	***Long-chain n3-PUFA***: EPA, n3-DPA, DHA, long-chain n3-PUFA	Stroke	9[30,32,33,57,58,62,121–123] (***2 non-duplicated***[121,122] + 7 duplicated[30,32,33,57,58,62,123])	**Not general population:** 1 in patients with CVD[124].
Chowdhury, 2014[125]	MEDLINE, Science Citation Index,Cochrane Central Register of Controlled Trials	1 Jul 2013	29	***All fatty acids:*** SFA, MUFA, n3-PUFA, n6-PUFA, trans FA, and individual FAs in each group	CHD	17[24,30,39–44,47,51–53,62,67,126–128] (***3 non-duplicated***[126–128] + 14 duplicated[24,30,39–44,47,51–53,62,67])	**Not general population:** 4 in patients with CAD or others[90,129–131]; **Not exposure of interest:** 5 did not assess FAs in relative concentration (%)[132–136]; **Not outcomes of interest:** 1 on CVD mortality[66]; **No estimates reported:** 2 did not report RR or 95% CI[48,137].
de Goede, 2013[75]	PubMed	NR	5	***PUFAs:*** LA, AA, ALA, EPA, DHA, EPA+DHA	CHD mortallity	4[39,47,75,138] (***1 non-duplicated***[138] + 3 duplicated[39,47,75])	**Not general population:** 1 in patients with CAD[90].
Pan, 2012[139]	PubMed, EMBASE, Web of Science,Cochrane Library, Clinical trial registry	Jan 2012	17	***ALA***	CVD	5 duplicated with above identified primary studies[30,39,44,51,121]	**Not prospective study design:** 8 case-control studies[140–147]; **Not general population:** 1 in patients with established CAD[90]; **Not outcomes of interest:** 2 on CVD mortality[47,67]; **No estimates reported:** 1 did not report RR or 95% CI[48].
Chowdhury, 2012[148]	Embase, BIOSIS, Science Citation Index	Sep 2012	4	***n3-PUFA***	Stroke	4 duplicated with above identified primary studies[29,30,121,122]	-

In total, 47 non-duplicated primary studies identified from above systematic reviews with meta-analysis were reviewed and estimates were summarized. Among them, Malik et al (2012)[95] was published as an abstract but the estimates were reported in Trieu et al (2021)'s study[38], which were also extracted in our analysis.

AA: Arachidonic acid; AF: Atrial fibrillation; ALA: Alpha-linolenic acid; CAD: Coronary artery disease; CHD: Coronary heart disease; CI: Confidence interval; CVD: Cardiovascular disease; DHA: Docosahexaenoic acid; EPA: Eicosapentaenoic acid; FA: Fatty acid; HF: Heart failure; LA: Linoleic acid; MUFA: Monounsaturated fatty acid; n3-DPA: Docosapentaenoic acid; PUFA: Polyunsaturated fatty acid; RR: Risk ratio; SFA: Saturated fatty acid; VLCSFA, very-long-chain saturated fatty acid.

* Primary studies are eligible for the inclusion of our current updated meta-analysis if they i) were in prospective study design; ii) accessed fatty acids (expressed as percentage of total fatty acids) of interest; iii) assessed associations of fatty acids with CHD or stroke; iv) were conducted among general populations; and v) not duplicated with our current study.

Supplementary eTable 17. Characteristics of 49 non-duplicated primary studies included in the updated meta-analysis of associations of FA biomarkers with CHD and stroke

Paper	Study design	Data source	Region	Population	N	Median/max follow-up, y	Mean age, y	Female (%)	Baseline year	FA assayed	Exposure in relative concentration (%)	Outcome
**Primary studies identified from published meta-analyses, with any non-duplicated associations of FAs with CHD or stroke (n=47)**												
Trieu 2021[38]	PC	60YO	Sweden	GP	4150	16.6/17.5	61*	51	1997-1999	serum CE	(n=1) C15:0	CHD (386)<br>IS (192)
Liu 2019[28]	NCC	WHI-OS	US	Postmenopausal women free of CVD	2428	4.5†/11.7	68	100	1994-1998	plasma PL	(n=6) total SFA, longer-chain SFA, total MUFA, total n-6 PUFA, total n-3 PUFA, total trans	CHD (1214)
	PC	3C Study	France		1416	6.6/9.8	75	61	1999-2000	plasma		CHD (57)<br>IS (26)
	PC	60YO	Sweden		4150	14.5/15.9	60	52	1997-1998	serum CE		CHD (199)<br>IS (155)
	PC	AGES-Reykjavik	Iceland		1195	10.0/13.3	77	61	2002-2006	plasma PL		CHD (286)<br>IS (123)
	PC	ARIC	US		3749	22.6/25.1	54	48	1987-1989	plasma PL		CHD (398)<br>IS (188)
	PC	CCCC	Taiwan		1838	11.0/22.8	61	45	1992-2000	plasma		CHD (196)<br>stroke (243)
	PC	CHS	US		2907	11.0/22.1	73	64	1992-1993	plasma PL		CHD (875)<br>IS (408)
	PC	FHS-Offspring	US		2500	6.4/9.3	66	57	2005-2008	RBC		CHD (103)<br>IS (79)
	NCC	HPFS	US		1510	12.6/17.4	65	0	1993-1995	plasma<br>RBC§		CHD (431)<br>stroke (120)
	PC	HS	Japan		3103	10.2/10.4	61	58	2002-2003	serum		CHD (78)<br>IS (97)
	PC	KIHD	Finland		1837	18.4/27.2	52	0	1984-1989	serum		CHD (472)<br>IS (151)
	PC	MCCS	Australia		6265	7.1/18.5	56	54	1990-1994	plasma PL		fatal CHD (238)<br>fatal IS (44)
Marklund 2019[118]	PC	MESA	US	GP free of CVD	2722	8.4/10.9	62	53	2000-2002	plasma PL	(n=2) C18:2n6, C20:4n6	CHD (143)<br>IS (53)
	NCC	MP-CVDRF	Netherlands		444	23.7/28.8	51	30	1987-1991	plasma CE		fatal CHD (222)
	NCC	NHS	US		1970	15.0/22.7	60	100	1989-1990	plasma<br>RBC§		CHD (437)<br>stroke (468)
	NCC	NSHDS I‡	Sweden		183	NR	54	21	1987-1994	plasma PL		CHD (64)
	NCC	NSHDS II‡	Sweden		759	3.7/10.8	54	24	1987-1999	plasma PL		CHD (353)
	NCC	NSHDS III‡	Sweden		317	2.5/6.5	55	39	1987-1995	plasma PL		IS (85)
	NCC	PHS	US		2000	NR	69	0	1995-2001	RBC		CHD (1000)
	PC	PIVUS	Sweden		835	10.0/10.9	70	53	2001-2004	serum PL<br>serum CE§		CHD (57)<br>IS (37)
	NCC	SCHS	Singapore		1555	4.6/14.4	66	35	1994-2005	plasma		CHD (759)
	PC	SHHEC	UK		4391	23.3/24.8	49	48	1985-1986	AT		CHD (936)<br>stroke (290)
	PC	ULSAM 50	Sweden		1992	27.9/41.7	50	0	1970-1973	serum CE		CHD (643)<br>IS (313)
	PC	ULSAM 70	Sweden		763	12.9/20.2	71	0	1991-1995	AT		CHD (181)<br>IS (115)

	PC	WHI-MS	US		5263	15.5/20.2	70	100	1996	RBC		CHD (484)<br>IS (295)
Papandreou 2019[74]	NCC	PREDIMED	Spain	GP free of CVD or any severe chronic illness	408	7.2 (max)	67	41	2003-2009	RBC	(n=28) total SFA, C14:0, C16:0, C18:0, C20:0, C22:0, C24:0, longer-chain SFA, total MUFA, C16:1n7, C18:1n9, C20:1n9, C24:1n9, total n6-PUFA, C18:2n6, C20:2n6, C20:3n6, C20:4n6, C22:4n6, C22:5n6, C18:3n3, C20:5n3, C22:5n3, C22:6n3, LCn-3PUFA, Omega-3 index, t16:1n7, t18:1n9	CHD (136)
Chei 2018[25]	NCC	CIRCS	Japan	GP free of stroke or CAD	608	11.0/21.0	66	39	1984-1998	serum	(n=17) even-chain SFA, C14:0, C16:0, C18:0, total MUFA, C16:1n7, C18:1n9, total n-6 PUFA, C18:2n6, C18:3n6, C20:3n6, C20:4n6, total n-3 PUFA, C18:3n3, C20:5n3, C22:5n3, C22:6n3	CAD (152)
Harris 2018[78]	PC	FHS-Offspring	US	GP free of prevalent CVD	2500	7.3/9.5	66	57	2005-2008	RBC	(n=8) total n-6 PUFA, C18:2n6, C18:3n6, C20:2n6, C20:3n6, C20:4n6, C22:4n6, C22:5n6	CHD (119)<br>IS (105)
Harris 2018[50]	PC	FHS-Offspring	US	GP free of CVD	2500	7.3/11.2	66	57	2005-2008	RBC	(n=6) C18:3n3, C20:5n3, C22:5n3, C22:6n3, Omega-3 index, LCn-3PUFA	CHD (119)<br>IS (105)
Otto 2018[35]	PC	CHS	US	GP free of CVD	2907	12.9†/22.0	75	64	1992-1993	plasma PL	(n=3) C15:0, C17:0, t16:1n7	CHD (876)<br>stroke (529)
Satizabal 2018[72] ♣	PC	FHS	US	GP free of CHD or stroke	943	9.1†/10.0	74	63	1985-1988	plasma PL	(n=4) C16:0, C22:6n3, C18:2n6, C20:4n6	CHD (130)<br>stroke (81)
	PC	3C Study	France		1406	8.1†/10.0	75	61	1999-2001	plasma		CHD (53)<br>stroke (51)
Virtanen 2018[73]	PC	KIHD	Finland	Males free of CVD, cancer, or diabetes	1461	23.6†/31.0	52	0	1984-1989	serum	(n=4) C18:2n6, C18:3n6, C20:3n6, C20:4n6	fatal CHD (145)
Hamazaki 2017[56]	NCC	JPHC	Japan	GP free of stroke, IHD or cancer	627	15.0/18.0	57	36	1990-1993	plasma PL	(n=5) C20:5n3, C22:5n3, C22:6n3, LCn-3PUFA, Omega-3 index	CHD (209)
	PC	CHS			3675	11.2/19.0	75	60	1992-1993	plasma PL		IS (516)
Saber 2017[59]	NCC	NHS	US	GP free of stroke or TIA	714	8.3/16.0	61	100	1989-1990	RBC	(n=3) C20:5n3, C22:5n3, C22:6n3	IS (357)
	NCC	HPFS			160		68	0	1993-1994	RBC		IS (80)
Daneshmand 2016[58] ♣	PC	KIHD	Finland	Males free of CVD	1828	21.2†/28.8	52	0	1984-1989	serum	(n=11) total n-3 PUFA, C18:3n3, LCn-3PUFA, C20:5n3, C22:5n3, C22:6n3, total n-6 PUFA, C18:2n6, C18:3n6, C20:3n6, C20:4n6	stroke (202)
Del Gobbo 2016[105]	PC	3C Study	France	GP free of MI, angina, coronary revascularization, or stroke	1416	6.6/8.5	75	61	1999-2000	plasma	(n=5) C18:3n3, C20:5n3, C22:5n3, C22:6n3, LCn-3PUFA	CHD (57)
	PC	ARIC	US		3793	22.4/25.1	54	52	1987-1989	plasma PL		CHD (398)
	PC	CHS	US		3941	21.6 (max)	74	60	1992-1993	plasma PL		CHD (1179)
	NCC	HPFS	US		1291	4.0/15.0	64	0	1994	plasma RBC§		CHD (466)
	NCC	InCHIANTI	Italy		839	9.0/10.0	65	55	1998-2000	plasma		CHD (115)
	PC	KIHD	Finland		1837	22.9/27.8	52	0	1984-1989	serum		CHD (452)
	PC	MCCS	Australia		5279	9.7/22.1	56	51	1990-1994	plasma PL		fatal CHD (202)
	PC	MESA	US		2856	8.5/10.9	62	53	2000-2002	plasma PL		CHD (94)
	NCC	NHS I	US		1040	16.0 (max)	60	100	1989-1990	plasma RBC§		CHD (437)
	NCC	NSHDS I‡	Sweden		183	1.3/4.3	55	21	1987-1994	plasma PL		CHD (64)
	NCC	NSHDS II‡	Sweden		759	3.7/10.8	55	38	1987-1999	plasma PL		CHD (353)
	NCC	PHS	US		2000	NA	69	0	1995-2001	RBC		CHD (416)
	NCC	SCHS	Singapore		1555	4.6/14.4	66	35	1994-2005	plasma		CHD (759)

	PC	SHHEC	Scotland		4391	23.4/24.8	49	48	1984-1987	AT		CHD (898)
	PC	ULSAM 50	Sweden		2001	30.0/42.0	50	0	1970	serum CE		CHD (749)
	PC	ULSAM 70	Sweden		752	14.0/20.0	71	0	1990	AT		CHD (206)
Fretts 2016[37]	PC	CHS	US	GP	3941	11.5†/20.0	75	59	1992–1993	plasma PL	(n=4) C16:0, C18:0, C22:0, C24:0	fatal CHD (788)
Sun 2016[55]	NCC	SCHS	Singapore	GP free of CAD or stroke	1488	17.0 (max)	66	35	1994-2005	plasma	(n=4) C18:3n3, C20:5n3, C22:6n3, Omega-3 index	AMI (744)
Sun 2016[27]	NCC	SCHS	Singapore	GP free of CHD or stroke	1488	17.0 (max)	66	35	1994-2005	plasma	(n=10) C16:0, C18:0, C16:1n7, C18:1n9, C18:2n6, C20:4n6, C20:5n3, C22:6n3, Omega-3 index, trans-18:1	AMI (744)
Malik 2015[26]	NCC	HPFS	US	Males free of cancer and CVD	1265	15.0 (max)	64	0	1993-1995	plasma RBC§	(n=4) C20:0, C22:0, C24:0, longer-chain SFA (C20:0+C22:0+C24:0)	CHD (446)
		NHS		Female nurses free of cancer or CVD	762	17.0 (max)	60	100	1989-1990			CHD (348)
Fretts 2014[64] ♣	PC	CHS	US	GP free of CVD	2709	11.3†/16.0	73.0*	71	1992-1993	plasma PL	(n=1) C18:3n3	CHD (426) stroke (430)
Matthan 2014[76]	NCC	WHI-OS	US	Postmenopausal women free of CVD	2448	4.5†/12.0	68	100	1993-1998	plasma PL	(n=32) total SFA, C12:0, C14:0, C15:0, C16:0, C18:0, C20:0, C22:0, C24:0, total MUFA, C16:1n7, C16:1n9, C18:1n7, C18:1n9, C20:1n9, C24:1n9, total n-6 PUFA, C18:2n6, C18:3n6, C20:2n6, C20:3n6, C20:4n6, C22:4n6, C22:5n6, total n-3 PUFA, C18:3n3, C20:5n3, C22:5n3, C22:6n3, total trans, t18:1, t18:2	CHD (1224)
Wu 2014[77] ♣	PC	CHS	US	GP free of CVD	2792	12.3†/18.0	74	64	1992-1993	plasma PL	(n=4) C18:2n6, C18:3n6, C20:3n6, C20:4n6	CHD (720) stroke (463)
Yakoob 2014[34] ♣	NCC	HPFS	US	Males free of CVD or cance	244	8.3/17.0	68	0	1993-1994	plasma RBC§	(n=4) C14:0, C15:0, C17:0, t16:1n7	stroke (122)
		NHS		Female nurses free of CVD or cancer	944		61	100	1989-1990			stroke (472)
De Goede 2013[75]	NCC	MP-CVDRF	Netherlands	GP free of MI or stroke	444	12.5/19.0	51	30	1987-1991	plasma CE	(n=6) C18:2n6, C20:4n6, C18:3n3, C20:5n3, C22:6n3, Omega-3 index	fatal CHD (222)
Matsumoto 2013[54]	NCC	PHS	US	Males free of CHD	2000	NR	69	0	1995-2001	RBC	(n=6) C18:3n3, C20:5n3, C22:5n3, C22:6n3, LCn-3PUFA, C18:4n3	CHD (1000)
Mozaffarian 2013[62] ♣	PC	CHS	US	GP free of CHD, stroke, or heart failure	2692	11.5†/16.0	74	64	1992-1993	plasma PL	(n=4) C20:5n3, C22:5n3, C22:6n3, LCn-3PUFA	CHD (630) stroke (406)
Otto 2013[96]	PC	MESA	US	GP free of CVD	2837	7.0†/10.0	62	53	2000-2002	plasma PL	(n=3) C14:0, C15:0, t16:1n7	CHD (146)
Otto 2013[63]	PC	MESA	US	GP free of CVD	2837	7.0†/10.0	62	53	2000-2002	plasma PL	(n=7) C20:5n3, C22:5n3, C22:6n3, LCn-3PUFA, C18:2n6, C20:4n6, C18:3n3	CHD (141)
Yaemsiri 2013[32]	NCC	WHI-OS	US	Postmenopausal women free of stroke	1928	10.0 (max)	69	100	1993-1998	serum	(n=25) C14:0, C15:0, C16:0, C17:0, C18:0, C16:1n7, C18:1n7, C18:1n9, C20:1n9, C24:1n9, C18:3n3, C20:4n3, C20:5n3, C22:5n3, C22:6n3, C18:2n6, C18:3n6, C20:2n6, C20:3n6, C20:4n6, C22:4n6, C22:5n6, t16:1, t18:1, t18:2	IS (964)
Yamagishi 2013[33]	PC	ARIC	US	GP free of a self-reported history of stroke or TIA	3870	19.9/22.1	54*	52	1987-1989	plasma PL plasma CE§	(n=18) total SFA, C14:0, C15:0, C16:0, C18:0, total MUFA,C16:1, C18:1, total n-6 PUFA, C18:2n6, C18:3n6, C20:3n6, C20:4n6, total n-3 PUFA, C18:3n3, LCn-3PUFA, C20:5n3, C22:6n3	IS (168)
Malik 2012[38,95]¶	NCC	HPFS	US	Males	1337	15 (max)	NR	0	1993-1994	RBC plasma§¶	(n=2) C15:0, C17:0	CHD (459)
Woodward 2011[36]	PC	SHHEC	Scotland	GP free of CVD	3944	19.5/22.0	49	47	1984-1987	AT	(n=17) even-chain SFA, C14:0, C16:0, C18:0, total MUFA, C16:1n7, C18:1n9, C20:1n9, total PUFA, LCn-3PUFA (DPA+DHA), C22:5n3, C22:6n3, total n-6 PUFA, C18:2n6, C18:3n6, C20:3n6, C20:4n6	CHD (651) stroke (219)
Wu 2011[42]	PC	CHS	US	GP free of CHD	2890	10.3†/14.0	74	63	1992-1993	plasma PL	(n=4) C16:0, C16:1n7, C16:1n9, C18:1n7	CHD (631)
Warensjö 2010[41]	NCC	NSHDS‡	Northern Sweden	GP free of prior MI, stroke, or cancer	1000	3.7†/13.0	60*	39	1987-1999	plasma PL	(n=3) odd-chain SFA (C15:0+C17:0), C15:0, C17:0	MI (444)
Clarke 2009[24]	NCC	Whitehall	UK	Males free of CHD, stroke, or statin use	355	6.8†/8.4	79	0	1997-1998	plasma PL	(n=3) total SFA, total MUFA, total PUFA	fatal CHD (116)
Warensjö 2009[31]	NCC	VIP +MONICA‡	Northern Sweden	GP free of AMI, stroke or cancer	324	3.0†/12.0	60*	38	1985-1996	plasma PL plasma CE§	(n=3) odd-chain SFA (C15:0+C17:0), C15:0, C17:0	stroke (108)

Sun 2008[52]	NCC	NHS	US	Female nurses free of diagnosed cancer or CVD	434	6.0 (max)	60	100	1989-1990	plasma RBC§	(n=5) C18:3n3, C20:5n3, C22:5n3, C22:6n3, LCn-3PUFA	non-fatal MI (146)
Sun 2007[40]	NCC	NHS	US	Female nurses free of cancers or CVD	493	7.0 (max)	60	100	1989-1990	plasma RBC§	(n=3) C15:0, C17:0, t16:1n7	CHD (166)
Sun 2007[128]	NCC	NHS	US	Female nurses free of cancers or CVD	493	6.0 (max)	60	100	1989-1990	RBC	(n=3) total trans, t18:1, t18:2	CHD (166)
Wennberg 2007[122]	NCC	VIP +MONICA‡	Northern Sweden	GP free of AMI, stroke or cancer	1107	5.8 (max)	55	40	1985-1994	plasma PL & RBC (mixed)	(n=1) Omega-3 index	stroke (369)
Lemaitre 2006[126]	NCC	CHS	US	GP	428	3.0†/6.0	77	40	1992-1993	plasma PL	(n=4) Total trans, t16:1, t18:1, t18:2	fatal CHD (214)
Wiberg 2006[30] ♣	PC	ULSAM 50	Sweden	Males free of stroke or TIA	2313	29.3/32.7	50	0	1970-1973	serum CE	(n=12) C14:0, C16:0, C18:0, C16:1, C18:1, C18:3n3, C20:5n3, C22:6n3, C18:2n6, C18:3n6, C20:3n6, C20:4n6	stroke (421)
Lemaitre 2003[51]	NCC	CHS	US	GP free of IHD and stroke	250<br>108	1.8/4.0	75<br>78	36<br>43	1992-1993	plasma PL	(n=3) C18:2n6, C18:3n3, Omega-3 index	non-fatal MI (125)<br>fatal IHD (54)
Iso 2002[29] ♣	NCC	JapanCRS	Japan	GP	788	15.0 (max)	65	47	1984-1993	serum	(n=7) even-chain SFA (C14+C16+C18), C14:0, C16:0, C16:1, C18:1, C18:2n6, C20:4n6	stroke (197)
Hallgren 2001[127]	NCC	VIP +MONICA‡	Northern Sweden	GP free of AMI or stroke	234	1.3/9.0	55	21	1985-1994	plasma PL	(n=1) Omega-3 index	MI (78)
Guallar 1995[138]	NCC	PHS	US	Males free of MI, stroke, cancer, severe debilitating disease	426	5.0 (max)	59	0	1982-1984	plasma PL plasma CE§	(n=3) C20:5n3, C22:6n3, Omega-3 index	MI (213)
Simon 1995[121]	NCC	MRFIT	US	Males with high TC, DBP, and smoking	192	6.9†	50	0	1973-1976	serum PL serum CE§	(n=16) C14:0, C16:0, C18:0, C16:1, C18:1, C18:3n3, C20:5n3, C22:5n3, C22:6n3, C18:2n6, C20:2n6, C20:3n6, C20:4n6, C22:4n6, C22:5n6, C20:3n9	stroke (96)
Simon 1995[39]	NCC	MRFIT	US	Males with high TC, DBP, and smoking	188	6.9†	50	0	1973-1976	serum PL serum CE§	(n=16) C14:0, C16:0, C18:0, C16:1, C18:1, C18:2n6, C20:2n6, C20:3n6, C20:4n6, C22:4n6, C22:5n6, C18:3n3, C20:5n3, C22:5n3, C22:6n3, C20:3n9	CHD (94)
**Studies identified from additionally manual search (n=2)**												
Tikkanen 2021[149]	PC	FINRISK(97 /02/07/12) & Health 2000	Finland	GP free of CVD	31657	14.0/20.0	50	53	1997,2002, 2007,2012, 2000-2001	serum	(n=7) total SFA, total MUFA, total PUFA, total n-3 PUFA, total n-6 PUFA, C22:6n3, C18:2n6	CAD (2073)
Holmes 2018[150]	NCC	CKB	China	GP free of CHD, stroke, TIA, cancer, or statin use	2378<br>2612	11.0 (max)	45	47<br>53	2004-2008	plasma	(n=7) total SFA, total MUFA, total PUFA, total n-3 PUFA, total n-6 PUFA, C22:6n3, C18:2n6	MI (912)<br>IS (1146)

AMI: Acute myocardial infarction; AT: Adipose tissue; CAD: Coronary artery disease; CE: Cholesteryl ester; CHD: Coronary heart disease; CVD: Cardiovascular disease; DBP: Diastolic blood pressure; GP: General population; IHD: Ischemic heart disease; IS: Ischemic stroke; LCn-3PUFA: Long-chain n-3 polyunsaturated fatty acids; MI: Myocardial infarction; MUFA: Monounsaturated fatty acids; NCC: Nested case-control study; PC: Prospective cohort; PL: Phospholipids; PUFA: Polyunsaturated fatty acids; RBC: Red blood cell (or erythrocyte); SFA: Saturated fatty acids; TC: Total cholesterol; TIA: Transient ischemic attack; UK: United Kingdom; US: United States.

Study abbreviations: 3C Study: Three City Study; 60YO: The Stockholm Cohort of 60-year-olds; AGES-Reykjavik: Age, gene/environment susceptibility-Reykjavik Study; ARIC: Atherosclerosis Risk in Communities; CCCC: Chin-Shan Community Cardiovascular Cohort Study; CHS: Cardiovascular Health Study; CIRCS: Circulatory Risk in Communities Study; CKB: China Kadoorie Biobank; DCH: Diet, Cancer and Health cohort; FHS: Framingham Heart Study; FHS-Offspring: Framingham Heart Study Offspring Cohort; HPFS: Health Professionals Follow-up Study; HS: The Hisayama Study; InCHIANTI: Invecchiare in Chianti; JapanCRS: Japanese cardiovascular risk surveys; JPHC: Japan Public Health Center-based study; KIHD: Kuopio Ischemic Heart Disease Risk Factor Study; MCCS: Melbourne Collaborative Cohort Study; MESA: Multi-Ethnic Study of Atherosclerosis; MP-CVDRF: Monitoring Project on Cardiovascular Disease Risk Factors; MRFIT: Multiple Risk Factor Intervention Trial; NHS: Nurses' Health Study; NSHDS: Northern Sweden Health and Disease Study; PHS: Physicians' health study; PIVUS: Prospective Investigation of the Vasculature in Uppsala Seniors; PREDIMED: Prevención con Dieta Mediterránea trial; SCHS: Singapore Chinese Health Study; SHHEC: Scottish Heart Health Extended Cohort; ULSAM 50: Uppsala Longitudinal Study of Adult Men investigations at ages 50 y; ULSAM 70: Uppsala Longitudinal Study of Adult Men investigations at ages 70 y; VIP+MONICA: Västerbotten Intervention Program + Monitoring of Trends and Determinants in Cardiovascular disease survey; WHI-MS: Women's Health Initiative Memory Study; WHI-OS: Women's Health Initiative Observational Study; Whitehall: Whitehall study of London civil servants.

* Median ages. † Mean follow-up years.

‡ These data sources also included the VIP study, which was also part of our EPIC-CVD study in Sweden; however, these studies were still included in the updated meta-analysis given the small overlap.

§ Fatty acids were also assayed in this compartment though not chosen in the updated meta-analysis.

¶ Malik 2012 was published as an abstract but the estimates were reported in the meta-analysis paper of Trieu 2021, which were also extracted in our updated meta-analysis.

♣ Studies also reported estimates on ischaemic stroke and further details were summarized in Supplementary eTable 20.

Supplementary eTable 18. Further characteristics of non-duplicated associations of fatty acid biomarkers with CHD included in the updated meta-analyses

Paper	Data source	Region	Biomarker	Outcome	N of events	N	reported RR (95%CI)	Scale of RR reported by study	Conversion factor	RR (95%CI) per 1-SD	Adjustment
**Total SFA**											
Tikkanen 2021	FINRISK & Health	Finland	serum	CAD	2073	31657	1.14 (1.10, 1.18)	per SD increase	1.00	1.14 (1.10, 1.18)	+
Papandreou 2019	PREDIMED	Spain	RBC	CHD	136	408	0.87 (0.68, 1.11)	per SD increase	1.00	0.87 (0.68, 1.11)	++
Holmes 2018	CKB	China	plasma	MI	912	2378	0.94 (0.85, 1.04)	per SD increase	1.00	0.94 (0.85, 1.04)	+++
Matthan 2014	WHI-OS	US	plasma PL	CHD	1224	2448	1.20 (1.08, 1.32)	per unit increase (SD=1.4)	1.40	1.29 (1.11, 1.47)	++
Clarke 2009	Whitehall	UK	plasma PL	fatal CHD	116	355	2.04 (1.06, 3.93)	Highest versus lowest fourth	0.39	1.32 (1.02, 1.71)	++
**Even-chain SFA**											
Chei 2018	CIRCS	Japan	serum	CAD	152	608	1.43 (1.11, 1.83)	per SD increase	1.00	1.43 (1.11, 1.83)	++
Woodward 2011	SHHEC	Scotland	AT	CHD	651	3944	0.84 (0.67, 1.06)	Highest versus lowest fourth	0.39	0.93 (0.85, 1.02)	+++
**Myristic acid (14:0)**											
Papandreou 2019	PREDIMED	Spain	RBC	CHD	136	408	0.74 (0.55, 0.98)	per SD increase	1.00	0.74 (0.55, 0.98)	++
Chei 2018	CIRCS	Japan	serum	CAD	152	608	1.34 (1.10, 1.64)	per SD increase	1.00	1.34 (1.10, 1.64)	++
Matthan 2014	WHI-OS	US	plasma PL	CHD	1224	2448	1.05 (0.70, 1.56)	per unit increase (SD=0.35)	0.35	1.02 (0.88, 1.17)	++
Otto 2013	MESA	US	plasma PL	CHD	146	2837	1.01 (0.84, 1.21)	per SD increase	1.00	1.01 (0.84, 1.21)	++
Woodward 2011	SHHEC	Scotland	AT	CHD	651	3944	0.88 (0.70, 1.10)	Highest versus lowest fourth	0.39	0.95 (0.87, 1.04)	+++
Simon 1995	MRFIT	US	serum PL	CHD	94	188	1.10 (0.79, 1.54)	per SD increase	1.00	1.10 (0.79, 1.54)	+
**Palmitic acid (16:0)**											
Papandreou 2019	PREDIMED	Spain	RBC	CHD	136	408	0.93 (0.74, 1.18)	per SD increase	1.00	0.93 (0.74, 1.18)	++
Satizabal 2018	3C Study	France	plasma	CHD	53	1174	1.08 (0.79, 1.47)	per SD increase	1.00	1.08 (0.79, 1.47)	++
Chei 2018	CIRCS	Japan	serum	CAD	152	608	1.49 (1.16, 1.91)	per SD increase	1.00	1.49 (1.16, 1.91)	++
Satizabal 2018	FHS	US	plasma PL	CHD	130	772	1.21 (1.02, 1.44)	per SD increase	1.00	1.21 (1.02, 1.44)	++
Fretts 2016	CHS	US	plasma PL	fatal CHD	788	3941	1.00 (0.79, 1.27)	Highest versus lowest fifth	0.36	1.00 (0.92, 1.09)	++
Sun 2016	SCHS	Singapore	plasma	AMI	744	1488	1.03 (0.89, 1.18)	per SD increase	1.00	1.03 (0.89, 1.18)	++
Matthan 2014	WHI-OS	US	plasma PL	CHD	1224	2448	3.26 (0.51, 20.81)	per unit increase (SD=2.1)	2.10	11.95 (0.24, 585.11)	++
Woodward 2011	SHHEC	Scotland	AT	CHD	651	3944	0.92 (0.73, 1.17)	Highest versus lowest fourth	0.39	0.97 (0.88, 1.06)	+++
Simon 1995	MRFIT	US	serum PL	CHD	94	188	1.30 (0.93, 1.80)	per SD increase	1.00	1.30 (0.93, 1.80)	+
**Stearic acid (18:0)**											
Papandreou 2019	PREDIMED	Spain	RBC	CHD	136	408	0.97 (0.77, 1.23)	per SD increase	1.00	0.97 (0.77, 1.23)	++
Chei 2018	CIRCS	Japan	serum	CAD	152	608	0.92 (0.70, 1.22)	per SD increase	1.00	0.92 (0.70, 1.22)	++
Fretts 2016	CHS	US	plasma PL	fatal CHD	788	3941	1.08 (0.85, 1.36)	Highest versus lowest fifth	0.36	1.03 (0.95, 1.12)	++
Sun 2016	SCHS	Singapore	plasma	AMI	744	1488	0.66 (0.47, 0.92)	per SD increase	1.00	0.66 (0.47, 0.92)	++
Matthan 2014	WHI-OS	US	plasma PL	CHD	1224	2448	1.57 (0.53, 4.61)	per unit increase (SD=1.4)	1.40	1.88 (0.41, 8.49)	++
Woodward 2011	SHHEC	Scotland	AT	CHD	651	3944	0.86 (0.69, 1.08)	Highest versus lowest fourth	0.39	0.94 (0.86, 1.03)	+++
Simon 1995	MRFIT	US	serum PL	CHD	94	188	0.99 (0.72, 1.35)	per SD increase	1.00	0.99 (0.72, 1.35)	+
**Odd-chain SFA**											
Warensjö 2010	NSHDS	Sweden	plasma PL	MI	444	1000	0.85 (0.73, 0.98)	per SD increase	1.00	0.85 (0.73, 0.98)	+
**Pentadecanoic acid (15:0)**											
Trieu 2021	60YO	Sweden	serum CE	CHD	386	4150	0.86 (0.77, 0.96)	per SD increase	1.00	0.86 (0.77, 0.96)	++
Otto 2018	CHS	US	plasma PL	CHD	876	2907	1.18 (0.94, 1.48)	Highest versus lowest fifth	0.36	1.06 (0.98, 1.15)	+
Matthan 2014	WHI-OS	US	plasma PL	CHD	1224	2448	1.02 (0.61, 1.72)	per unit increase (SD=0.03)	0.03	1.00 (0.98, 1.02)	++
Otto 2013	MESA	US	plasma PL	CHD	146	2837	0.74 (0.60, 0.92)	per SD increase	1.00	0.74 (0.60, 0.92)	++
Malik 2012^	HPFS	US	RBC	CHD	458	1337	0.83 (0.54, 1.28)	Highest versus lowest third	0.46	0.92 (0.75, 1.12)	NR
Warensjö 2010	NSHDS	Sweden	plasma PL	MI	444	1000	0.87 (0.76, 1.00)	per SD increase	1.00	0.87 (0.76, 1.00)	+
Sun 2007	NHS	US	plasma	CHD	166	493	1.39 (0.85, 2.26)	Highest versus lowest third	0.46	1.16 (0.93, 1.46)	++
**Heptadecanoic acid (17:0)**											
Otto 2018	CHS	US	plasma PL	CHD	876	2907	0.90 (0.72, 1.12)	Highest versus lowest fifth	0.36	0.96 (0.89, 1.04)	+
Malik 2012^	HPFS	US	RBC	CHD	458	1337	1.10 (0.74, 1.64)	Highest versus lowest third	0.46	1.04 (0.87, 1.25)	NR

Warensjö 2010	NSHDS	Sweden	plasma PL	MI	444	1000	0.86 (0.74, 0.99)	per SD increase	1.00	0.86 (0.74, 0.99)	+
Sun 2007	NHS	US	plasma	CHD	166	493	0.94 (0.59, 1.48)	Highest versus lowest third	0.46	0.97 (0.79, 1.20)	++
**Longer-chain SFA**											
Papandreou 2019	PREDIMED	Spain	RBC	CHD	136	408	0.45 (0.29, 0.70)	per SD increase	1.00	0.45 (0.29, 0.70)	++
Liu 2019	WHI-OS	US	plasma PL	CHD	1214	2428	1.00 (0.77, 1.30)	per unit increase (SD=0.46)	0.46	1.00 (0.89, 1.13)	++
Malik 2015	HPFS	US	plasma	CHD	446	1265	0.55 (0.33, 0.93)	Highest versus lowest fifth	0.36	0.81 (0.67, 0.97)	++
Malik 2015	NHS	US	plasma	CHD	348	762	0.36 (0.19, 0.68)	Highest versus lowest fifth	0.36	0.69 (0.55, 0.87)	++
**Arachidic acid (20:0)**											
Papandreou 2019	PREDIMED	Spain	RBC	CHD	136	408	1.01 (0.81, 1.25)	per SD increase	1.00	1.01 (0.81, 1.25)	++
Malik 2015	HPFS	US	plasma	CHD	446	1265	0.62 (0.37, 1.02)	Highest versus lowest fifth	0.36	0.84 (0.70, 1.01)	++
Malik 2015	NHS	US	plasma	CHD	348	762	0.47 (0.25, 0.89)	Highest versus lowest fifth	0.36	0.76 (0.61, 0.96)	++
Matthan 2014	WHI-OS	US	plasma PL	CHD	1224	2448	0.99 (0.60, 1.62)	per unit increase (SD=0.07)	0.07	1.00 (0.96, 1.03)	++
**Behenic acid (22:0)**											
Papandreou 2019	PREDIMED	Spain	RBC	CHD	136	408	0.44 (0.28, 0.69)	per SD increase	1.00	0.44 (0.28, 0.69)	++
Fretts 2016	CHS	US	plasma PL	fatal CHD	788	3941	0.88 (0.70, 1.11)	Highest versus lowest fifth	0.36	0.96 (0.88, 1.04)	++
Malik 2015	HPFS	US	plasma	CHD	446	1265	0.76 (0.45, 1.30)	Highest versus lowest fifth	0.36	0.91 (0.75, 1.10)	++
Malik 2015	NHS	US	plasma	CHD	348	762	0.36 (0.19, 0.68)	Highest versus lowest fifth	0.36	0.69 (0.55, 0.87)	++
Matthan 2014	WHI-OS	US	plasma PL	CHD	1224	2448	1.35 (0.90, 2.01)	per unit increase (SD=0.35)	0.35	1.11 (0.96, 1.28)	++
**Lignoceric acid (24:0)**											
Papandreou 2019	PREDIMED	Spain	RBC	CHD	136	408	0.41 (0.25, 0.65)	per SD increase	1.00	0.41 (0.25, 0.65)	++
Fretts 2016	CHS	US	plasma PL	fatal CHD	788	3941	0.81 (0.65, 1.02)	Highest versus lowest fifth	0.36	0.93 (0.86, 1.01)	++
Malik 2015	HPFS	US	plasma	CHD	446	1265	0.54 (0.32, 0.91)	Highest versus lowest fifth	0.36	0.80 (0.67, 0.97)	++
Malik 2015	NHS	US	plasma	CHD	348	762	0.40 (0.21, 0.77)	Highest versus lowest fifth	0.36	0.72 (0.57, 0.91)	++
Matthan 2014	WHI-OS	US	plasma PL	CHD	1224	2448	1.22 (0.83, 1.80)	per unit increase (SD=0.35)	0.35	1.07 (0.94, 1.23)	++
**Total MUFA**											
Tikkanen 2021	FINRISK & Health	Finland	serum	CAD	2073	31657	1.26 (1.21, 1.31)	per SD increase	1.00	1.26 (1.21, 1.31)	+
Papandreou 2019	PREDIMED	Spain	RBC	CHD	136	408	1.31 (1.04, 1.65)	per SD increase	1.00	1.31 (1.04, 1.65)	++
Chei 2018	CIRCS	Japan	serum	CAD	152	608	1.30 (1.05, 1.62)	per SD increase	1.00	1.30 (1.05, 1.62)	++
Holmes 2018	CKB	China	plasma	MI	912	2378	1.18 (1.07, 1.30)	per SD increase	1.00	1.18 (1.07, 1.30)	+++
Matthan 2014	WHI-OS	US	plasma PL	CHD	1224	2448	0.97 (0.91, 1.04)	per unit increase (SD=1.75)	1.75	0.95 (0.85, 1.07)	++
Woodward 2011	SHHEC	Scotland	AT	CHD	651	3944	1.21 (0.96, 1.53)	Highest versus lowest fourth	0.39	1.08 (0.98, 1.18)	+++
Clarke 2009	Whitehall	UK	plasma PL	fatal CHD	116	355	1.00 (0.52, 1.93)	Highest versus lowest fourth	0.39	1.00 (0.77, 1.29)	++
**Palmitoleic acid (16:1n7)**											
Papandreou 2019	PREDIMED	Spain	RBC	CHD	136	408	1.11 (0.89, 1.37)	per SD increase	1.00	1.11 (0.89, 1.37)	++
Chei 2018	CIRCS	Japan	serum	CAD	152	608	1.46 (1.16, 1.85)	per SD increase	1.00	1.46 (1.16, 1.85)	++
Sun 2016	SCHS	Singapore	plasma	AMI	744	1488	1.04 (0.92, 1.17)	per SD increase	1.00	1.04 (0.92, 1.17)	++
Matthan 2014	WHI-OS	US	plasma PL	CHD	1224	2448	1.06 (0.76, 1.47)	per unit increase (SD=0.35)	0.35	1.02 (0.91, 1.14)	++
Wu 2011	CHS	US	plasma PL	CHD	631	2890	0.89 (0.58, 1.37)	per interquintile range (10-90th)	0.39	0.96 (0.81, 1.13)	++
Woodward 2011	SHHEC	Scotland	AT	CHD	651	3944	1.23 (0.98, 1.53)	Highest versus lowest fourth	0.39	1.08 (0.99, 1.18)	+++
Simon 1995	MRFIT	US	serum PL	CHD	94	188	1.08 (0.77, 1.51)	per SD increase	1.00	1.08 (0.77, 1.51)	+
**Oleic acid (18:1n9)**											
Papandreou 2019	PREDIMED	Spain	RBC	CHD	136	408	1.36 (1.08, 1.72)	per SD increase	1.00	1.36 (1.08, 1.72)	++
Chei 2018	CIRCS	Japan	serum	CAD	152	608	1.18 (0.96, 1.46)	per SD increase	1.00	1.18 (0.96, 1.46)	++
Sun 2016	SCHS	Singapore	plasma	AMI	744	1488	1.14 (1.01, 1.28)	per SD increase	1.00	1.14 (1.01, 1.28)	++
Matthan 2014	WHI-OS	US	plasma PL	CHD	1224	2448	0.71 (0.34, 1.46)	per unit increase (SD=1.4)	1.40	0.62 (0.22, 1.70)	++
Woodward 2011	SHHEC	Scotland	AT	CHD	651	3944	1.23 (0.97, 1.56)	Highest versus lowest fourth	0.39	1.08 (0.99, 1.19)	+++
Simon 1995	MRFIT	US	serum PL	CHD	94	188	1.26 (0.90, 1.77)	per SD increase	1.00	1.26 (0.90, 1.77)	+
**Eicosenoic acid (20:1n9)**											
Papandreou 2019	PREDIMED	Spain	RBC	CHD	136	408	1.58 (1.25, 2.00)	per SD increase	1.00	1.58 (1.25, 2.00)	++
Matthan 2014	WHI-OS	US	plasma PL	CHD	1224	2448	0.85 (0.62, 1.17)	per unit increase (SD=0.03)	0.03	0.99 (0.98, 1.01)	++
Woodward 2011	SHHEC	Scotland	AT	CHD	651	3944	0.98 (0.79, 1.22)	Highest versus lowest fourth	0.39	0.99 (0.91, 1.08)	+++

**Nervonic acid (24:1n9)**											
Papandreou 2019	PREDIMED	Spain	RBC	CHD	136	408	0.66 (0.50, 0.86)	per SD increase	1.00	0.66 (0.50, 0.86)	++
Matthan 2014	WHI-OS	US	plasma PL	CHD	1224	2448	1.12 (0.78, 1.61)	per unit increase (SD=0.35)	0.35	1.04 (0.92, 1.18)	++
**Total PUFA**											
Tikkanen 2021	FINRISK & Health	Finland	serum	CAD	2073	31657	0.81 (0.78, 0.84)	per SD increase	1.00	0.81 (0.78, 0.84)	+
Holmes 2018	CKB	China	plasma	MI	912	2378	0.90 (0.82, 0.99)	per SD increase	1.00	0.90 (0.82, 0.99)	+++
Woodward 2011	SHHEC	Scotland	AT	CHD	651	3944	0.85 (0.68, 1.08)	Highest versus lowest fourth	0.39	0.94 (0.86, 1.03)	+++
Clarke 2009	Whitehall	UK	plasma PL	fatal CHD	116	355	0.49 (0.25, 0.95)	Highest versus lowest fourth	0.39	0.76 (0.58, 0.98)	++
**Total N3 PUFA**											
Tikkanen 2021	FINRISK & Health	Finland	serum	CAD	2073	31657	0.96 (0.92, 1.00)	per SD increase	1.00	0.96 (0.92, 1.00)	+
Chei 2018	CIRCS	Japan	serum	CAD	152	608	0.99 (0.80, 1.22)	per SD increase	1.00	0.99 (0.80, 1.22)	++
Holmes 2018	CKB	China	plasma	MI	912	2378	0.91 (0.83, 1.01)	per SD increase	1.00	0.91 (0.83, 1.01)	+++
Matthan 2014	WHI-OS	US	plasma PL	CHD	1224	2448	0.89 (0.83, 0.97)	per unit increase (SD=1.75)	1.75	0.82 (0.72, 0.95)	++
**ALA (18:3n3)**											
Papandreou 2019	PREDIMED	Spain	RBC	CHD	136	408	1.16 (0.94, 1.43)	per SD increase	1.00	1.16 (0.94, 1.43)	++
Chei 2018	CIRCS	Japan	serum	CAD	152	608	0.84 (0.67, 1.06)	per SD increase	1.00	0.84 (0.67, 1.06)	++
Harris 2018	FHS-Offspring	US	RBC	CHD	119	2500	1.03 (0.55, 1.94)	Highest versus lowest fifth	0.36	1.01 (0.81, 1.27)	+++
Del Gobbo 2016	3C Study	France	plasma	CHD	57	1416	1.06 (0.20, 5.64)	per SD increase	1.00	1.06 (0.20, 5.64)	++++*
Del Gobbo 2016	ARIC	US	plasma PL	CHD	398	3793	1.06 (0.95, 1.18)	per SD increase	1.00	1.06 (0.95, 1.18)	++++*
Del Gobbo 2016	CHS	US	plasma PL	CHD	1179	3941	0.90 (0.83, 0.97)	per SD increase	1.00	0.90 (0.83, 0.97)	++++*
Del Gobbo 2016	HPFS	US	plasma	CHD	466	1291	1.51 (0.86, 2.66)	per SD increase	1.00	1.51 (0.86, 2.66)	++++*
Del Gobbo 2016	InCHIANTI	Italy	plasma	CHD	115	839	1.09 (0.92, 1.30)	per SD increase	1.00	1.09 (0.92, 1.30)	++++*
Del Gobbo 2016	KIHD	Finland	serum	CHD	452	1837	0.91 (0.81, 1.03)	per SD increase	1.00	0.91 (0.81, 1.03)	++++*
Del Gobbo 2016	MCCS	Australia	plasma PL	fatal CHD	202	5266	0.86 (0.72, 1.02)	per SD increase	1.00	0.86 (0.72, 1.02)	++++*
Del Gobbo 2016	NHS I	US	plasma	CHD	437	1040	1.17 (0.97, 1.40)	per SD increase	1.00	1.17 (0.97, 1.40)	++++*
Del Gobbo 2016	NSHDS I	Sweden	plasma PL	CHD	64	183	1.05 (0.67, 1.65)	per SD increase	1.00	1.05 (0.67, 1.65)	++++*
Del Gobbo 2016	NSHDS II	Sweden	plasma PL	CHD	353	759	1.23 (1.00, 1.51)	per SD increase	1.00	1.23 (1.00, 1.51)	++++*
Del Gobbo 2016	SCHS	Singapore	plasma	CHD	759	1555	0.84 (0.47, 1.51)	per SD increase	1.00	0.84 (0.47, 1.51)	++++*
Del Gobbo 2016	ULSAM 50	Sweden	serum CE	CHD	749	2001	1.00 (0.92, 1.08)	per SD increase	1.00	1.00 (0.92, 1.08)	++++*
Del Gobbo 2016	ULSAM 70	Sweden	AT	CHD	206	752	1.09 (0.74, 1.62)	per SD increase	1.00	1.09 (0.74, 1.62)	++++*
Matthan 2014	WHI-OS	US	plasma PL	CHD	1224	2448	0.77 (0.57, 1.05)	per unit increase (SD=0.07)	0.07	0.98 (0.96, 1.00)	++
Otto 2013	MESA	US	plasma PL	CHD	141	2837	1.18 (0.74, 1.91)	Highest versus lowest fourth	0.39	1.07 (0.89, 1.29)	++
De Goede 2013	MP–CVDRF	Netherlands	plasma CE	fatal CHD	222	444	1.01 (0.80, 1.26)	per SD increase	1.00	1.01 (0.80, 1.26)	++
Matsumoto 2013	PHS	US	RBC	CHD	1000	2000	1.04 (0.94, 1.16)	per SD increase	1.00	1.04 (0.94, 1.16)	++
Simon 1995	MRFIT	US	serum PL	CHD	94	188	0.86 (0.63, 1.17)	per SD increase	1.00	0.86 (0.63, 1.17)	+
**LCn-3PUFA**											
Papandreou 2019	PREDIMED	Spain	RBC	CHD	136	408	1.05 (0.84, 1.32)	per SD increase	1.00	1.05 (0.84, 1.32)	++
Harris 2018	FHS-Offspring	US	RBC	CHD	119	2500	0.54 (0.27, 1.05)	Highest versus lowest fifth	0.36	0.80 (0.63, 1.02)	+++
Hamazaki 2017	JPHC	Japan	plasma PL	CHD	209	627	0.79 (0.41, 1.51)	Highest versus lowest fourth	0.39	0.91 (0.71, 1.18)	++
Del Gobbo 2016	HPFS	US	plasma	fatal CHD	123	1291	0.90 (0.65, 1.25)	per SD increase	1.00	0.90 (0.65, 1.25)	++++*
Del Gobbo 2016	KIHD	Finland	serum	fatal CHD	168	1837	0.92 (0.77, 1.10)	per SD increase	1.00	0.92 (0.77, 1.10)	++++*
Del Gobbo 2016	MCCS	Australia	plasma PL	fatal CHD	202	5266	0.95 (0.82, 1.11)	per SD increase	1.00	0.95 (0.82, 1.11)	++++*
Del Gobbo 2016	NHS I	US	plasma	fatal CHD	44	461	0.61 (0.06, 6.16)	per SD increase	1.00	0.61 (0.06, 6.16)	++++*
Del Gobbo 2016	NSHDS II	Sweden	plasma PL	fatal CHD	80	759	0.37 (0.18, 0.76)	per SD increase	1.00	0.37 (0.18, 0.76)	++++*
Del Gobbo 2016	SCHS	Singapore	plasma	fatal CHD	292	1555	0.71 (0.57, 0.88)	per SD increase	1.00	0.71 (0.57, 0.88)	++++*
Del Gobbo 2016	ULSAM 50	Sweden	serum CE	fatal CHD	398	2001	0.99 (0.88, 1.11)	per SD increase	1.00	0.99 (0.88, 1.11)	++++*
Del Gobbo 2016	ULSAM 70	Sweden	AT	fatal CHD	72	666	0.86 (0.63, 1.18)	per SD increase	1.00	0.86 (0.63, 1.18)	++++*
Mozaffarian 2013	CHS	US	plasma PL	CHD	630	2692	0.72 (0.55, 0.95)	Highest versus lowest fifth	0.36	0.89 (0.81, 0.98)	++
Otto 2013	MESA	US	plasma PL	CHD	141	2837	0.45 (0.25, 0.82)	Highest versus lowest fourth	0.39	0.73 (0.58, 0.92)	++
Matsumoto 2013	PHS	US	RBC	CHD	1000	2000	0.97 (0.88, 1.07)	per SD increase	1.00	0.97 (0.88, 1.07)	++
Woodward 2011	SHHEC	Scotland	AT	CHD	651	3944	0.78 (0.62, 0.98)	Highest versus lowest fourth	0.39	0.91 (0.83, 0.99)	+++

Sun 2008	NHS	US	plasma	nonfatal MI	146	434	0.35 (0.18, 0.66)	Highest versus lowest fourth	0.39	0.66 (0.51, 0.85)	++
**EPA (20:5n3)**											
Papandreou 2019	PREDIMED	Spain	RBC	CHD	136	408	1.20 (0.97, 1.48)	per SD increase	1.00	1.20 (0.97, 1.48)	++
Chei 2018	CIRCS	Japan	serum	CAD	152	608	1.10 (0.90, 1.36)	per SD increase	1.00	1.10 (0.90, 1.36)	++
Harris 2018	FHS-Offspring	US	RBC	CHD	119	2500	0.69 (0.37, 1.29)	Highest versus lowest fifth	0.36	0.88 (0.70, 1.09)	+++
Hamazaki 2017	JPHC	Japan	plasma PL	CHD	209	627	0.84 (0.43, 1.64)	Highest versus lowest fourth	0.39	0.93 (0.72, 1.22)	++
Del Gobbo 2016	3C Study	France	plasma	CHD	57	1416	1.23 (0.77, 1.96)	per SD increase	1.00	1.23 (0.77, 1.96)	++++*
Del Gobbo 2016	ARIC	US	plasma PL	CHD	398	3793	1.02 (0.93, 1.12)	per SD increase	1.00	1.02 (0.93, 1.12)	++++*
Del Gobbo 2016	CHS	US	plasma PL	CHD	1163	3941	0.85 (0.67, 1.08)	per SD increase	1.00	0.85 (0.67, 1.08)	++++*
Del Gobbo 2016	HPFS	US	plasma	CHD	466	1291	0.72 (0.52, 1.00)	per SD increase	1.00	0.72 (0.52, 1.00)	++++*
Del Gobbo 2016	InCHIANTI	Italy	plasma	CHD	115	839	0.94 (0.79, 1.12)	per SD increase	1.00	0.94 (0.79, 1.12)	++++*
Del Gobbo 2016	KIHD	Finland	serum	CHD	452	1837	1.08 (0.98, 1.19)	per SD increase	1.00	1.08 (0.98, 1.19)	++++*
Del Gobbo 2016	MCCS	Australia	plasma PL	fatal CHD	202	5279	0.91 (0.78, 1.06)	per SD increase	1.00	0.91 (0.78, 1.06)	++++*
Del Gobbo 2016	NHS I	US	plasma	CHD	345	762	1.07 (0.93, 1.24)	per SD increase	1.00	1.07 (0.93, 1.24)	++++*
Del Gobbo 2016	NSHDS I	Sweden	plasma PL	CHD	64	183	0.73 (0.42, 1.28)	per SD increase	1.00	0.73 (0.42, 1.28)	++++*
Del Gobbo 2016	NSHDS II	Sweden	plasma PL	CHD	353	759	0.69 (0.55, 0.86)	per SD increase	1.00	0.69 (0.55, 0.86)	++++*
Del Gobbo 2016	SCHS	Singapore	plasma	CHD	759	1555	0.40 (0.23, 0.68)	per SD increase	1.00	0.40 (0.23, 0.68)	++++*
Del Gobbo 2016	ULSAM 50	Sweden	serum CE	CHD	749	2001	0.97 (0.88, 1.07)	per SD increase	1.00	0.97 (0.88, 1.07)	++++*
Del Gobbo 2016	ULSAM 70	Sweden	AT	CHD	181	666	1.19 (1.05, 1.33)	per SD increase	1.00	1.19 (1.05, 1.33)	++++*
Matthan 2014	WHI-OS	US	plasma PL	CHD	1224	2448	0.73 (0.58, 0.93)	per unit increase (SD=0.35)	0.35	0.90 (0.83, 0.97)	++
Otto 2013	MESA	US	plasma PL	CHD	141	2837	0.42 (0.23, 0.75)	Highest versus lowest fourth	0.39	0.71 (0.56, 0.90)	++
De Goede 2013	MP–CVDRF	Netherlands	plasma CE	fatal CHD	222	444	1.06 (0.87, 1.28)	per SD increase	1.00	1.06 (0.87, 1.28)	++
Matsumoto 2013	PHS	US	RBC	CHD	1000	2000	0.94 (0.85, 1.03)	per SD increase	1.00	0.94 (0.85, 1.03)	++
Simon 1995	MRFIT	US	serum PL	CHD	94	188	0.81 (0.55, 1.19)	per SD increase	1.00	0.81 (0.55, 1.19)	+
**DPA (22:5n3)**											
Papandreou 2019	PREDIMED	Spain	RBC	CHD	136	408	0.92 (0.73, 1.15)	per SD increase	1.00	0.92 (0.73, 1.15)	++
Chei 2018	CIRCS	Japan	serum	CAD	152	608	1.23 (0.98, 1.54)	per SD increase	1.00	1.23 (0.98, 1.54)	++
Harris 2018	FHS-Offspring	US	RBC	CHD	119	2500	0.60 (0.31, 1.15)	Highest versus lowest fifth	0.36	0.83 (0.66, 1.05)	+++
Hamazaki 2017	JPHC	Japan	plasma PL	CHD	209	627	0.96 (0.52, 1.78)	Highest versus lowest fourth	0.39	0.98 (0.77, 1.25)	++
Del Gobbo 2016	3C Study	France	plasma	CHD	57	1416	1.45 (0.63, 3.31)	per SD increase	1.00	1.45 (0.63, 3.31)	++++*
Del Gobbo 2016	ARIC	US	plasma PL	CHD	398	3793	0.89 (0.80, 0.99)	per SD increase	1.00	0.89 (0.80, 0.99)	++++*
Del Gobbo 2016	CHS	US	plasma PL	CHD	1163	3941	0.85 (0.67, 1.08)	per SD increase	1.00	0.85 (0.67, 1.08)	++++*
Del Gobbo 2016	HPFS	US	plasma	CHD	466	1291	0.83 (0.26, 2.66)	per SD increase	1.00	0.83 (0.26, 2.66)	++++*
Del Gobbo 2016	KIHD	Finland	serum	CHD	452	1837	0.93 (0.85, 1.03)	per SD increase	1.00	0.93 (0.85, 1.03)	++++*
Del Gobbo 2016	MCCS	Australia	plasma PL	fatal CHD	202	5279	0.99 (0.85, 1.15)	per SD increase	1.00	0.99 (0.85, 1.15)	++++*
Del Gobbo 2016	NHS I	US	plasma	CHD	437	1040	0.65 (0.35, 1.20)	per SD increase	1.00	0.65 (0.35, 1.20)	++++*
Del Gobbo 2016	NSHDS I	Sweden	plasma PL	CHD	64	183	0.57 (0.29, 1.12)	per SD increase	1.00	0.57 (0.29, 1.12)	++++*
Del Gobbo 2016	NSHDS II	Sweden	plasma PL	CHD	353	759	0.85 (0.70, 1.04)	per SD increase	1.00	0.85 (0.70, 1.04)	++++*
Del Gobbo 2016	SHHEC	Scotland	AT	CHD	898	4391	0.99 (0.92, 1.06)	per SD increase	1.00	0.99 (0.92, 1.06)	++++*
Del Gobbo 2016	ULSAM 70	Sweden	AT	CHD	194	722	1.08 (0.93, 1.27)	per SD increase	1.00	1.08 (0.93, 1.27)	++++*
Matthan 2014	WHI-OS	US	plasma PL	CHD	1224	2448	0.56 (0.33, 0.94)	per unit increase (SD=0.35)	0.35	0.82 (0.68, 0.98)	++
Otto 2013	MESA	US	plasma PL	CHD	141	2837	0.80 (0.48, 1.35)	Highest versus lowest fourth	0.39	0.92 (0.75, 1.12)	++
Matsumoto 2013	PHS	US	RBC	CHD	1000	2000	0.96 (0.87, 1.06)	per SD increase	1.00	0.96 (0.87, 1.06)	++
Simon 1995	MRFIT	US	serum PL	CHD	94	188	0.67 (0.47, 0.95)	per SD increase	1.00	0.67 (0.47, 0.95)	+
**DHA (22:6n3)**											
Tikkanen 2021	FINRISK & Health	Finland	serum	CAD	2073	31657	0.90 (0.87, 0.94)	per SD increase	1.00	0.90 (0.87, 0.94)	+
Papandreou 2019	PREDIMED	Spain	RBC	CHD	136	408	1.05 (0.84, 1.32)	per SD increase	1.00	1.05 (0.84, 1.32)	++
Chei 2018	CIRCS	Japan	serum	CAD	152	608	0.87 (0.70, 1.09)	per SD increase	1.00	0.87 (0.70, 1.09)	++
Holmes 2018	CKB	China	plasma	MI	912	2378	0.84 (0.76, 0.92)	per SD increase	1.00	0.84 (0.76, 0.92)	+++
Satizabal 2018	FHS	US	plasma PL	CHD	130	772	0.97 (0.82, 1.16)	per SD increase	1.00	0.97 (0.82, 1.16)	++
Harris 2018	FHS-Offspring	US	RBC	CHD	119	2500	0.54 (0.27, 1.07)	Highest versus lowest fifth	0.36	0.80 (0.63, 1.03)	+++

Hamazaki 2017	JPHC	Japan	plasma PL	CHD	209	627	0.71 (0.37, 1.36)	Highest versus lowest fourth	0.39	0.87 (0.68, 1.13)	++
Del Gobbo 2016	3C Study	France	plasma	CHD	57	1416	1.31 (0.93, 1.86)	per SD increase	1.00	1.31 (0.93, 1.86)	++++*
Del Gobbo 2016	ARIC	US	plasma PL	CHD	398	3793	0.98 (0.89, 1.09)	per SD increase	1.00	0.98 (0.89, 1.09)	++++*
Del Gobbo 2016	CHS	US	plasma PL	CHD	1163	3941	0.91 (0.85, 0.99)	per SD increase	1.00	0.91 (0.85, 0.99)	++++*
Del Gobbo 2016	HPFS	US	plasma	CHD	466	1291	0.85 (0.71, 1.03)	per SD increase	1.00	0.85 (0.71, 1.03)	++++*
Del Gobbo 2016	InCHIANTI	Italy	plasma	CHD	115	839	0.99 (0.79, 1.23)	per SD increase	1.00	0.99 (0.79, 1.23)	++++*
Del Gobbo 2016	KIHD	Finland	serum	CHD	452	1837	0.96 (0.87, 1.06)	per SD increase	1.00	0.96 (0.87, 1.06)	++++*
Del Gobbo 2016	MCCS	Australia	plasma PL	fatal CHD	202	5279	0.98 (0.85, 1.13)	per SD increase	1.00	0.98 (0.85, 1.13)	++++*
Del Gobbo 2016	NHS I	US	plasma	CHD	437	1040	0.83 (0.63, 1.08)	per SD increase	1.00	0.83 (0.63, 1.08)	++++*
Del Gobbo 2016	NSHDS I	Sweden	plasma PL	CHD	64	183	0.73 (0.44, 1.21)	per SD increase	1.00	0.73 (0.44, 1.21)	++++*
Del Gobbo 2016	NSHDS II	Sweden	plasma PL	CHD	353	759	0.81 (0.66, 0.99)	per SD increase	1.00	0.81 (0.66, 0.99)	++++*
Del Gobbo 2016	SCHS	Singapore	plasma	CHD	759	1555	0.80 (0.70, 0.92)	per SD increase	1.00	0.80 (0.70, 0.92)	++++*
Del Gobbo 2016	SHHEC	Scotland	AT	CHD	898	4391	1.01 (0.94, 1.09)	per SD increase	1.00	1.01 (0.94, 1.09)	++++*
Del Gobbo 2016	ULSAM 50	Sweden	serum CE	CHD	749	2001	1.01 (0.93, 1.09)	per SD increase	1.00	1.01 (0.93, 1.09)	++++*
Del Gobbo 2016	ULSAM 70	Sweden	AT	CHD	198	728	1.13 (0.98, 1.29)	per SD increase	1.00	1.13 (0.98, 1.29)	++++*
Matthan 2014	WHI-OS	US	plasma PL	CHD	1224	2448	0.56 (0.39, 0.80)	per unit increase (SD=1.05)	1.05	0.54 (0.37, 0.79)	++
Otto 2013	MESA	US	plasma PL	CHD	141	2837	0.29 (0.15, 0.58)	Highest versus lowest fourth	0.39	0.61 (0.47, 0.80)	++
De Goede 2013	MP–CVDRF	Netherlands	plasma CE	fatal CHD	222	444	1.07 (0.88, 1.30)	per SD increase	1.00	1.07 (0.88, 1.30)	++
Matsumoto 2013	PHS	US	RBC	CHD	1000	2000	0.99 (0.90, 1.10)	per SD increase	1.00	0.99 (0.90, 1.10)	++
Simon 1995	MRFIT	US	serum PL	CHD	94	188	0.66 (0.46, 0.94)	per SD increase	1.00	0.66 (0.46, 0.94)	+
**Omega-3 index (EPA+DHA)**											
Papandreou 2019	PREDIMED	Spain	RBC	CHD	136	408	1.09 (0.87, 1.36)	per SD increase	1.00	1.09 (0.87, 1.36)	++
Harris 2018	FHS-Offspring	US	RBC	CHD	119	2500	0.58 (0.29, 1.18)	Highest versus lowest fifth	0.36	0.82 (0.64, 1.06)	+++
Hamazaki 2017	JPHC	Japan	plasma PL	CHD	209	627	0.77 (0.40, 1.50)	Highest versus lowest fourth	0.39	0.90 (0.70, 1.17)	++
Sun 2016	SCHS	Singapore	plasma	AMI	744	1488	0.62 (0.41, 0.94)	Highest versus lowest fourth	0.39	0.83 (0.70, 0.98)	++++*
De Goede 2013	MP–CVDRF	Netherlands	plasma CE	fatal CHD	222	444	1.06 (0.88, 1.29)	per SD increase	1.00	1.06 (0.88, 1.29)	++
Lemaitre 2003	CHS	US	plasma PL	fatal CHD	54	108	0.30 (0.12, 0.76)	per SD increase	1.00	0.30 (0.12, 0.76)	++
Lemaitre 2003	CHS	US	plasma PL	non-fatal MI	125	250	0.97 (0.71, 1.33)	per SD increase	1.00	0.97 (0.71, 1.33)	++
Hallgren 2001	VIP+MONICA	Sweden	plasma PL	CHD	78	234	0.43 (0.24, 0.79)	Cutoff: 5.5% (mean=6.3%, SD=1.6%)	0.60	0.60 (0.42, 0.86)	+
Guallar 1995	PHS	US	plasma PL	MI	213	426	1.55 (0.67, 3.54)	Highest versus lowest fifth	0.36	1.17 (0.87, 1.57)	+++
**Total N6 PUFA**											
Tikkanen 2021	FINRISK & Health	Finland	serum	CAD	2073	31657	0.81 (0.78, 0.84)	per SD increase	1.00	0.81 (0.78, 0.84)	+
Papandreou 2019	PREDIMED	Spain	RBC	CHD	136	408	0.94 (0.74, 1.20)	per SD increase	1.00	0.94 (0.74, 1.20)	++
Chei 2018	CIRCS	Japan	serum	CAD	152	608	0.66 (0.52, 0.84)	per SD increase	1.00	0.66 (0.52, 0.84)	++
Holmes 2018	CKB	China	plasma	MI	912	2378	0.92 (0.84, 1.02)	per SD increase	1.00	0.92 (0.84, 1.02)	+++
Harris 2018	FHS-Offspring	US	RBC	CHD	119	2500	1.69 (0.87, 3.26)	Highest versus lowest fifth	0.36	1.21 (0.95, 1.53)	+++
Matthan 2014	WHI-OS	US	plasma PL	CHD	1224	2448	1.02 (0.97, 1.07)	per unit increase (SD=2.45)	2.45	1.05 (0.93, 1.18)	++
Woodward 2011	SHHEC	Scotland	AT	CHD	651	3944	0.85 (0.68, 1.08)	Highest versus lowest fourth	0.39	0.94 (0.86, 1.03)	+++
**LA (18:2n6)**											
Tikkanen 2021	FINRISK & Health	Finland	serum	CAD	2073	31657	0.86 (0.82, 0.89)	per SD increase	1.00	0.86 (0.82, 0.89)	+
Marklund 2019	3C Study	France	plasma	CHD	57	1416	1.35 (0.66, 2.78)	per interquintile range (10-90th)	0.39	1.12 (0.85, 1.49)	++++*
Marklund 2019	60YO	Sweden	serum CE	CHD	199	4150	0.77 (0.52, 1.12)	per interquintile range (10-90th)	0.39	0.90 (0.78, 1.05)	++++*
Marklund 2019	AGES-Reykjavik	Iceland	plasma PL	CHD	286	1195	1.16 (0.87, 1.55)	per interquintile range (10-90th)	0.39	1.06 (0.95, 1.19)	++++*
Marklund 2019	ARIC	US	plasma PL	CHD	398	3749	1.16 (0.87, 1.56)	per interquintile range (10-90th)	0.39	1.06 (0.95, 1.19)	++++*
Marklund 2019	CCCC	Taiwan	plasma	CHD	196	1838	0.19 (0.09, 0.39)	per interquintile range (10-90th)	0.39	0.52 (0.39, 0.70)	++++*
Marklund 2019	CHS	US	plasma PL	CHD	875	2907	1.07 (0.85, 1.34)	per interquintile range (10-90th)	0.39	1.03 (0.94, 1.12)	++++*
Marklund 2019	HPFS-total plasma	US	plasma	CHD	431	1510	0.76 (0.54, 1.08)	per interquintile range (10-90th)	0.39	0.90 (0.78, 1.03)	++++*
Marklund 2019	HS	Japan	serum	CHD	78	3103	0.82 (0.41, 1.67)	per interquintile range (10-90th)	0.39	0.93 (0.70, 1.22)	++++*
Marklund 2019	KIHD	Finland	serum	CHD	472	1837	1.00 (0.77, 1.30)	per interquintile range (10-90th)	0.39	1.00 (0.90, 1.11)	++++*
Marklund 2019	MCCS	Australia	plasma PL	fatal CHD	238	6265	0.94 (0.59, 1.49)	per interquintile range (10-90th)	0.39	0.98 (0.81, 1.17)	++++*
Marklund 2019	MESA	US	plasma PL	CHD	143	2722	1.40 (0.73, 2.68)	per interquintile range (10-90th)	0.39	1.14 (0.88, 1.47)	++++*

Marklund 2019	NHS (total plasma)	US	plasma	CHD	437	1970	0.96 (0.63, 1.47)	per interquintile range (10-90th)	0.39	0.98 (0.83, 1.16)	++++*
Marklund 2019	NSHDS I	Sweden	plasma PL	CHD	64	183	2.45 (0.47, 12.69)	per interquintile range (10-90th)	0.39	1.42 (0.75, 2.70)	++++*
Marklund 2019	NSHDS II	Sweden	plasma PL	CHD	353	759	0.28 (0.14, 0.53)	per interquintile range (10-90th)	0.39	0.61 (0.47, 0.79)	++++*
Marklund 2019	PHS	US	RBC	CHD	1000	2000	1.02 (0.88, 1.19)	per interquintile range (10-90th)	0.39	1.01 (0.95, 1.07)	++++*
Marklund 2019	PIVUS	Sweden	serum PL	CHD	57	835	1.10 (0.48, 2.52)	per interquintile range (10-90th)	0.39	1.04 (0.75, 1.43)	++++*
Papandreou 2019	PREDIMED	Spain	RBC	CHD	136	408	1.08 (0.86, 1.35)	per SD increase	1.00	1.08 (0.86, 1.35)	++
Marklund 2019	SCHS	Singapore	plasma	CHD	759	1555	0.89 (0.66, 1.20)	per interquintile range (10-90th)	0.39	0.96 (0.85, 1.07)	++++*
Marklund 2019	SHHEC	UK	AT	CHD	936	4391	0.91 (0.76, 1.08)	per interquintile range (10-90th)	0.39	0.96 (0.90, 1.03)	++++*
Marklund 2019	ULSAM 50	Sweden	serum CE	CHD	643	1992	0.78 (0.62, 0.99)	per interquintile range (10-90th)	0.39	0.91 (0.83, 0.99)	++++*
Marklund 2019	ULSAM 70	Sweden	AT	CHD	181	763	0.69 (0.44, 1.09)	per interquintile range (10-90th)	0.39	0.87 (0.72, 1.03)	++++*
Marklund 2019	WHI-MS	US	RBC	CHD	484	5263	0.84 (0.62, 1.13)	per interquintile range (10-90th)	0.39	0.93 (0.83, 1.05)	++++*
Chei 2018	CIRCS	Japan	serum	CAD	152	608	0.67 (0.53, 0.85)	per SD increase	1.00	0.67 (0.53, 0.85)	++
Holmes 2018	CKB	China	plasma	MI	912	2378	0.97 (0.88, 1.07)	per SD increase	1.00	0.97 (0.88, 1.07)	+++
Satizabal 2018	FHS	US	plasma PL	CHD	130	772	1.02 (0.86, 1.21)	per SD increase	1.00	1.02 (0.86, 1.21)	++
Harris 2018	FHS-Offspring	US	RBC	CHD	119	2500	1.56 (0.75, 3.25)	Highest versus lowest fifth	0.36	1.17 (0.90, 1.52)	+++
Matthan 2014	WHI-OS	US	plasma PL	CHD	1224	2448	0.94 (0.43, 2.07)	per unit increase (SD=2.8)	2.80	0.84 (0.09, 7.66)	++
De Goede 2013	MP–CVDRF	Netherlands	plasma CE	fatal CHD	222	444	0.90 (0.74, 1.10)	per SD increase	1.00	0.90 (0.74, 1.10)	++
Simon 1995	MRFIT	US	serum PL	CHD	94	188	0.83 (0.61, 1.14)	per SD increase	1.00	0.83 (0.61, 1.14)	+
**GLA (18:3n6)**											
Chei 2018	CIRCS	Japan	serum	CAD	152	608	1.09 (0.88, 1.34)	per SD increase	1.00	1.09 (0.88, 1.34)	++
Harris 2018	FHS-Offspring	US	RBC	CHD	119	2500	0.92 (0.49, 1.71)	Highest versus lowest fifth	0.36	0.97 (0.78, 1.21)	+++
Virtanen 2018	KIHD	Finland	serum	fatal CHD	145	1461	1.15 (0.98, 1.34)	per SD increase	1.00	1.15 (0.98, 1.34)	+
Wu 2014	CHS	US	plasma PL	CHD	720	2792	1.16 (0.91, 1.47)	Highest versus lowest fifth	0.36	1.05 (0.97, 1.15)	++++*
Matthan 2014	WHI-OS	US	plasma PL	CHD	1224	2448	1.24 (0.98, 1.56)	per unit increase (SD=0.03)	0.03	1.01 (1.00, 1.02)	++
Woodward 2011	SHHEC	Scotland	AT	CHD	651	3944	0.86 (0.68, 1.09)	Highest versus lowest fourth	0.39	0.94 (0.86, 1.03)	+++
**EDA (20:2n6)**											
Papandreou 2019	PREDIMED	Spain	RBC	CHD	136	408	1.04 (0.85, 1.29)	per SD increase	1.00	1.04 (0.85, 1.29)	++
Harris 2018	FHS-Offspring	US	RBC	CHD	119	2500	1.64 (0.92, 2.93)	Highest versus lowest fifth	0.36	1.19 (0.97, 1.47)	+++
Matthan 2014	WHI-OS	US	plasma PL	CHD	1224	2448	0.96 (0.69, 1.32)	per unit increase (SD=0.35)	0.35	0.99 (0.88, 1.10)	++
Simon 1995	MRFIT	US	serum PL	CHD	94	188	1.01 (0.75, 1.37)	per SD increase	1.00	1.01 (0.75, 1.37)	+
**DGLA (20:3n6)**											
Papandreou 2019	PREDIMED	Spain	RBC	CHD	136	408	1.22 (0.97, 1.55)	per SD increase	1.00	1.22 (0.97, 1.55)	++
Chei 2018	CIRCS	Japan	serum	CAD	152	608	1.19 (0.96, 1.49)	per SD increase	1.00	1.19 (0.96, 1.49)	++
Harris 2018	FHS-Offspring	US	RBC	CHD	119	2500	1.29 (0.69, 2.42)	Highest versus lowest fifth	0.36	1.10 (0.88, 1.37)	+++
Virtanen 2018	KIHD	Finland	serum	fatal CHD	145	1461	1.09 (0.93, 1.28)	per SD increase	1.00	1.09 (0.93, 1.28)	+
Wu 2014	CHS	US	plasma PL	CHD	720	2792	1.07 (0.84, 1.37)	Highest versus lowest fifth	0.36	1.02 (0.94, 1.12)	++++*
Matthan 2014	WHI-OS	US	plasma PL	CHD	1224	2448	3.22 (1.95, 5.32)	per unit increase (SD=0.7)	0.70	2.27 (1.60, 3.22)	++
Woodward 2011	SHHEC	Scotland	AT	CHD	651	3944	0.89 (0.69, 1.13)	Highest versus lowest fourth	0.39	0.96 (0.87, 1.05)	+++
Simon 1995	MRFIT	US	serum PL	CHD	94	188	1.40 (1.01, 1.95)	per SD increase	1.00	1.40 (1.01, 1.95)	+
**AA (20:4n6)**											
Marklund 2019	3C Study	France	plasma	CHD	57	1416	1.13 (0.62, 2.08)	per interquintile range (10-90th)	0.39	1.05 (0.83, 1.33)	++++*
Marklund 2019	60YO	Sweden	serum CE	CHD	199	4150	1.19 (0.77, 1.82)	per interquintile range (10-90th)	0.39	1.07 (0.90, 1.27)	++++*
Marklund 2019	AGES-Reykjavik	Iceland	plasma PL	CHD	286	1195	0.82 (0.58, 1.15)	per interquintile range (10-90th)	0.39	0.93 (0.81, 1.06)	++++*
Marklund 2019	ARIC	US	plasma PL	CHD	398	3749	1.05 (0.81, 1.35)	per interquintile range (10-90th)	0.39	1.02 (0.92, 1.13)	++++*
Marklund 2019	CCCC	Taiwan	plasma	CHD	196	1838	0.51 (0.31, 0.87)	per interquintile range (10-90th)	0.39	0.77 (0.63, 0.94)	++++*
Marklund 2019	CHS	US	plasma PL	CHD	875	2907	1.08 (0.87, 1.33)	per interquintile range (10-90th)	0.39	1.03 (0.95, 1.12)	++++*
Marklund 2019	HPFS-total plasma	US	plasma	CHD	431	1510	0.84 (0.57, 1.24)	per interquintile range (10-90th)	0.39	0.93 (0.80, 1.09)	++++*
Marklund 2019	HS	Japan	serum	CHD	78	3103	1.19 (0.68, 2.10)	per interquintile range (10-90th)	0.39	1.07 (0.86, 1.33)	++++*
Marklund 2019	KIHD	Finland	serum	CHD	472	1837	0.78 (0.58, 1.06)	per interquintile range (10-90th)	0.39	0.91 (0.81, 1.02)	++++*
Marklund 2019	MCCS	Australia	plasma PL	fatal CHD	238	6265	0.71 (0.49, 1.03)	per interquintile range (10-90th)	0.39	0.87 (0.76, 1.01)	++++*
Marklund 2019	MESA	US	plasma PL	CHD	143	2722	1.02 (0.55, 1.89)	per interquintile range (10-90th)	0.39	1.01 (0.79, 1.28)	++++*

Marklund 2019	NHS (total plasma)	US	plasma	CHD	437	1970	0.48 (0.30, 0.79)	per interquintile range (10-90th)	0.39	0.75 (0.62, 0.91)	++++*
Marklund 2019	NSHDS I	Sweden	plasma PL	CHD	64	183	0.19 (0.05, 0.77)	per interquintile range (10-90th)	0.39	0.52 (0.31, 0.89)	++++*
Marklund 2019	NSHDS II	Sweden	plasma PL	CHD	353	759	2.50 (1.41, 4.41)	per interquintile range (10-90th)	0.39	1.43 (1.14, 1.79)	++++*
Marklund 2019	PHS	US	RBC	CHD	1000	2000	0.98 (0.90, 1.07)	per interquintile range (10-90th)	0.39	0.99 (0.96, 1.03)	++++*
Marklund 2019	PIVUS	Sweden	serum PL	CHD	57	835	0.87 (0.43, 1.76)	per interquintile range (10-90th)	0.39	0.95 (0.72, 1.25)	++++*
Papandreou 2019	PREDIMED	Spain	RBC	CHD	136	408	0.94 (0.75, 1.17)	per SD increase	1.00	0.94 (0.75, 1.17)	++
Marklund 2019	SCHS	Singapore	plasma	CHD	759	1555	1.33 (0.95, 1.86)	per interquintile range (10-90th)	0.39	1.12 (0.98, 1.27)	++++*
Marklund 2019	SHHEC	UK	AT	CHD	936	4391	0.98 (0.86, 1.13)	per interquintile range (10-90th)	0.39	0.99 (0.94, 1.05)	++++*
Marklund 2019	ULSAM 50	Sweden	serum CE	CHD	643	1992	0.98 (0.78, 1.24)	per interquintile range (10-90th)	0.39	0.99 (0.91, 1.09)	++++*
Marklund 2019	ULSAM 70	Sweden	AT	CHD	181	763	1.05 (0.71, 1.57)	per interquintile range (10-90th)	0.39	1.02 (0.87, 1.19)	++++*
Marklund 2019	WHI-MS	US	RBC	CHD	484	5263	0.90 (0.64, 1.28)	per interquintile range (10-90th)	0.39	0.96 (0.84, 1.10)	++++*
Chei 2018	CIRCS	Japan	serum	CAD	152	608	0.93 (0.75, 1.14)	per SD increase	1.00	0.93 (0.75, 1.14)	++
Satizabal 2018	FHS	US	plasma PL	CHD	130	772	0.97 (0.82, 1.16)	per SD increase	1.00	0.97 (0.82, 1.16)	++
Harris 2018	FHS-Offspring	US	RBC	CHD	119	2500	0.93 (0.48, 1.77)	Highest versus lowest fifth	0.36	0.97 (0.77, 1.23)	+++
Matthan 2014	WHI-OS	US	plasma PL	CHD	1224	2448	0.68 (0.38, 1.23)	per unit increase (SD=2.1)	2.10	0.45 (0.13, 1.54)	++
De Goede 2013	MP–CVDRF	Netherlands	plasma CE	fatal CHD	222	444	1.02 (0.83, 1.25)	per SD increase	1.00	1.02 (0.83, 1.25)	++
Simon 1995	MRFIT	US	serum PL	CHD	94	188	0.99 (0.73, 1.35)	per SD increase	1.00	0.99 (0.73, 1.35)	+
**DTA (22:4n6)**											
Papandreou 2019	PREDIMED	Spain	RBC	CHD	136	408	0.73 (0.58, 0.93)	per SD increase	1.00	0.73 (0.58, 0.93)	++
Harris 2018	FHS-Offspring	US	RBC	CHD	119	2500	1.81 (0.89, 3.69)	Highest versus lowest fifth	0.36	1.24 (0.96, 1.59)	+++
Matthan 2014	WHI-OS	US	plasma PL	CHD	1224	2448	1.39 (0.94, 2.06)	per unit increase (SD=0.1)	0.10	1.04 (0.99, 1.08)	++
Simon 1995	MRFIT	US	serum PL	CHD	94	188	1.17 (0.87, 1.59)	per SD increase	1.00	1.17 (0.87, 1.59)	+
**DPA (22:5n6)**											
Papandreou 2019	PREDIMED	Spain	RBC	CHD	136	408	0.66 (0.51, 0.86)	per SD increase	1.00	0.66 (0.51, 0.86)	++
Harris 2018	FHS-Offspring	US	RBC	CHD	119	2500	1.41 (0.73, 2.70)	Highest versus lowest fifth	0.36	1.13 (0.90, 1.43)	+++
Matthan 2014	WHI-OS	US	plasma PL	CHD	1224	2448	1.63 (1.20, 2.23)	per unit increase (SD=0.14)	0.14	1.07 (1.03, 1.12)	++
Simon 1995	MRFIT	US	serum PL	CHD	94	188	1.20 (0.87, 1.67)	per SD increase	1.00	1.20 (0.87, 1.67)	+
**Total trans**											
Matthan 2014	WHI-OS	US	plasma PL	CHD	1224	2448	1.00 (0.81, 1.24)	per unit increase (SD=0.35)	0.35	1.00 (0.93, 1.08)	++
Sun 2007	NHS	US	RBC	CHD	166	493	2.70 (1.30, 5.60)	Highest versus lowest fourth	0.39	1.48 (1.11, 1.97)	++
Lemaitre 2006	CHS	US	plasma PL	fatal CHD	214	428	0.90 (0.65, 1.24)	per interquintile range (10-90th)	0.39	0.96 (0.85, 1.09)	++
**trans-16:1**											
Papandreou 2019	PREDIMED	Spain	RBC	CHD	136	408	1.15 (0.93, 1.42)	per SD increase	1.00	1.15 (0.93, 1.42)	++
Otto 2018	CHS	US	plasma PL	CHD	876	2907	0.95 (0.77, 1.19)	Highest versus lowest fifth	0.36	0.98 (0.91, 1.06)	+
Otto 2013	MESA	US	plasma PL	CHD	146	2837	0.91 (0.75, 1.10)	per SD increase	1.00	0.91 (0.75, 1.10)	++
Sun 2007	NHS	US	plasma	CHD	166	493	0.61 (0.39, 0.97)	Highest versus lowest third	0.46	0.80 (0.65, 0.98)	++
**trans-18:1**											
Papandreou 2019	PREDIMED	Spain	RBC	CHD	136	408	1.16 (0.91, 1.48)	per SD increase	1.00	1.16 (0.91, 1.48)	++
Sun 2016	SCHS	Singapore	plasma	AMI	744	1488	0.88 (0.78, 1.01)	per SD increase	1.00	0.88 (0.78, 1.01)	++
Matthan 2014	WHI-OS	US	plasma PL	CHD	1224	2448	0.99 (0.82, 1.20)	per unit increase (SD=0.35)	0.35	1.00 (0.93, 1.07)	++
Sun 2007	NHS	US	RBC	CHD	166	493	2.50 (1.20, 5.00)	Highest versus lowest fourth	0.39	1.43 (1.08, 1.90)	++
Lemaitre 2006	CHS	US	plasma PL	fatal CHD	214	428	0.38 (0.17, 0.86)	Highest versus lowest fifth	0.36	0.71 (0.53, 0.95)	++++*
**trans-18:2**											
Matthan 2014	WHI-OS	US	plasma PL	CHD	1224	2448	1.05 (0.81, 1.37)	per unit increase (SD=0.07)	0.07	1.00 (0.99, 1.02)	++
Sun 2007	NHS	US	RBC	CHD	166	493	2.20 (1.00, 4.80)	Highest versus lowest fourth	0.39	1.36 (1.00, 1.86)	++
Lemaitre 2006	CHS	US	plasma PL	fatal CHD	214	428	1.20 (0.94, 1.53)	per interquintile range (10-90th)	0.39	1.07 (0.98, 1.18)	++

RR: Relative risk; SD: Standard deviation. See Supplementary eTable 17. Footnote for other abbreviations.

Adjustment: +, adjusted for sex, age, and/or other demographic information; ++, adjusted for preceding plus any non-lipid conventional risk factors; +++, adjusted for preceding plus lipids; ++++, adjusted for preceding plus other circulating fatty acids (* not included lipids).

^Malik 2012 was published as an abstract but the estimates were reported in the meta-analysis paper of Trieu 2021, which were also extracted in our updated meta-analysis.

Supplementary eTable 19. Further characteristics of non-duplicated associations of fatty acid biomarkers with stroke included in the updated meta-analyses

Paper	Data source	Region	Biomarker	Outcome	N of events	N	reported RR (95%CI)	Scale of RR reported by study	Conversion factor	RR (95%CI) per 1-SD	Adjustment
**Total SFA**											
Holmes 2018	CKB	China	plasma	IS	1146	2612	0.84 (0.77, 0.93)	per SD increase	1.00	0.84 (0.77, 0.93)	+++
Yamagishi 2013	ARIC	US	plasma PL	IS	168	3870	1.64 (1.05, 2.57)	Highest versus lowest fourth	0.39	1.22 (1.02, 1.45)	+
**Even-chain SFA**											
Woodward 2011	SHHEC	Scotland	AT	stroke	219	3944	0.78 (0.53, 1.16)	Highest versus lowest fourth	0.39	0.91 (0.78, 1.06)	+++
Iso 2002	JapanCRS	Japan	serum	Stroke	197	788	1.31 (1.05, 1.65)	per SD increase	1.00	1.31 (1.05, 1.65)	+++
**Myristic acid (14:0)**											
Yakoob 2014	HPFS	US	plasma	stroke	122	244	1.18 (0.90, 1.53)	per SD increase	1.00	1.18 (0.90, 1.53)	++
Yakoob 2014	NHS	US	plasma	stroke	472	944	1.16 (1.00, 1.33)	per SD increase	1.00	1.16 (1.00, 1.33)	++
Yamagishi 2013	ARIC	US	plasma PL	IS	168	3870	1.29 (0.83, 2.01)	Highest versus lowest fourth	0.39	1.11 (0.93, 1.32)	+
Yaemsiri 2013	WHI-OS	US	serum	IS	964	1928	1.16 (0.97, 1.38)	per SD increase	1.00	1.16 (0.97, 1.38)	++
Woodward 2011	SHHEC	Scotland	AT	stroke	219	3944	0.82 (0.56, 1.21)	Highest versus lowest fourth	0.39	0.92 (0.79, 1.08)	+++
Wiberg 2006	ULSAM 50	Sweden	serum CE	Stroke or TIA	421	2313	1.07 (0.97, 1.18)	per SD increase	1.00	1.07 (0.97, 1.18)	++
Iso 2002	JapanCRS	Japan	serum	Stroke	197	788	1.34 (1.14, 1.58)	per SD increase	1.00	1.34 (1.14, 1.58)	+
Simon 1995	MRFIT	US	serum PL	stroke	96	192	0.96 (0.70, 1.31)	per SD increase	1.00	0.96 (0.70, 1.31)	+
**Palmitic acid (16:0)**											
Satizabal 2018	3C Study	France	plasma	Stroke	51	1313	1.24 (0.97, 1.58)	per SD increase	1.00	1.24 (0.97, 1.58)	++
Satizabal 2018	FHS	US	plasma PL	Stroke	81	906	1.22 (0.98, 1.52)	per SD increase	1.00	1.22 (0.98, 1.52)	++
Yamagishi 2013	ARIC	US	plasma PL	IS	168	3870	1.30 (0.84, 2.02)	Highest versus lowest fourth	0.39	1.11 (0.93, 1.32)	+
Yaemsiri 2013	WHI-OS	US	serum	IS	964	1928	1.23 (1.02, 1.47)	per SD increase	1.00	1.23 (1.02, 1.47)	++
Woodward 2011	SHHEC	Scotland	AT	stroke	219	3944	0.65 (0.43, 0.97)	Highest versus lowest fourth	0.39	0.84 (0.72, 0.99)	+++
Wiberg 2006	ULSAM 50	Sweden	serum CE	Stroke or TIA	421	2313	1.11 (1.00, 1.23)	per SD increase	1.00	1.11 (1.00, 1.23)	++
Iso 2002	JapanCRS	Japan	serum	Stroke	197	788	1.35 (1.14, 1.59)	per SD increase	1.00	1.35 (1.14, 1.59)	+
Simon 1995	MRFIT	US	serum PL	stroke	96	192	0.80 (0.59, 1.09)	per SD increase	1.00	0.80 (0.59, 1.09)	+
**Stearic acid (18:0)**											
Yamagishi 2013	ARIC	US	plasma PL	IS	168	3870	1.17 (0.74, 1.84)	Highest versus lowest fourth	0.39	1.06 (0.89, 1.27)	+
Yaemsiri 2013	WHI-OS	US	serum	IS	964	1928	0.93 (0.77, 1.12)	per SD increase	1.00	0.93 (0.77, 1.12)	++
Woodward 2011	SHHEC	Scotland	AT	stroke	219	3944	0.83 (0.56, 1.22)	Highest versus lowest fourth	0.39	0.93 (0.80, 1.08)	+++
Wiberg 2006	ULSAM 50	Sweden	serum CE	Stroke or TIA	421	2313	0.98 (0.90, 1.11)	per SD increase	1.00	0.98 (0.90, 1.11)	++
Simon 1995	MRFIT	US	serum PL	stroke	96	192	1.37 (1.01, 1.87)	per SD increase	1.00	1.37 (1.01, 1.87)	+
**Odd-chain SFA**											
Warensjö 2009	VIP+MONICA	Sweden	plasma PL	stroke	108	324	0.70 (0.53, 0.92)	per SD increase	1.00	0.70 (0.53, 0.92)	+
**Pentadecanoic acid (15:0)**											
Trieu 2021	60YO	Sweden	serum CE	IS	192	4150	0.94 (0.81, 1.10)	per SD increase	1.00	0.94 (0.81, 1.10)	++
Otto 2018	CHS	US	plasma PL	stroke	529	2907	0.93 (0.70, 1.25)	Highest versus lowest fifth	0.36	0.97 (0.88, 1.08)	+
Yakoob 2014	HPFS	US	plasma	stroke	122	244	1.02 (0.81, 1.28)	per SD increase	1.00	1.02 (0.81, 1.28)	++
Yakoob 2014	NHS	US	plasma	stroke	472	944	1.01 (0.88, 1.17)	per SD increase	1.00	1.01 (0.88, 1.17)	++
Yamagishi 2013	ARIC	US	plasma PL	IS	168	3870	0.89 (0.60, 1.33)	Highest versus lowest fourth	0.39	0.96 (0.82, 1.12)	+
Yaemsiri 2013	WHI-OS	US	serum	IS	964	1928	1.11 (0.90, 1.35)	per SD increase	1.00	1.11 (0.90, 1.35)	++
Warensjö 2009	VIP+MONICA	Sweden	plasma PL	stroke	108	324	0.81 (0.62, 1.10)	per SD increase	1.00	0.81 (0.62, 1.10)	+
**Heptadecanoic acid (17:0)**											
Otto 2018	CHS	US	plasma PL	stroke	529	2907	0.86 (0.65, 1.14)	Highest versus lowest fifth	0.36	0.95 (0.86, 1.05)	+
Yakoob 2014	HPFS	US	plasma	stroke	122	244	0.81 (0.62, 1.06)	per SD increase	1.00	0.81 (0.62, 1.06)	++
Yakoob 2014	NHS	US	plasma	stroke	472	944	0.96 (0.84, 1.09)	per SD increase	1.00	0.96 (0.84, 1.09)	++
Yaemsiri 2013	WHI-OS	US	serum	IS	964	1928	1.00 (0.84, 1.19)	per SD increase	1.00	1.00 (0.84, 1.19)	++
Warensjö 2009	VIP+MONICA	Sweden	plasma PL	stroke	108	324	0.67 (0.51, 0.89)	per SD increase	1.00	0.67 (0.51, 0.89)	+

**Total MUFA**										
Holmes 2018	CKB	China	plasma	IS	1146	2612	1.06 (0.96, 1.16)	per SD increase	1.00	1.06 (0.96, 1.16)	+++
Yamagishi 2013	ARIC	US	plasma PL	IS	168	3870	1.42 (0.92, 2.18)	Highest versus lowest fourth	0.39	1.15 (0.97, 1.36)	+
Woodward 2011	SHHEC	Scotland	AT	stroke	219	3944	1.72 (1.14, 2.58)	Highest versus lowest fourth	0.39	1.24 (1.05, 1.45)	+++
**Palmitoleic acid (16:1n7)**											
Yamagishi 2013	ARIC	US	plasma PL	IS	168	3870	1.52 (0.99, 2.34)	Highest versus lowest fourth	0.39	1.18 (1.00, 1.40)	+
Yaemsiri 2013	WHI-OS	US	serum	IS	964	1928	1.13 (0.95, 1.34)	per SD increase	1.00	1.13 (0.95, 1.34)	++
Woodward 2011	SHHEC	Scotland	AT	stroke	219	3944	1.17 (0.80, 1.72)	Highest versus lowest fourth	0.39	1.06 (0.91, 1.24)	+++
Wiberg 2006	ULSAM 50	Sweden	serum CE	Stroke or TIA	421	2313	1.17 (1.06, 1.30)	per SD increase	1.00	1.17 (1.06, 1.30)	++
Iso 2002	JapanCRS	Japan	serum	Stroke	197	788	1.39 (1.17, 1.65)	per SD increase	1.00	1.39 (1.17, 1.65)	+
Simon 1995	MRFIT	US	serum PL	stroke	96	192	1.14 (0.86, 1.52)	per SD increase	1.00	1.14 (0.86, 1.52)	+
**Oleic acid (18:1n9)**											
Yamagishi 2013	ARIC	US	plasma PL	IS	168	3870	1.38 (0.88, 2.15)	Highest versus lowest fourth	0.39	1.14 (0.95, 1.35)	+
Yaemsiri 2013	WHI-OS	US	serum	IS	964	1928	1.18 (0.99, 1.41)	per SD increase	1.00	1.18 (0.99, 1.41)	++
Woodward 2011	SHHEC	Scotland	AT	stroke	219	3944	1.48 (0.99, 2.20)	Highest versus lowest fourth	0.39	1.17 (1.00, 1.37)	+++
Wiberg 2006	ULSAM 50	Sweden	serum CE	Stroke or TIA	421	2313	1.22 (1.10, 1.35)	per SD increase	1.00	1.22 (1.10, 1.35)	++
Iso 2002	JapanCRS	Japan	serum	Stroke	197	788	1.32 (1.11, 1.56)	per SD increase	1.00	1.32 (1.11, 1.56)	+
Simon 1995	MRFIT	US	serum PL	stroke	96	192	1.27 (0.91, 1.77)	per SD increase	1.00	1.27 (0.91, 1.77)	+
**Eicosenoic acid (20:1n9)**											
Yaemsiri 2013	WHI-OS	US	serum	IS	964	1928	1.12 (0.81, 1.55)	per SD increase	1.00	1.12 (0.81, 1.55)	++
Woodward 2011	SHHEC	Scotland	AT	stroke	219	3944	1.52 (1.06, 2.20)	Highest versus lowest fourth	0.39	1.18 (1.02, 1.36)	+++
**Nervonic acid (24:1n9)**											
Yaemsiri 2013	WHI-OS	US	serum	IS	964	1928	0.88 (0.66, 1.17)	per SD increase	1.00	0.88 (0.66, 1.17)	++
**Total PUFA**											
Holmes 2018	CKB	China	plasma	IS	1146	2612	1.05 (0.96, 1.15)	per SD increase	1.00	1.05 (0.96, 1.15)	+++
Woodward 2011	SHHEC	Scotland	AT	stroke	219	3944	0.72 (0.48, 1.10)	Highest versus lowest fourth	0.39	0.88 (0.75, 1.03)	+++
**Total N3 PUFA**											
Holmes 2018	CKB	China	plasma	IS	1146	2612	1.02 (0.92, 1.12)	per SD increase	1.00	1.02 (0.92, 1.12)	+++
Daneshmand 2016	KIHD	Finland	serum	Stroke	202	1828	1.06 (0.72, 1.55)	Highest versus lowest fourth	0.39	1.02 (0.88, 1.19)	++
Yamagishi 2013	ARIC	US	plasma PL	IS	168	3870	0.86 (0.56, 1.32)	Highest versus lowest fourth	0.39	0.94 (0.80, 1.12)	+
**ALA (18:3n3)**											
Harris 2018	FHS-Offspring	US	RBC	IS	105	2500	1.42 (0.69, 2.92)	Highest versus lowest fifth	0.36	1.13 (0.88, 1.47)	+++
Daneshmand 2016	KIHD	Finland	serum	Stroke	202	1828	0.77 (0.51, 1.15)	Highest versus lowest fourth	0.39	0.90 (0.77, 1.06)	++
Fretts 2014	CHS	US	plasma PL	stroke	430	2709	0.97 (0.71, 1.31)	Highest versus lowest fifth	0.36	0.99 (0.89, 1.10)	++
Yamagishi 2013	ARIC	US	plasma PL	IS	168	3870	1.29 (0.82, 2.02)	Highest versus lowest fourth	0.39	1.11 (0.93, 1.32)	+
Yaemsiri 2013	WHI-OS	US	serum	IS	964	1928	0.92 (0.75, 1.12)	per SD increase	1.00	0.92 (0.75, 1.12)	++
Wiberg 2006	ULSAM 50	Sweden	serum CE	Stroke or TIA	421	2313	1.01 (0.92, 1.12)	per SD increase	1.00	1.01 (0.92, 1.12)	++
Simon 1995	MRFIT	US	serum PL	stroke	96	192	0.72 (0.53, 1.00)	per SD increase	1.00	0.72 (0.53, 1.00)	+
**LCn-3PUFA**											
Harris 2018	FHS-Offspring	US	RBC	IS	105	2500	0.51 (0.23, 1.14)	Highest versus lowest fifth	0.36	0.79 (0.59, 1.05)	+++
Daneshmand 2016	KIHD	Finland	serum	Stroke	202	1828	0.94 (0.64, 1.38)	Highest versus lowest fourth	0.39	0.98 (0.84, 1.14)	++
Yamagishi 2013	ARIC	US	plasma PL	IS	168	3870	0.85 (0.55, 1.29)	Highest versus lowest fourth	0.39	0.94 (0.79, 1.11)	+
Mozaffarian 2013	CHS	US	plasma PL	stroke	406	2692	0.75 (0.53, 1.06)	Highest versus lowest fifth	0.36	0.90 (0.80, 1.02)	++
Woodward 2011	SHHEC	Scotland	AT	stroke	219	3944	0.71 (0.48, 1.06)	Highest versus lowest fourth	0.39	0.87 (0.75, 1.02)	+++
**EPA (20:5n3)**											
Harris 2018	FHS-Offspring	US	RBC	IS	105	2500	0.95 (0.43, 2.11)	Highest versus lowest fifth	0.36	0.98 (0.74, 1.30)	+++
Saber 2017	CHS	US	plasma PL	IS	516	3675	1.03 (0.80, 1.31)	Highest versus lowest fourth	0.39	1.01 (0.92, 1.11)	++
Saber 2017	HPFS	US	RBC	IS	80	160	3.86 (0.88, 17.00)	Highest versus lowest fourth	0.39	1.70 (0.95, 3.05)	++
Saber 2017	NHS	US	RBC	IS	357	714	0.61 (0.37, 0.98)	Highest versus lowest fourth	0.39	0.82 (0.68, 1.00)	++
Daneshmand 2016	KIHD	Finland	serum	Stroke	202	1828	1.20 (0.81, 1.79)	Highest versus lowest fourth	0.39	1.07 (0.92, 1.26)	++

Yamagishi 2013	ARIC	US	plasma PL	IS	168	3870	1.18 (0.78, 1.78)	Highest versus lowest fourth	0.39	1.07 (0.91, 1.26)	+
Yaemsiri 2013	WHI-OS	US	serum	IS	964	1928	0.88 (0.73, 1.06)	per SD increase	1.00	0.88 (0.73, 1.06)	++
Wiberg 2006	ULSAM 50	Sweden	serum CE	Stroke or TIA	421	2313	1.05 (0.95, 1.16)	per SD increase	1.00	1.05 (0.95, 1.16)	++
Simon 1995	MRFIT	US	serum PL	stroke	96	192	1.00 (0.73, 1.36)	per SD increase	1.00	1.00 (0.73, 1.36)	+
**DPA (22:5n3)**											
Harris 2018	FHS-Offspring	US	RBC	IS	105	2500	1.17 (0.52, 2.66)	Highest versus lowest fifth	0.36	1.06 (0.79, 1.42)	+++
Saber 2017	CHS	US	plasma PL	IS	516	3675	0.71 (0.55, 0.91)	Highest versus lowest fourth	0.39	0.87 (0.79, 0.96)	++
Saber 2017	HPFS	US	RBC	IS	80	160	0.73 (0.18, 2.99)	Highest versus lowest fourth	0.39	0.88 (0.51, 1.54)	++
Saber 2017	NHS	US	RBC	IS	357	714	0.86 (0.48, 1.53)	Highest versus lowest fourth	0.39	0.94 (0.75, 1.18)	++
Daneshmand 2016	KIHD	Finland	serum	Stroke	202	1828	0.98 (0.66, 1.45)	Highest versus lowest fourth	0.39	0.99 (0.85, 1.16)	++
Yaemsiri 2013	WHI-OS	US	serum	IS	964	1928	0.75 (0.62, 0.91)	per SD increase	1.00	0.75 (0.62, 0.91)	++
Woodward 2011	SHHEC	Scotland	AT	stroke	219	3944	0.74 (0.50, 1.11)	Highest versus lowest fourth	0.39	0.89 (0.76, 1.04)	+++
Simon 1995	MRFIT	US	serum PL	stroke	96	192	0.78 (0.56, 1.09)	per SD increase	1.00	0.78 (0.56, 1.09)	+
**DHA (22:6n3)**											
Satizabal 2018	3C Study	France	plasma	Stroke	51	1313	0.99 (0.74, 1.32)	per SD increase	1.00	0.99 (0.74, 1.32)	++
Holmes 2018	CKB	China	plasma	IS	1146	2612	0.89 (0.81, 0.98)	per SD increase	1.00	0.89 (0.81, 0.98)	+++
Satizabal 2018	FHS	US	plasma PL	Stroke	81	906	0.89 (0.71, 1.11)	per SD increase	1.00	0.89 (0.71, 1.11)	++
Harris 2018	FHS-Offspring	US	RBC	IS	105	2500	0.41 (0.18, 0.93)	Highest versus lowest fifth	0.36	0.73 (0.54, 0.98)	+++
Saber 2017	CHS	US	plasma PL	IS	516	3675	0.83 (0.65, 1.06)	Highest versus lowest fourth	0.39	0.93 (0.84, 1.02)	++
Saber 2017	HPFS	US	RBC	IS	80	160	2.33 (0.62, 8.83)	Highest versus lowest fourth	0.39	1.40 (0.83, 2.35)	++
Saber 2017	NHS	US	RBC	IS	357	714	0.55 (0.32, 0.96)	Highest versus lowest fourth	0.39	0.79 (0.64, 0.98)	++
Daneshmand 2016	KIHD	Finland	serum	Stroke	202	1828	1.08 (0.73, 1.58)	Highest versus lowest fourth	0.39	1.03 (0.89, 1.20)	++
Yamagishi 2013	ARIC	US	plasma PL	IS	168	3870	0.69 (0.46, 1.06)	Highest versus lowest fourth	0.39	0.86 (0.73, 1.02)	+
Yaemsiri 2013	WHI-OS	US	serum	IS	964	1928	0.75 (0.62, 0.91)	per SD increase	1.00	0.75 (0.62, 0.91)	++
Woodward 2011	SHHEC	Scotland	AT	stroke	219	3944	0.76 (0.51, 1.13)	Highest versus lowest fourth	0.39	0.90 (0.77, 1.05)	+++
Wiberg 2006	ULSAM 50	Sweden	serum CE	Stroke or TIA	421	2313	1.01 (0.91, 1.12)	per SD increase	1.00	1.01 (0.91, 1.12)	++
Simon 1995	MRFIT	US	serum PL	stroke	96	192	0.94 (0.70, 1.27)	per SD increase	1.00	0.94 (0.70, 1.27)	+
**Omega-3 index (EPA+DHA)**											
Harris 2018	FHS-Offspring	US	RBC	IS	105	2500	0.45 (0.20, 1.03)	Highest versus lowest fifth	0.36	0.75 (0.56, 1.01)	+++
Wennberg 2007	VIP+MONICA	Sweden	plasma PL +RBC	Stroke	369	1107	1.05 (0.89, 1.24)	per SD increase	1.00	1.05 (0.89, 1.24)	++
**Total N6 PUFA**											
Holmes 2018	CKB	China	plasma	IS	1146	2612	1.05 (0.96, 1.15)	per SD increase	1.00	1.05 (0.96, 1.15)	+++
Harris 2018	FHS-Offspring	US	RBC	IS	105	2500	0.77 (0.32, 1.86)	Highest versus lowest fifth	0.36	0.91 (0.67, 1.25)	+++
Daneshmand 2016	KIHD	Finland	serum	Stroke	202	1828	0.89 (0.59, 1.34)	Highest versus lowest fourth	0.39	0.96 (0.81, 1.12)	++
Yamagishi 2013	ARIC	US	plasma PL	IS	168	3870	0.93 (0.60, 1.45)	Highest versus lowest fourth	0.39	0.97 (0.82, 1.16)	+
Woodward 2011	SHHEC	Scotland	AT	stroke	219	3944	0.75 (0.49, 1.14)	Highest versus lowest fourth	0.39	0.89 (0.76, 1.05)	+++
**LA (18:2n6)**											
Marklund 2019	60YO	Sweden	serum CE	IS	155	4150	0.78 (0.49, 1.26)	per interquintile range (10-90th)	0.39	0.91 (0.75, 1.09)	++++*
Marklund 2019	AGES-Reykjavik	Iceland	plasma PL	IS	123	1195	1.16 (0.71, 1.89)	per interquintile range (10-90th)	0.39	1.06 (0.88, 1.28)	++++*
Marklund 2019	ARIC	US	plasma PL	IS	188	3749	1.32 (0.89, 1.96)	per interquintile range (10-90th)	0.39	1.11 (0.96, 1.30)	++++*
Marklund 2019	CCCC	Taiwan	plasma	Stroke	243	1838	0.43 (0.22, 0.82)	per interquintile range (10-90th)	0.39	0.72 (0.56, 0.93)	++++*
Marklund 2019	HPFS	US	plasma	Stroke	120	1510	0.55 (0.22, 1.38)	per interquintile range (10-90th)	0.39	0.79 (0.55, 1.13)	++++*
Marklund 2019	HS	Japan	serum	IS	97	3103	1.14 (0.58, 2.25)	per interquintile range (10-90th)	0.39	1.05 (0.81, 1.37)	++++*
Marklund 2019	MCCS	Australia	plasma PL	fatal IS	44	6265	0.44 (0.15, 1.29)	per interquintile range (10-90th)	0.39	0.73 (0.48, 1.10)	++++*
Marklund 2019	MESA	US	plasma PL	IS	53	2722	0.88 (0.27, 2.91)	per interquintile range (10-90th)	0.39	0.95 (0.60, 1.51)	++++*
Marklund 2019	NHS	US	plasma	Stroke	468	1970	0.84 (0.56, 1.26)	per interquintile range (10-90th)	0.39	0.93 (0.80, 1.09)	++++*
Marklund 2019	NSHDS III	Sweden	plasma PL	IS	85	317	0.46 (0.15, 1.47)	per interquintile range (10-90th)	0.39	0.74 (0.47, 1.15)	++++*
Marklund 2019	PIVUS	Sweden	serum PL	IS	37	835	1.16 (0.28, 4.85)	per interquintile range (10-90th)	0.39	1.06 (0.61, 1.85)	++++*
Marklund 2019	SHHEC	UK	AT	Stroke	290	4391	0.66 (0.46, 0.95)	per interquintile range (10-90th)	0.39	0.85 (0.74, 0.98)	++++*
Marklund 2019	ULSAM 70	Sweden	AT	IS	115	763	1.30 (0.83, 2.02)	per interquintile range (10-90th)	0.39	1.11 (0.93, 1.32)	++++*

Marklund 2019	WHI-MS	US	RBC	IS	295	5263	1.00 (0.71, 1.41)	per interquintile range (10-90th)	0.39	1.00 (0.87, 1.14)	++++*
Satizabal 2018	3C Study	France	plasma	Stroke	51	1313	0.94 (0.70, 1.26)	per SD increase	1.00	0.94 (0.70, 1.26)	++
Holmes 2018	CKB	China	plasma	IS	1146	2612	1.10 (1.00, 1.22)	per SD increase	1.00	1.10 (1.00, 1.22)	+++
Satizabal 2018	FHS	US	plasma PL	Stroke	81	906	0.92 (0.73, 1.15)	per SD increase	1.00	0.92 (0.73, 1.15)	++
Harris 2018	FHS-Offspring	US	RBC	IS	105	2500	1.09 (0.49, 2.39)	Highest versus lowest fifth	0.36	1.03 (0.78, 1.37)	+++
Daneshmand 2016	KIHD	Finland	serum	Stroke	202	1828	0.93 (0.62, 1.39)	Highest versus lowest fourth	0.39	0.97 (0.83, 1.14)	++
Wu 2014	CHS	US	plasma PL	stroke	463	2792	0.92 (0.67, 1.27)	Highest versus lowest fifth	0.36	0.97 (0.87, 1.09)	++++*
Yaemsiri 2013	WHI-OS	US	serum	IS	964	1928	0.94 (0.80, 1.10)	per SD increase	1.00	0.94 (0.80, 1.10)	++
Wiberg 2006	ULSAM 50	Sweden	serum CE	Stroke or TIA	421	2313	0.84 (0.76, 0.93)	per SD increase	1.00	0.84 (0.76, 0.93)	++
Iso 2002	JapanCRS	Japan	serum	Stroke	197	788	0.65 (0.54, 0.79)	per SD increase	1.00	0.65 (0.54, 0.79)	+
Simon 1995	MRFIT	US	serum PL	stroke	96	192	0.95 (0.70, 1.28)	per SD increase	1.00	0.95 (0.70, 1.28)	+
**GLA (18:3n6)**											
Harris 2018	FHS-Offspring	US	RBC	IS	105	2500	0.81 (0.41, 1.61)	Highest versus lowest fifth	0.36	0.93 (0.73, 1.18)	+++
Daneshmand 2016	KIHD	Finland	serum	Stroke	202	1828	1.14 (0.78, 1.68)	Highest versus lowest fourth	0.39	1.05 (0.91, 1.22)	++
Wu 2014	CHS	US	plasma PL	stroke	463	2792	1.03 (0.76, 1.41)	Highest versus lowest fifth	0.36	1.01 (0.91, 1.13)	++++*
Yamagishi 2013	ARIC	US	plasma PL	IS	168	3870	1.26 (0.80, 1.99)	Highest versus lowest fourth	0.39	1.10 (0.92, 1.31)	+
Yaemsiri 2013	WHI-OS	US	serum	IS	964	1928	0.91 (0.77, 1.08)	per SD increase	1.00	0.91 (0.77, 1.08)	++
Woodward 2011	SHHEC	Scotland	AT	stroke	219	3944	1.44 (0.98, 2.13)	Highest versus lowest fourth	0.39	1.15 (0.99, 1.34)	+++
Wiberg 2006	ULSAM 50	Sweden	serum CE	Stroke or TIA	421	2313	1.07 (0.97, 1.19)	per SD increase	1.00	1.07 (0.97, 1.19)	++
**EDA (20:2n6)**											
Harris 2018	FHS-Offspring	US	RBC	IS	105	2500	1.16 (0.59, 2.28)	Highest versus lowest fifth	0.36	1.05 (0.83, 1.34)	+++
Yaemsiri 2013	WHI-OS	US	serum	IS	964	1928	1.02 (0.86, 1.21)	per SD increase	1.00	1.02 (0.86, 1.21)	++
Simon 1995	MRFIT	US	serum PL	stroke	96	192	1.11 (0.81, 1.51)	per SD increase	1.00	1.11 (0.81, 1.51)	+
**DGLA (20:3n6)**											
Harris 2018	FHS-Offspring	US	RBC	IS	105	2500	0.90 (0.42, 1.94)	Highest versus lowest fifth	0.36	0.96 (0.73, 1.27)	+++
Daneshmand 2016	KIHD	Finland	serum	Stroke	202	1828	1.02 (0.67, 1.54)	Highest versus lowest fourth	0.39	1.01 (0.86, 1.19)	++
Wu 2014	CHS	US	plasma PL	stroke	463	2792	0.87 (0.63, 1.18)	Highest versus lowest fifth	0.36	0.95 (0.85, 1.06)	++++*
Yamagishi 2013	ARIC	US	plasma PL	IS	168	3870	1.17 (0.78, 1.78)	Highest versus lowest fourth	0.39	1.06 (0.90, 1.25)	+
Yaemsiri 2013	WHI-OS	US	serum	IS	964	1928	0.94 (0.79, 1.12)	per SD increase	1.00	0.94 (0.79, 1.12)	++
Woodward 2011	SHHEC	Scotland	AT	stroke	219	3944	0.67 (0.44, 1.04)	Highest versus lowest fourth	0.39	0.85 (0.72, 1.01)	+++
Wiberg 2006	ULSAM 50	Sweden	serum CE	Stroke or TIA	421	2313	1.06 (0.96, 1.17)	per SD increase	1.00	1.06 (0.96, 1.17)	++
Simon 1995	MRFIT	US	serum PL	stroke	96	192	1.19 (0.88, 1.61)	per SD increase	1.00	1.19 (0.88, 1.61)	+
**AA (20:4n6)**											
Marklund 2019	60YO	Sweden	serum CE	IS	155	4150	1.08 (0.70, 1.65)	per interquintile range (10-90th)	0.39	1.03 (0.87, 1.22)	++++*
Marklund 2019	AGES-Reykjavik	Iceland	plasma PL	IS	123	1195	0.67 (0.39, 1.15)	per interquintile range (10-90th)	0.39	0.86 (0.69, 1.06)	++++*
Marklund 2019	ARIC	US	plasma PL	IS	188	3749	0.95 (0.67, 1.35)	per interquintile range (10-90th)	0.39	0.98 (0.86, 1.12)	++++*
Marklund 2019	CCCC	Taiwan	plasma	Stroke	243	1838	1.58 (1.02, 2.45)	per interquintile range (10-90th)	0.39	1.20 (1.01, 1.42)	++++*
Marklund 2019	HPFS	US	plasma	Stroke	120	1510	0.68 (0.22, 2.14)	per interquintile range (10-90th)	0.39	0.86 (0.55, 1.34)	++++*
Marklund 2019	HS	Japan	serum	IS	97	3103	0.80 (0.43, 1.51)	per interquintile range (10-90th)	0.39	0.92 (0.72, 1.17)	++++*
Marklund 2019	MCCS	Australia	plasma PL	fatal IS	44	6265	1.22 (0.52, 2.84)	per interquintile range (10-90th)	0.39	1.08 (0.78, 1.50)	++++*
Marklund 2019	MESA	US	plasma PL	IS	53	2722	0.34 (0.11, 1.02)	per interquintile range (10-90th)	0.39	0.66 (0.43, 1.01)	++++*
Marklund 2019	NHS	US	plasma	Stroke	468	1970	0.49 (0.28, 0.84)	per interquintile range (10-90th)	0.39	0.76 (0.61, 0.94)	++++*
Marklund 2019	NSHDS III	Sweden	plasma PL	IS	85	317	0.58 (0.21, 1.60)	per interquintile range (10-90th)	0.39	0.81 (0.54, 1.20)	++++*
Marklund 2019	PIVUS	Sweden	serum PL	IS	37	835	1.00 (0.37, 2.76)	per interquintile range (10-90th)	0.39	1.00 (0.68, 1.48)	++++*
Marklund 2019	SHHEC	UK	AT	Stroke	290	4391	0.95 (0.76, 1.19)	per interquintile range (10-90th)	0.39	0.98 (0.90, 1.07)	++++*
Marklund 2019	ULSAM 70	Sweden	AT	IS	115	763	0.67 (0.38, 1.16)	per interquintile range (10-90th)	0.39	0.86 (0.69, 1.06)	++++*
Marklund 2019	WHI-MS	US	RBC	IS	295	5263	1.36 (0.87, 2.13)	per interquintile range (10-90th)	0.39	1.13 (0.95, 1.34)	++++*
Satizabal 2018	3C Study	France	plasma	Stroke	51	1313	0.97 (0.73, 1.30)	per SD increase	1.00	0.97 (0.73, 1.30)	++
Satizabal 2018	FHS	US	plasma PL	Stroke	81	906	1.06 (0.84, 1.34)	per SD increase	1.00	1.06 (0.84, 1.34)	++
Harris 2018	FHS-Offspring	US	RBC	IS	105	2500	1.42 (0.73, 2.77)	Highest versus lowest fifth	0.36	1.13 (0.89, 1.44)	+++

Daneshmand 2016	KIHD	Finland	serum	Stroke	202	1828	1.13 (0.77, 1.67)	Highest versus lowest fourth	0.39	1.05 (0.90, 1.22)	++
Wu 2014	CHS	US	plasma PL	stroke	463	2792	1.06 (0.78, 1.45)	Highest versus lowest fifth	0.36	1.02 (0.91, 1.14)	++++*
Yaemsiri 2013	WHI-OS	US	serum	IS	964	1928	0.82 (0.67, 0.99)	per SD increase	1.00	0.82 (0.67, 0.99)	++
Wiberg 2006	ULSAM 50	Sweden	serum CE	Stroke or TIA	421	2313	0.98 (0.88, 1.08)	per SD increase	1.00	0.98 (0.88, 1.08)	++
Iso 2002	JapanCRS	Japan	serum	Stroke	197	788	0.78 (0.65, 0.93)	per SD increase	1.00	0.78 (0.65, 0.93)	+
Simon 1995	MRFIT	US	serum PL	stroke	96	192	0.85 (0.64, 1.14)	per SD increase	1.00	0.85 (0.64, 1.14)	+
**DTA (22:4n6)**											
Harris 2018	FHS-Offspring	US	RBC	IS	105	2500	2.11 (0.97, 4.59)	Highest versus lowest fifth	0.36	1.31 (0.99, 1.72)	+++
Yaemsiri 2013	WHI-OS	US	serum	IS	964	1928	0.91 (0.69, 1.22)	per SD increase	1.00	0.91 (0.69, 1.22)	++
Simon 1995	MRFIT	US	serum PL	stroke	96	192	0.82 (0.61, 1.12)	per SD increase	1.00	0.82 (0.61, 1.12)	+
**DPA (22:5n6)**											
Harris 2018	FHS-Offspring	US	RBC	IS	105	2500	2.01 (0.93, 4.35)	Highest versus lowest fifth	0.36	1.28 (0.97, 1.69)	+++
Yaemsiri 2013	WHI-OS	US	serum	IS	964	1928	0.86 (0.72, 1.04)	per SD increase	1.00	0.86 (0.72, 1.04)	++
Simon 1995	MRFIT	US	serum PL	stroke	96	192	1.10 (0.81, 1.51)	per SD increase	1.00	1.10 (0.81, 1.51)	+
**trans-16:1**											
Otto 2018	CHS	US	plasma PL	stroke	529	2907	1.05 (0.79, 1.40)	Highest versus lowest fifth	0.36	1.02 (0.92, 1.13)	+
Yakoob 2014	HPFS	US	plasma	stroke	122	244	0.76 (0.55, 1.04)	per SD increase	1.00	0.76 (0.55, 1.04)	++
Yakoob 2014	NHS	US	plasma	stroke	472	944	0.99 (0.83, 1.18)	per SD increase	1.00	0.99 (0.83, 1.18)	++
Yaemsiri 2013	WHI-OS	US	serum	IS	964	1928	1.02 (0.86, 1.22)	per SD increase	1.00	1.02 (0.86, 1.22)	++
**trans-18:1**											
Yaemsiri 2013	WHI-OS	US	serum	IS	964	1928	1.13 (0.95, 1.34)	per SD increase	1.00	1.13 (0.95, 1.34)	++
**trans-18:2**											
Yaemsiri 2013	WHI-OS	US	serum	IS	964	1928	1.34 (1.00, 1.79)	per SD increase	1.00	1.34 (1.00, 1.79)	++

RR: Relative risk; SD: Standard deviation. See Supplementary eTable 17. footnote for other abbreviations.
Adjustment: +, adjusted for sex, age, and/or other demographic information; ++, adjusted for preceding plus any non-lipid conventional risk factors; +++, adjusted for preceding plus lipids; ++++, adjusted for preceding plus other circulating fatty acids (* not included lipids).

Supplementary eTable 20. Further characteristics of non-duplicated associations of fatty acid biomarkers with ischaemic stroke included in the updated meta-analyses

Paper	Data source	Region	Biomarker	Outcome	N of events	N	reported RR (95%CI)	Scale of RR reported by study	Conversion factor	RR (95%CI) per 1-SD	Adjustment
**Total SFA**											
Holmes 2018	CKB	China	plasma	IS	1146	2612	0.84 (0.77, 0.93)	per SD increase	1.00	0.84 (0.77, 0.93)	+++
Yamagishi 2013	ARIC	US	plasma PL	IS	168	3870	1.64 (1.05, 2.57)	Highest versus lowest fourth	0.39	1.22 (1.02, 1.45)	+
**Even-chain SFA**											
Iso 2002	JapanCRS	Japan	serum	IS	122	488	1.35 (1.01, 1.79)	per SD increase	1.00	1.35 (1.01, 1.79)	+++
**Myristic acid (14:0)**											
Yakoob 2014	HPFS	US	plasma	IS	80	160	1.34 (0.12, 15.13)	Highest versus lowest fourth	0.39	1.12 (0.43, 2.91)	++++*
Yakoob 2014	NHS	US	plasma	IS	371	742	0.86 (0.45, 1.66)	Highest versus lowest fourth	0.39	0.94 (0.73, 1.22)	++++*
Yamagishi 2013	ARIC	US	plasma PL	IS	168	3870	1.29 (0.83, 2.01)	Highest versus lowest fourth	0.39	1.11 (0.93, 1.32)	+
Yaemsiri 2013	WHI-OS	US	serum	IS	964	1928	1.16 (0.97, 1.38)	per SD increase	1.00	1.16 (0.97, 1.38)	++
Wiberg 2006	ULSAM 50	Sweden	serum CE	IS	308	2313	1.12 (1.00, 1.25)	per SD increase	1.00	1.12 (1.00, 1.25)	++
Iso 2002	JapanCRS	Japan	serum	IS	122	488	1.47 (1.19, 1.82)	per SD increase	1.00	1.47 (1.19, 1.82)	+
**Palmitic acid (16:0)**											
Satizabal 2018	3C Study	France	plasma	IS	38	1313	1.36 (1.06, 1.76)	per SD increase	1.00	1.36 (1.06, 1.76)	++
Satizabal 2018	FHS	US	plasma PL	IS	75	906	1.25 (0.99, 1.57)	per SD increase	1.00	1.25 (0.99, 1.57)	++
Yamagishi 2013	ARIC	US	plasma PL	IS	168	3870	1.30 (0.84, 2.02)	Highest versus lowest fourth	0.39	1.11 (0.93, 1.32)	+
Yaemsiri 2013	WHI-OS	US	serum	IS	964	1928	1.23 (1.02, 1.47)	per SD increase	1.00	1.23 (1.02, 1.47)	++
Wiberg 2006	ULSAM 50	Sweden	serum CE	IS	308	2313	1.15 (1.02, 1.29)	per SD increase	1.00	1.15 (1.02, 1.29)	++
Iso 2002	JapanCRS	Japan	serum	IS	122	488	1.47 (1.19, 1.82)	per SD increase	1.00	1.47 (1.19, 1.82)	+
**Stearic acid (18:0)**											
Yamagishi 2013	ARIC	US	plasma PL	IS	168	3870	1.17 (0.74, 1.84)	Highest versus lowest fourth	0.39	1.06 (0.89, 1.27)	+
Yaemsiri 2013	WHI-OS	US	serum	IS	964	1928	0.93 (0.77, 1.12)	per SD increase	1.00	0.93 (0.77, 1.12)	++
Wiberg 2006	ULSAM 50	Sweden	serum CE	IS	308	2313	1.02 (0.90, 1.16)	per SD increase	1.00	1.02 (0.90, 1.16)	++
**Pentadecanoic acid (15:0)**											
Trieu 2021	60YO	Sweden	serum CE	IS	192	4150	0.94 (0.81, 1.10)	per SD increase	1.00	0.94 (0.81, 1.10)	++
Yakoob 2014	HPFS	US	plasma	IS	80	160	0.60 (0.06, 6.14)	Highest versus lowest fourth	0.39	0.82 (0.33, 2.03)	++++*
Yakoob 2014	NHS	US	plasma	IS	371	742	0.79 (0.45, 1.38)	Highest versus lowest fourth	0.39	0.91 (0.73, 1.14)	++++*
Yamagishi 2013	ARIC	US	plasma PL	IS	168	3870	0.89 (0.60, 1.33)	Highest versus lowest fourth	0.39	0.96 (0.82, 1.12)	+
Yaemsiri 2013	WHI-OS	US	serum	IS	964	1928	1.11 (0.90, 1.35)	per SD increase	1.00	1.11 (0.90, 1.35)	++
**Heptadecanoic acid (17:0)**											
Yakoob 2014	HPFS	US	plasma	IS	80	160	0.40 (0.08, 1.96)	Highest versus lowest fourth	0.39	0.70 (0.37, 1.31)	++++*
Yakoob 2014	NHS	US	plasma	IS	371	742	1.08 (0.66, 1.76)	Highest versus lowest fourth	0.39	1.03 (0.85, 1.25)	++++*
Yaemsiri 2013	WHI-OS	US	serum	IS	964	1928	1.00 (0.84, 1.19)	per SD increase	1.00	1.00 (0.84, 1.19)	++
**Total MUFA**											
Holmes 2018	CKB	China	plasma	IS	1146	2612	1.06 (0.96, 1.16)	per SD increase	1.00	1.06 (0.96, 1.16)	+++
Yamagishi 2013	ARIC	US	plasma PL	IS	168	3870	1.42 (0.92, 2.18)	Highest versus lowest fourth	0.39	1.15 (0.97, 1.36)	+
**Palmitoleic acid (16:1n7)**											
Yamagishi 2013	ARIC	US	plasma PL	IS	168	3870	1.52 (0.99, 2.34)	Highest versus lowest fourth	0.39	1.18 (1.00, 1.40)	+
Yaemsiri 2013	WHI-OS	US	serum	IS	964	1928	1.13 (0.95, 1.34)	per SD increase	1.00	1.13 (0.95, 1.34)	++
Wiberg 2006	ULSAM 50	Sweden	serum CE	IS	308	2313	1.22 (1.08, 1.37)	per SD increase	1.00	1.22 (1.08, 1.37)	++
Iso 2002	JapanCRS	Japan	serum	IS	122	488	1.51 (1.21, 1.89)	per SD increase	1.00	1.51 (1.21, 1.89)	+
**Oleic acid (18:1n9)**											
Yamagishi 2013	ARIC	US	plasma PL	IS	168	3870	1.38 (0.88, 2.15)	Highest versus lowest fourth	0.39	1.14 (0.95, 1.35)	+
Yaemsiri 2013	WHI-OS	US	serum	IS	964	1928	1.18 (0.99, 1.41)	per SD increase	1.00	1.18 (0.99, 1.41)	++
Wiberg 2006	ULSAM 50	Sweden	serum CE	IS	308	2313	1.27 (1.13, 1.43)	per SD increase	1.00	1.27 (1.13, 1.43)	++
Iso 2002	JapanCRS	Japan	serum	IS	122	488	1.23 (1.01, 1.51)	per SD increase	1.00	1.23 (1.01, 1.51)	+

**Eicosenoic acid (20:1n9)**											
Yaemsiri 2013	WHI-OS	US	serum	IS	964	1928	1.12 (0.81, 1.55)	per SD increase	1.00	1.12 (0.81, 1.55)	++
**Nervonic acid (24:1n9)**											
Yaemsiri 2013	WHI-OS	US	serum	IS	964	1928	0.88 (0.66, 1.17)	per SD increase	1.00	0.88 (0.66, 1.17)	++
**Total PUFA**											
Holmes 2018	CKB	China	plasma	IS	1146	2612	1.05 (0.96, 1.15)	per SD increase	1.00	1.05 (0.96, 1.15)	+++
**Total N3 PUFA**											
Holmes 2018	CKB	China	plasma	IS	1146	2612	1.02 (0.92, 1.12)	per SD increase	1.00	1.02 (0.92, 1.12)	+++
Daneshmand 2016	KIHD	Finland	serum	IS	153	1828	1.13 (0.73, 1.74)	Highest versus lowest fourth	0.39	1.05 (0.88, 1.24)	++
Yamagishi 2013	ARIC	US	plasma PL	IS	168	3870	0.86 (0.56, 1.32)	Highest versus lowest fourth	0.39	0.94 (0.80, 1.12)	+
**ALA (18:3n3)**											
Harris 2018	FHS-Offspring	US	RBC	IS	105	2500	1.42 (0.69, 2.92)	Highest versus lowest fifth	0.36	1.13 (0.88, 1.47)	+++
Daneshmand 2016	KIHD	Finland	serum	IS	153	1828	0.90 (0.58, 1.41)	Highest versus lowest fourth	0.39	0.96 (0.81, 1.14)	++
Fretts 2014	CHS	US	plasma PL	IS	337	2709	0.97 (0.69, 1.36)	Highest versus lowest fifth	0.36	0.99 (0.88, 1.12)	++
Yamagishi 2013	ARIC	US	plasma PL	IS	168	3870	1.29 (0.82, 2.02)	Highest versus lowest fourth	0.39	1.11 (0.93, 1.32)	+
Yaemsiri 2013	WHI-OS	US	serum	IS	964	1928	0.92 (0.75, 1.12)	per SD increase	1.00	0.92 (0.75, 1.12)	++
Wiberg 2006	ULSAM 50	Sweden	serum CE	IS	308	2313	1.03 (0.92, 1.16)	per SD increase	1.00	1.03 (0.92, 1.16)	++
**LCn-3PUFA**											
Harris 2018	FHS-Offspring	US	RBC	IS	105	2500	0.51 (0.23, 1.14)	Highest versus lowest fifth	0.36	0.79 (0.59, 1.05)	+++
Daneshmand 2016	KIHD	Finland	serum	IS	153	1828	0.98 (0.64, 1.51)	Highest versus lowest fourth	0.39	0.99 (0.84, 1.17)	++
Yamagishi 2013	ARIC	US	plasma PL	IS	168	3870	0.85 (0.55, 1.29)	Highest versus lowest fourth	0.39	0.94 (0.79, 1.11)	+
Mozaffarian 2013	CHS	US	plasma PL	IS	319	2692	0.63 (0.43, 0.94)	Highest versus lowest fifth	0.36	0.85 (0.74, 0.97)	++
**EPA (20:5n3)**											
Harris 2018	FHS-Offspring	US	RBC	IS	105	2500	0.95 (0.43, 2.11)	Highest versus lowest fifth	0.36	0.98 (0.74, 1.30)	+++
Saber 2017	CHS	US	plasma PL	IS	516	3675	1.03 (0.80, 1.31)	Highest versus lowest fourth	0.39	1.01 (0.92, 1.11)	++
Saber 2017	HPFS	US	RBC	IS	80	160	3.86 (0.88, 17.00)	Highest versus lowest fourth	0.39	1.70 (0.95, 3.05)	++
Saber 2017	NHS	US	RBC	IS	357	714	0.61 (0.37, 0.98)	Highest versus lowest fourth	0.39	0.82 (0.68, 1.00)	++
Daneshmand 2016	KIHD	Finland	serum	IS	153	1828	1.27 (0.80, 2.00)	Highest versus lowest fourth	0.39	1.10 (0.92, 1.32)	++
Yamagishi 2013	ARIC	US	plasma PL	IS	168	3870	1.18 (0.78, 1.78)	Highest versus lowest fourth	0.39	1.07 (0.91, 1.26)	+
Yaemsiri 2013	WHI-OS	US	serum	IS	964	1928	0.88 (0.73, 1.06)	per SD increase	1.00	0.88 (0.73, 1.06)	++
Wiberg 2006	ULSAM 50	Sweden	serum CE	IS	308	2313	1.07 (0.95, 1.20)	per SD increase	1.00	1.07 (0.95, 1.20)	++
**DPA (22:5n3)**											
Harris 2018	FHS-Offspring	US	RBC	IS	105	2500	1.17 (0.52, 2.66)	Highest versus lowest fifth	0.36	1.06 (0.79, 1.42)	+++
Saber 2017	CHS	US	plasma PL	IS	516	3675	0.71 (0.55, 0.91)	Highest versus lowest fourth	0.39	0.87 (0.79, 0.96)	++
Saber 2017	HPFS	US	RBC	IS	80	160	0.73 (0.18, 2.99)	Highest versus lowest fourth	0.39	0.88 (0.51, 1.54)	++
Saber 2017	NHS	US	RBC	IS	357	714	0.86 (0.48, 1.53)	Highest versus lowest fourth	0.39	0.94 (0.75, 1.18)	++
Daneshmand 2016	KIHD	Finland	serum	IS	153	1828	1.20 (0.76, 1.90)	Highest versus lowest fourth	0.39	1.07 (0.90, 1.29)	++
Yaemsiri 2013	WHI-OS	US	serum	IS	964	1928	0.75 (0.62, 0.91)	per SD increase	1.00	0.75 (0.62, 0.91)	++
**DHA (22:6n3)**											
Satizabal 2018	3C Study	France	plasma	IS	38	1313	0.87 (0.63, 1.21)	per SD increase	1.00	0.87 (0.63, 1.21)	++
Holmes 2018	CKB	China	plasma	IS	1146	2612	0.89 (0.81, 0.98)	per SD increase	1.00	0.89 (0.81, 0.98)	+++
Satizabal 2018	FHS	US	plasma PL	IS	75	906	0.91 (0.73, 1.15)	per SD increase	1.00	0.91 (0.73, 1.15)	++
Harris 2018	FHS-Offspring	US	RBC	IS	105	2500	0.41 (0.18, 0.93)	Highest versus lowest fifth	0.36	0.73 (0.54, 0.98)	+++
Saber 2017	CHS	US	plasma PL	IS	516	3675	0.83 (0.65, 1.06)	Highest versus lowest fourth	0.39	0.93 (0.84, 1.02)	++
Saber 2017	HPFS	US	RBC	IS	80	160	2.33 (0.62, 8.83)	Highest versus lowest fourth	0.39	1.40 (0.83, 2.35)	++
Saber 2017	NHS	US	RBC	IS	357	714	0.55 (0.32, 0.96)	Highest versus lowest fourth	0.39	0.79 (0.64, 0.98)	++
Daneshmand 2016	KIHD	Finland	serum	IS	153	1828	1.01 (0.70, 1.69)	Highest versus lowest fourth	0.39	1.00 (0.84, 1.19)	++
Yamagishi 2013	ARIC	US	plasma PL	IS	168	3870	0.69 (0.46, 1.06)	Highest versus lowest fourth	0.39	0.86 (0.73, 1.02)	+
Yaemsiri 2013	WHI-OS	US	serum	IS	964	1928	0.75 (0.62, 0.91)	per SD increase	1.00	0.75 (0.62, 0.91)	++
Wiberg 2006	ULSAM 50	Sweden	serum CE	IS	308	2313	1.01 (0.90, 1.14)	per SD increase	1.00	1.01 (0.90, 1.14)	++

**Omega-3 index (EPA+DHA)**											
Harris 2018	FHS-Offspring	US	RBC	IS	105	2500	0.45 (0.20, 1.03)	Highest versus lowest fifth	0.36	0.75 (0.56, 1.01)	+++
**Total N6 PUFA**											
Holmes 2018	CKB	China	plasma	IS	1146	2612	1.05 (0.96, 1.15)	per SD increase	1.00	1.05 (0.96, 1.15)	+++
Harris 2018	FHS-Offspring	US	RBC	IS	105	2500	0.77 (0.32, 1.86)	Highest versus lowest fifth	0.36	0.91 (0.67, 1.25)	+++
Daneshmand 2016	KIHD	Finland	serum	IS	153	1828	0.99 (0.63, 1.57)	Highest versus lowest fourth	0.39	1.00 (0.83, 1.19)	++
Yamagishi 2013	ARIC	US	plasma PL	IS	168	3870	0.93 (0.60, 1.45)	Highest versus lowest fourth	0.39	0.97 (0.82, 1.16)	+
**LA (18:2n6)**											
Marklund 2019	60YO	Sweden	serum CE	IS	155	4150	0.78 (0.49, 1.26)	per interquintile range (10-90th)	0.39	0.91 (0.75, 1.09)	++++*
Marklund 2019	AGES-Reykjavik	Iceland	plasma PL	IS	123	1195	1.16 (0.71, 1.89)	per interquintile range (10-90th)	0.39	1.06 (0.88, 1.28)	++++*
Marklund 2019	ARIC	US	plasma PL	IS	188	3749	1.32 (0.89, 1.96)	per interquintile range (10-90th)	0.39	1.11 (0.96, 1.30)	++++*
Marklund 2019	CHS	US	plasma PL	IS	408	2907	0.95 (0.68, 1.34)	per interquintile range (10-90th)	0.39	0.98 (0.86, 1.12)	++++*
Marklund 2019	HS	Japan	serum	IS	97	3103	1.14 (0.58, 2.25)	per interquintile range (10-90th)	0.39	1.05 (0.81, 1.37)	++++*
Marklund 2019	MCCS	Australia	plasma PL	fatal IS	44	6265	0.44 (0.15, 1.29)	per interquintile range (10-90th)	0.39	0.73 (0.48, 1.10)	++++*
Marklund 2019	MESA	US	plasma PL	IS	53	2722	0.88 (0.27, 2.91)	per interquintile range (10-90th)	0.39	0.95 (0.60, 1.51)	++++*
Marklund 2019	NSHDS III	Sweden	plasma PL	IS	85	317	0.46 (0.15, 1.47)	per interquintile range (10-90th)	0.39	0.74 (0.47, 1.15)	++++*
Marklund 2019	PIVUS	Sweden	serum PL	IS	37	835	1.16 (0.28, 4.85)	per interquintile range (10-90th)	0.39	1.06 (0.61, 1.85)	++++*
Marklund 2019	ULSAM 50	Sweden	serum CE	IS	313	1992	0.63 (0.44, 0.91)	per interquintile range (10-90th)	0.39	0.84 (0.72, 0.96)	++++*
Marklund 2019	ULSAM 70	Sweden	AT	IS	115	763	1.30 (0.83, 2.02)	per interquintile range (10-90th)	0.39	1.11 (0.93, 1.32)	++++*
Marklund 2019	WHI-MS	US	RBC	IS	295	5263	1.00 (0.71, 1.41)	per interquintile range (10-90th)	0.39	1.00 (0.87, 1.14)	++++*
Satizabal 2018	3C Study	France	plasma	IS	38	1313	0.85 (0.61, 1.19)	per SD increase	1.00	0.85 (0.61, 1.19)	++
Holmes 2018	CKB	China	plasma	IS	1146	2612	1.10 (1.00, 1.22)	per SD increase	1.00	1.10 (1.00, 1.22)	+++
Satizabal 2018	FHS	US	plasma PL	IS	75	906	0.98 (0.77, 1.24)	per SD increase	1.00	0.98 (0.77, 1.24)	++
Harris 2018	FHS-Offspring	US	RBC	IS	105	2500	1.09 (0.49, 2.39)	Highest versus lowest fifth	0.36	1.03 (0.78, 1.37)	+++
Daneshmand 2016	KIHD	Finland	serum	IS	153	1828	1.07 (0.68, 1.67)	Highest versus lowest fourth	0.39	1.03 (0.86, 1.23)	++
Yaemsiri 2013	WHI-OS	US	serum	IS	964	1928	0.94 (0.80, 1.10)	per SD increase	1.00	0.94 (0.80, 1.10)	++
Iso 2002	JapanCRS	Japan	serum	IS	122	488	0.60 (0.46, 0.77)	per SD increase	1.00	0.60 (0.46, 0.77)	+
**GLA (18:3n6)**											
Harris 2018	FHS-Offspring	US	RBC	IS	105	2500	0.81 (0.41, 1.61)	Highest versus lowest fifth	0.36	0.93 (0.73, 1.18)	+++
Daneshmand 2016	KIHD	Finland	serum	IS	153	1828	1.25 (0.81, 1.93)	Highest versus lowest fourth	0.39	1.09 (0.92, 1.30)	++
Wu 2014	CHS	US	plasma PL	IS	362	2792	1.09 (0.76, 1.55)	Highest versus lowest fifth	0.36	1.03 (0.91, 1.17)	++++*
Yamagishi 2013	ARIC	US	plasma PL	IS	168	3870	1.26 (0.80, 1.99)	Highest versus lowest fourth	0.39	1.10 (0.92, 1.31)	+
Yaemsiri 2013	WHI-OS	US	serum	IS	964	1928	0.91 (0.77, 1.08)	per SD increase	1.00	0.91 (0.77, 1.08)	++
Wiberg 2006	ULSAM 50	Sweden	serum CE	IS	308	2313	1.08 (0.96, 1.22)	per SD increase	1.00	1.08 (0.96, 1.22)	++
**EDA (20:2n6)**											
Harris 2018	FHS-Offspring	US	RBC	IS	105	2500	1.16 (0.59, 2.28)	Highest versus lowest fifth	0.36	1.05 (0.83, 1.34)	+++
Yaemsiri 2013	WHI-OS	US	serum	IS	964	1928	1.02 (0.86, 1.21)	per SD increase	1.00	1.02 (0.86, 1.21)	++
**DGLA (20:3n6)**											
Harris 2018	FHS-Offspring	US	RBC	IS	105	2500	0.90 (0.42, 1.94)	Highest versus lowest fifth	0.36	0.96 (0.73, 1.27)	+++
Daneshmand 2016	KIHD	Finland	serum	IS	153	1828	1.09 (0.68, 1.76)	Highest versus lowest fourth	0.39	1.03 (0.86, 1.25)	++
Wu 2014	CHS	US	plasma PL	IS	362	2792	1.04 (0.72, 1.50)	Highest versus lowest fifth	0.36	1.01 (0.89, 1.16)	++++*
Yamagishi 2013	ARIC	US	plasma PL	IS	168	3870	1.17 (0.78, 1.78)	Highest versus lowest fourth	0.39	1.06 (0.90, 1.25)	+
Yaemsiri 2013	WHI-OS	US	serum	IS	964	1928	0.94 (0.79, 1.12)	per SD increase	1.00	0.94 (0.79, 1.12)	++
Wiberg 2006	ULSAM 50	Sweden	serum CE	IS	308	2313	1.12 (1.00, 1.25)	per SD increase	1.00	1.12 (1.00, 1.25)	++
**AA (20:4n6)**											
Marklund 2019	60YO	Sweden	serum CE	IS	155	4150	1.08 (0.70, 1.65)	per interquintile range (10-90th)	0.39	1.03 (0.87, 1.22)	++++*
Marklund 2019	AGES-Reykjavik	Iceland	plasma PL	IS	123	1195	0.67 (0.39, 1.15)	per interquintile range (10-90th)	0.39	0.86 (0.69, 1.06)	++++*
Marklund 2019	ARIC	US	plasma PL	IS	188	3749	0.95 (0.67, 1.35)	per interquintile range (10-90th)	0.39	0.98 (0.86, 1.12)	++++*
Marklund 2019	CHS	US	plasma PL	IS	408	2907	1.12 (0.82, 1.54)	per interquintile range (10-90th)	0.39	1.05 (0.92, 1.18)	++++*
Marklund 2019	HS	Japan	serum	IS	97	3103	0.80 (0.43, 1.51)	per interquintile range (10-90th)	0.39	0.92 (0.72, 1.17)	++++*

Marklund 2019	MCCS	Australia	plasma PL	fatal IS	44	6265	1.22 (0.52, 2.84)	per interquintile range (10-90th)	0.39	1.08 (0.78, 1.50)	++++*
Marklund 2019	MESA	US	plasma PL	IS	53	2722	0.34 (0.11, 1.02)	per interquintile range (10-90th)	0.39	0.66 (0.43, 1.01)	++++*
Marklund 2019	NSHDS III	Sweden	plasma PL	IS	85	317	0.58 (0.21, 1.60)	per interquintile range (10-90th)	0.39	0.81 (0.54, 1.20)	++++*
Marklund 2019	PIVUS	Sweden	serum PL	IS	37	835	1.00 (0.37, 2.76)	per interquintile range (10-90th)	0.39	1.00 (0.68, 1.48)	++++*
Marklund 2019	ULSAM 50	Sweden	serum CE	IS	313	1992	1.07 (0.79, 1.47)	per interquintile range (10-90th)	0.39	1.03 (0.91, 1.16)	++++*
Marklund 2019	ULSAM 70	Sweden	AT	IS	115	763	0.67 (0.38, 1.16)	per interquintile range (10-90th)	0.39	0.86 (0.69, 1.06)	++++*
Marklund 2019	WHI-MS	US	RBC	IS	295	5263	1.36 (0.87, 2.13)	per interquintile range (10-90th)	0.39	1.13 (0.95, 1.34)	++++*
Satizabal 2018	3C Study	France	plasma	IS	27	1055	0.72 (0.49, 1.06)	per SD increase	1.00	0.72 (0.49, 1.06)	++
Satizabal 2018	FHS	US	plasma PL	IS	75	906	1.04 (0.82, 1.33)	per SD increase	1.00	1.04 (0.82, 1.33)	++
Harris 2018	FHS-Offspring	US	RBC	IS	105	2500	1.42 (0.73, 2.77)	Highest versus lowest fifth	0.36	1.13 (0.89, 1.44)	+++
Daneshmand 2016	KIHD	Finland	serum	IS	153	1828	0.96 (0.61, 1.53)	Highest versus lowest fourth	0.39	0.98 (0.82, 1.18)	++
Yaemsiri 2013	WHI-OS	US	serum	IS	964	1928	0.82 (0.67, 0.99)	per SD increase	1.00	0.82 (0.67, 0.99)	++
**DTA (22:4n6)**											
Harris 2018	FHS-Offspring	US	RBC	IS	105	2500	2.11 (0.97, 4.59)	Highest versus lowest fifth	0.36	1.31 (0.99, 1.72)	+++
Yaemsiri 2013	WHI-OS	US	serum	IS	964	1928	0.91 (0.69, 1.22)	per SD increase	1.00	0.91 (0.69, 1.22)	++
**DPA (22:5n6)**											
Harris 2018	FHS-Offspring	US	RBC	IS	105	2500	2.01 (0.93, 4.35)	Highest versus lowest fifth	0.36	1.28 (0.97, 1.69)	+++
Yaemsiri 2013	WHI-OS	US	serum	IS	964	1928	0.86 (0.72, 1.04)	per SD increase	1.00	0.86 (0.72, 1.04)	++
**trans-16:1**											
Yakoob 2014	HPFS	US	plasma	IS	80	160	0.52 (0.10, 2.71)	Highest versus lowest fourth	0.39	0.77 (0.40, 1.48)	++++*
Yakoob 2014	NHS	US	plasma	IS	371	742	1.01 (0.55, 1.84)	Highest versus lowest fourth	0.39	1.00 (0.79, 1.27)	++++*
Yaemsiri 2013	WHI-OS	US	serum	IS	964	1928	1.02 (0.86, 1.22)	per SD increase	1.00	1.02 (0.86, 1.22)	++
**trans-18:1**											
Yaemsiri 2013	WHI-OS	US	serum	IS	964	1928	1.13 (0.95, 1.34)	per SD increase	1.00	1.13 (0.95, 1.34)	++
**trans-18:2**											
Yaemsiri 2013	WHI-OS	US	serum	IS	964	1928	1.34 (1.00, 1.79)	per SD increase	1.00	1.34 (1.00, 1.79)	++

RR: Relative risk; SD: Standard deviation. See Supplementary eTable 17. footnote for other abbreviations.
Adjustment: +, adjusted for sex, age, and/or other demographic information; ++, adjusted for preceding plus any non-lipid conventional risk factors; +++, adjusted for preceding plus lipids; ++++, adjusted for preceding plus other circulating fatty acids (* not included lipids).

Supplementary eTable 21. The evidence for CHD and stroke, summarized from the most comprehensive meta-analyses of randomized controlled trials on fatty acids supplementation

Author, year	Databases	Fatty acids of interest	Outcome of interest	N of participants (trials)	Relative effect (95% CI)	Certainty of evidence (GRADE)*	Comments
Abdelhamid, 2020[151]	CENTRAL, MEDLINE, Embase (February 2019); ClinicalTrials.gov, WHO ICTRP (August 2019)	***n3-PUFA:*** long-chain n3-PUFA	CHD	134,116 (32 RCTs)	0.91 (0.85 to 0.97)	⊕⊕⊖⊖ Low	√ a small protective effect in the main analysis and some sensitivity analyses;<br>√ limited to trials at low risk of compliance problems or larger trials: RR ↓;<br>⊗ limited to trials at low summary risk of bias: closer to no effect (RR 1.0) (⊖);<br>⊗ suggestion of a dose response in meta-regression was lost when REDUCE-IT was omitted (⊖);<br>∴ ***Increasing long-chain n3 fat intake may slightly reduce the risk of CHD*** (NNTB 167, 95% CI 100 to 500; NNTB 200 for primary prevention; NNTB 143 for secondary prevention).
			Stoke	138,888 (31 RCTs)	1.02 (0.94 to 1.12)	⊕⊕⊕⊖ Moderate	√ effect size consistently suggested little or no effect for all sensitivity analyses;<br>√ very large numbers of participants took part in long-term RCTs with consistent results;<br>⊗ but the 95% CI do not exclude important harms (⊖);<br>∴ ***Long-chain n3 fat intake probably makes little or no difference to risk of stroke.***
		n3-PUFA: ALA	CHD	19,061 (4 RCTs)	1.00 (0.82 to 1.22)	⊕⊕⊖⊖ Low	√ little or no effect in the main analyses and some sensitivity analyses;<br>⊗ limited to RCTs at low summary risk of bias: risk reduction (9%) though non-significant (⊖);<br>⊗ the 95% CI do not exclude important benefits or harms (⊖);<br>***∴ ALA intake may make little or no difference to CHD.***
			Stoke	19,327 (5 RCTs)	1.15 (0.66 to 2.01)	⊕⊖⊖⊖ Very low	⊗ inconsistent effect across the main analysis and sensitivity analyses (⊖⊖);<br>⊗ only 49 participants experienced strokes in the included trials (⊖);<br>∴ The effect of ALA intake on stroke is ***unclear*** as the evidence is of very low certainty.
Hooper, 2020[152]	CENTRAL, MEDLINE, Embase (15 October 2019); Clinicaltrials.gov, WHO ICTRP (17 October 2019)	***SFA***	CHD	53,199 (11 RCTs)	0.83 (0.68 to 1.01)	⊕⊖⊖⊖ Very low	⊗ limited to trials at low summary risk of bias: moved the RR slightly towards 1.0 (⊖);<br>⊗ heterogeneity was high (⊖);<br>⊗ the 95% CI includes both no effect and a benefit (⊖);<br>∴ The effect of reducing saturated fat on CHD is ***unclear*** as the evidence is of very low quality.
			Stoke	50,952 (7 RCTs)	0.92 (0.68 to 1.25)	⊕⊖⊖⊖ Very Low	⊗ limited to trials at low summary risk of bias: moved the RR slightly away from 1.0 (⊖);<br>⊗ the 95% CI includes both important benefits and important harms (⊖⊖);<br>∴ The effect of reducing saturated fat on stroke is ***unclear*** as the evidence is of very low quality.
Hooper, 2018[153]	CENTRAL, MEDLINE, Embase (May 2017); clinicaltrials.gov, WHO ICTRP to (September 2016)	***n6-PUFA***	CHD	3997 (7 RCTs)	0.88 (0.66 to 1.17)	⊕⊖⊖⊖ Very low	⊗ one study at low summary risk of bias as many of the trials are older and detailed information on the conduct of trials can be scarce (⊖);<br>⊗ heterogeneity was high (⊖);<br>⊗ the 95% CI do not exclude important benefits or harms (⊖);<br>∴ The effect of increasing omega-6 fats on CHD is ***unclear*** as the evidence is of very low quality.
			Stoke	3730 (4 RCTs)	1.36 (0.45 to 4.11)	⊕⊖⊖⊖ Very low	⊗ one study at low summary risk of bias as many of the trials are older and detailed information on the conduct of trials can be scarce (⊖);<br>⊗ the 95% CI included large benefits (RR < 0.5) and large harms (RR > 2.0) (⊖⊖);<br>∴ The effect of increasing omega-6 fats on stroke is ***unclear*** as the evidence is of very low quality.
Abdelhamid, 2018[154]	CENTRAL, MEDLINE, Embase (April 2017); clinicaltrials.gov, WHO ICTRP (September 2016)	***PUFA***	CHD	10,076 (15 RCTs)	0.87 (0.72 to 1.06)	⊕⊕⊕⊖ Moderate	√ a small protective effect in the some sensitivity analyses (e.g. fixed effect RR 0.90 [0.82, 0.99]);<br>⊗ but some sensitivity analyses differed and the 95% CI do not exclude harms (⊖);<br>∴ ***Increasing PUFA intake may slightly reduce risk of CHD*** (from 14.2% to 12.3% in the study population, NNT = 53)
			Stoke	14,742 (11 RCTs)	0.91 (0.58 to 1.44)	⊕⊕⊖⊖ Low	√ results of the most heavily weighted trials are consistent with the main results;<br>⊗ some sensitivity analyses suggested benefit while some suggested harm or little effect, and with only 166 participants experiencing a stroke, the 95% CI did not exclude important harm (⊖⊖);<br>∴ ***Increasing PUFA intake may slightly reduce risk of stroke*** (from 1.2% to 1.1% in the study population, NNT= 1000).

ALA: Alpha-linolenic acid; CENTRAL: the Cochrane Central Register of Controlled Trials; CHD: Coronary heart disease; CI: Confidence interval; ICTRP: International Clinical Trials Registry Platform; PUFA: Polyunsaturated fatty acid; RR: Risk ratio; RCT: randomized controlled trial; SFA: Saturated fatty acid.

The most comprehensive one with larger numbers of included RCTs were selected when multiple systematic reviews with meta-analysis were identified for the same FA intake and cardiovascular outcomes of interest.

* Based on Risk of bias; Inconsistency; Indirectness; Imprecision; and Publication bias.

# eResults: Figures

Supplementary eFigure 3. Heatmap of correlations between plasma phospholipid FAs (%) and self-reported food intake (g/day) in EPIC-CVD study

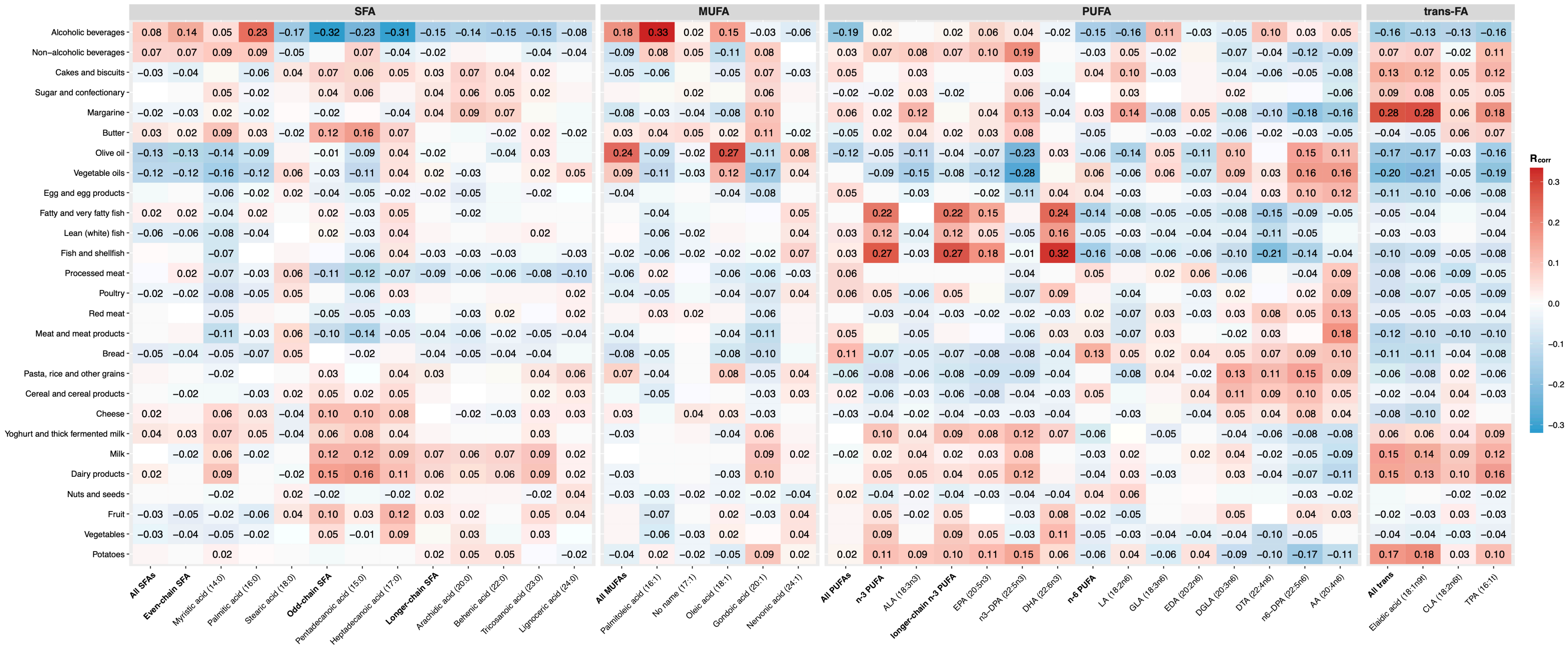

FA: Fatty acid; MUFA: Monounsaturated fatty acid; PUFA: Polyunsaturated fatty acid; SFA: Saturated fatty acid. Abbreviations for individual metabolites refer to Supplementary eTable 4.
Semipartial correlation coefficients with p-value <0.05, adjusted for batch, sex, age, and total energy intake. Analyses were conducted restricted to the sub-cohort of EPIC-CVD study (n=15,838).

Supplementary eFigure 4A-G. Hazard ratios per 1-SD higher FAs for CHD and stroke by explored baseline characteristics, estimated from EPIC-CVD, UKB and INTERVAL studies.

A. SFA

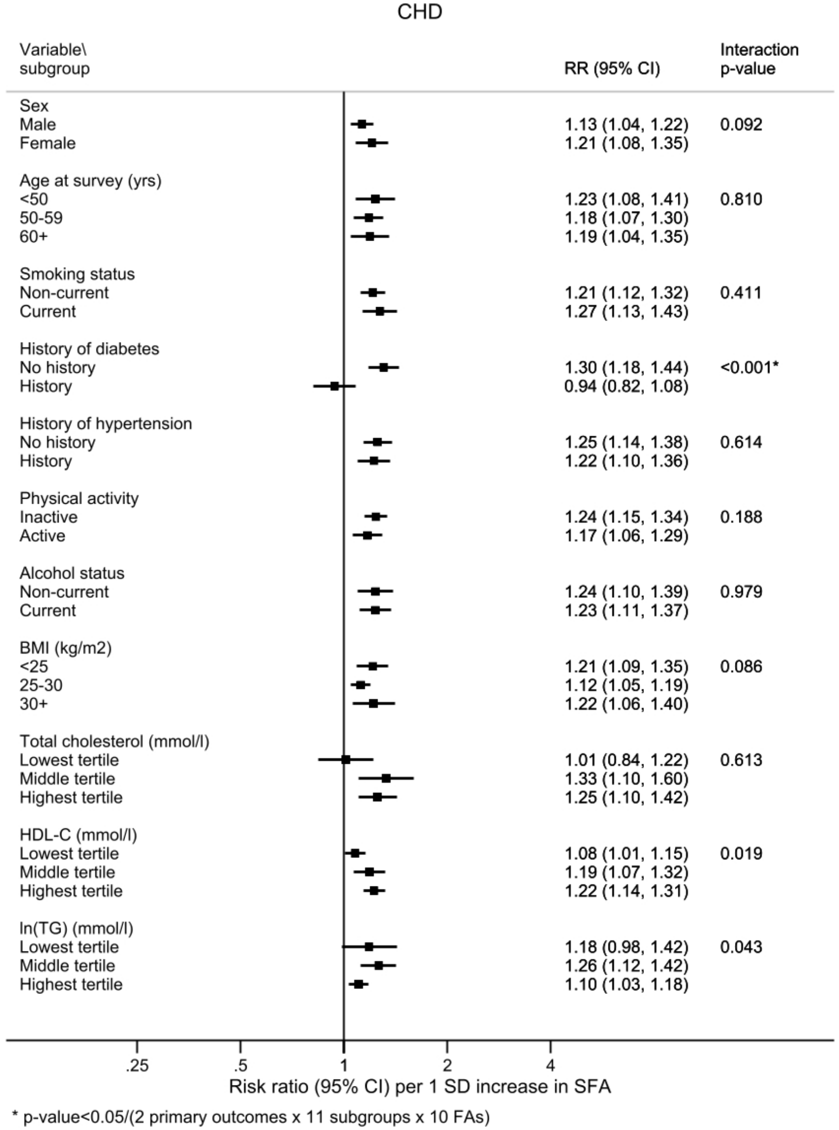

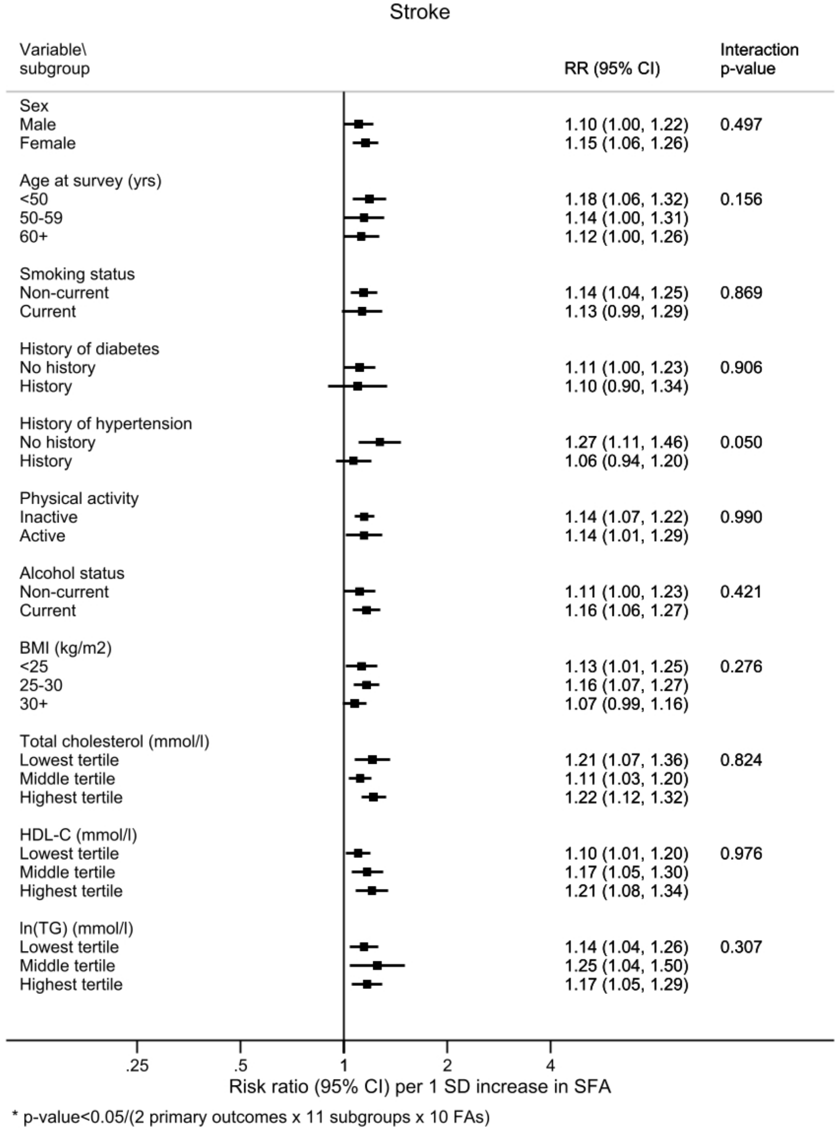

CHD: Coronary heart disease; SFA: Saturated fatty acids; SD: Standard deviation. Data are pooled hazard ratios (95%CI) per 1-SD higher fatty acids, adjusted for batch (EPIC-CVD only), age, smoking status, history of diabetes, history of hypertension, and physical activity, and stratified by center (EPIC-CVD only) and sex.

B. MUFA

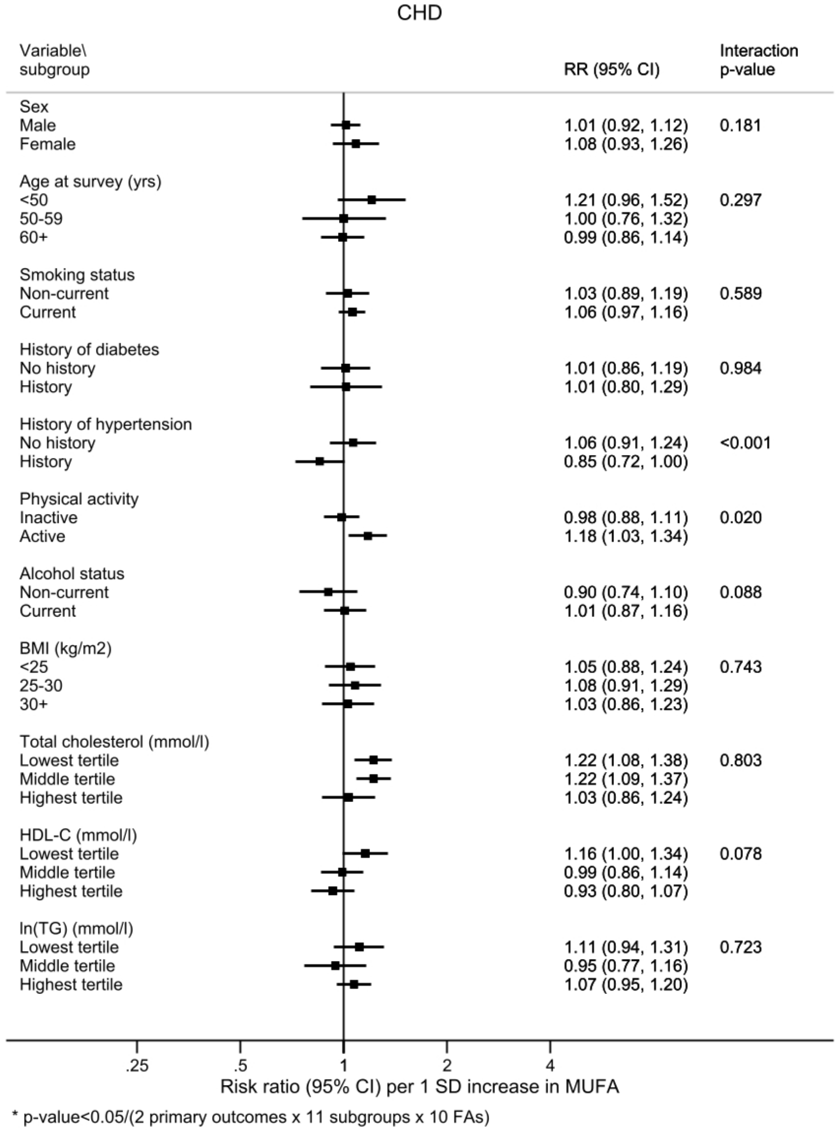

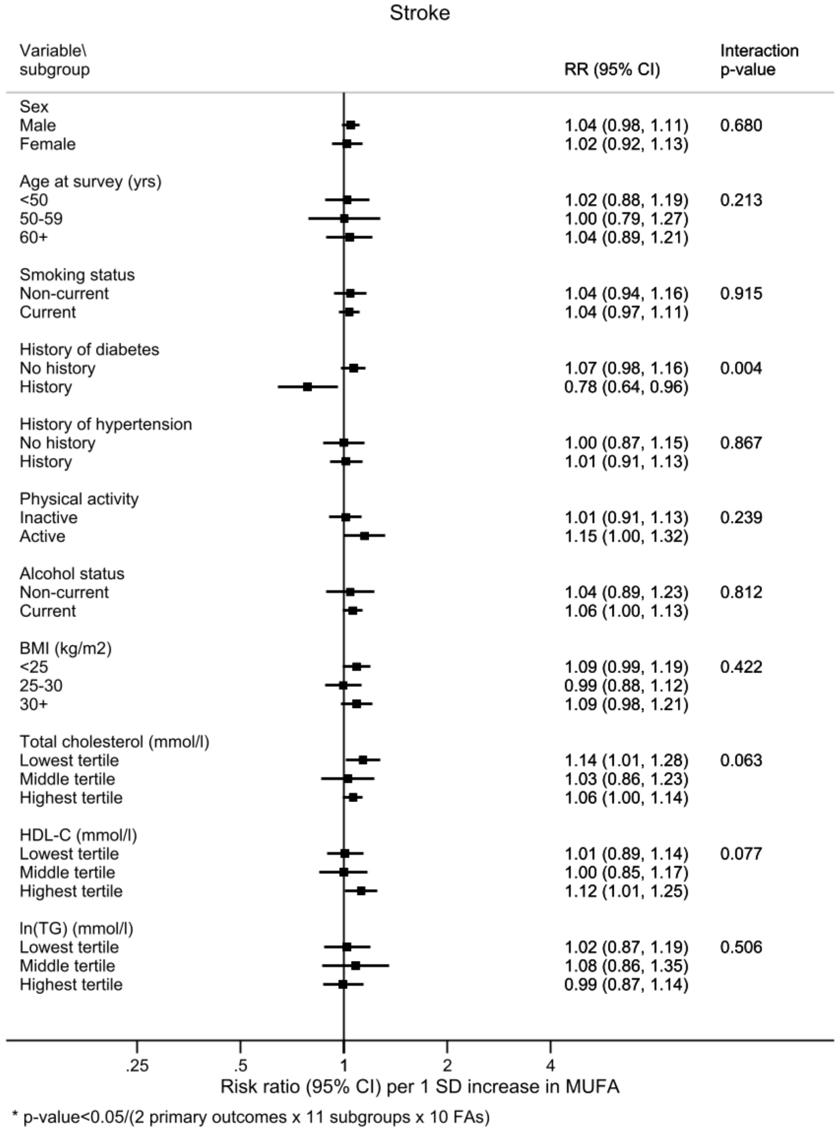

CHD: Coronary heart disease; MUFA: Monounsaturated fatty acids; SD: Standard deviation. Data are pooled hazard ratios (95%CI) per 1-SD higher fatty acids, adjusted for batch (EPIC-CVD only), age, smoking status, history of diabetes, history of hypertension, and physical activity, and stratified by center (EPIC-CVD only) and sex.

C. PUFA

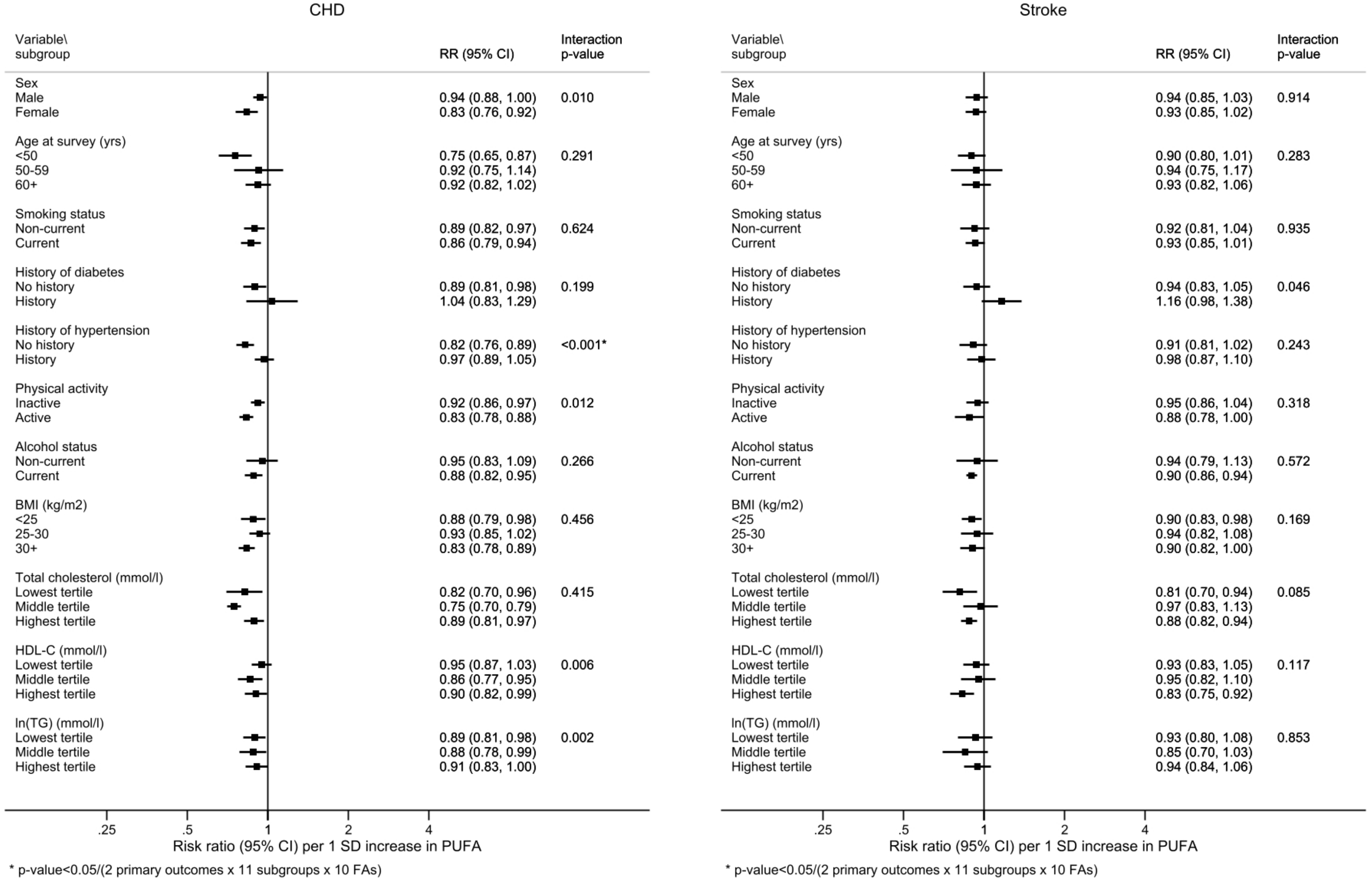

CHD: Coronary heart disease; PUFA: Polyunsaturated fatty acids; SD: Standard deviation. Data are pooled hazard ratios (95%CI) per 1-SD higher fatty acids, adjusted for batch (EPIC-CVD only), age, smoking status, history of diabetes, history of hypertension, and physical activity, and stratified by center (EPIC-CVD only) and sex.

D. n-6 PUFA

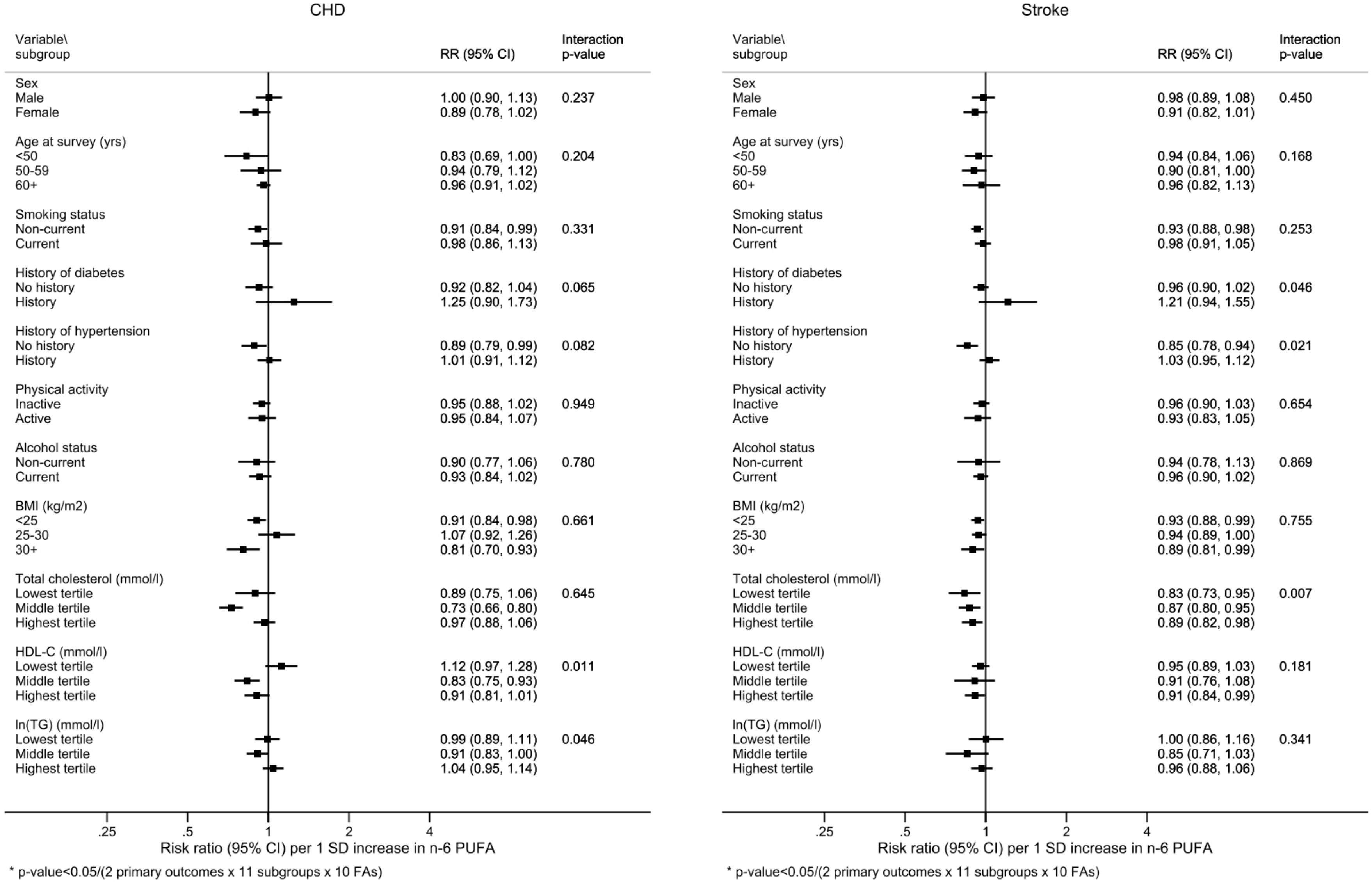

CHD: Coronary heart disease; PUFA: Polyunsaturated fatty acids; SD: Standard deviation. Data are pooled hazard ratios (95%CI) per 1-SD higher fatty acids, adjusted for batch (EPIC-CVD only), age, smoking status, history of diabetes, history of hypertension, and physical activity, and stratified by center (EPIC-CVD only) and sex.

E. n-3 PUFA

CHD

Variable\ subgroup	RR (95% CI)	Interaction p-value
Sex		
Male	0.89 (0.80, 0.99)	0.875
Female	0.88 (0.78, 0.99)	
Age at survey (yrs)		
<50	0.82 (0.62, 1.07)	0.843
50-59	0.86 (0.76, 0.98)	
60+	0.93 (0.86, 1.02)	
Smoking status		
Non-current	0.94 (0.86, 1.04)	0.021
Current	0.76 (0.64, 0.90)	
History of diabetes		
No history	0.96 (0.83, 1.10)	0.111
History	0.74 (0.58, 0.94)	
History of hypertension		
No history	0.94 (0.83, 1.06)	0.669
History	0.91 (0.81, 1.02)	
Physical activity		
Inactive	0.98 (0.86, 1.11)	0.039
Active	0.81 (0.74, 0.90)	
Alcohol status		
Non-current	0.98 (0.81, 1.19)	0.353
Current	0.89 (0.82, 0.97)	
BMI (kg/m2)		
<25	0.94 (0.85, 1.03)	0.929
25-30	0.85 (0.74, 0.97)	
30+	1.09 (0.86, 1.39)	
Total cholesterol (mmol/l)		
Lowest tertile	0.77 (0.65, 0.92)	0.019
Middle tertile	1.04 (0.87, 1.25)	
Highest tertile	0.88 (0.79, 0.98)	
HDL-C (mmol/l)		
Lowest tertile	0.82 (0.71, 0.93)	0.351
Middle tertile	1.07 (0.95, 1.20)	
Highest tertile	0.95 (0.87, 1.04)	
ln(TG) (mmol/l)		
Lowest tertile	0.92 (0.84, 1.01)	0.085
Middle tertile	1.06 (0.93, 1.20)	
Highest tertile	0.82 (0.74, 0.92)	

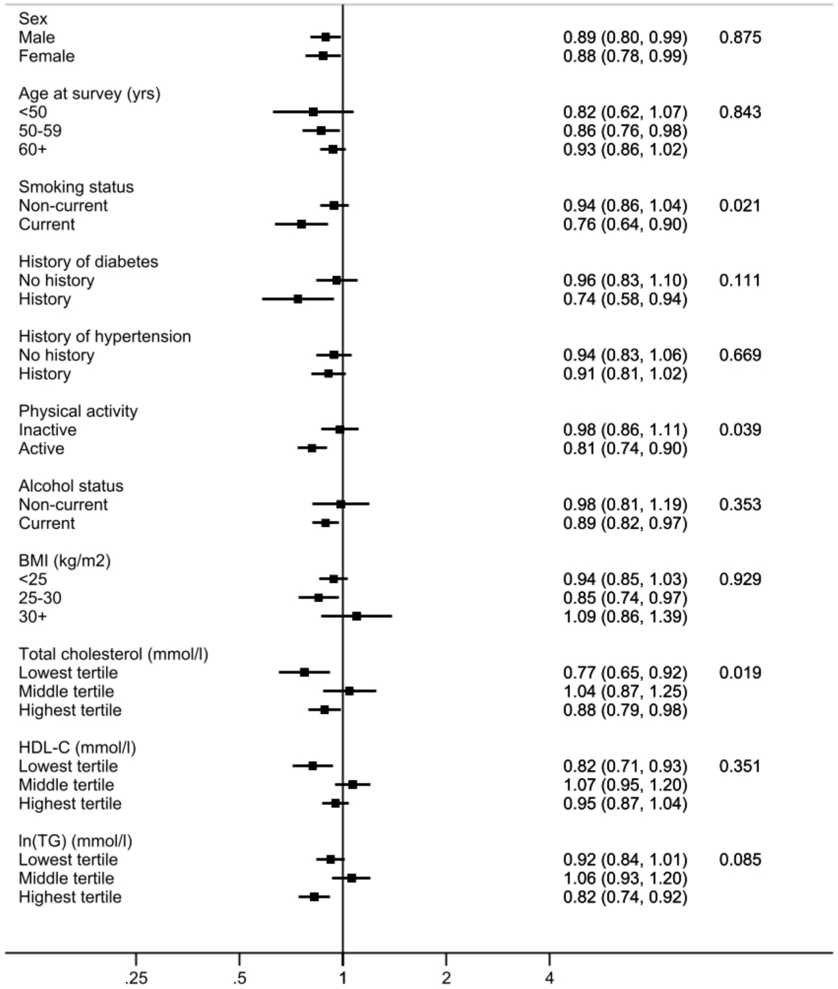

* p-value<0.05/(2 primary outcomes x 11 subgroups x 10 FAs)

Stroke

Variable\ subgroup	RR (95% CI)	Interaction p-value
Sex		
Male	0.94 (0.85, 1.03)	0.287
Female	1.03 (0.92, 1.16)	
Age at survey (yrs)		
<50	0.98 (0.87, 1.09)	0.656
50-59	1.05 (0.87, 1.28)	
60+	0.92 (0.84, 1.01)	
Smoking status		
Non-current	0.98 (0.91, 1.06)	0.182
Current	0.91 (0.85, 0.99)	
History of diabetes		
No history	0.95 (0.87, 1.03)	0.948
History	0.94 (0.77, 1.16)	
History of hypertension		
No history	1.09 (0.95, 1.24)	0.041
History	0.91 (0.82, 1.01)	
Physical activity		
Inactive	0.96 (0.83, 1.12)	0.627
Active	0.91 (0.77, 1.06)	
Alcohol status		
Non-current	1.00 (0.87, 1.15)	0.321
Current	0.92 (0.87, 0.98)	
BMI (kg/m2)		
<25	0.97 (0.89, 1.05)	0.954
25-30	0.96 (0.86, 1.07)	
30+	1.01 (0.88, 1.17)	
Total cholesterol (mmol/l)		
Lowest tertile	1.00 (0.89, 1.12)	0.469
Middle tertile	1.06 (0.90, 1.24)	
Highest tertile	1.00 (0.89, 1.14)	
HDL-C (mmol/l)		
Lowest tertile	1.04 (0.91, 1.18)	0.192
Middle tertile	1.07 (0.90, 1.27)	
Highest tertile	0.96 (0.87, 1.06)	
ln(TG) (mmol/l)		
Lowest tertile	0.96 (0.87, 1.05)	0.542
Middle tertile	1.07 (0.89, 1.28)	
Highest tertile	0.94 (0.87, 1.03)	

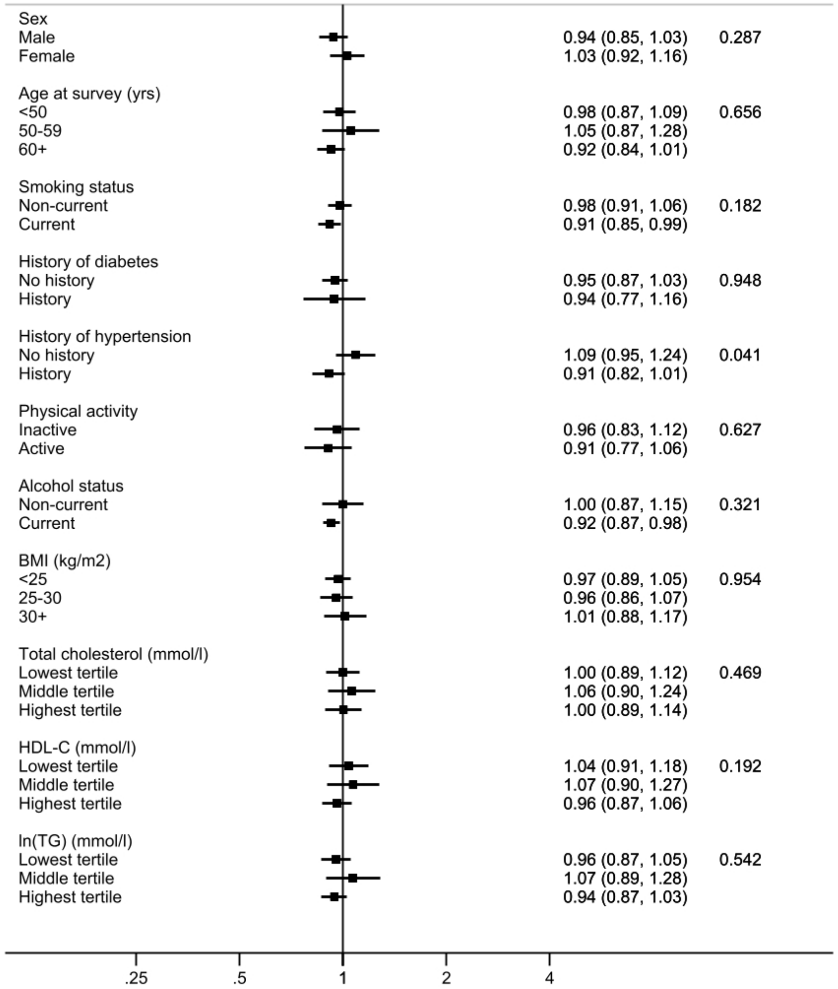

* p-value<0.05/(2 primary outcomes x 11 subgroups x 10 FAs)

CHD: Coronary heart disease; PUFA: Polyunsaturated fatty acids; SD: Standard deviation. Data are pooled hazard ratios (95%CI) per 1-SD higher fatty acids, adjusted for batch (EPIC-CVD only), age, smoking status, history of diabetes, history of hypertension, and physical activity, and stratified by center (EPIC-CVD only) and sex.

F. DHA

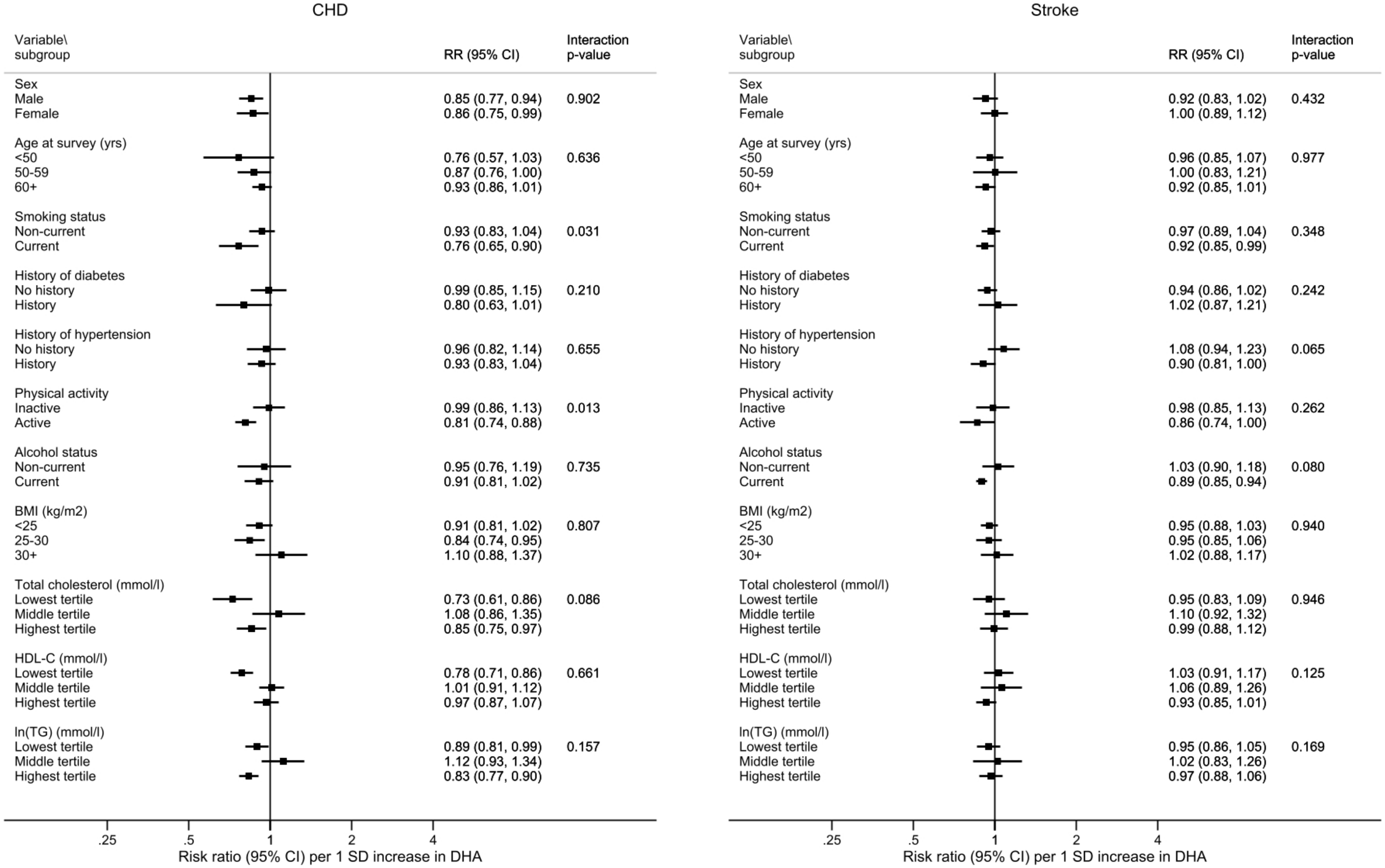

CHD

Variable\ subgroup	RR (95% CI)	Interaction p-value
Sex		
Male	0.85 (0.77, 0.94)	0.902
Female	0.86 (0.75, 0.99)	
Age at survey (yrs)		
<50	0.76 (0.57, 1.03)	0.636
50-59	0.87 (0.76, 1.00)	
60+	0.93 (0.86, 1.01)	
Smoking status		
Non-current	0.93 (0.83, 1.04)	0.031
Current	0.76 (0.65, 0.90)	
History of diabetes		
No history	0.99 (0.85, 1.15)	0.210
History	0.80 (0.63, 1.01)	
History of hypertension		
No history	0.96 (0.82, 1.14)	0.655
History	0.93 (0.83, 1.04)	
Physical activity		
Inactive	0.99 (0.86, 1.13)	0.013
Active	0.81 (0.74, 0.88)	
Alcohol status		
Non-current	0.95 (0.76, 1.19)	0.735
Current	0.91 (0.81, 1.02)	
BMI (kg/m2)		
<25	0.91 (0.81, 1.02)	0.807
25-30	0.84 (0.74, 0.95)	
30+	1.10 (0.88, 1.37)	
Total cholesterol (mmol/l)		
Lowest tertile	0.73 (0.61, 0.86)	0.086
Middle tertile	1.08 (0.86, 1.35)	
Highest tertile	0.85 (0.75, 0.97)	
HDL-C (mmol/l)		
Lowest tertile	0.78 (0.71, 0.86)	0.661
Middle tertile	1.01 (0.91, 1.12)	
Highest tertile	0.97 (0.87, 1.07)	
ln(TG) (mmol/l)		
Lowest tertile	0.89 (0.81, 0.99)	0.157
Middle tertile	1.12 (0.93, 1.34)	
Highest tertile	0.83 (0.77, 0.90)	

* p-value<0.05/(2 primary outcomes x 11 subgroups x 10 FAs)

Stroke

Variable\ subgroup	RR (95% CI)	Interaction p-value
Sex		
Male	0.92 (0.83, 1.02)	0.432
Female	1.00 (0.89, 1.12)	
Age at survey (yrs)		
<50	0.96 (0.85, 1.07)	0.977
50-59	1.00 (0.83, 1.21)	
60+	0.92 (0.85, 1.01)	
Smoking status		
Non-current	0.97 (0.89, 1.04)	0.348
Current	0.92 (0.85, 0.99)	
History of diabetes		
No history	0.94 (0.86, 1.02)	0.242
History	1.02 (0.87, 1.21)	
History of hypertension		
No history	1.08 (0.94, 1.23)	0.065
History	0.90 (0.81, 1.00)	
Physical activity		
Inactive	0.98 (0.85, 1.13)	0.262
Active	0.86 (0.74, 1.00)	
Alcohol status		
Non-current	1.03 (0.90, 1.18)	0.080
Current	0.89 (0.85, 0.94)	
BMI (kg/m2)		
<25	0.95 (0.88, 1.03)	0.940
25-30	0.95 (0.85, 1.06)	
30+	1.02 (0.88, 1.17)	
Total cholesterol (mmol/l)		
Lowest tertile	0.95 (0.83, 1.09)	0.946
Middle tertile	1.10 (0.92, 1.32)	
Highest tertile	0.99 (0.88, 1.12)	
HDL-C (mmol/l)		
Lowest tertile	1.03 (0.91, 1.17)	0.125
Middle tertile	1.06 (0.89, 1.26)	
Highest tertile	0.93 (0.85, 1.01)	
ln(TG) (mmol/l)		
Lowest tertile	0.95 (0.86, 1.05)	0.169
Middle tertile	1.02 (0.83, 1.26)	
Highest tertile	0.97 (0.88, 1.06)	

* p-value<0.05/(2 primary outcomes x 11 subgroups x 10 FAs)

CHD: Coronary heart disease; DHA: Docosahexaenoic acid; SD: Standard deviation. Data are pooled hazard ratios (95%CI) per 1-SD higher fatty acids, adjusted for batch (EPIC-CVD only), age, smoking status, history of diabetes, history of hypertension, and physical activity, and stratified by center (EPIC-CVD only) and sex.

G. LA

CHD

Variable\ subgroup	RR (95% CI)	Interaction p-value
Sex		
Male	0.98 (0.90, 1.07)	0.350
Female	0.91 (0.83, 1.00)	
Age at survey (yrs)		
<50	0.77 (0.65, 0.92)	0.143
50-59	0.94 (0.84, 1.05)	
60+	0.96 (0.88, 1.04)	
Smoking status		
Non-current	0.90 (0.85, 0.96)	0.794
Current	0.92 (0.80, 1.04)	
History of diabetes		
No history	0.91 (0.84, 0.98)	0.003
History	1.08 (0.95, 1.21)	
History of hypertension		
No history	0.85 (0.77, 0.94)	0.064
History	0.98 (0.90, 1.08)	
Physical activity		
Inactive	0.94 (0.87, 1.01)	0.584
Active	0.91 (0.84, 0.99)	
Alcohol status		
Non-current	0.92 (0.82, 1.03)	0.845
Current	0.90 (0.82, 0.99)	
BMI (kg/m2)		
<25	0.90 (0.85, 0.96)	0.952
25-30	0.99 (0.89, 1.09)	
30+	0.88 (0.79, 0.97)	
Total cholesterol (mmol/l)		
Lowest tertile	0.88 (0.72, 1.06)	0.484
Middle tertile	0.80 (0.72, 0.90)	
Highest tertile	0.93 (0.87, 0.99)	
HDL-C (mmol/l)		
Lowest tertile	1.04 (0.91, 1.19)	0.052
Middle tertile	0.89 (0.82, 0.97)	
Highest tertile	0.87 (0.81, 0.93)	
ln(TG) (mmol/l)		
Lowest tertile	0.91 (0.84, 0.99)	<0.001*
Middle tertile	0.85 (0.74, 0.99)	
Highest tertile	1.04 (0.95, 1.15)	

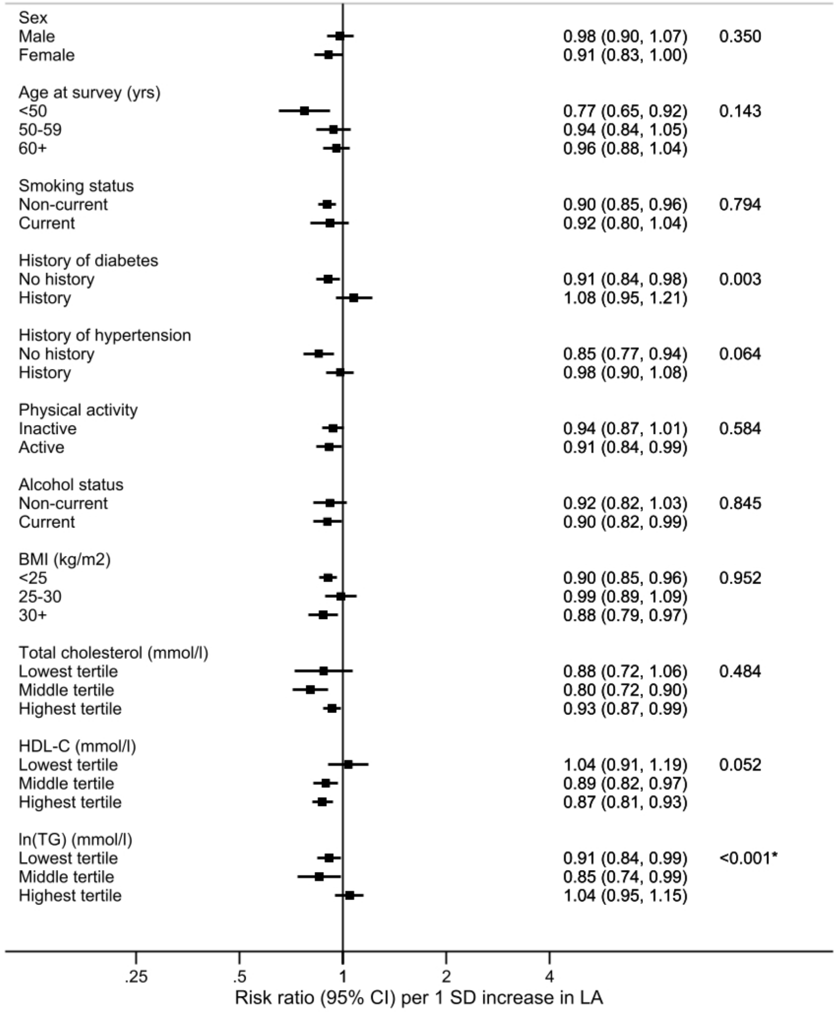

* p-value<0.05/(2 primary outcomes x 11 subgroups x 10 FAs)

Stroke

Variable\ subgroup	RR (95% CI)	Interaction p-value
Sex		
Male	0.95 (0.85, 1.07)	0.527
Female	0.90 (0.82, 0.98)	
Age at survey (yrs)		
<50	0.92 (0.80, 1.06)	0.347
50-59	0.86 (0.77, 0.96)	
60+	0.92 (0.80, 1.06)	
Smoking status		
Non-current	0.89 (0.83, 0.96)	0.808
Current	0.90 (0.82, 0.99)	
History of diabetes		
No history	0.92 (0.87, 0.97)	0.031
History	1.14 (0.94, 1.39)	
History of hypertension		
No history	0.82 (0.74, 0.91)	0.026
History	0.97 (0.89, 1.06)	
Physical activity		
Inactive	0.93 (0.86, 1.00)	0.861
Active	0.91 (0.80, 1.04)	
Alcohol status		
Non-current	0.93 (0.78, 1.10)	0.856
Current	0.91 (0.84, 0.98)	
BMI (kg/m2)		
<25	0.92 (0.87, 0.98)	0.867
25-30	0.92 (0.86, 0.99)	
30+	0.82 (0.71, 0.96)	
Total cholesterol (mmol/l)		
Lowest tertile	0.84 (0.71, 1.00)	0.003
Middle tertile	0.88 (0.81, 0.96)	
Highest tertile	0.83 (0.75, 0.92)	
HDL-C (mmol/l)		
Lowest tertile	0.92 (0.84, 1.01)	0.598
Middle tertile	0.82 (0.70, 0.96)	
Highest tertile	0.90 (0.81, 0.99)	
ln(TG) (mmol/l)		
Lowest tertile	0.98 (0.90, 1.08)	0.377
Middle tertile	0.82 (0.68, 0.97)	
Highest tertile	0.91 (0.84, 0.99)	

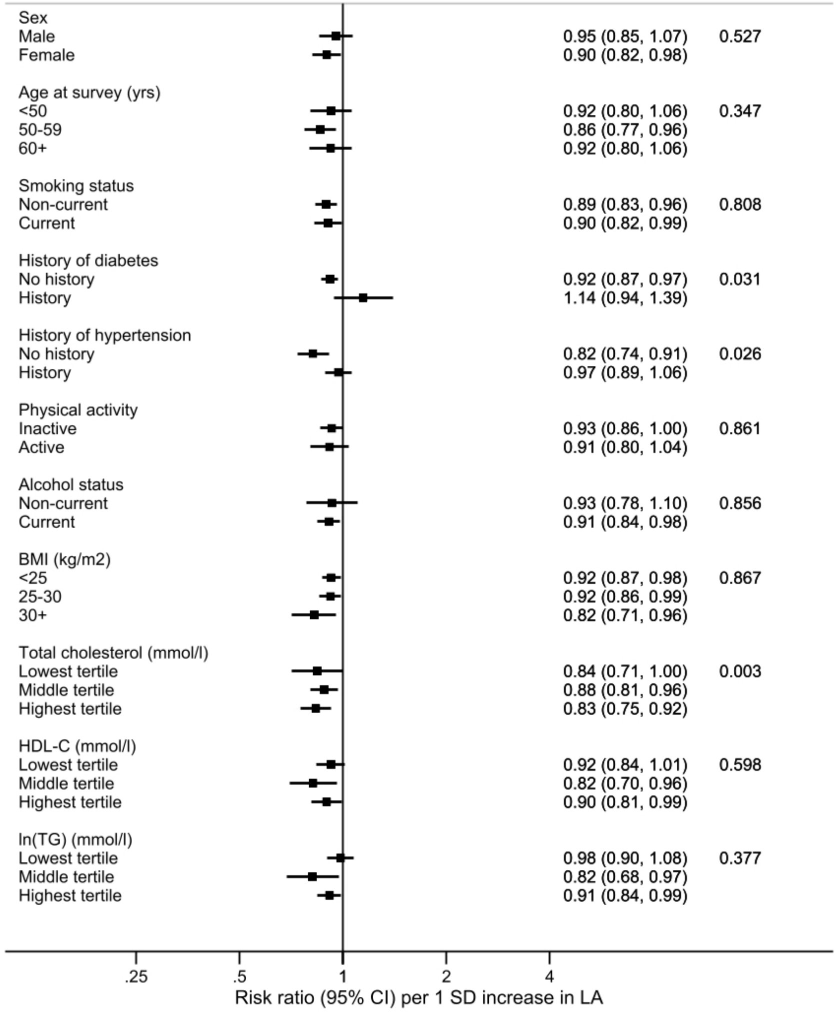

* p-value<0.05/(2 primary outcomes x 11 subgroups x 10 FAs)

CHD: Coronary heart disease; LA: Linoleic acid; SD: Standard deviation. Data are pooled hazard ratios (95%CI) per 1-SD higher fatty acids, adjusted for batch (EPIC-CVD only), age, smoking status, history of diabetes, history of hypertension, and physical activity, and stratified by center (EPIC-CVD only) and sex.

Supplementary eFigure 5A-C. Hazard ratios per 1-SD higher SFA subtypes for CHD and stroke by explored baseline characteristics, estimated from EPIC-CVD participants.

A. Even-chain SFA

CHD

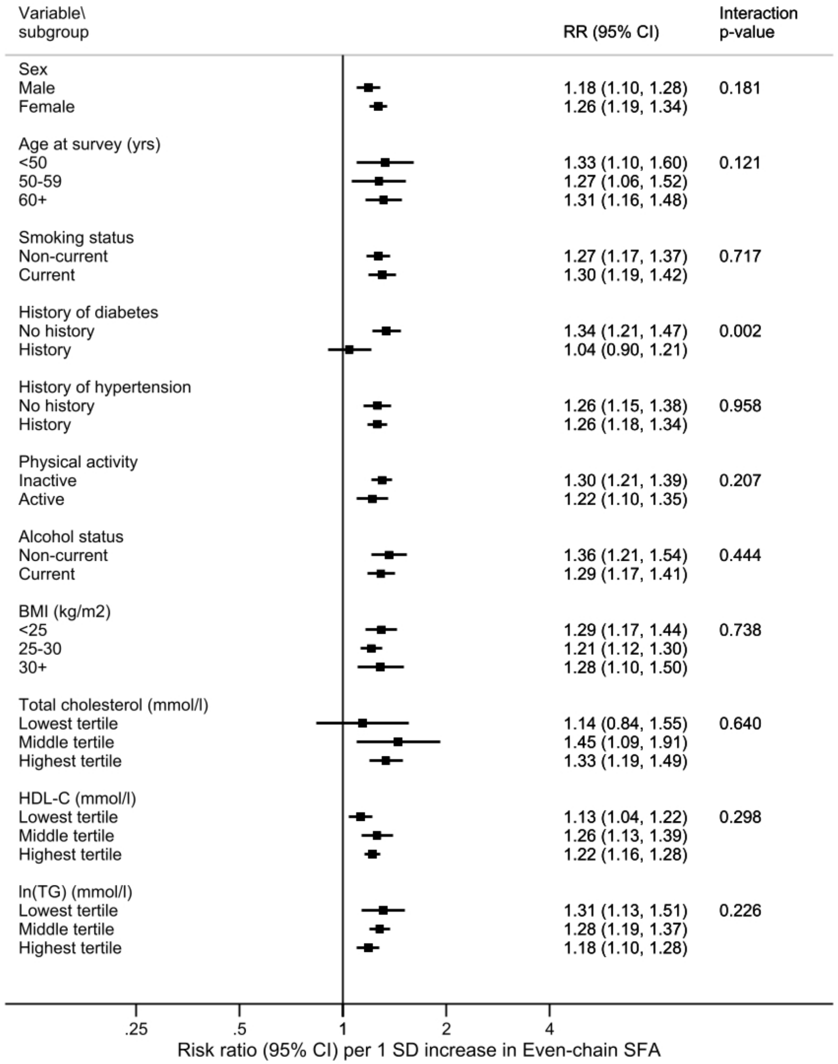

* p-value<0.05/(2 primary outcomes x 11 subgroups x 10 FAs)

Stroke

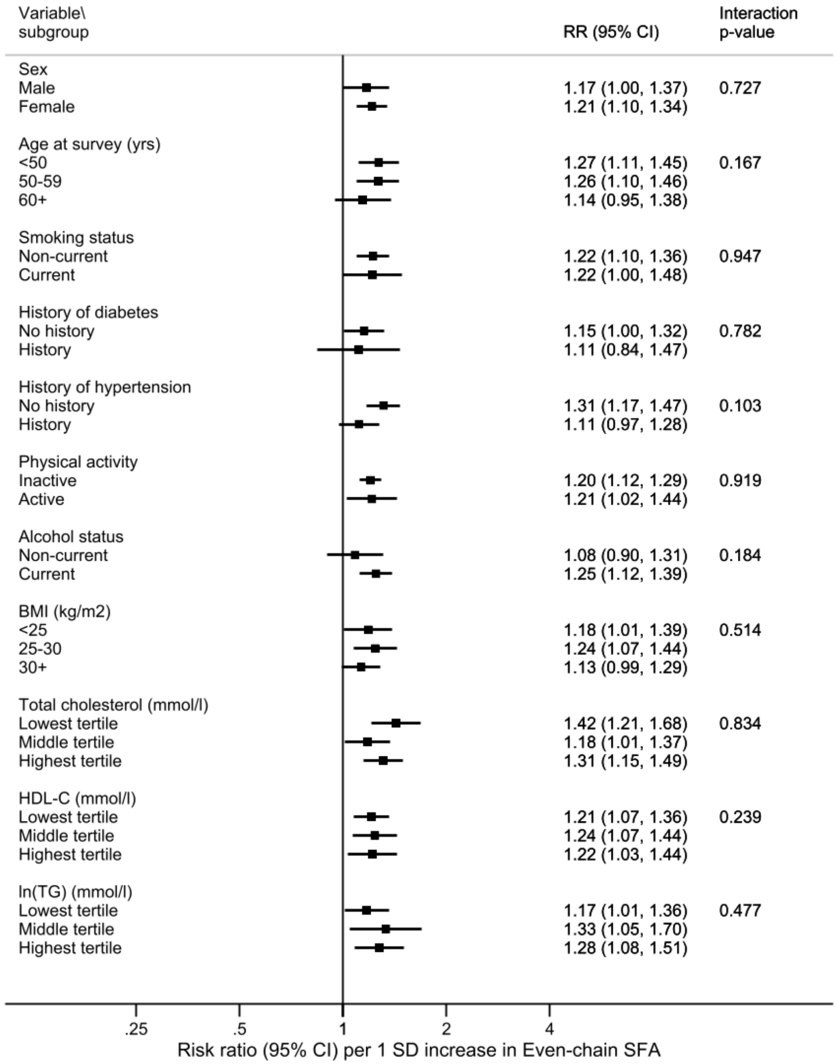

* p-value<0.05/(2 primary outcomes x 11 subgroups x 10 FAs)

CHD: Coronary heart disease; SFA: Saturated fatty acids; SD: Standard deviation. Data are pooled hazard ratios (95%CI) per 1-SD higher fatty acids, adjusted for batch, age, smoking status, history of diabetes, history of hypertension, and physical activity, and stratified by center and sex.

B. Odd-chain SFA

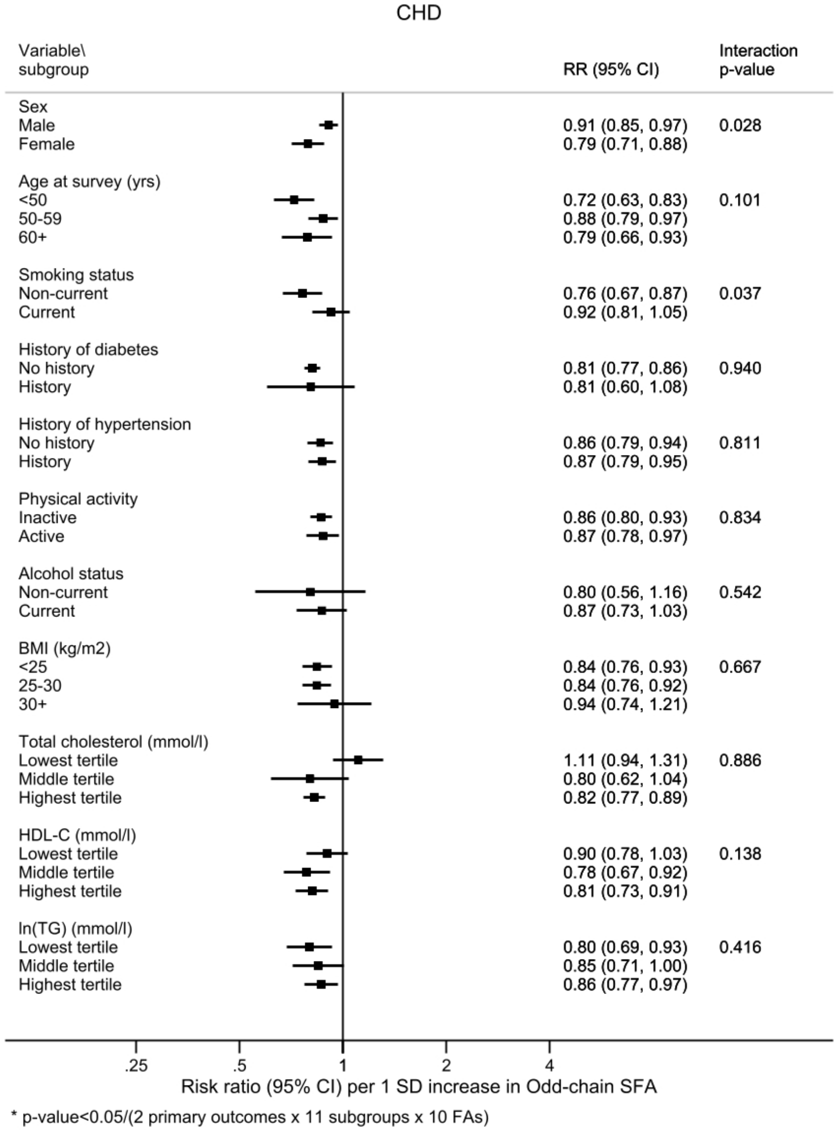

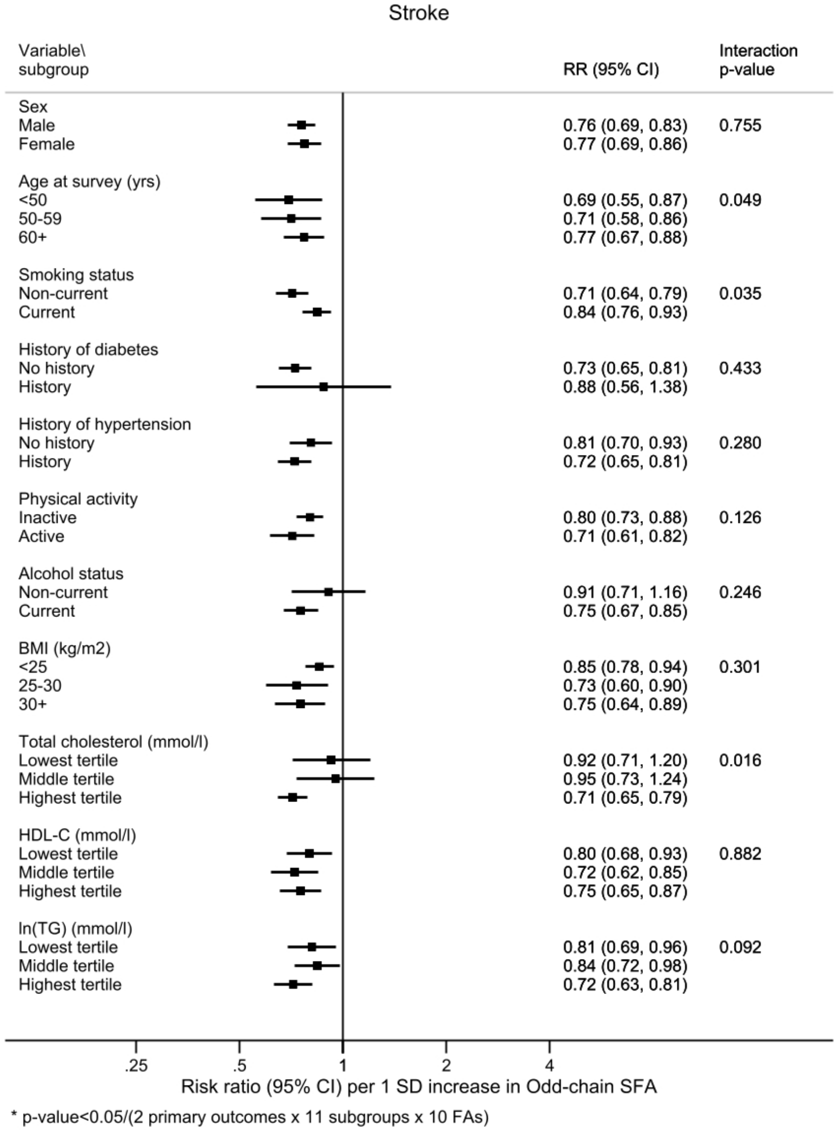

CHD: Coronary heart disease; SFA: Saturated fatty acids; SD: Standard deviation. Data are pooled hazard ratios (95%CI) per 1-SD higher fatty acids, adjusted for batch, age, smoking status, history of diabetes, history of hypertension, and physical activity, and stratified by center and sex.

C. Longer-chain SFA

CHD

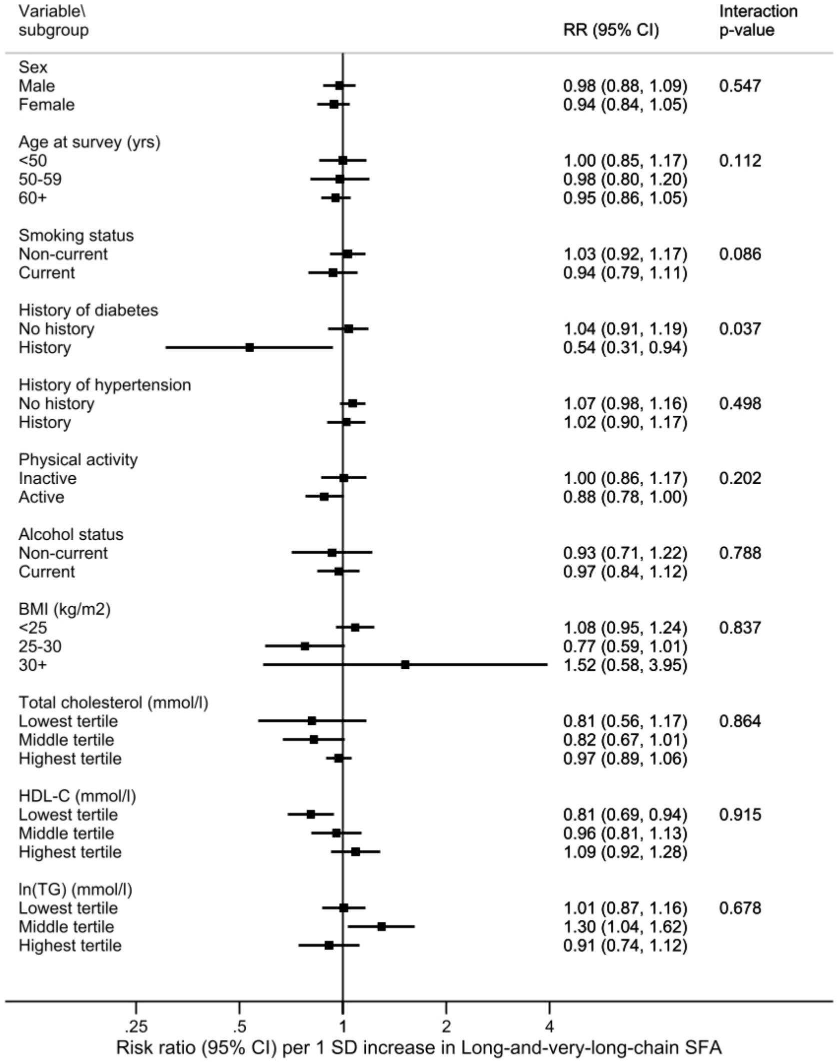

* p-value<0.05/(2 primary outcomes x 11 subgroups x 10 FAs)

Stroke

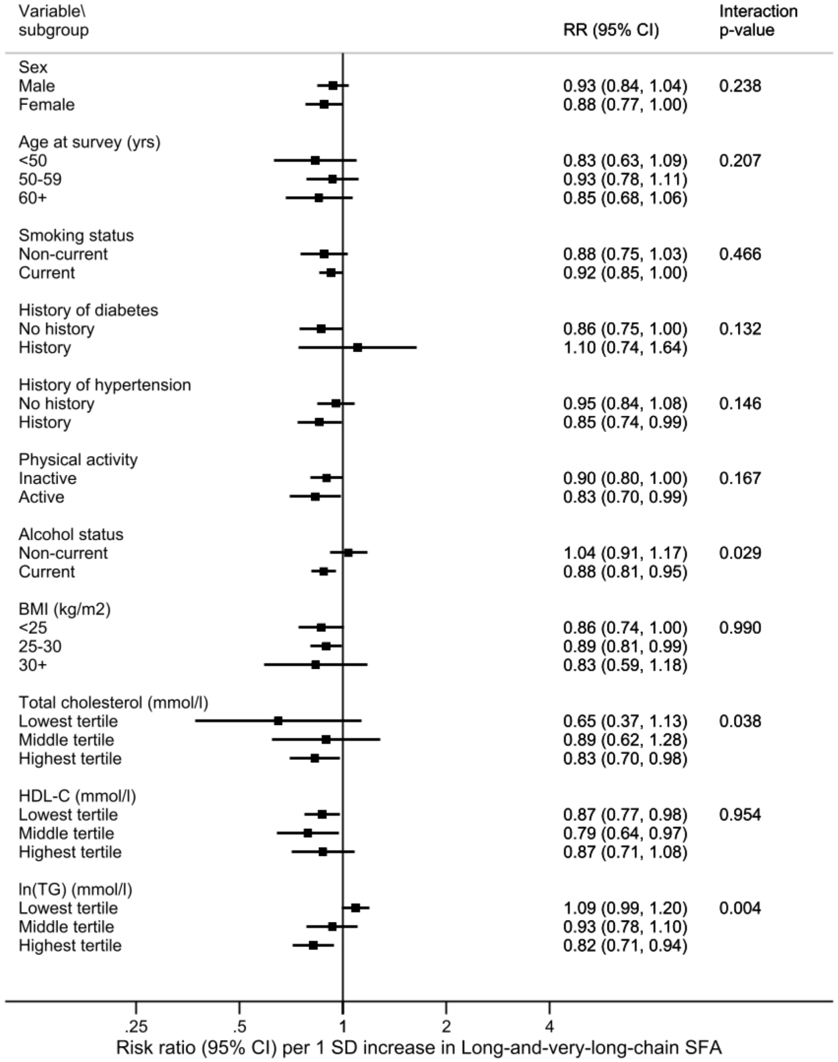

* p-value<0.05/(2 primary outcomes x 11 subgroups x 10 FAs)

CHD: Coronary heart disease; SFA: Saturated fatty acids; SD: Standard deviation. Data are pooled hazard ratios (95%CI) per 1-SD higher fatty acids, adjusted for batch, age, smoking status, history of diabetes, history of hypertension, and physical activity, and stratified by center and sex

Supplementary eFigure 6. Literature review flow diagram.

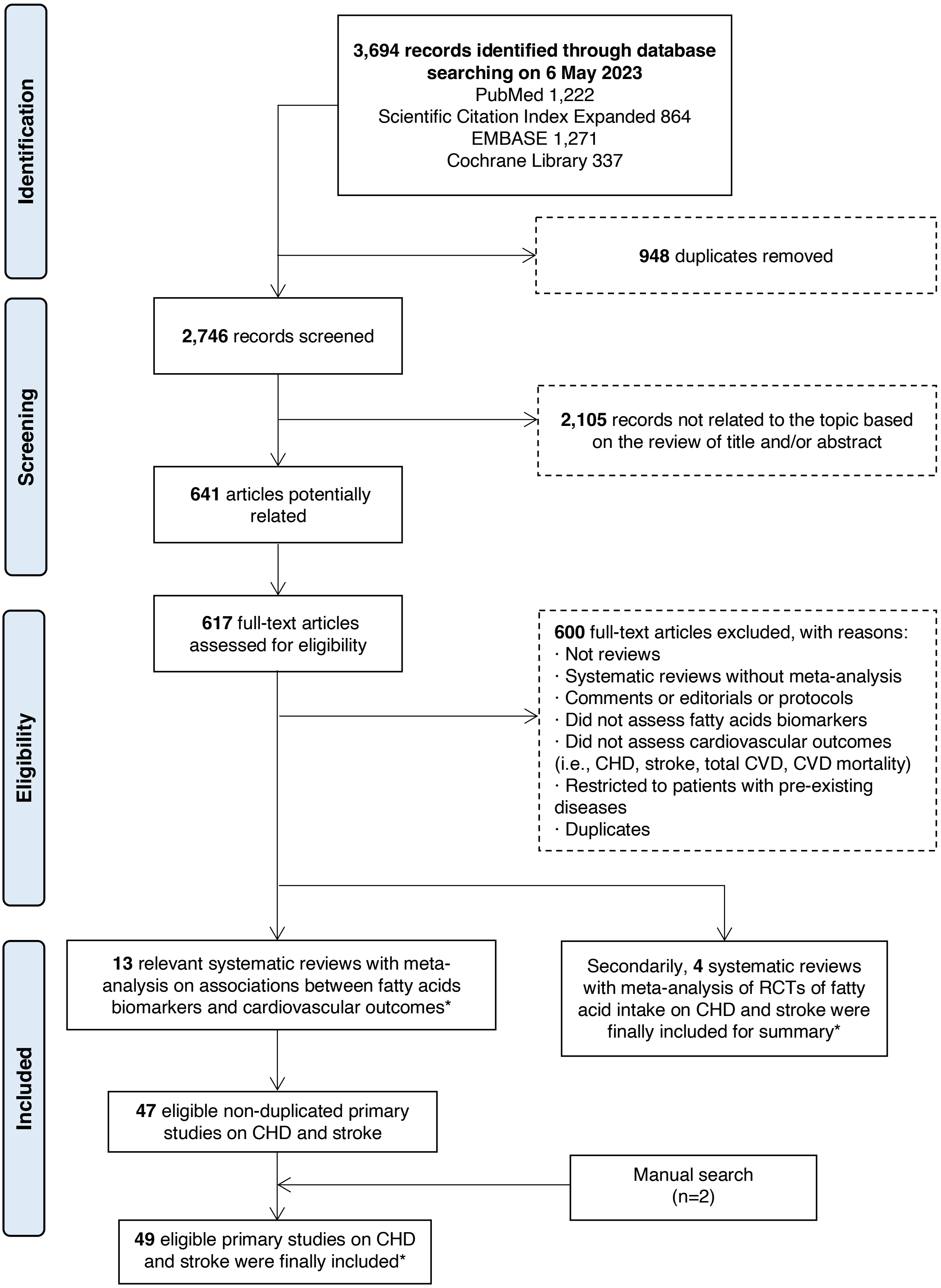

CHD: Coronary heart disease; CVD: Cardiovascular disease; RCT: Randomized controlled trial.
* More details in Supplementary eTable 16-21.

Supplementary eFigure 7. Updated meta-analysis combining results from EPIC-CVD, UKB and INTERVAL studies with published evidence for associations of fatty acid biomarkers with ischaemic stroke.

Fatty acids	N of data sources	N of cases	RR (95% CI)	I² (%)
**Total SFA**	**5**	**6,249**	**1.13 (1.01, 1.25)**	**80**
**Even-chain SFA**	**2**	**3,956**	**1.29 (1.14, 1.45)**	**55**
Myristic acid (14:0)	7	5,847	1.05 (0.96, 1.16)	52
Palmitic acid (16:0)	7	5,509	1.22 (1.14, 1.30)	21
Stearic acid (18:0)	4	5,274	1.00 (0.94, 1.06)	0
**Odd-chain SFA**				
Pentadecanoic acid (15:0)	6	5,609	0.84 (0.74, 0.96)	61
Heptadecanoic acid (17:0)	4	5,249	0.81 (0.72, 0.92)	54
**Total MUFA**	**5**	**6,249**	**1.04 (0.96, 1.12)**	**58**
Palmitoleic acid (16:1n7)	5	5,396	1.17 (1.09, 1.26)	55
Oleic acid (18:1n9)	5	5,396	1.05 (0.93, 1.18)	72
Eicosenoic acid (20:1n9)	2	4,798	0.91 (0.75, 1.11)	72
Nervonic acid (24:1n9)	2	4,798	0.92 (0.81, 1.04)	43
**Total PUFA**	**4**	**6,081**	**0.96 (0.88, 1.05)**	**66**
**Total N3 PUFA**	**6**	**6,402**	**0.99 (0.91, 1.07)**	**59**
ALA (18:3n3)	7	5,869	1.01 (0.95, 1.06)	0
**LCn-3PUFA**	**5**	**4,579**	**0.94 (0.86, 1.03)**	**39**
EPA (20:5n3)	9	6,485	1.01 (0.95, 1.08)	39
DPA (22:5n3)	7	6,009	0.90 (0.82, 0.98)	43
DHA (22:6n3)	14	8,845	0.93 (0.87, 0.98)	51
**Omega-3 index (EPA+DHA)**	**2**	**3,939**	**0.94 (0.80, 1.09)**	**58**
**Total N6 PUFA**	**7**	**6,507**	**0.97 (0.94, 1.01)**	**0**
LA (18:2n6)	22	9,451	0.94 (0.90, 0.99)	45
GLA (18:3n6)	7	5,894	1.02 (0.96, 1.09)	30
EDA (20:2n6)	3	4,903	1.01 (0.96, 1.07)	0
DGLA (20:3n6)	7	5,894	1.12 (1.04, 1.21)	47
AA (20:4n6)	18	7,071	1.01 (0.96, 1.07)	33
DTA (22:4n6)	3	4,903	1.13 (0.95, 1.34)	73
DPA (22:5n6)	3	4,903	1.10 (0.96, 1.27)	53
**Total trans**				
trans-16:1	4	4,432	0.89 (0.78, 1.03)	76
trans-18:1	2	4,798	0.82 (0.64, 1.06)	77
trans-18:2	3	4,936	0.90 (0.78, 1.04)	67

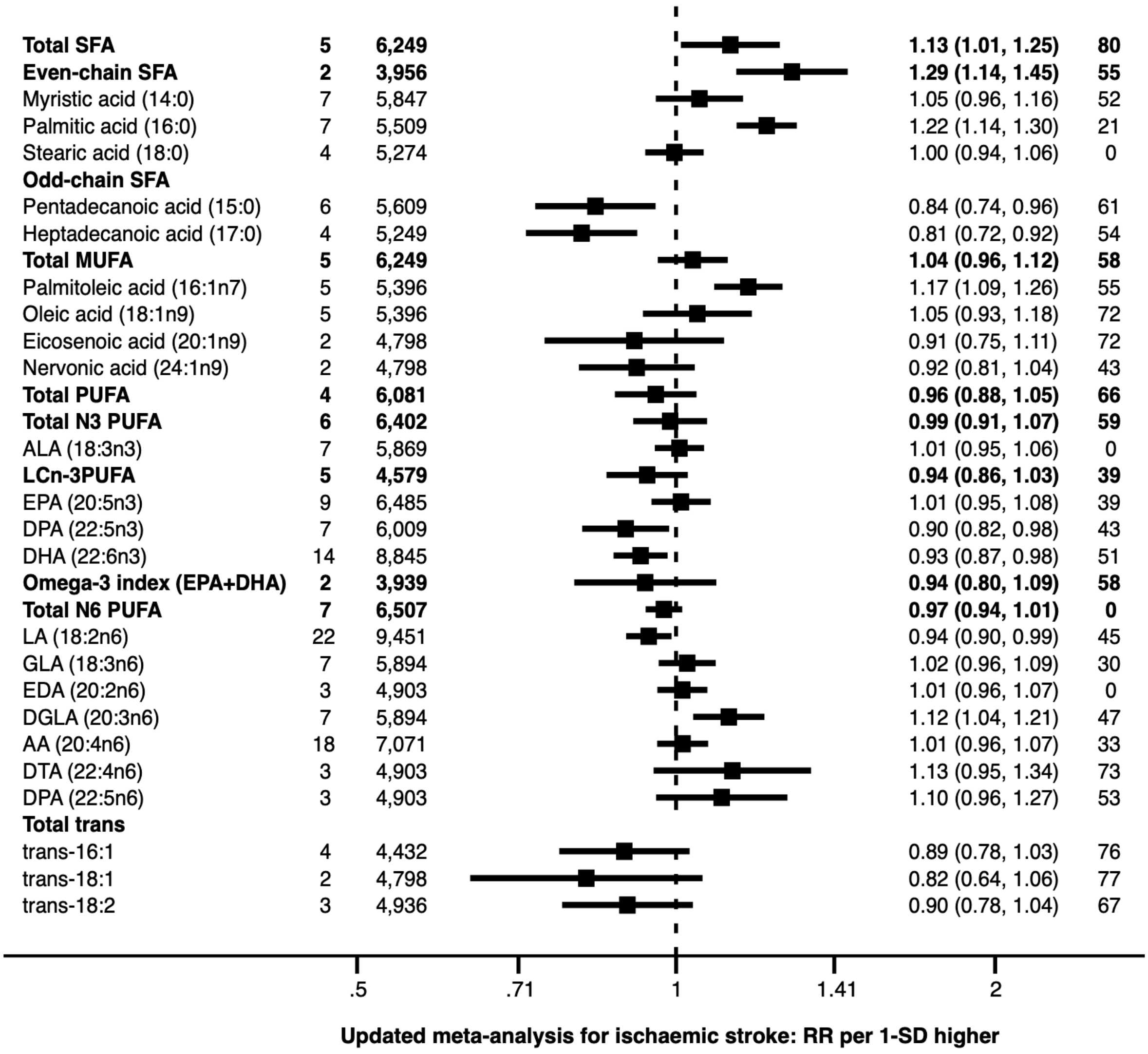

RR: Relative risk; SD: Standard deviation. For the abbreviations of fatty acids, please refer to the Supplementary eTable 4. The results of updated meta-analyses using random-effects method were summarised for the fatty acids, of which associations with ischaemic stroke were identified from existing published evidence.

Supplementary eFigure 8. Associations of FAs with CHD risk by geographical region.

Fatty acids \ subgroup	N of Data sources	N of Cases	N of Participants	Relative risk (95%CI) per 1-SD	p-value*
**Total SFA**	**8**	**13914**	**201367**	**1.14 (1.08, 1.21)**	**0.230**
Europe	6	11778	196541	1.15 (1.09, 1.22)	ref
US	1	912	2378	1.29 (1.12, 1.49)	0.470
Others	1	1224	2448	0.94 (0.85, 1.04)	0.135
**Even-chain SFA**	**3**	**8146**	**27388**	**1.24 (1.13, 1.35)**	**0.424**
Europe	2	7994	26780	1.22 (1.12, 1.34)	
Others	1	152	608	1.43 (1.11, 1.84)	
**Myristic acid (14:0)**	**7**	**9746**	**33269**	**1.06 (0.99, 1.15)**	**0.267**
Europe	3	8130	27188	1.05 (0.95, 1.15)	ref
US	3	1464	5473	1.02 (0.92, 1.14)	0.804
Others	1	152	608	1.34 (1.10, 1.64)	0.126
**Palmitic acid (16:0)**	**10**	**11315**	**37807**	**1.07 (0.99, 1.15)**	**0.454**
Europe	4	8183	28362	1.03 (0.94, 1.13)	ref
US	4	2236	7349	1.13 (0.95, 1.34)	0.407
Others	2	896	2096	1.22 (0.85, 1.75)	0.283
**Stearic acid (18:0)**	**8**	**11132**	**35861**	**1.08 (0.98, 1.19)**	**0.158**
Europe	3	8130	27188	1.14 (1.01, 1.30)	ref
US	3	2106	6577	1.03 (0.95, 1.11)	0.528
Others	2	896	2096	0.79 (0.57, 1.09)	0.063
**Pentadecanoic acid (15:0)**	**8**	**11043**	**38008**	**0.92 (0.86, 0.97)**	**0.001**
Europe	3	8173	27986	0.87 (0.83, 0.92)	
US	5	2870	10022	0.99 (0.91, 1.08)	
**Heptadecanoic acid (17:0)**	**5**	**9287**	**28573**	**0.86 (0.80, 0.93)**	**0.021**
Europe	2	7787	23836	0.82 (0.76, 0.88)	
US	3	1500	4737	0.98 (0.91, 1.04)	
**Longer-chain SFA**	**5**	**9487**	**27699**	**0.88 (0.77, 1.00)**	**0.740**
Europe	2	7479	23244	0.90 (0.75, 1.07)	
US	3	2008	4455	0.84 (0.68, 1.04)	
**Arachidic acid (20:0)**	**5**	**9497**	**27719**	**0.87 (0.78, 0.97)**	**0.787**
Europe	2	7479	23244	0.86 (0.76, 0.97)	
US	3	2018	4475	0.89 (0.75, 1.05)	
**Behenic acid (22:0)**	**6**	**10285**	**31660**	**0.94 (0.84, 1.06)**	**0.822**
Europe	2	7479	23244	0.96 (0.80, 1.15)	
US	4	2806	8416	0.92 (0.79, 1.07)	
**Lignoceric acid (24:0)**	**6**	**10285**	**31660**	**0.89 (0.81, 0.98)**	**0.973**
Europe	2	7479	23244	0.89 (0.77, 1.02)	
US	4	2806	8416	0.89 (0.77, 1.03)	
**Total MUFA**	**10**	**14717**	**205919**	**1.07 (1.00, 1.15)**	**0.352**
Europe	7	12429	200485	1.06 (0.98, 1.14)	ref
US	1	1224	2448	0.95 (0.84, 1.07)	0.487
Others	2	1064	2986	1.20 (1.10, 1.31)	0.235
**Palmitoleic acid (16:1n7)**	**8**	**10975**	**34810**	**1.06 (1.01, 1.11)**	**0.556**
Europe	3	8130	27188	1.06 (1.01, 1.12)	ref
US	3	1949	5526	1.00 (0.92, 1.10)	0.425
Others	2	896	2096	1.21 (0.87, 1.69)	0.539
**Oleic acid (18:1n9)**	**7**	**10344**	**31920**	**1.02 (0.94, 1.11)**	**0.333**
Europe	3	8130	27188	0.98 (0.90, 1.08)	ref
US	2	1318	2636	1.05 (0.57, 1.93)	0.475
Others	2	896	2096	1.15 (1.04, 1.27)	0.178
**Eicosenoic acid (20:1n9)**	**4**	**9354**	**29636**	**1.04 (0.96, 1.12)**	**0.776**
Europe	3	8130	27188	1.05 (0.94, 1.17)	
US	1	1224	2448	0.99 (0.98, 1.01)	
**Nervonic acid (24:1n9)**	**3**	**8703**	**25692**	**0.89 (0.79, 1.00)**	**0.457**
Europe	2	7479	23244	0.87 (0.76, 0.99)	
US	1	1224	2448	1.04 (0.92, 1.18)	
**Total PUFA**	**7**	**13205**	**202455**	**0.91 (0.86, 0.96)**	**0.911**
Europe	6	12293	200077	0.91 (0.86, 0.96)	
Others	1	912	2378	0.90 (0.82, 0.99)	
**Total N3 PUFA**	**7**	**13814**	**201212**	**0.91 (0.86, 0.96)**	**0.605**
Europe	4	11526	195778	0.92 (0.86, 0.98)	ref
US	1	1224	2448	0.82 (0.71, 0.93)	0.354
Others	2	1064	2986	0.92 (0.85, 1.01)	0.830
**ALA (18:3n3)**	**22**	**15868**	**58942**	**0.99 (0.95, 1.02)**	**0.158**
Europe	10	9697	31475	0.99 (0.94, 1.04)	ref
US	9	5058	20038	1.00 (0.95, 1.06)	0.693
Others	3	1113	7429	0.85 (0.74, 0.98)	0.082
**LCn-3PUFA**	**17**	**11754**	**52114**	**0.90 (0.86, 0.95)**	**0.345**
Europe	7	8848	32451	0.93 (0.87, 1.00)	ref
US	7	2203	12215	0.85 (0.76, 0.94)	0.182
Others	3	703	7448	0.86 (0.71, 1.03)	0.389
**EPA (20:5n3)**	**23**	**15944**	**59218**	**0.94 (0.89, 0.99)**	**0.619**
Europe	10	9672	31389	0.96 (0.89, 1.04)	ref
US	9	4950	19760	0.91 (0.85, 0.99)	0.378
Others	4	1322	8069	0.87 (0.68, 1.11)	0.538
**DPA (22:5n3)**	**20**	**15102**	**59104**	**0.93 (0.90, 0.97)**	**0.063**
Europe	8	9497	32552	0.95 (0.90, 0.99)	ref
US	9	5042	20038	0.89 (0.84, 0.95)	0.127
Others	3	563	6514	1.05 (0.92, 1.20)	0.139

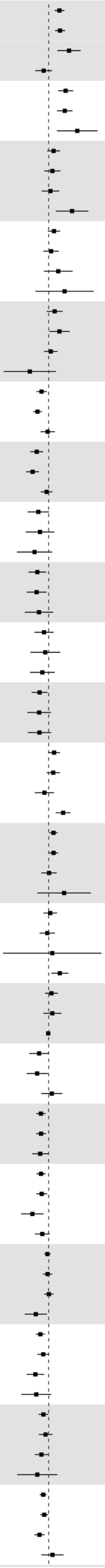

(Continued on next page)

(Continued)

**DHA (22:6n3)**	**29**	**22176**	**240041**	**0.92 (0.88, 0.95)**	**0.170**
Europe	14	14770	208784	0.95 (0.90, 1.00)	ref
US	10	5172	20810	0.86 (0.78, 0.94)	0.096
Others	5	2234	10447	0.86 (0.80, 0.93)	0.208
**Omega-3 index (EPA+DHA)**	**10**	**9243**	**29321**	**0.93 (0.86, 1.01)**	**0.781**
Europe	4	7779	23922	0.95 (0.86, 1.04)	ref
US	4	511	3284	0.88 (0.64, 1.19)	0.792
Others	2	953	2115	0.85 (0.74, 0.97)	0.500
**Total N6 PUFA**	**10**	**14720**	**208064**	**0.95 (0.89, 1.02)**	**0.141**
Europe	6	12313	200130	0.95 (0.88, 1.03)	ref
US	2	1343	4948	1.08 (0.97, 1.21)	0.192
Others	2	1064	2986	0.79 (0.57, 1.10)	0.173
**LA (18:2n6)**	**33**	**22802**	**255927**	**0.94 (0.91, 0.98)**	**0.119**
Europe	16	15132	214151	0.94 (0.90, 0.98)	ref
US	11	5335	26029	1.00 (0.97, 1.04)	0.142
Others	6	2335	15747	0.84 (0.72, 0.98)	0.276
**GLA (18:3n6)**	**7**	**10354**	**36589**	**1.06 (1.01, 1.10)**	**0.610**
Europe	3	8139	28241	1.08 (1.01, 1.15)	ref
US	3	2063	7740	1.01 (1.00, 1.02)	0.344
Others	1	152	608	1.09 (0.88, 1.35)	0.921
**EDA (20:2n6)**	**5**	**8916**	**28380**	**1.02 (0.96, 1.08)**	**0.685**
Europe	2	7479	23244	1.01 (0.94, 1.09)	
US	3	1437	5136	1.04 (0.92, 1.17)	
**DGLA (20:3n6)**	**9**	**10584**	**37185**	**1.30 (1.17, 1.45)**	**0.916**
Europe	4	8275	28649	1.31 (1.15, 1.49)	ref
US	4	2157	7928	1.33 (0.99, 1.78)	0.914
Others	1	152	608	1.19 (0.96, 1.48)	0.684
**AA (20:4n6)**	**29**	**17707**	**80607**	**0.99 (0.96, 1.02)**	**0.684**
Europe	13	10949	41209	1.00 (0.96, 1.05)	ref
US	11	5335	26029	0.98 (0.94, 1.02)	0.551
Others	5	1423	13369	0.95 (0.83, 1.09)	0.442
**DTA (22:4n6)**	**5**	**8916**	**28380**	**1.07 (1.01, 1.13)**	**0.818**
Europe	2	7479	23244	1.06 (0.98, 1.16)	
US	3	1437	5136	1.07 (0.98, 1.16)	
**DPA (22:5n6)**	**5**	**8916**	**28380**	**1.10 (1.02, 1.18)**	**0.861**
Europe	2	7479	23244	1.09 (0.97, 1.22)	
US	3	1437	5136	1.07 (1.03, 1.12)	
**Total trans**	**4**	**8947**	**26205**	**0.92 (0.80, 1.04)**	**0.045**
Europe	1	7343	22836	0.82 (0.73, 0.93)	
US	3	1604	3369	1.06 (0.91, 1.24)	
**trans-16:1**	**5**	**7800**	**22539**	**0.96 (0.88, 1.04)**	**0.547**
Europe	2	6612	16302	0.98 (0.86, 1.11)	
US	3	1188	6237	0.92 (0.82, 1.04)	
**trans-18:1**	**6**	**9827**	**28101**	**0.93 (0.85, 1.01)**	**0.679**
Europe	2	7479	23244	0.88 (0.82, 0.95)	ref
US	3	1604	3369	1.00 (0.75, 1.35)	0.443
Others	1	744	1488	0.88 (0.77, 1.00)	0.829
**trans-18:2**	**5**	**9200**	**64329**	**1.00 (0.92, 1.08)**	**0.877**
Europe	2	7596	60960	0.98 (0.87, 1.11)	
US	3	1604	3369	1.05 (0.96, 1.16)	

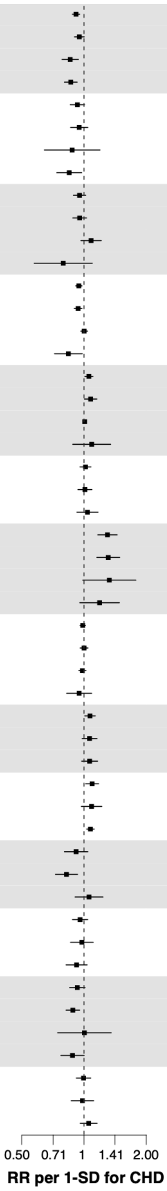

CHD: Coronary heart disease; SD: Standard deviation; US: United States. Abbreviations for fatty acids refer to Supplementary eTable 4.
* By meta-regression. Joint p value was provided if more than two subgroups for comparison.

Supplementary eFigure 9. Associations of FAs with stroke risk by geographical region.

Fatty acids \ subgroup	N of Data sources	N of Cases	N of Participants	Relative risk (95%CI) per 1-SD	p-value*
**Total SFA**	**5**	**9496**	**169199**	**1.10 (1.01, 1.21)**	**0.073**
Europe	3	8182	162717	1.13 (1.04, 1.22)	ref
US	1	1146	2612	1.22 (1.02, 1.45)	0.614
Others	1	168	3870	0.84 (0.76, 0.92)	0.032
**Even-chain SFA**	**3**	**6915**	**26164**	**1.20 (1.07, 1.34)**	**0.636**
Europe	2	6718	25376	1.18 (1.05, 1.34)	
Others	1	197	788	1.31 (1.05, 1.64)	
**Myristic acid (14:0)**	**9**	**9158**	**35655**	**1.07 (1.00, 1.14)**	**0.034**
Europe	3	7139	27689	1.01 (0.94, 1.09)	ref
US	5	1822	7178	1.13 (1.04, 1.23)	0.099
Others	1	197	788	1.34 (1.14, 1.58)	0.018
**Palmitic acid (16:0)**	**9**	**8696**	**36686**	**1.13 (1.05, 1.22)**	**0.490**
Europe	4	7190	29002	1.12 (1.02, 1.23)	ref
US	4	1309	6896	1.11 (0.95, 1.29)	0.882
Others	1	197	788	1.35 (1.14, 1.59)	0.261
**Stearic acid (18:0)**	**6**	**8367**	**33679**	**0.99 (0.93, 1.05)**	**0.374**
Europe	3	7139	27689	0.97 (0.92, 1.03)	
US	3	1228	5990	1.07 (0.89, 1.29)	
**Pentadecanoic acid (15:0)**	**8**	**9054**	**35799**	**0.88 (0.80, 0.96)**	**0.010**
Europe	3	6799	25906	0.79 (0.69, 0.90)	
US	5	2255	9893	1.00 (0.93, 1.06)	
**Heptadecanoic acid (17:0)**	**6**	**8694**	**27779**	**0.82 (0.76, 0.90)**	**0.001**
Europe	2	6607	21756	0.76 (0.70, 0.81)	
US	4	2087	6023	0.95 (0.89, 1.02)	
**Total MUFA**	**6**	**9715**	**173143**	**1.08 (1.02, 1.15)**	**0.877**
Europe	4	8401	166661	1.07 (1.00, 1.16)	ref
US	1	168	3870	1.15 (0.97, 1.36)	0.631
Others	1	1146	2612	1.06 (0.96, 1.17)	0.935
**Palmitoleic acid (16:1n7)**	**7**	**8564**	**34467**	**1.17 (1.11, 1.22)**	**0.236**
Europe	3	7139	27689	1.16 (1.09, 1.22)	ref
US	3	1228	5990	1.15 (1.03, 1.29)	0.995
Others	1	197	788	1.39 (1.17, 1.65)	0.098
**Oleic acid (18:1n9)**	**7**	**8564**	**34467**	**1.11 (1.02, 1.20)**	**0.336**
Europe	3	7139	27689	1.06 (0.95, 1.18)	ref
US	3	1228	5990	1.17 (1.04, 1.32)	0.361
Others	1	197	788	1.32 (1.11, 1.56)	0.201
**Eicosenoic acid (20:1n9)**	**3**	**7682**	**27304**	**0.99 (0.86, 1.13)**	**0.615**
Europe	2	6718	25376	0.97 (0.84, 1.13)	
US	1	964	1928	1.12 (0.81, 1.55)	
**Nervonic acid (24:1n9)**	**2**	**7463**	**23360**	**0.90 (0.80, 1.02)**	**0.887**
Europe	1	6499	21432	0.90 (0.79, 1.03)	
US	1	964	1928	0.88 (0.66, 1.17)	
**Total PUFA**	**5**	**9547**	**169273**	**0.93 (0.86, 1.00)**	**0.296**
Europe	4	8401	166661	0.91 (0.84, 0.99)	
Others	1	1146	2612	1.05 (0.96, 1.15)	
**Total N3 PUFA**	**6**	**9698**	**171027**	**0.98 (0.92, 1.04)**	**0.869**
Europe	4	8384	164545	0.97 (0.91, 1.05)	ref
US	1	1146	2612	0.94 (0.80, 1.12)	0.787
Others	1	168	3870	1.02 (0.92, 1.13)	0.683
**ALA (18:3n3)**	**8**	**8885**	**36772**	**0.99 (0.94, 1.04)**	**0.990**
Europe	3	7122	25573	0.99 (0.93, 1.05)	
US	5	1763	11199	0.99 (0.88, 1.11)	
**LCn-3PUFA**	**6**	**7599**	**36266**	**0.94 (0.88, 1.00)**	**0.418**
Europe	3	6920	27204	0.96 (0.88, 1.04)	
US	3	679	9062	0.90 (0.82, 0.99)	
**EPA (20:5n3)**	**10**	**9408**	**38612**	**1.00 (0.94, 1.06)**	**0.594**
Europe	3	7122	25573	1.01 (0.93, 1.11)	
US	7	2286	13039	0.98 (0.89, 1.07)	
**DPA (22:5n3)**	**9**	**9038**	**36373**	**0.89 (0.84, 0.96)**	**0.517**
Europe	3	6920	27204	0.91 (0.83, 1.01)	
US	6	2118	9169	0.87 (0.80, 0.94)	
**DHA (22:6n3)**	**16**	**12588**	**188672**	**0.93 (0.89, 0.97)**	**0.092**
Europe	7	9075	172115	0.97 (0.92, 1.02)	ref
US	8	2367	13945	0.86 (0.79, 0.94)	0.038
Others	1	1146	2612	0.89 (0.81, 0.98)	0.286
**Omega-3 index (EPA+DHA)**	**3**	**6973**	**25039**	**0.97 (0.87, 1.07)**	**0.203**
Europe	2	6868	22539	0.99 (0.89, 1.10)	
US	1	105	2500	0.75 (0.56, 1.01)	
**Total N6 PUFA**	**8**	**10022**	**177471**	**0.96 (0.93, 0.99)**	**0.138**
Europe	5	8603	168489	0.94 (0.91, 0.98)	ref
US	2	273	6370	0.96 (0.82, 1.11)	0.845
Others	1	1146	2612	1.05 (0.96, 1.15)	0.052
**LA (18:2n6)**	**27**	**14221**	**217960**	**0.92 (0.88, 0.96)**	**0.311**
Europe	12	9661	179822	0.91 (0.86, 0.95)	ref
US	10	2833	23532	0.98 (0.92, 1.04)	0.198
Others	5	1727	14606	0.84 (0.65, 1.09)	0.635
**GLA (18:3n6)**	**8**	**9041**	**40607**	**1.04 (0.98, 1.09)**	**0.331**
Europe	4	7341	29517	1.05 (0.99, 1.13)	
US	4	1700	11090	0.99 (0.92, 1.08)	

(Continued on next page)

(Continued)

**EDA (20:2n6)**	**4**	**7664**	**26052**	**1.00 (0.95, 1.05)**	**0.503**
Europe	1	6499	21432	0.98 (0.91, 1.06)	
US	3	1165	4620	1.04 (0.92, 1.19)	
**DGLA (20:3n6)**	**9**	**9137**	**40799**	**1.08 (1.01, 1.17)**	**0.169**
Europe	4	7341	29517	1.12 (1.02, 1.24)	
US	5	1796	11282	0.99 (0.92, 1.06)	
**AA (20:4n6)**	**24**	**11392**	**74063**	**0.99 (0.95, 1.04)**	**0.479**
Europe	10	7978	38537	1.02 (0.97, 1.06)	ref
US	10	2833	23532	0.95 (0.86, 1.04)	0.242
Others	4	581	11994	0.98 (0.78, 1.22)	0.566
**DTA (22:4n6)**	**4**	**7664**	**26052**	**1.06 (0.94, 1.20)**	**0.584**
Europe	1	6499	21432	1.08 (0.94, 1.25)	
US	3	1165	4620	1.00 (0.75, 1.32)	
**DPA (22:5n6)**	**4**	**7664**	**26052**	**1.08 (0.97, 1.20)**	**0.705**
Europe	1	6499	21432	1.09 (0.97, 1.24)	
US	3	1165	4620	1.05 (0.81, 1.35)	
**trans-16:1**	**5**	**6827**	**20116**	**0.92 (0.84, 1.01)**	**0.520**
Europe	1	4740	14093	0.89 (0.76, 1.05)	
US	4	2087	6023	1.00 (0.92, 1.08)	
**trans-18:1**	**2**	**7463**	**23360**	**0.89 (0.74, 1.07)**	**0.342**
Europe	1	6499	21432	0.85 (0.69, 1.04)	
US	1	964	1928	1.13 (0.95, 1.34)	
**trans-18:2**	**3**	**7655**	**61484**	**0.90 (0.80, 1.01)**	**0.078**
Europe	2	6691	59556	0.87 (0.78, 0.97)	
US	1	964	1928	1.34 (1.00, 1.79)	

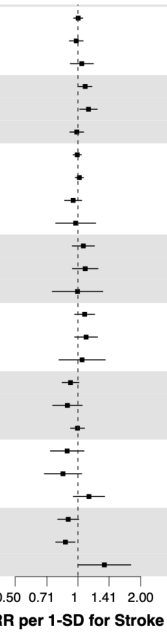

SD: Standard deviation; US: United States. Abbreviations for fatty acids refer to Supplementary eTable 4.
* By meta-regression. Joint p value was provided if more than two subgroups for comparison.

Supplementary eFigure 10. Associations of FAs with CHD risk by lipid compartments.

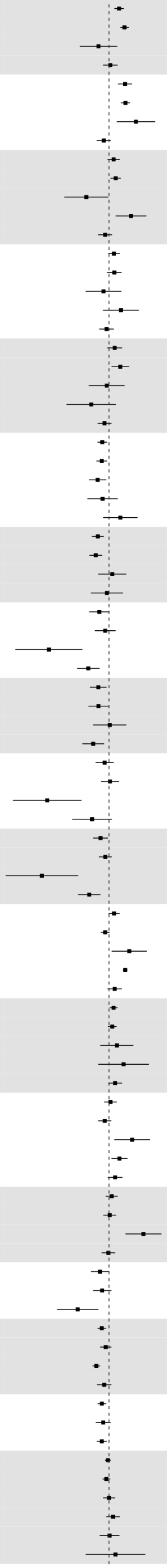

Fatty acids \ subgroup	N of Data sources	N of Cases	N of Participants	Relative risk (95%CI) per 1-SD	p-value*
**Total SFA**	**8**	**13914**	**201367**	**1.14 (1.08, 1.21)**	**0.004**
plasma/serum PL	3	8683	25639	1.23 (1.17, 1.30)	ref
RBC	1	136	408	0.87 (0.68, 1.11)	0.032
Total plasma/serum	4	5095	175320	1.02 (0.93, 1.12)	0.003
**Even-chain SFA**	**3**	**8146**	**27388**	**1.24 (1.13, 1.35)**	**0.010**
plasma/serum PL	1	7343	22836	1.24 (1.18, 1.32)	ref
Total plasma/serum	1	152	608	1.43 (1.11, 1.84)	0.354
AT	1	651	3944	0.93 (0.85, 1.02)	0.004
**Myristic acid (14:0)**	**7**	**9746**	**33269**	**1.06 (0.99, 1.15)**	**0.047**
plasma/serum PL	4	8807	28309	1.09 (1.02, 1.17)	ref
RBC	1	136	408	0.74 (0.55, 0.99)	0.039
Total plasma/serum	1	152	608	1.34 (1.10, 1.64)	0.131
AT	1	651	3944	0.95 (0.87, 1.04)	0.137
**Palmitic acid (16:0)**	**10**	**11315**	**37807**	**1.07 (0.99, 1.15)**	**0.691**
plasma/serum PL	5	9579	30185	1.07 (0.98, 1.18)	ref
RBC	1	136	408	0.93 (0.74, 1.17)	0.501
Total plasma/serum	3	949	3270	1.17 (0.93, 1.48)	0.515
AT	1	651	3944	0.97 (0.88, 1.06)	0.558
**Stearic acid (18:0)**	**8**	**11132**	**35861**	**1.08 (0.98, 1.19)**	**0.192**
plasma/serum PL	4	9449	29413	1.16 (1.04, 1.30)	ref
RBC	1	136	408	0.97 (0.77, 1.23)	0.424
Total plasma/serum	2	896	2096	0.79 (0.57, 1.09)	0.053
AT	1	651	3944	0.94 (0.86, 1.03)	0.297
**Pentadecanoic acid (15:0)**	**8**	**11043**	**38008**	**0.92 (0.86, 0.97)**	**0.416**
plasma/serum PL	5	10033	32028	0.91 (0.85, 0.97)	ref
plasma/serum CE	1	386	4150	0.86 (0.77, 0.96)	0.644
RBC	1	458	1337	0.92 (0.75, 1.12)	0.934
Total plasma/serum	1	166	493	1.16 (0.93, 1.46)	0.126
**Heptadecanoic acid (17:0)**	**5**	**9287**	**28573**	**0.86 (0.80, 0.93)**	**0.244**
plasma/serum PL	3	8663	26743	0.84 (0.77, 0.91)	ref
RBC	1	458	1337	1.04 (0.87, 1.25)	0.145
Total plasma/serum	1	166	493	0.97 (0.79, 1.20)	0.345
**Longer-chain SFA**	**5**	**9487**	**27699**	**0.88 (0.77, 1.00)**	**0.132**
plasma/serum PL	2	8557	25264	0.95 (0.83, 1.09)	ref
RBC	1	136	408	0.45 (0.29, 0.70)	0.071
Total plasma/serum	2	794	2027	0.76 (0.66, 0.88)	0.270
**Arachidic acid (20:0)**	**5**	**9497**	**27719**	**0.87 (0.78, 0.97)**	**0.668**
plasma/serum PL	2	8567	25284	0.87 (0.76, 0.99)	ref
RBC	1	136	408	1.01 (0.81, 1.25)	0.493
Total plasma/serum	2	794	2027	0.81 (0.70, 0.93)	0.645
**Behenic acid (22:0)**	**6**	**10285**	**31660**	**0.94 (0.84, 1.06)**	**0.112**
plasma/serum PL	3	9355	29225	1.01 (0.90, 1.14)	ref
RBC	1	136	408	0.44 (0.28, 0.69)	0.055
Total plasma/serum	2	794	2027	0.80 (0.62, 1.04)	0.298
**Lignoceric acid (24:0)**	**6**	**10285**	**31660**	**0.89 (0.81, 0.98)**	**0.020**
plasma/serum PL	3	9355	29225	0.95 (0.88, 1.03)	ref
RBC	1	136	408	0.41 (0.25, 0.66)	0.014
Total plasma/serum	2	794	2027	0.77 (0.67, 0.89)	0.080
**Total MUFA**	**10**	**14717**	**205919**	1.07 (1.00, 1.15)	**<0.001**
plasma/serum PL	3	8683	25639	0.95 (0.90, 1.00)	ref
RBC	1	136	408	1.31 (1.04, 1.65)	0.020
Total plasma/serum	5	5247	175928	1.24 (1.21, 1.27)	<0.001
AT	1	651	3944	1.08 (0.98, 1.18)	0.037
**Palmitoleic acid (16:1n7)**	**8**	**10975**	**34810**	**1.06 (1.01, 1.11)**	**0.833**
plasma/serum PL	4	9292	28362	1.04 (0.99, 1.10)	ref
RBC	1	136	408	1.11 (0.89, 1.38)	0.720
Total plasma/serum	2	896	2096	1.21 (0.87, 1.69)	0.451
AT	1	651	3944	1.08 (0.99, 1.18)	0.619
**Oleic acid (18:1n9)**	**7**	**10344**	**31920**	**1.02 (0.94, 1.11)**	**0.039**
plasma/serum PL	3	8661	25472	0.95 (0.87, 1.03)	ref
RBC	1	136	408	1.36 (1.08, 1.72)	0.030
Total plasma/serum	2	896	2096	1.15 (1.04, 1.27)	0.030
AT	1	651	3944	1.08 (0.99, 1.19)	0.112
**Eicosenoic acid (20:1n9)**	**4**	**9354**	**29636**	**1.04 (0.96, 1.12)**	**0.078**
plasma/serum PL	2	8567	25284	1.01 (0.93, 1.10)	ref
RBC	1	136	408	1.58 (1.25, 2.00)	0.029
AT	1	651	3944	0.99 (0.91, 1.08)	0.899
**Nervonic acid (24:1n9)**	**3**	**8703**	**25692**	**0.89 (0.79, 1.00)**	**0.236**
plasma/serum PL	2	8567	25284	0.91 (0.81, 1.03)	
RBC	1	136	408	0.66 (0.50, 0.87)	
**Total PUFA**	**7**	**13205**	**202455**	0.91 (0.86, 0.96)	**0.036**
plasma/serum PL	2	7459	23191	0.96 (0.89, 1.03)	ref
Total plasma/serum	4	5095	175320	0.85 (0.81, 0.89)	0.014
AT	1	651	3944	0.94 (0.86, 1.03)	0.751
**Total N3 PUFA**	**7**	**13814**	**201212**	**0.91 (0.86, 0.96)**	**0.938**
plasma/serum PL	2	8567	25284	0.93 (0.84, 1.02)	
Total plasma/serum	5	5247	175928	0.91 (0.85, 0.97)	
**ALA (18:3n3)**	**22**	**15868**	**58942**	**0.99 (0.95, 1.02)**	**0.666**
plasma/serum PL	9	10998	42251	0.97 (0.92, 1.01)	ref
plasma/serum CE	2	971	2445	1.00 (0.93, 1.08)	0.595
RBC	3	1255	4908	1.06 (0.97, 1.15)	0.172
Total plasma/serum	7	2438	8586	1.01 (0.89, 1.14)	0.550
AT	1	206	752	1.09 (0.74, 1.61)	0.583

(Continued on next page)

(Continued)

**LCn-3PUFA**	**17**	**11754**	**52114**	**0.90 (0.86, 0.95)**	**0.490**
plasma/serum PL	6	8605	35017	0.91 (0.84, 0.98)	ref
plasma/serum CE	1	398	2001	0.99 (0.88, 1.11)	0.470
RBC	3	1255	4908	0.95 (0.85, 1.07)	0.610
Total plasma/serum	5	773	5578	0.79 (0.68, 0.92)	0.162
AT	2	723	4610	0.90 (0.83, 0.98)	0.918
**EPA (20:5n3)**	**23**	**15944**	**59218**	**0.94 (0.89, 0.99)**	**0.347**
plasma/serum PL	10	11191	42891	0.88 (0.82, 0.95)	ref
plasma/serum CE	2	971	2445	0.99 (0.91, 1.08)	0.315
RBC	3	1255	4908	0.99 (0.84, 1.16)	0.322
Total plasma/serum	7	2346	8308	0.96 (0.83, 1.11)	0.288
AT	1	181	666	1.19 (1.06, 1.34)	0.088
**DPA (22:5n3)**	**20**	**15102**	**59104**	**0.93 (0.90, 0.97)**	**0.077**
plasma/serum PL	10	11191	42891	0.90 (0.86, 0.94)	ref
RBC	3	1255	4908	0.94 (0.86, 1.02)	0.419
Total plasma/serum	5	1564	6192	1.01 (0.81, 1.25)	0.171
AT	2	1092	5113	1.00 (0.94, 1.07)	0.013
**DHA (22:6n3)**	**29**	**22176**	**240041**	**0.92 (0.88, 0.95)**	**0.107**
plasma/serum PL	11	11321	43663	0.89 (0.83, 0.96)	ref
plasma/serum CE	2	971	2445	1.02 (0.95, 1.10)	0.118
RBC	3	1255	4908	0.96 (0.85, 1.09)	0.391
Total plasma/serum	11	7533	183906	0.88 (0.83, 0.92)	0.680
AT	2	1096	5119	1.05 (0.95, 1.17)	0.049
**Omega-3 index (EPA+DHA)**	**10**	**9243**	**29321**	**0.93 (0.86, 1.01)**	**0.815**
plasma/serum PL	6	8022	24481	0.93 (0.84, 1.02)	ref
plasma/serum CE	1	222	444	1.06 (0.88, 1.28)	0.508
RBC	2	255	2908	0.95 (0.72, 1.25)	0.852
Total plasma/serum	1	744	1488	0.83 (0.70, 0.98)	0.562
**Total N6 PUFA**	**10**	**14720**	**208064**	**0.95 (0.89, 1.02)**	**0.021**
plasma/serum PL	2	8567	25284	1.04 (0.97, 1.12)	ref
RBC	2	255	2908	1.07 (0.84, 1.36)	0.811
Total plasma/serum	5	5247	175928	0.85 (0.79, 0.92)	0.004
AT	1	651	3944	0.94 (0.86, 1.03)	0.308
**LA (18:2n6)**	**33**	**22802**	**255927**	**0.94 (0.91, 0.98)**	**0.485**
plasma/serum PL	12	11205	44859	0.97 (0.92, 1.02)	ref
plasma/serum CE	3	1064	6586	0.91 (0.84, 0.97)	0.353
RBC	4	1739	10171	1.00 (0.95, 1.06)	0.527
Total plasma/serum	12	7677	189157	0.91 (0.85, 0.97)	0.203
AT	2	1117	5154	0.94 (0.87, 1.03)	0.624
**GLA (18:3n6)**	**7**	**10354**	**36589**	**1.06 (1.01, 1.10)**	**0.318**
plasma/serum PL	3	9287	28076	1.07 (1.01, 1.13)	ref
RBC	1	119	2500	0.97 (0.78, 1.21)	0.470
Total plasma/serum	2	297	2069	1.13 (1.00, 1.28)	0.522
AT	1	651	3944	0.94 (0.86, 1.03)	0.126
**EDA (20:2n6)**	**5**	**8916**	**28380**	**1.02 (0.96, 1.08)**	**0.241**
plasma/serum PL	3	8661	25472	1.00 (0.94, 1.07)	
RBC	2	255	2908	1.11 (0.96, 1.29)	
**DGLA (20:3n6)**	**9**	**10584**	**37185**	**1.30 (1.17, 1.45)**	**0.103**
plasma/serum PL	4	9381	28264	1.41 (1.24, 1.60)	ref
RBC	2	255	2908	1.15 (0.98, 1.36)	0.210
Total plasma/serum	2	297	2069	1.12 (0.99, 1.28)	0.150
AT	1	651	3944	0.96 (0.87, 1.05)	0.039
**AA (20:4n6)**	**29**	**17707**	**80607**	**0.99 (0.96, 1.02)**	**0.677**
plasma/serum PL	12	11205	44859	1.00 (0.95, 1.05)	ref
plasma/serum CE	3	1064	6586	1.01 (0.94, 1.09)	0.817
RBC	4	1739	10171	0.99 (0.96, 1.02)	0.644
Total plasma/serum	8	2582	13837	0.93 (0.85, 1.03)	0.179
AT	2	1117	5154	0.99 (0.95, 1.05)	0.959
**DTA (22:4n6)**	**5**	**8916**	**28380**	**1.07 (1.01, 1.13)**	**0.200**
plasma/serum PL	3	8661	25472	1.06 (1.02, 1.09)	
RBC	2	255	2908	0.95 (0.57, 1.59)	
**DPA (22:5n6)**	**5**	**8916**	**28380**	**1.10 (1.02, 1.18)**	**0.055**
plasma/serum PL	3	8661	25472	1.09 (1.05, 1.13)	
RBC	2	255	2908	0.87 (0.51, 1.47)	
**Total trans**	**4**	**8947**	**26205**	**0.92 (0.80, 1.04)**	**0.029**
plasma/serum PL	3	8781	25712	0.88 (0.77, 0.99)	
RBC	1	166	493	1.48 (1.11, 1.97)	
**trans-16:1**	**5**	**7800**	**22539**	**0.96 (0.88, 1.04)**	**0.439**
plasma/serum PL	3	7498	21638	0.96 (0.87, 1.05)	ref
RBC	1	136	408	1.15 (0.93, 1.42)	0.380
Total plasma/serum	1	166	493	0.80 (0.65, 0.98)	0.398
**trans-18:1**	**6**	**9827**	**28101**	**0.93 (0.85, 1.01)**	**0.031**
plasma/serum PL	3	8781	25712	0.89 (0.82, 0.97)	ref
RBC	2	302	901	1.27 (1.04, 1.57)	0.011
Total plasma/serum	1	744	1488	0.88 (0.77, 1.00)	0.928
**trans-18:2**	**5**	**9200**	**64329**	**1.00 (0.92, 1.08)**	**0.896**
plasma/serum PL	3	8781	25712	0.98 (0.90, 1.08)	ref
RBC	1	166	493	1.36 (1.00, 1.86)	0.698
Total plasma/serum	1	253	38124	0.96 (0.85, 1.09)	0.836

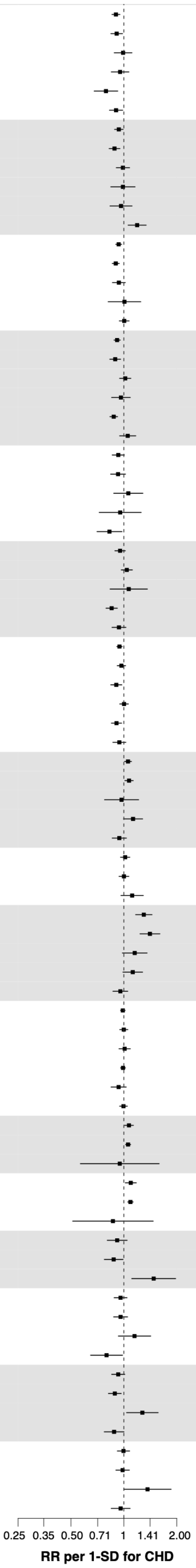

AT: Adipose tissue; CE: Cholesteryl ester; CHD: Coronary heart disease; PL: Phospholipids; RBC: Red blood cell; SD: Standard deviation. Abbreviations for fatty acids refer to Supplementary eTable 4.

* By meta-regression. Joint p value was provided if more than two subgroups for comparison.

Supplementary eFigure 11. Associations of FAs with stroke risk by lipid compartments.

Fatty acids \ subgroup	N of Data sources	N of Cases	N of Participants	Relative risk (95%CI) per 1-SD	p-value*
**Total SFA**	**5**	**9496**	**169199**	**1.10 (1.01, 1.21)**	**0.048**
plasma/serum PL	2	6667	25302	1.18 (1.07, 1.29)	
Total plasma/serum	3	2829	143897	0.97 (0.83, 1.14)	
**Even-chain SFA**	**3**	**6915**	**26164**	**1.20 (1.07, 1.34)**	**0.191**
plasma/serum PL	1	6499	21432	1.23 (1.10, 1.38)	ref
Total plasma/serum	1	197	788	1.31 (1.05, 1.64)	0.741
AT	1	219	3944	0.91 (0.78, 1.06)	0.091
**Myristic acid (14:0)**	**9**	**9158**	**35655**	**1.07 (1.00, 1.14)**	**0.087**
plasma/serum PL	3	6763	25494	1.02 (0.94, 1.11)	ref
plasma/serum CE	1	421	2313	1.07 (0.97, 1.18)	0.633
Total plasma/serum	4	1755	3904	1.21 (1.11, 1.32)	0.032
AT	1	219	3944	0.92 (0.79, 1.08)	0.355
**Palmitic acid (16:0)**	**9**	**8696**	**36686**	**1.13 (1.05, 1.22)**	**0.046**
plasma/serum PL	4	6844	26400	1.14 (1.05, 1.24)	ref
plasma/serum CE	1	421	2313	1.11 (1.00, 1.23)	0.728
Total plasma/serum	3	1212	4029	1.28 (1.15, 1.43)	0.192
AT	1	219	3944	0.84 (0.72, 0.99)	0.020
**Stearic acid (18:0)**	**6**	**8367**	**33679**	**0.99 (0.93, 1.05)**	**0.848**
plasma/serum PL	3	6763	25494	1.02 (0.93, 1.11)	ref
plasma/serum CE	1	421	2313	0.98 (0.88, 1.09)	0.755
Total plasma/serum	1	964	1928	0.93 (0.77, 1.12)	0.550
AT	1	219	3944	0.93 (0.80, 1.08)	0.508
**Pentadecanoic acid (15:0)**	**8**	**9054**	**35799**	**0.88 (0.80, 0.96)**	**0.130**
plasma/serum PL	4	7304	28533	0.82 (0.72, 0.92)	ref
plasma/serum CE	1	192	4150	0.94 (0.81, 1.10)	0.422
Total plasma/serum	3	1558	3116	1.04 (0.94, 1.15)	0.052
**Heptadecanoic acid (17:0)**	**6**	**8694**	**27779**	**0.82 (0.76, 0.90)**	**0.071**
plasma/serum PL	3	7136	24663	0.79 (0.71, 0.87)	
Total plasma/serum	3	1558	3116	0.95 (0.86, 1.05)	
**Total MUFA**	**6**	**9715**	**173143**	**1.08 (1.02, 1.15)**	**0.515**
plasma/serum PL	2	6667	25302	1.05 (0.94, 1.17)	ref
Total plasma/serum	3	2829	143897	1.07 (1.02, 1.11)	0.822
AT	1	219	3944	1.24 (1.05, 1.45)	0.263
**Palmitoleic acid (16:1n7)**	**7**	**8564**	**34467**	**1.17 (1.11, 1.22)**	**0.654**
plasma/serum PL	3	6763	25494	1.16 (1.10, 1.23)	ref
plasma/serum CE	1	421	2313	1.17 (1.06, 1.30)	0.975
Total plasma/serum	2	1161	2716	1.25 (1.02, 1.54)	0.420
AT	1	219	3944	1.06 (0.91, 1.24)	0.407
**Oleic acid (18:1n9)**	**7**	**8564**	**34467**	**1.11 (1.02, 1.20)**	**0.449**
plasma/serum PL	3	6763	25494	1.04 (0.93, 1.17)	ref
plasma/serum CE	1	421	2313	1.22 (1.10, 1.35)	0.322
Total plasma/serum	2	1161	2716	1.25 (1.11, 1.41)	0.180
AT	1	219	3944	1.17 (1.00, 1.37)	0.509
**Eicosenoic acid (20:1n9)**	**3**	**7682**	**27304**	**0.99 (0.86, 1.13)**	**0.562**
plasma/serum PL	1	6499	21432	0.94 (0.79, 1.12)	ref
Total plasma/serum	1	964	1928	1.12 (0.81, 1.55)	0.543
AT	1	219	3944	1.18 (1.02, 1.36)	0.357
**Nervonic acid (24:1n9)**	**2**	**7463**	**23360**	**0.90 (0.80, 1.02)**	**0.887**
plasma/serum PL	1	6499	21432	0.90 (0.79, 1.03)	
Total plasma/serum	1	964	1928	0.88 (0.66, 1.17)	
**Total PUFA**	**5**	**9547**	**169273**	0.93 (0.86, 1.00)	**0.860**
plasma/serum PL	1	6499	21432	0.92 (0.80, 1.07)	ref
Total plasma/serum	3	2829	143897	0.96 (0.88, 1.05)	0.693
AT	1	219	3944	0.88 (0.75, 1.03)	0.794
**Total N3 PUFA**	**6**	**9698**	**171027**	**0.98 (0.92, 1.04)**	**0.684**
plasma/serum PL	2	6667	25302	0.97 (0.88, 1.06)	
Total plasma/serum	4	3031	145725	0.99 (0.91, 1.07)	
**ALA (18:3n3)**	**8**	**8885**	**36772**	**0.99 (0.94, 1.04)**	**0.524**
plasma/serum PL	4	7193	28203	0.99 (0.93, 1.07)	ref
plasma/serum CE	1	421	2313	1.01 (0.92, 1.11)	0.859
RBC	1	105	2500	1.13 (0.88, 1.47)	0.424
Total plasma/serum	2	1166	3756	0.91 (0.80, 1.03)	0.267
**LCn-3PUFA**	**6**	**7599**	**36266**	**0.94 (0.88, 1.00)**	**0.650**
plasma/serum PL	3	7073	27994	0.95 (0.88, 1.04)	ref
RBC	1	105	2500	0.79 (0.59, 1.05)	0.301
Total plasma/serum	1	202	1828	0.98 (0.84, 1.14)	0.862
AT	1	219	3944	0.87 (0.75, 1.02)	0.503
**EPA (20:5n3)**	**10**	**9408**	**38612**	**1.00 (0.94, 1.06)**	**0.905**
plasma/serum PL	4	7279	29169	1.01 (0.93, 1.09)	ref
plasma/serum CE	1	421	2313	1.05 (0.95, 1.16)	0.723
RBC	3	542	3374	1.00 (0.74, 1.37)	0.591
Total plasma/serum	2	1166	3756	0.98 (0.81, 1.19)	0.810
**DPA (22:5n3)**	**9**	**9038**	**36373**	**0.89 (0.84, 0.96)**	**0.872**
plasma/serum PL	3	7111	25299	0.89 (0.80, 0.98)	ref
RBC	3	542	3374	0.97 (0.82, 1.16)	0.454
Total plasma/serum	2	1166	3756	0.87 (0.66, 1.14)	0.900
AT	1	219	3944	0.89 (0.76, 1.04)	0.984

(Continued on next page)

(Continued)

**DHA (22:6n3)**	**16**	**12588**	**188672**	**0.93 (0.89, 0.97)**	**0.639**
plasma/serum PL	5	7360	30075	0.94 (0.88, 1.00)	ref
plasma/serum CE	1	421	2313	1.01 (0.91, 1.12)	0.429
RBC	3	542	3374	0.86 (0.65, 1.14)	0.241
Total plasma/serum	6	4046	148966	0.93 (0.86, 1.00)	0.916
AT	1	219	3944	0.90 (0.77, 1.05)	0.717
**Omega-3 index (EPA+DHA)**	**3**	**6973**	**25039**	**0.97 (0.87, 1.07)**	**0.696**
plasma/serum PL	1	6499	21432	0.98 (0.86, 1.11)	
RBC	2	474	3607	0.91 (0.66, 1.26)	
**Total N6 PUFA**	**8**	**10022**	**177471**	0.96 (0.93, 0.99)	**0.810**
plasma/serum PL	2	6667	25302	0.94 (0.88, 1.00)	ref
RBC	1	105	2500	0.91 (0.67, 1.25)	0.837
Total plasma/serum	4	3031	145725	0.96 (0.90, 1.03)	0.592
AT	1	219	3944	0.89 (0.76, 1.05)	0.579
**LA (18:2n6)**	**27**	**14221**	**217960**	**0.92 (0.88, 0.96)**	**0.795**
plasma/serum PL	10	7669	40405	0.92 (0.87, 0.98)	ref
plasma/serum CE	2	576	6463	0.86 (0.78, 0.93)	0.550
RBC	2	400	7763	1.01 (0.89, 1.13)	0.376
Total plasma/serum	11	5171	158175	0.91 (0.83, 0.99)	0.879
AT	2	405	5154	0.97 (0.75, 1.25)	0.646
**GLA (18:3n6)**	**8**	**9041**	**40607**	**1.04 (0.98, 1.09)**	**0.724**
plasma/serum PL	3	7130	28094	1.04 (0.97, 1.12)	ref
plasma/serum CE	1	421	2313	1.07 (0.97, 1.19)	0.775
RBC	1	105	2500	0.93 (0.73, 1.18)	0.475
Total plasma/serum	2	1166	3756	0.98 (0.85, 1.13)	0.550
AT	1	219	3944	1.15 (0.99, 1.34)	0.389
**EDA (20:2n6)**	**4**	**7664**	**26052**	**1.00 (0.95, 1.05)**	**0.895**
plasma/serum PL	2	6595	21624	0.99 (0.92, 1.06)	ref
RBC	1	105	2500	1.05 (0.83, 1.34)	0.684
Total plasma/serum	1	964	1928	1.02 (0.86, 1.21)	0.810
**DGLA (20:3n6)**	**9**	**9137**	**40799**	**1.08 (1.01, 1.17)**	**0.224**
plasma/serum PL	4	7226	28286	1.15 (1.05, 1.27)	ref
plasma/serum CE	1	421	2313	1.06 (0.96, 1.17)	0.510
RBC	1	105	2500	0.96 (0.73, 1.27)	0.339
Total plasma/serum	2	1166	3756	0.98 (0.87, 1.10)	0.139
AT	1	219	3944	0.85 (0.72, 1.01)	0.059
**AA (20:4n6)**	**24**	**11392**	**74063**	**0.99 (0.95, 1.04)**	**0.262**
plasma/serum PL	10	7669	40405	1.03 (0.97, 1.09)	ref
plasma/serum CE	2	576	6463	0.99 (0.91, 1.08)	0.792
RBC	2	400	7763	1.13 (0.98, 1.30)	0.313
Total plasma/serum	8	2342	14278	0.92 (0.81, 1.04)	0.094
AT	2	405	5154	0.95 (0.85, 1.06)	0.325
**DTA (22:4n6)**	**4**	**7664**	**26052**	**1.06 (0.94, 1.20)**	**0.521**
plasma/serum PL	2	6595	21624	1.05 (0.92, 1.21)	ref
RBC	1	105	2500	1.31 (0.99, 1.72)	0.378
Total plasma/serum	1	964	1928	0.91 (0.68, 1.21)	0.536
**DPA (22:5n6)**	**4**	**7664**	**26052**	**1.08 (0.97, 1.20)**	**0.257**
plasma/serum PL	2	6595	21624	1.10 (0.98, 1.23)	ref
RBC	1	105	2500	1.28 (0.97, 1.69)	0.436
Total plasma/serum	1	964	1928	0.86 (0.72, 1.03)	0.173
**trans-16:1**	**5**	**6827**	**20116**	**0.92 (0.84, 1.01)**	**0.822**
plasma/serum PL	2	5269	17000	0.91 (0.81, 1.03)	
Total plasma/serum	3	1558	3116	0.96 (0.84, 1.10)	
**trans-18:1**	**2**	**7463**	**23360**	**0.89 (0.74, 1.07)**	**0.342**
plasma/serum PL	1	6499	21432	0.85 (0.69, 1.04)	
Total plasma/serum	1	964	1928	1.13 (0.95, 1.34)	
**trans-18:2**	**3**	**7655**	**61484**	**0.90 (0.80, 1.01)**	**0.208**
plasma/serum PL	1	6499	21432	0.86 (0.76, 0.98)	
Total plasma/serum	2	1156	40052	1.08 (0.75, 1.57)	

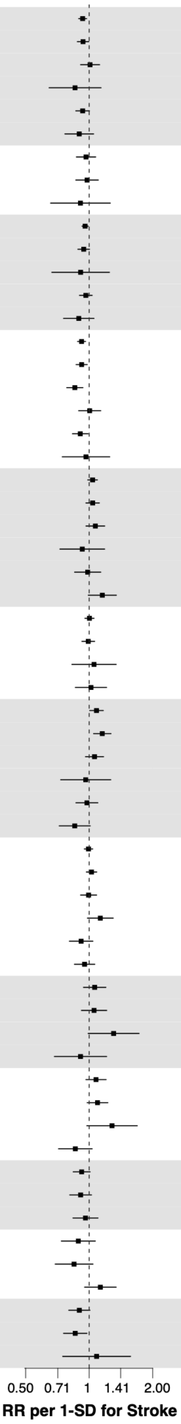

AT: Adipose tissue; CE: Cholesteryl ester; PL: Phospholipids; RBC: Red blood cell; SD: Standard deviation. Abbreviations for fatty acids refer to Supplementary eTable 4.

* By meta-regression. Joint p value was provided if more than two subgroups for comparison.

Reference

1. Hodson L, Skeaff CM, Fielding BA. Fatty acid composition of adipose tissue and blood in humans and its use as a biomarker of dietary intake. *Prog Lipid Res*. 2008;47(5):348-380. doi:10.1016/j.plipres.2008.03.003

2. Wang L, Summerhill K, Rodriguez-Canas C, et al. Development and validation of a robust automated analysis of plasma phospholipid fatty acids for metabolic phenotyping of large epidemiological studies. *Genome Med*. 2013;5(4):39. doi:10.1186/gm443

3. Browning LM, Walker CG, Mander AP, et al. Incorporation of eicosapentaenoic and docosahexaenoic acids into lipid pools when given as supplements providing doses equivalent to typical intakes of oily fish. *Am J Clin Nutr*. 2012;96(4):748-758. doi:10.3945/ajcn.112.041343

4. Chow LS, Li S, Eberly LE, et al. Estimated plasma stearoyl co-A desaturase-1 activity and risk of incident diabetes: The Atherosclerosis Risk in Communities (ARIC) study. *Metabolism*. 2013;62(1):100-108. doi:10.1016/j.metabol.2012.06.004

5. Telle-Hansen VH, Larsen LN, Høstmark AT, et al. Daily Intake of Cod or Salmon for 2 Weeks Decreases the 18:1n-9/18:0 Ratio and Serum Triacylglycerols in Healthy Subjects. *Lipids*. 2012;47(2):151-160. doi:10.1007/s11745-011-3637-y

6. Vessby B, Gustafsson I-B, Tengblad S, Berglund L. Indices of fatty acid desaturase activity in healthy human subjects: effects of different types of dietary fat. *Br J Nutr*. 2013;110(5):871-879. doi:10.1017/S0007114512005934

7. Warensjö E, Rosell M, Hellenius M-L, Vessby B, De Faire U, Risérus U. Associations between estimated fatty acid desaturase activities in serum lipids and adipose tissue in humans: links to obesity and insulin resistance. *Lipids Health Dis*. 2009;8(1):37. doi:10.1186/1476-511X-8-37

8. Forouhi NG, Imamura F, Sharp SJ, et al. Association of Plasma Phospholipid n-3 and n-6 Polyunsaturated Fatty Acids with Type 2 Diabetes: The EPIC-InterAct Case-Cohort Study. Ma RCW, ed. *PLOS Med*. 2016;13(7):e1002094. doi:10.1371/journal.pmed.1002094

9. Borges MC, Schmidt AF, Jefferis B, et al. Circulating Fatty Acids and Risk of Coronary Heart Disease and Stroke: Individual Participant Data Meta-Analysis in Up to 16 126 Participants. *J Am Heart Assoc*. 2020;9(5):e013131. doi:10.1161/JAHA.119.013131

10. Soininen P, Kangas AJ, Würtz P, Suna T, Ala-Korpela M. Quantitative Serum Nuclear Magnetic Resonance Metabolomics in Cardiovascular Epidemiology and Genetics. *Circ Cardiovasc Genet*. 2015;8(1):192-206. doi:10.1161/CIRCGENETICS.114.000216

11. Buchanan CDC, Lust CAC, Burns JL, et al. Analysis of major fatty acids from matched plasma and serum samples reveals highly comparable absolute and relative levels. *Prostaglandins Leukot Essent Fat Acids*. 2021. doi:10.1016/j.plefa.2021.102268

12. Thesing CS, Bot M, Milaneschi Y, Giltay EJ, Penninx BWJH. Omega-3 and omega-6 fatty acid levels in depressive and anxiety disorders. *Psychoneuroendocrinology*. 2018;87:53-62. doi:10.1016/j.psyneuen.2017.10.005

13. Würtz P, Havulinna AS, Soininen P, et al. Metabolite Profiling and Cardiovascular Event Risk. *Circulation*. 2015;131(9):774-785. doi:10.1161/CIRCULATIONAHA.114.013116

14. CHEN M, LIN V. Different Types of Dietary Fats and Health. Food Safety Focus– Food Safety Platform. https://www.cfs.gov.hk/english/multimedia/multimedia_pub/multimedia_pub_fsf_158_02.html. Published 2019. Accessed May 13, 2024.

15. Wang Z, Wang DH, Goykhman Y, et al. The elongation of very long-chain fatty acid 6 gene product catalyses elongation of n -13 : 0 and n -15 : 0 odd-chain SFA in human cells. *Br J Nutr*. 2019;121(3):241-248. doi:10.1017/S0007114518003185

16. Sassa T, Kihara A. Metabolism of Very Long-Chain Fatty Acids: Genes and Pathophysiology. *Biomol Ther (Seoul)*. 2014;22(2):83-92. doi:10.4062/biomolther.2014.017

17. Sun S, Wang Y, Goh P-T, et al. Evolution and Functional Characteristics of the Novel elovl8 That Play Pivotal Roles in Fatty Acid Biosynthesis. *Genes (Basel)*. 2021;12(8):1287. doi:10.3390/genes12081287

18. Peters T, Brage S, Westgate K, et al. Validity of a short questionnaire to assess physical activity in 10 European countries. *Eur J Epidemiol*. 2012;27(1):15-25. doi:10.1007/s10654-011-9625-y

19. IPAQ Group (2005). Guidelines for Data Processing and Analysis of the International Physical Activity Questionnaire (IPAQ) – Short and Long Forms. http://www.ipaq.ki.se.

20. Who WHO. Global recommendations on physical activity for health. *Geneva World Heal Organ*. 2010.

21. Mingyang S, Xin Z, Mathew P, Donna S. The missing covariate indicator method is nearly valid almost always. Paper presented at 2016 Epidemiology Congress of the Americas; June 21, 2016; Miami, Florida.

22. CEU. Stata programs-Cardiovascular Epidemiology Unit. http://www.phpc.cam.ac.uk/ceu/erfc/programs/.

23. Li Z, Lei H, Jiang H, et al. Saturated fatty acid biomarkers and risk of cardiometabolic diseases: A meta-analysis of prospective studies. *Front Nutr*. 2022;9(August):1-11. doi:10.3389/fnut.2022.963471

24. Clarke R, Shipley M, Armitage J, Collins R, Harris W. Plasma phospholipid fatty acids and CHD in older men: Whitehall study of London civil servants. *Br J Nutr*. 2008;102(2):279-284. doi:10.1017/S0007114508143562

25. Chei C-L, Yamagishi K, Kitamura A, et al. Serum Fatty Acid and Risk of Coronary Artery Disease — Circulatory Risk in Communities Study (CIRCS) —. *Circ J*. 2018;82(12):3013-3020. doi:10.1253/circj.CJ-18-0240

26. Malik VS, Chiuve SE, Campos H, et al. Circulating Very-Long-Chain Saturated Fatty Acids and Incident Coronary Heart Disease in US Men and Women. *Circulation*. 2015;132(4):260-268. doi:10.1161/CIRCULATIONAHA.114.014911

27. Sun Y, Koh HWL, Choi H, et al. Plasma fatty acids, oxylipins, and risk of myocardial infarction: The Singapore Chinese Health Study. *J Lipid Res*. 2016;57(7):1300-1307. doi:10.1194/jlr.P066423

28. Liu Q, Matthan NR, Manson JE, et al. Plasma Phospholipid Fatty Acids and Coronary Heart Disease Risk: A Matched Case-Control Study within the Women's Health Initiative Observational Study. *Nutrients*. 2019;11(7):1672. doi:10.3390/nu11071672

29. Iso H, Sato S, Umemura U, et al. Linoleic Acid, Other Fatty Acids, and the Risk of Stroke. *Stroke*. 2002;33(8):2086-2093. doi:10.1161/01.STR.0000023890.25066.50

30. Wiberg B, Sundström J, Arnlöv J, et al. Metabolic Risk Factors for Stroke and Transient Ischemic Attacks in Middle-Aged Men. *Stroke*. 2006;37(12):2898-2903. doi:10.1161/01.STR.0000249056.24657.8b

31. Warensjö E, Smedman A, Stegmayr B, et al. Stroke and plasma markers of milk fat intake – a prospective nested case-control study. *Nutr J*. 2009;8(1):21. doi:10.1186/1475-2891-8-21

32. Yaemsiri S, Sen S, Tinker LF, et al. Serum Fatty Acids and Incidence of Ischemic Stroke Among Postmenopausal Women. *Stroke*. 2013;44(10):2710-2717. doi:10.1161/STROKEAHA.111.000834

33. Yamagishi K, Folsom AR, Steffen LM. Plasma Fatty Acid Composition and Incident Ischemic Stroke in Middle-Aged Adults: The Atherosclerosis Risk in Communities (ARIC) Study. *Cerebrovasc Dis*. 2013;36(1):38-46. doi:10.1159/000351205

34. Yakoob MY, Shi P, Hu FB, et al. Circulating biomarkers of dairy fat and risk of incident stroke in U.S. men and women in 2 large prospective cohorts > *Am J Clin Nutr*. 2014;100(6):1437-1447. doi:10.3945/ajcn.114.083097

35. de Oliveira Otto MC, Lemaitre RN, Song X, King IB, Siscovick DS, Mozaffarian D. Serial measures of circulating biomarkers of dairy fat and total and cause-specific mortality in older adults: the Cardiovascular Health Study. *Am J Clin Nutr*. 2018;108(3):476-484. doi:10.1093/ajcn/nqy117

36. Woodward M, Tunstall-Pedoe H, Batty GD, Tavendale R, Hu FB, Czernichow S. The prognostic value of adipose tissue fatty acids for incident cardiovascular disease: results from 3944 subjects in the Scottish Heart Health Extended Cohort Study. *Eur Heart J*. 2011;32(11):1416-1423. doi:10.1093/eurheartj/ehr036

37. Fretts AM, Mozaffarian D, Siscovick DS, et al. Associations of Plasma Phospholipid SFAs with Total and Cause-Specific Mortality in Older Adults Differ According to SFA Chain Length. *J Nutr*. 2016;146(2):298-305. doi:10.3945/jn.115.222117

38. Trieu K, Bhat S, Dai Z, et al. Biomarkers of dairy fat intake, incident cardiovascular disease, and all-cause mortality: A cohort study, systematic review, and meta-analysis. *PLOS Med*. 2021;18(9):e1003763. doi:10.1371/journal.pmed.1003763

39. Simon JA, Hodgkins ML, Browner WS, Neuhaus JM, Bernert JT, Hulley SB. Serum Fatty Acids and the Risk of Coronary Heart Disease. *Am J Epidemiol*. 1995;142(5):469-476. doi:10.1093/oxfordjournals.aje.a117662

40. Sun Q, Ma J, Campos H, Hu FB. Plasma and erythrocyte biomarkers of dairy fat intake and risk of ischemic heart disease. *Am J Clin Nutr*. 2007;86(4):929-937. doi:10.1093/ajcn/86.4.929

41. Warensjö E, Jansson J-H, Cederholm T, et al. Biomarkers of milk fat and the risk of myocardial infarction in men and women: a prospective, matched case-control study. *Am J Clin Nutr*. 2010;92(1):194-202. doi:10.3945/ajcn.2009.29054

42. Wu JHY, Lemaitre RN, Imamura F, et al. Fatty acids in the de novo lipogenesis pathway and risk of coronary heart disease: the Cardiovascular Health Study. *Am J Clin Nutr*. 2011;94(2):431-438. doi:10.3945/ajcn.111.012054

43. Warensjö E, Jansson J-H, Berglund L, et al. Estimated intake of milk fat is negatively associated with cardiovascular risk factors and does not increase the risk of a first acute myocardial infarction. A prospective case–control study. *Br J Nutr*. 2004;91(4):635-642. doi:10.1079/BJN20041080

44. Khaw K-T, Friesen MD, Riboli E, Luben R, Wareham N. Plasma Phospholipid Fatty Acid Concentration and Incident Coronary Heart Disease in Men and Women: The EPIC-Norfolk Prospective Study. Katan MB, ed. *PLoS Med*. 2012;9(7):e1001255. doi:10.1371/journal.pmed.1001255

45. Huang NK, Biggs ML, Matthan NR, et al. Serum Nonesterified Fatty Acids and Incident Stroke: The CHS. *J Am Heart Assoc*. 2021;10(22):e022725. doi:10.1161/JAHA.121.022725

46. Chien K-L, Lin H-J, Hsu H-C, et al. Comparison of predictive performance of various fatty acids for the risk of cardiovascular disease events and all-cause deaths in a community-based cohort. *Atherosclerosis*. 2013;230(1):140-147. doi:10.1016/j.atherosclerosis.2013.06.015

47. Warensjö E, Sundström J, Vessby B, Cederholm T, Risérus U. Markers of dietary fat quality and fatty acid desaturation as predictors of total and cardiovascular mortality: a population-based prospective study. *Am J Clin Nutr*. 2008;88(1):203-209. doi:10.1093/ajcn/88.1.203

48. Wang L, Folsom AR, Eckfeldt JH, the ARIC Study Investigators. Plasma fatty acid composition and incidence of coronary heart disease in middle aged adults: The Atherosclerosis Risk in Communities (ARIC) Study. *Nutr Metab Cardiovasc Dis*. 2003;13(5):256-266. doi:10.1016/S0939-4753(03)80029-7

49. Jiang H, Wang L, Wang D, et al. Omega-3 polyunsaturated fatty acid biomarkers and risk of type 2 diabetes, cardiovascular disease, cancer, and mortality. *Clin Nutr*. 2022;41(8):1798-1807. doi:10.1016/j.clnu.2022.06.034

50. Harris WS, Tintle NL, Etherton MR, Vasan RS. Erythrocyte long-chain omega-3 fatty acid levels are inversely associated with mortality and with incident cardiovascular disease: The Framingham Heart Study. *J Clin Lipidol*. 2018;12(3):718-727.e6. doi:10.1016/j.jacl.2018.02.010

51. Lemaitre RN, King IB, Mozaffarian D, Kuller LH, Tracy RP, Siscovick DS. n–3 Polyunsaturated fatty acids, fatal ischemic heart disease, and nonfatal myocardial infarction in older adults: the Cardiovascular Health Study. *Am J Clin Nutr*. 2003;77(2):319-325. doi:10.1093/ajcn/77.2.319

52. Sun Q, Ma J, Campos H, et al. Blood concentrations of individual long-chain n–3 fatty acids and risk of nonfatal myocardial infarction. *Am J Clin Nutr*. 2008;88(1):216-223. doi:10.1093/ajcn/88.1.216

53. Joensen AM, Overvad K, Dethlefsen C, et al. Marine n-3 Polyunsaturated Fatty Acids in Adipose Tissue and the Risk of Acute Coronary Syndrome. *Circulation*. 2011;124(11):1232-1238. doi:10.1161/CIRCULATIONAHA.110.987057

54. Matsumoto C, Matthan NR, Wilk JB, Lichtenstein AH, Gaziano JM, Djoussé L. Erythrocyte stearidonic acid and other n -3 fatty acids and CHD in the Physicians' Health Study. *Br J Nutr*. 2013;109(11):2044-2049. doi:10.1017/S0007114512004060

55. Sun Y, Koh W-P, Yuan J-M, et al. Plasma α-Linolenic and Long-Chain ω-3 Fatty Acids Are Associated with a Lower Risk of Acute Myocardial Infarction in Singapore Chinese Adults. *J Nutr*. 2016;146(2):275-282. doi:10.3945/jn.115.220418

56. Hamazaki K, Iso H, Eshak ES, et al. Plasma levels of n-3 fatty acids and risk of coronary heart disease among Japanese: The Japan Public Health Center-based (JPHC) study. *Atherosclerosis*. 2018;272:226-232. doi:10.1016/j.atherosclerosis.2017.12.004

57. De Goede J, Verschuren WMM, Boer JMA, Kromhout D, Geleijnse JM. N-6 and n-3 fatty acid cholesteryl esters in relation to incident stroke in a Dutch adult population: A nested case–control study. *Nutr Metab Cardiovasc Dis*. 2013;23(8):737-743. doi:10.1016/j.numecd.2012.03.001

58. Daneshmand R, Kurl S, Tuomainen T-P, Virtanen JK. Associations of serum n -3 and n -6 PUFA and hair mercury with the risk of incident stroke in men: the Kuopio Ischaemic Heart Disease Risk Factor Study (KIHD). *Br J Nutr*. 2016;115(10):1851-1859. doi:10.1017/S0007114516000982

59. Saber H, Yakoob MY, Shi P, et al. Omega-3 Fatty Acids and Incident Ischemic Stroke and Its Atherothrombotic and Cardioembolic Subtypes in 3 US Cohorts. *Stroke*. 2017;48(10):2678-2685. doi:10.1161/STROKEAHA.117.018235

60. Bork CS, Venø SK, Lundbye-Christensen S, et al. Adipose tissue content of alpha-linolenic acid and the risk of ischemic stroke and ischemic stroke subtypes: A Danish case-cohort study. Loor JJ, ed. *PLoS One*. 2018;13(6):e0198927. doi:10.1371/journal.pone.0198927

61. Venø SK, Bork CS, Jakobsen MU, et al. Marine n-3 Polyunsaturated Fatty Acids and the Risk of Ischemic Stroke. *Stroke*. 2019;50(2):274-282. doi:10.1161/STROKEAHA.118.023384

62. Mozaffarian D, Lemaitre RN, King IB, et al. Plasma Phospholipid Long-Chain ω-3 Fatty Acids and Total and Cause-Specific Mortality in Older Adults. *Ann Intern Med*. 2013;158(7):515. doi:10.7326/0003-4819-158-7-201304020-00003

63. de Oliveira Otto MC, Wu JHY, Baylin A, et al. Circulating and Dietary Omega-3 and Omega-6 Polyunsaturated Fatty Acids and Incidence of CVD in the Multi-Ethnic Study of Atherosclerosis. *J Am Heart Assoc*. 2013;2(6):e000506. doi:10.1161/JAHA.113.000506

64. Fretts AM, Mozaffarian D, Siscovick DS, et al. Plasma phospholipid and dietary α-linolenic acid, mortality, CHD and stroke: the Cardiovascular Health Study. *Br J Nutr*. 2014;112(7):1206-1213. doi:10.1017/S0007114514001925

65. Marklund M, Leander K, Vikström M, et al. Polyunsaturated Fat Intake Estimated by Circulating Biomarkers and Risk of Cardiovascular Disease and All-Cause Mortality in a Population-Based Cohort of 60-Year-Old Men and Women. *Circulation*. 2015;132(7):586-594. doi:10.1161/CIRCULATIONAHA.115.015607

66. Albert CM, Campos H, Stampfer MJ, et al. Blood Levels of Long-Chain n–3 Fatty Acids and the Risk of Sudden Death. *N Engl J Med*. 2002;346(15):1113-1118. doi:10.1056/NEJMoa012918

67. Laaksonen DE. Prediction of Cardiovascular Mortality in Middle-aged Men by Dietary and Serum Linoleic and Polyunsaturated Fatty Acids. *Arch Intern Med*. 2005;165(2):193. doi:10.1001/archinte.165.2.193

68. Virtanen JK, Laukkanen JA, Mursu J, Voutilainen S, Tuomainen T-P. Serum Long-Chain n-3 Polyunsaturated Fatty Acids, Mercury, and Risk of Sudden Cardiac Death in Men: A Prospective Population-Based Study. Moormann AM, ed. *PLoS One*. 2012;7(7):e41046. doi:10.1371/journal.pone.0041046

69. Harris WS, Luo J, Pottala J V., et al. Red blood cell polyunsaturated fatty acids and mortality in the Women's Health Initiative Memory Study. *J Clin Lipidol*. 2017;11(1):250-259.e5. doi:10.1016/j.jacl.2016.12.013

70. Zhang Y, Guo X, Gao J, et al. The associations of circulating common and uncommon polyunsaturated fatty acids and modification effects on dietary quality with all-cause and disease-specific mortality in NHANES 2003–2004 and 2011–2012. *Ann Med*. 2021;53(1):1744-1757. doi:10.1080/07853890.2021.1937693

71. Ren X-L, Liu Y, Chu W-J, et al. Blood levels of omega-6 fatty acids and coronary heart disease: a systematic review and metaanalysis of observational epidemiology. *Crit Rev Food Sci Nutr*. 2022;0(0):1-13. doi:10.1080/10408398.2022.2056867

72. Satizabal CL, Samieri C, Davis-Plourde KL, et al. APOE and the Association of Fatty Acids With the Risk of Stroke, Coronary Heart Disease, and Mortality. *Stroke*. 2018;49(12):2822-2829. doi:10.1161/STROKEAHA.118.022132

73. Virtanen JK, Wu JHY, Voutilainen S, Mursu J, Tuomainen T-P. Serum n–6 polyunsaturated fatty acids and risk of death: the Kuopio Ischaemic Heart Disease Risk Factor Study. *Am J Clin Nutr*. 2018;107(3):427-435. doi:10.1093/ajcn/nqx063

74. Papandreou C, Sala-Vila A, Galié S, et al. Association Between Fatty Acids of Blood Cell Membranes and Incidence of Coronary Heart Disease. *Arterioscler Thromb Vasc Biol*. 2019;39(4):819-825. doi:10.1161/ATVBAHA.118.312073

75. de Goede J, Verschuren WMM, Boer JMA, Verberne LDM, Kromhout D, Geleijnse JM. N-6 and N-3 Fatty Acid Cholesteryl Esters in Relation to Fatal CHD in a Dutch Adult Population: A Nested Case-Control Study and Meta-Analysis. *PLoS One*. 2013. doi:10.1371/journal.pone.0059408

76. Matthan NR, Ooi EM, Van Horn L, Neuhouser ML, Woodman R, Lichtenstein AH. Plasma phospholipid fatty acid biomarkers of dietary fat quality and endogenous metabolism predict coronary heart disease risk: a nested case-control study within the Women's Health Initiative observational study. *J Am Heart Assoc*. 2014;3(4). doi:10.1161/JAHA.113.000764

77. Wu JHY, Lemaitre RN, King IB, et al. Circulating Omega-6 Polyunsaturated Fatty Acids and Total and Cause-Specific Mortality. *Circulation*. 2014;130(15):1245-1253. doi:10.1161/CIRCULATIONAHA.114.011590

78. Harris W, Tintle N, Ramachandran V. Erythrocyte n-6 Fatty Acids and Risk for Cardiovascular Outcomes and Total Mortality in the Framingham Heart Study. *Nutrients*. 2018;10(12):2012. doi:10.3390/nu10122012

79. Kirkeby K, Ingvaldsen P, Bjerkedal I. FATTY ACID COMPOSITION OF SERUM LIPIDS IN MEN WITH MYOCARDIAL INFARCTION. *Acta Med Scand*. 1972;192(1-6):513-519. doi:10.1111/j.0954-6820.1972.tb04857.x

80. SKULADOTTIR G, HARDARSON T, SIGFUSSON N, ODDSSON G, GUDBJARNASON S. Arachidonic Acid Levels in Serum Phospholipids of Patients with Angina Pectoris or Fatal Myocardial Infarction. *Acta Med Scand*. 2009;218(1):55-58. doi:10.1111/j.0954-6820.1985.tb08824.x

81. Hadj Ahmed S, Kaoubaa N, Kharroubi W, et al. Association of plasma fatty acid alteration with the severity of coronary artery disease lesions in Tunisian patients. *Lipids Health Dis*. 2017;16(1):154. doi:10.1186/s12944-017-0538-y

82. Luostarinena R, Boberg M, Saldeen T. Fatty acid composition in total phospholipids of human coronary arteries in sudden cardiac death. *Atherosclerosis*. 1993;99(2):187-193. doi:10.1016/0021-9150(93)90021-L

83. Öhrvall M, Berglund L, Salminen I, Lithell H, Aro A, Vessby B. The serum cholesterol ester fatty acid composition but not the serum concentration of alpha tocopherol predicts the development of myocardial infarction in 50-year-old men: 19 years follow-up. *Atherosclerosis*. 1996;127(1):65-71. doi:10.1016/S0021-9150(96)05936-9

84. YLI-JAMA P, MEYER HE, RINGSTAD J, PEDERSEN JI. Serum free fatty acid pattern and risk of myocardial infarction: a case-control study. *J Intern Med*. 2002;251(1):19-28. doi:10.1046/j.1365-2796.2002.00922.x

85. Lemaitre RN, King IB, Raghunathan TE, et al. Cell Membrane Trans -Fatty Acids and the Risk of Primary Cardiac Arrest. *Circulation*. 2002;105(6):697-701. doi:10.1161/hc0602.103583

86. Oda E, Hatada K, Katoh K, Kodama M, Nakamura Y, Aizawa Y. A Case-control Pilot Study on n-3 Polyunsaturated Fatty Acid as a Negative Risk Factor for Myocardial Infarction. *Int Heart J*. 2005;46(4):583-591. doi:10.1536/ihj.46.583

87. Freije A. Fatty Acid Profile of the Erythrocyte Membranes of Healthy Bahraini Citizens in Comparison with Coronary Heart Disease Patients. *J Oleo Sci*. 2009;58(7):379-388. doi:10.5650/jos.58.379

88. Marangoni F, Novo G, Perna G, et al. Omega-6 and omega-3 polyunsaturated fatty acid levels are reduced in whole blood of Italian patients with a recent myocardial infarction: the AGE-IM study. *Atherosclerosis*. 2014;232(2):334-338. doi:10.1016/j.atherosclerosis.2013.11.048

89. Lemaitre RN, King IB, Rice K, et al. Erythrocyte very long-chain saturated fatty Acids associated with lower risk of incident sudden cardiac arrest. *Prostaglandins, Leukot Essent Fat Acids*. 2014;91(4):149-153. doi:10.1016/j.plefa.2014.07.010

90. Erkkilä AT, Lehto S, Pyörälä K, Uusitupa MIJ. n–3 Fatty acids and 5-y risks of death and cardiovascular

disease events in patients with coronary artery disease. *Am J Clin Nutr*. 2003;78(1):65-71. doi:10.1093/ajcn/78.1.65

91. Harris WS, Kennedy KF, O'Keefe JH, Spertus JA. Red blood cell fatty acid levels improve GRACE score prediction of 2-yr mortality in patients with myocardial infarction. *Int J Cardiol*. 2013. doi:10.1016/j.ijcard.2012.09.076

92. Delgado GE, März W, Lorkowski S, von Schacky C, Kleber ME. Omega-6 fatty acids: Opposing associations with risk—The Ludwigshafen Risk and Cardiovascular Health Study. *J Clin Lipidol*. 2017;11(4):1082-1090.e14. doi:10.1016/j.jacl.2017.05.003

93. Huang NK, Bůžková P, Matthan NR, et al. Associations of Serum Nonesterified Fatty Acids With Coronary Heart Disease Mortality and Nonfatal Myocardial Infarction: The CHS (Cardiovascular Health Study) Cohort. *J Am Heart Assoc*. 2021;10(6):e019135. doi:10.1161/JAHA.120.019135

94. Yang W-S, Chen Y-Y, Chen P-C, et al. Association between Plasma N-6 Polyunsaturated Fatty Acids Levels and the Risk of Cardiovascular Disease in a Community-based Cohort Study. *Sci Rep*. 2019;9(1):19298. doi:10.1038/s41598-019-55686-7

95. Malik VS, Wu H, Hu FB, Sun Q. Abstract MP006: Plasma and Erythrocyte Biomarkers of Dairy Fat Intake and Risk of Coronary Heart Disease in Men. *Circulation*. 2012;125(suppl_10). doi:10.1161/circ.125.suppl_10.AMP006

96. de Oliveira Otto MC, Nettleton JA, Lemaitre RN, et al. Biomarkers of Dairy Fatty Acids and Risk of Cardiovascular Disease in the Multi-Ethnic Study of Atherosclerosis. *J Am Heart Assoc*. 2013;2(4):e000092. doi:10.1161/JAHA.113.000092

97. Matthan NR, Ooi EM, Horn L Van, Neuhouser ML, Woodman R, Lichtenstein AH. Plasma phospholipid fatty acid biomarkers of dietary fat quality and endogenous metabolism predict coronary heart disease risk: A nested case-control study within the women's health initiative observational study. *J Am Heart Assoc*. 2014. doi:10.1161/JAHA.113.000764

98. Laursen ASD, Dahm CC, Johnsen SP, Schmidt EB, Overvad K, Jakobsen MU. Adipose tissue fatty acids present in dairy fat and risk of stroke: the Danish Diet, Cancer and Health cohort. *Eur J Nutr*. 2019;58(2):529-539. doi:10.1007/s00394-018-1608-2

99. Yamagishi K, Nettleton JA, Folsom AR. Plasma fatty acid composition and incident heart failure in middle-aged adults: The Atherosclerosis Risk in Communities (ARIC) Study. *Am Heart J*. 2008;156(5):965-974. doi:10.1016/j.ahj.2008.06.017

100. Matsumoto C, Hanson NQ, Tsai MY, Glynn RJ, Gaziano JM, Djoussé L. Plasma phospholipid saturated fatty acids and heart failure risk in the physicians' health study. *Clin Nutr*. 2013;32(5):819-823. doi:10.1016/j.clnu.2013.02.006

101. Tokede OA, Petrone AB, Hanson NQ, et al. Plasma phospholipid trans fatty acids and risk of heart failure. *Am J Clin Nutr*. 2013;97(4):698-705. doi:10.3945/ajcn.112.050120

102. Iggman D, Ärnlöv J, Cederholm T, Risérus U. Association of Adipose Tissue Fatty Acids With Cardiovascular and All-Cause Mortality in Elderly Men. *JAMA Cardiol*. 2016;1(7):745. doi:10.1001/jamacardio.2016.2259

103. Naghshi S, Aune D, Beyene J, Mobarak S, Asadi M, Sadeghi O. Dietary intake and biomarkers of alpha linolenic acid and risk of all cause, cardiovascular, and cancer mortality: systematic review and dose-response meta-analysis of cohort studies. *BMJ*. 2021;375:n2213. doi:10.1136/bmj.n2213

104. Bork CS, Jakobsen MU, Lundbye-Christensen S, Tjønneland A, Schmidt EB, Overvad K. Dietary intake and adipose tissue content of α-linolenic acid and risk of myocardial infarction: a Danish cohort study. *Am J Clin Nutr*. 2016;104(1):41-48. doi:10.3945/ajcn.115.127019

105. Del Gobbo LC, Imamura F, Aslibekyan S, et al. ω-3 Polyunsaturated Fatty Acid Biomarkers and Coronary Heart Disease: Pooling Project of 19 Cohort Studies. *JAMA Intern Med*. 2016;176(8):1155-1166. doi:10.1001/jamainternmed.2016.2925

106. Kleber ME, Delgado GE, Lorkowski S, März W, von Schacky C. Omega-3 fatty acids and mortality in patients referred for coronary angiography. The Ludwigshafen Risk and Cardiovascular Health Study. *Atherosclerosis*. 2016;252:175-181. doi:10.1016/j.atherosclerosis.2016.06.049

107. Lelli D, Antonelli Incalzi R, Ferrucci L, Bandinelli S, Pedone C. Association between PUFA intake and serum concentration and mortality in older adults: A cohort study. *Clin Nutr*. 2020;39(2):510-515.

doi:10.1016/j.clnu.2019.02.030

108. Harris WS, Tintle NL, Imamura F, et al. Blood n-3 fatty acid levels and total and cause-specific mortality from 17 prospective studies. *Nat Commun*. 2021;12(1):2329. doi:10.1038/s41467-021-22370-2

109. Liu M, Zuo L-S-Y, Sun T-Y, et al. Circulating Very-Long-Chain Saturated Fatty Acids Were Inversely Associated with Cardiovascular Health: A Prospective Cohort Study and Meta-Analysis. *Nutrients*. 2020;12(9):2709. doi:10.3390/nu12092709

110. Chung H-K, Cho Y, Do HJ, Oh K, Seo W-K, Shin M-J. Plasma phospholipid arachidonic acid and lignoceric acid are associated with the risk of cardioembolic stroke. *Nutr Res*. 2015;35(11):1001-1008. doi:10.1016/j.nutres.2015.09.007

111. Kleber ME, Delgado GE, Dawczynski C, Lorkowski S, März W, von Schacky C. Saturated fatty acids and mortality in patients referred for coronary angiography—The Ludwigshafen Risk and Cardiovascular Health study. *J Clin Lipidol*. 2018;12(2):455-463.e3. doi:10.1016/j.jacl.2018.01.007

112. Lemaitre RN, McKnight B, Sotoodehnia N, et al. Circulating Very Long-Chain Saturated Fatty Acids and Heart Failure: The Cardiovascular Health Study. *J Am Heart Assoc*. 2018;7(21):e010019. doi:10.1161/JAHA.118.010019

113. Fretts AM, Mozaffarian D, Siscovick DS, et al. Plasma Phospholipid Saturated Fatty Acids and Incident Atrial Fibrillation: The Cardiovascular Health Study. *J Am Heart Assoc*. 2014;3(3):e000889. doi:10.1161/JAHA.114.000889

114. Zhang W, Zhou F, Huang H, Mao Y, Ye D. Biomarker of dietary linoleic acid and risk for stroke: A systematic review and meta-analysis. *Nutrition*. 2020;79-80:110953. doi:10.1016/j.nut.2020.110953

115. Venø SK, Bork CS, Jakobsen MU, et al. Linoleic Acid in Adipose Tissue and Development of Ischemic Stroke: A Danish Case-Cohort Study. *J Am Heart Assoc*. 2018;7(13). doi:10.1161/JAHA.118.009820

116. Park Y, Park S, Yi H, et al. Low level of n-3 polyunsaturated fatty acids in erythrocytes is a risk factor for both acute ischemic and hemorrhagic stroke in Koreans. *Nutr Res*. 2009;29(12):825-830. doi:10.1016/j.nutres.2009.10.018

117. Li J, Guasch-Ferré M, Li Y, Hu FB. Dietary intake and biomarkers of linoleic acid and mortality: systematic review and meta-analysis of prospective cohort studies. *Am J Clin Nutr*. 2020;112(1):150-167. doi:10.1093/ajcn/nqz349

118. Marklund M, Wu JHY, Imamura F, et al. Biomarkers of Dietary Omega-6 Fatty Acids and Incident Cardiovascular Disease and Mortality. *Circulation*. 2019;139(21):2422-2436. doi:10.1161/CIRCULATIONAHA.118.038908

119. Kilander L, Berglund L, Boberg M, Vessby B, Lithell H. Education, lifestyle factors and mortality from cardiovascular disease and cancer. A 25-year follow-up of Swedish 50-year-old men. *Int J Epidemiol*. 2001;30(5):1119-1126. doi:10.1093/ije/30.5.1119

120. Yang B, Ren X-L, Huang H, Guo X-J, Ma A-G, Li D. Circulating long-chain n-3 polyunsaturated fatty acid and incidence of stroke: a meta-analysis of prospective cohort studies. *Oncotarget*. 2017;8(48):83781-83791. doi:10.18632/oncotarget.19530

121. Simon JA, Fong J, Bernert JT, Browner WS. Serum Fatty Acids and the Risk of Stroke. *Stroke*. 1995;26(5):778-782. doi:10.1161/01.STR.26.5.778

122. Wennberg M, Bergdahl IA, Stegmayr B, et al. Fish intake, mercury, long-chain n -3 polyunsaturated fatty acids and risk of stroke in northern Sweden. *Br J Nutr*. 2007;98(5):1038-1045. doi:10.1017/S0007114507756519

123. Virtanen JK, Siscovick DS, Lemaitre RN, et al. Circulating Omega-3 Polyunsaturated Fatty Acids and Subclinical Brain Abnormalities on MRI in Older Adults: The Cardiovascular Health Study. *J Am Heart Assoc*. 2013;2(5):e000305. doi:10.1161/JAHA.113.000305

124. Fezeu LK, Laporte F, Kesse-Guyot E, et al. Baseline Plasma Fatty Acids Profile and Incident Cardiovascular Events in the SU.FOL.OM3 Trial: The Evidence Revisited. Lionetti V, ed. *PLoS One*. 2014;9(4):e92548. doi:10.1371/journal.pone.0092548

125. Chowdhury R, Warnakula S, Kunutsor S, et al. Association of Dietary, Circulating, and Supplement Fatty Acids With Coronary Risk. *Ann Intern Med*. 2014;160(6):398. doi:10.7326/M13-1788

126. Lemaitre RN, King IB, Mozaffarian D, et al. Plasma Phospholipid Trans Fatty Acids, Fatal Ischemic

Heart Disease, and Sudden Cardiac Death in Older Adults. *Circulation*. 2006;114(3):209-215. doi:10.1161/CIRCULATIONAHA.106.620336

127. Hallgren CG, Hallmans G, Jansson J-H, et al. Markers of high fish intake are associated with decreased risk of a first myocardial infarction. *Br J Nutr*. 2001;86(3):397-404. doi:10.1079/BJN2001415

128. Sun Q, Ma J, Campos H, et al. A Prospective Study of Trans Fatty Acids in Erythrocytes and Risk of Coronary Heart Disease. *Circulation*. 2007;115(14):1858-1865. doi:10.1161/CIRCULATIONAHA.106.679985

129. Domei T, Yokoi H, Kuramitsu S, et al. Ratio of serum n-3 to n-6 polyunsaturated fatty acids and the incidence of major adverse cardiac events in patients undergoing percutaneous coronary intervention. *Circ J*. 2012. doi:10.1253/circj.CJ-11-0941

130. Pilz S, Scharnagl H, Tiran B, et al. Free Fatty Acids Are Independently Associated with All-Cause and Cardiovascular Mortality in Subjects with Coronary Artery Disease. *J Clin Endocrinol Metab*. 2006;91(7):2542-2547. doi:10.1210/jc.2006-0195

131. Pilz S, Scharnagl H, Tiran B, et al. Elevated plasma free fatty acids predict sudden cardiac death: a 6.85-year follow-up of 3315 patients after coronary angiography. *Eur Heart J*. 2007;28(22):2763-2769. doi:10.1093/eurheartj/ehm343

132. Itakura H, Yokoyama M, Matsuzaki M, et al. Relationships between Plasma Fatty Acid Composition and Coronary Artery Disease. *J Atheroscler Thromb*. 2011;18(2):99-107. doi:10.5551/jat.5876

133. Rissanen T, Voutilainen S, Nyyssönen K, Lakka TA, Salonen JT. Fish Oil–Derived Fatty Acids, Docosahexaenoic Acid and Docosapentaenoic Acid, and the Risk of Acute Coronary Events. *Circulation*. 2000;102(22):2677-2679. doi:10.1161/01.CIR.102.22.2677

134. Aline Charles M, Fontbonne A, Thibult N, et al. High Plasma Nonesterified Fatty Acids Are Predictive of Cancer Mortality but Not of Coronary Heart Disease Mortality: Results from the Paris Prospective Study. *Am J Epidemiol*. 2001;153(3):292-298. doi:10.1093/aje/153.3.292

135. Jouven X, Charles M-A, Desnos M, Ducimetière P. Circulating Nonesterified Fatty Acid Level as a Predictive Risk Factor for Sudden Death in the Population. *Circulation*. 2001;104(7):756-761. doi:10.1161/hc3201.094151

136. Pirro M, Mauriège P, Tchernof A, et al. Plasma free fatty acid levels and the risk of ischemic heart disease in men: prospective results from the Québec Cardiovascular Study. *Atherosclerosis*. 2002;160(2):377-384. doi:10.1016/S0021-9150(01)00588-3

137. Tavendale R, Lee AJ, Smith WCS, Tunstall-Pedoe H. Adipose tissue fatty acids in Scottish men and women: results from the Scottish Heart Health Study. *Atherosclerosis*. 1992;94(2-3):161-169. doi:10.1016/0021-9150(92)90241-8

138. Guallar E, Hennekens CH, Sacks FM, Willett WC, Stampfer MJ. A prospective study of plasma fish oil levels and incidence of myocardial infarction in U.S. male physicians. *J Am Coll Cardiol*. 1995;25(2):387-394. doi:10.1016/0735-1097(94)00370-6

139. Pan A, Chen M, Chowdhury R, et al. α-Linolenic acid and risk of cardiovascular disease: a systematic review and meta-analysis. *Am J Clin Nutr*. 2012;96(6):1262-1273. doi:10.3945/ajcn.112.044040

140. Törnwall ME, Salminen I, Aro A, et al. Effect of serum and dietary fatty acids on the short-term risk of acute myocardial infarction in male smokers. *Nutr Metab Cardiovasc Dis*. 1996.

141. Guallar E, Aro A, Jiménez FJ, et al. Omega-3 Fatty Acids in Adipose Tissue and Risk of Myocardial Infarction. *Arterioscler Thromb Vasc Biol*. 1999;19(4):1111-1118. doi:10.1161/01.ATV.19.4.1111

142. Pedersen J, Ringstad J, Almendingen K, Haugen T, Stensvold I, Thelle D. Adipose tissue fatty acids and risk of myocardial infarction—a case-control study. *Eur J Clin Nutr*. 2000;54(8):618-625. doi:10.1038/sj.ejcn.1601064

143. Kark JD, Kaufmann NA, Binka F, Goldberger N, Berry EM. Adipose tissue n–6 fatty acids and acute myocardial infarction in a population consuming a diet high in polyunsaturated fatty acids. *Am J Clin Nutr*. 2003;77(4):796-802. doi:10.1093/ajcn/77.4.796

144. Lemaitre RN, King IB, Sotoodehnia N, et al. Red blood cell membrane α-linolenic acid and the risk of sudden cardiac arrest. *Metabolism*. 2009;58(4):534-540. doi:10.1016/j.metabol.2008.11.013

145. Shearer GC, Pottala J V., Spertus JA, Harris WS. Red Blood Cell Fatty Acid Patterns and Acute

Coronary Syndrome. Tomé D, ed. *PLoS One*. 2009;4(5):e5444. doi:10.1371/journal.pone.0005444

146. Lopes C, Aro A, Azevedo A, Ramos E, Barros H. Intake and Adipose Tissue Composition of Fatty Acids and Risk of Myocardial Infarction in a Male Portuguese Community Sample. *J Am Diet Assoc*. 2007;107(2):276-286. doi:10.1016/j.jada.2006.11.008

147. Campos H, Baylin A, Willett WC. α-Linolenic Acid and Risk of Nonfatal Acute Myocardial Infarction. *Circulation*. 2008;118(4):339-345. doi:10.1161/CIRCULATIONAHA.107.762419

148. Chowdhury R, Stevens S, Gorman D, et al. Association between fish consumption, long chain omega 3 fatty acids, and risk of cerebrovascular disease: systematic review and meta-analysis. *BMJ*. 2012;345(oct30 3):e6698-e6698. doi:10.1136/bmj.e6698

149. Tikkanen E, Jägerroos V, Holmes M V., et al. Metabolic Biomarker Discovery for Risk of Peripheral Artery Disease Compared With Coronary Artery Disease: Lipoprotein and Metabolite Profiling of 31 657 Individuals From 5 Prospective Cohorts. *J Am Heart Assoc*. 2021;10(23):e021995. doi:10.1161/JAHA.121.021995

150. Holmes M V., Millwood IY, Kartsonaki C, et al. Lipids, Lipoproteins, and Metabolites and Risk of Myocardial Infarction and Stroke. *J Am Coll Cardiol*. 2018;71(6):620-632. doi:10.1016/j.jacc.2017.12.006

151. Abdelhamid AS, Brown TJ, Brainard JS, et al. Omega-3 fatty acids for the primary and secondary prevention of cardiovascular disease. *Cochrane Database Syst Rev*. 2020;2020(3):CD003177. doi:10.1002/14651858.CD003177.pub5

152. Hooper L, Martin N, Jimoh OF, Kirk C, Foster E, Abdelhamid AS. Reduction in saturated fat intake for cardiovascular disease. *Cochrane Database Syst Rev*. 2020;5(5):CD011737. doi:10.1002/14651858.CD011737.pub2

153. Hooper L, Al-Khudairy L, Abdelhamid AS, et al. Omega-6 fats for the primary and secondary prevention of cardiovascular disease. *Cochrane database Syst Rev*. 2018;7(7):CD011094. doi:10.1002/14651858.CD011094.pub3

154. Abdelhamid AS, Martin N, Bridges C, et al. Polyunsaturated fatty acids for the primary and secondary prevention of cardiovascular disease. *Cochrane Database Syst Rev*. 2018;11(11):CD012345. doi:10.1002/14651858.CD012345.pub3
